# Molecular characteristics and spatial distribution of adult human corneal cell subtypes

**DOI:** 10.1038/s41598-021-94933-8

**Published:** 2021-08-11

**Authors:** Ann J. Ligocki, Wen Fury, Christian Gutierrez, Christina Adler, Tao Yang, Min Ni, Yu Bai, Yi Wei, Guillermo L. Lehmann, Carmelo Romano

**Affiliations:** Regeneron Pharmaceuticals, Inc., Tarrytown, NY 10591 USA

**Keywords:** RNA sequencing, Functional clustering, Gene expression, Visual system

## Abstract

Bulk RNA sequencing of a tissue captures the gene expression profile from all cell types combined. Single-cell RNA sequencing identifies discrete cell-signatures based on transcriptomic identities. Six adult human corneas were processed for single-cell RNAseq and 16 cell clusters were bioinformatically identified. Based on their transcriptomic signatures and RNAscope results using representative cluster marker genes on human cornea cross-sections, these clusters were confirmed to be stromal keratocytes, endothelium, several subtypes of corneal epithelium, conjunctival epithelium, and supportive cells in the limbal stem cell niche. The complexity of the epithelial cell layer was captured by eight distinct corneal clusters and three conjunctival clusters. These were further characterized by enriched biological pathways and molecular characteristics which revealed novel groupings related to development, function, and location within the epithelial layer. Moreover, epithelial subtypes were found to reflect their initial generation in the limbal region, differentiation, and migration through to mature epithelial cells. The single-cell map of the human cornea deepens the knowledge of the cellular subsets of the cornea on a whole genome transcriptional level. This information can be applied to better understand normal corneal biology, serve as a reference to understand corneal disease pathology, and provide potential insights into therapeutic approaches.

## Introduction

The human cornea serves as a barrier against the external environment and provides the main refractive power to focus light to the retina. The cornea has five distinctive layers, three composed by cellular elements (epithelium, stroma, endothelium) and two by extracellular membranes (Bowman’s and Descemet’s membranes). The molecular, biomechanical and structural attributes of each layer is associated with characteristic cellular composition. The outermost stratified epithelium provides a protective barrier between the environment and the anterior chamber of the eye. Superficial epithelial cell exuviation necessitates renewal by epithelial stem cells located in the corneal limbus (LESC) that differentiate into early progenitor and transient amplifying cells which continually differentiate and migrate to replenish the epithelium. Structural integrity and clarity are provided by a thick stromal layer (about 90 percent of the cornea). Stromal keratocytes deposit and maintain the extracellular matrix that provides corneal structure and transparency. The corneal endothelium (CenC) is a monolayer that unlike the epithelium does not divide. This layer separates the cornea from the aqueous humor with extensive tight junctions to maintain barrier functions to pump fluids from the stroma in order to maintain proper hydration and corneal clarity.

The molecular bases underlying these highly distinct physiologic functions have not been fully elucidated by previous technology platforms, including proteomics, microarrays, and bulk RNAseq^1–8^. We aimed to generate a single-cell RNAseq transcriptome of the human cornea to characterize the functions of each cell type at the whole genome transcriptomic level and gain insights into the corneal cell organization using RNAscope. Single-cell RNAseq offers the ability to investigate both prominent and rare cell populations within the same complex tissue and has been successfully utilized for studying ocular cell subtypes^9–13^. Here we present a single-cell RNAseq transcriptomic map from six healthy adult cornea samples. Clustering analysis revealed 16 unique cell clusters and captures all major cellular regions of the cornea.

This transcriptome map of the healthy human cornea could be utilized to better understand the cell populations affected by corneal dystrophy mutations and inform potential cellular and gene therapy approaches. Furthermore, transcriptomic information can provide functional insight into mechanisms of related polygenic corneal disorders and those associated with complex systemic or other organ diseases.

## Results

### Transcriptomic map of the human cornea

**Figure 1:**
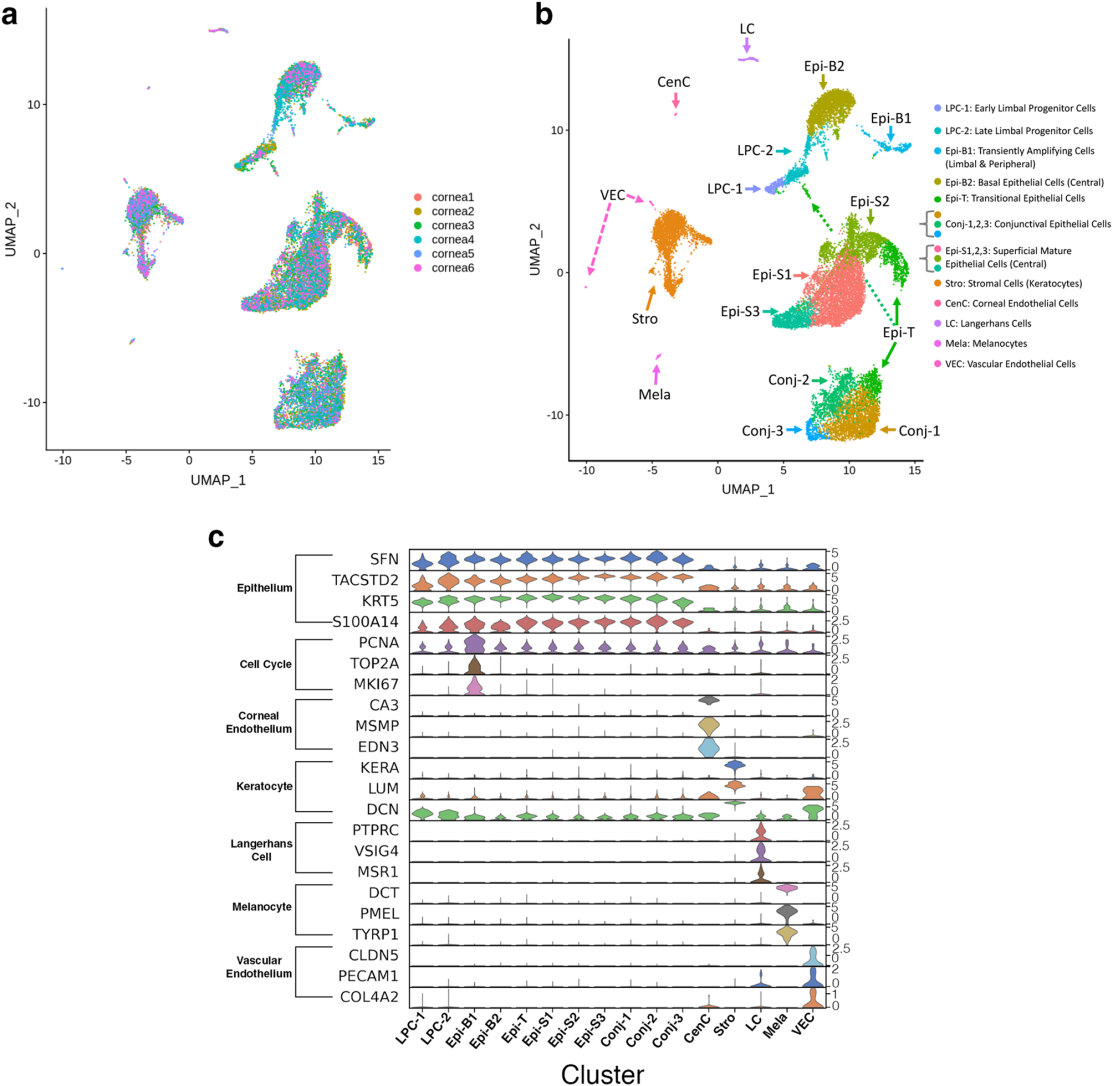
Sixteen clusters of cells were identified in the healthy adult human cornea. **(a)** UMAP visualization of the contribution of each of the 6 cornea samples. **(b)** UMAP visualization of the 16 clusters. Individual points correspond to single cells colored according to clusters identified. **(c)** Violin plot of marker gene expression in each cluster. Cell type related marker genes are listed on the left and cell types identified based on marker gene expression are labeled at the bottom. Clusters Conj-1–3 are conjunctival epithelial cells, LPC-1, 2, Epi-B1, B2, T, S1-3 are corneal epithelial cells, Stro are keratocytes, LC are Langerhans cells, Mela are melanocytes, VEC are vascular endothelial cells, and CenC are corneal endothelial cells. SCANPY 1.7.1 in Python was used to generate the violin plots as indicted in the "Materials and methods" (https://pypi.org/project/scanpy/).

One healthy cornea from each of the six donors (2M, 4F; median age 57) were analyzed using single-cell RNAseq techniques. Using criteria described in the Materials and methods (10X Genomics V2 chemistry), the number of cells which passed QC ranged 1575–4271 from each sample and resulted in a combined total of 16,234 cells for further analysis (Table 1). Uniform Maniford Approximation and Projection (UMAP) demonstrates a relatively even contribution of cells from each individual sample (Fig. 1a). Unsupervised clustering algorithm found 16 transcriptionally distinctive cell clusters, with some clusters residing in close groups, for instance, superficial corneal epithelium (Epi-S1,2,3) or conjunctival epithelium (Conj-1,2,3), which suggests that these could be highly similar, while other clusters, for instance stroma (Stro) are highly distinctive (Fig. 1b). These clusters were given these names because of their distinctive anatomical cell type-specific marker gene expression patterns, as discussed below. Cluster identities were assigned by comparing gene expression of cells in each cluster to that of cells in all the other clusters combined (Fig. 1c; heatmap: Fig. S1), and the presence of well-known cell type-specific marker genes. These 16 clusters correspond to 11 subtypes of epithelial cells, keratocytes, Langerhans cells, melanocytes, vascular endothelial cells and corneal endothelial cells (CenC) (Fig. 1b,c).

**Table 1:** Cell numbers contributing to each cell cluster from each donor cornea.

Cell cluster	LPC-1	LPC-2	Epi-B1	Epi-B2	Epi-T	Epi-S1	Epi-S2	Epi-S3	Conj-1	Conj-2	Conj-3	CenC	Stro	LC	Mela	VEC
Cornea 1	1	17	42	208	254	459	237	130	374	173	55	6	291	3	0	0
Cornea 2	142	225	50	350	316	1044	370	238	528	130	53	2	404	22	24	6
Cornea 3	21	46	64	266	203	487	269	75	353	255	52	7	694	18	1	0
Cornea 4	79	183	109	914	192	1115	515	270	501	146	66	7	120	33	14	7
Cornea 5	5	44	7	19	80	41	68	16	187	208	29	3	657	25	10	14
Cornea 6	10	28	10	224	61	464	131	145	88	52	23	3	299	29	3	5
Sum	258	543	282	1981	1106	3620	1590	874	2031	964	278	28	2465	130	52	32
Percent	1.6	3.3	1.7	12.2	6.8	22.3	9.8	5.4	12.5	5.9	1.7	0.2	15.2	0.8	0.3	0.2

### Molecular characteristics of cornea endothelial cells (CenCs)

Previous studies aiming to characterize human CenCs were either based on low throughput analysis of a subset of genes or genome-wide transcriptomic analysis of cultured and expanded CenCs which may not always fully reflect primary tissues^14–18^. Single-cell RNAseq of primary tissues better ensured CenC purity and provides a comprehensive genome-wide transcriptomic profile which cannot be achieved with bulk RNAseq approaches^3,19^.

**Figure 2:**
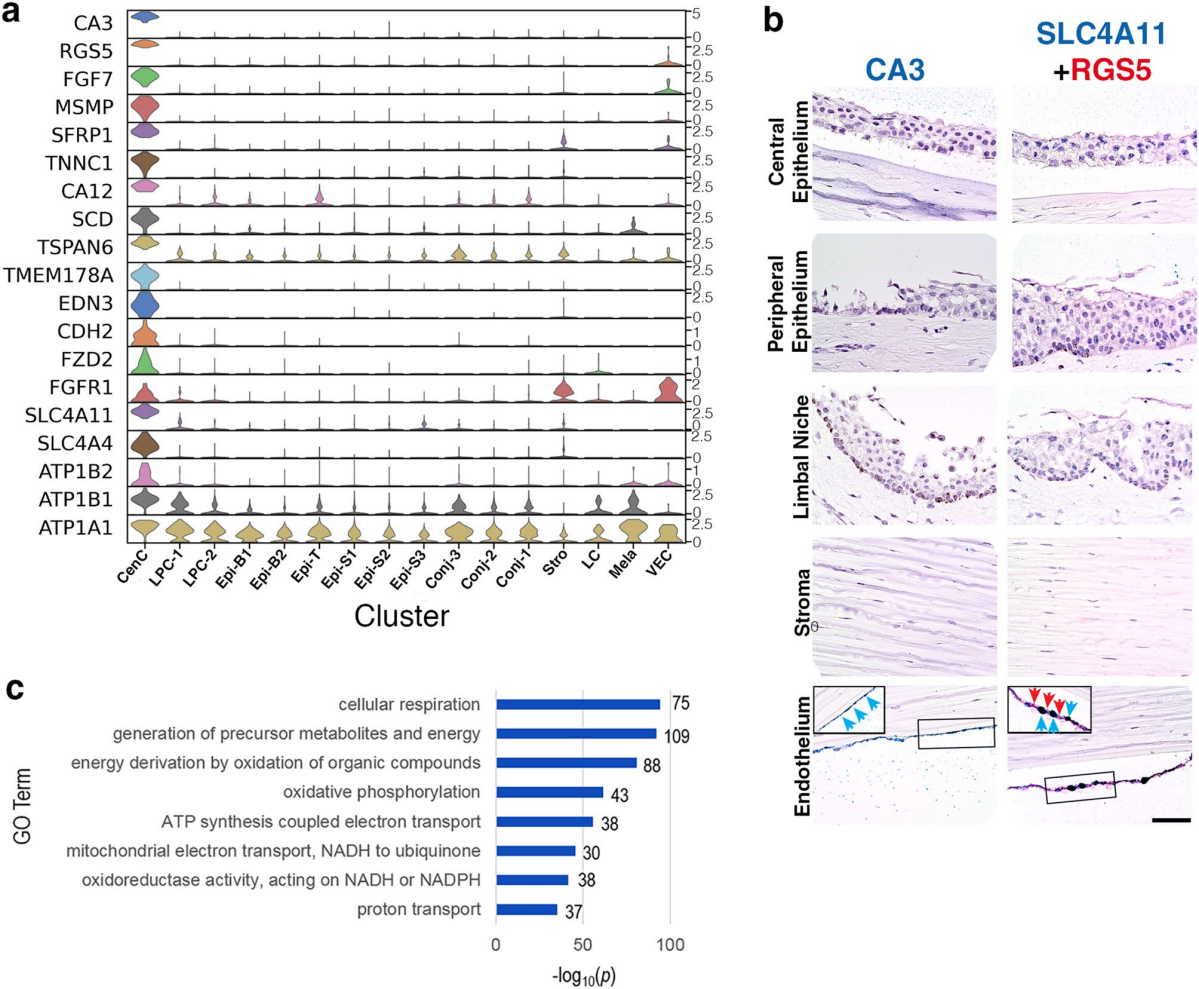
Molecular characteristics of corneal endothelial cells (CenC). **(a)** Violin plots of cluster marker gene expression. SCANPY 1.7.1 in Python was used to generate the violin plots as indicted in the "Materials and methods" (https://pypi.org/project/scanpy/). **(b)** RNAScope on human cornea of CenC specific genes *CA3*, *SLC4A11*, and *RGS5* (scale bar: 50 uM). Insert of zoomed in selection of endothelium is outlined for *SLC4A11* and *RGS5*. **(c)** Topmost enriched GO terms (Biological Process) of genes up-regulated in CenC compared to other clusters. The number of overlap genes are indicated next to each bar.

Besides those shown in Fig. 1c, Fig. 2a includes an expanded set of CenC specific or enriched marker genes (heatmap: Fig. S2; full gene list: Table S1) RNAscope results indicated that the expression of *CA3*, *SLC4A11* and *RGS5* were restricted to the CenC monolayer confirming their identity (Fig. 2b). Previously, *CA3* and *SCL4A11* have been reported to be highly expressed in CenCs^3,15,19^. We also find functionally related family members, *CA12* and *SLC4A4,* to be highly enriched in CenCs (Fig. 2a). *CA3* and *CA12* belong to a family of carbonic anhydrase (CA) proteins involved in fluid transport, a major CenC biological function.

Among the CenC marker genes in Fig. 2a, *MSMP* and *EDN3* are highly CenC enriched. *TMEM178A* is reported as a quality assessment marker for CenC cultures^16^. Both WNT and FGF pathways are manipulated during in vitro CenC differentiation and expansion for potentially engineering CenC grafts^20^. We find *FZD2*, a component in WNT signaling pathway, and *FGF7* and *FGFR1,* components of FGF signaling pathway, are enriched in CenCs (Fig. 2a). Pathway analysis suggests a higher expression of genes involved in cellular respiration in CenCs compared to other cornea cells: *SLC4A11*, *SLC4A4*, and N + /K + ATPases *ATP1A1*, *ATP1B1*, *ATP1B2* (Fig. 2c). This finding aligns with the key CenC function of actively transporting fluid requiring high metabolic activity.

*RGS5* has been considered as a marker of pericytes which are associated with vascular endothelial cells. In the cornea we find *RGS5* highly enriched in CenCs (Fig. 2a,b). The negative expression of another pericyte marker *PDGFRB* ruled out the possibility that this cluster was a mixture of CenCs and pericytes and supports *RGS5* as another CenC marker (Table S2).

### Molecular signature underlying key cornea stromal functions

**Figure 3:**
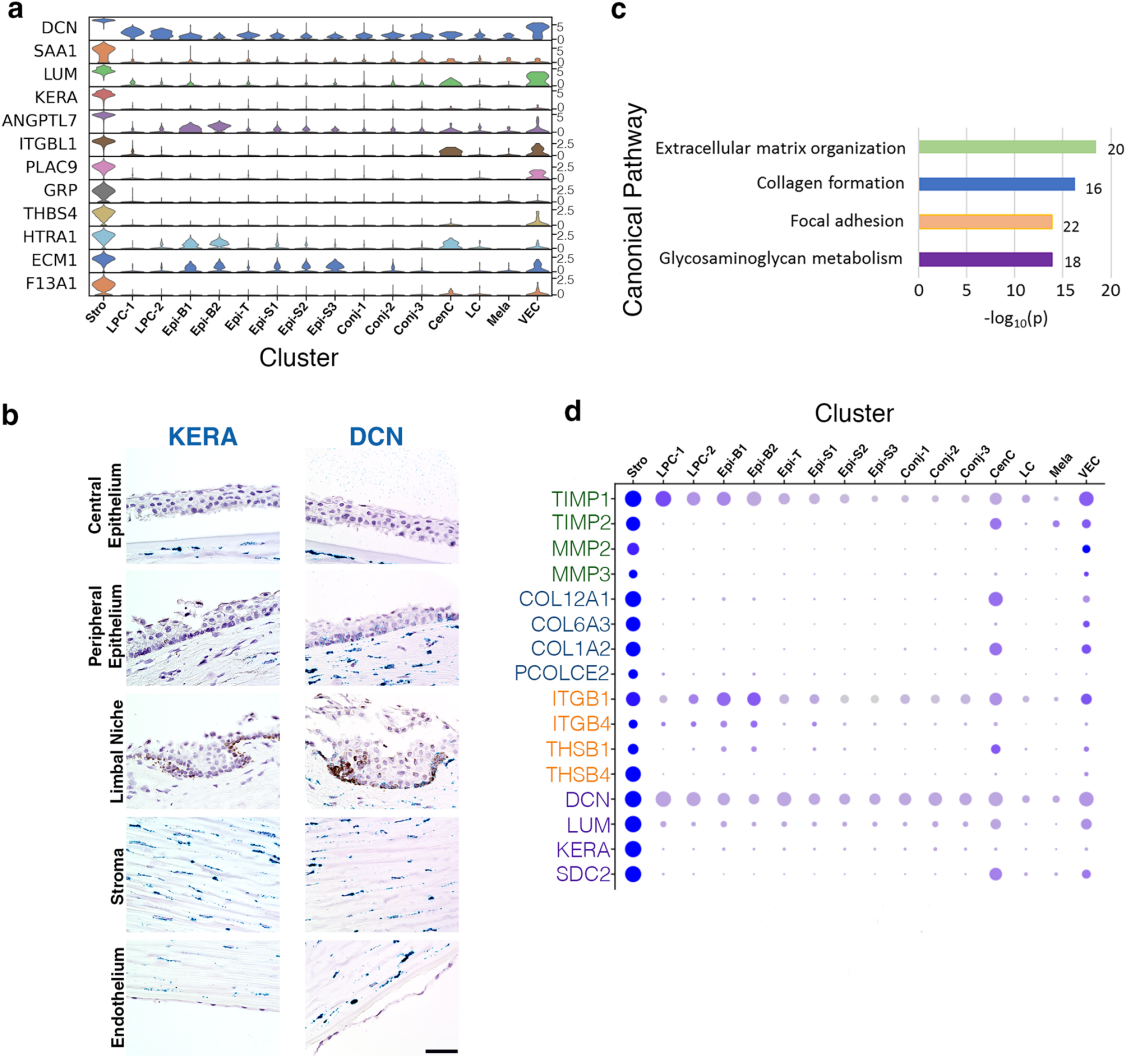
Molecular characteristics of corneal keratocytes. **(a)** Violin plots of stroma (Stro) marker gene expression. SCANPY 1.7.1 in Python was used to generate the violin plots as indicted in the “Materials and methods” (https://pypi.org/project/scanpy/). **(b)** RNAScope on human cornea of stroma keratocyte specific and enriched gene *KERA* and *DCN* (scale bar: 50 uM). **(c)** Topmost enriched MSigDB Canonical Pathways of genes up-regulated in Stro compared to other clusters. The number of overlap genes are indicated next to each bar. **(d)** Expression of representative pathway overlap genes identified in **(c)** with the circle diameter representing number of cells expressing the gene, and color saturation reflecting the level of expression. Color coordination represents the genes in (**d)** by their canonical pathway in **(c)**.

The cornea stroma is a thick layer primarily comprised of highly ordered layering of collagens that provide structure and strength to the cornea while also permitting passage of light. The dominant cell type in the stroma is the keratocyte. The stroma cluster (Stro) markers include classical keratocyte markers: *KERA, LUM, DCN* (Fig. 3a; heatmap: Fig. S3). Mutations in these genes are associated with stromal corneal dystrophies. As evidenced by knock-out mice with abnormal corneas, *KERA* is critical in maintaining proper corneal shape^21^ and *LUM* is required for ordered stromal collagen fibral spacing for corneal transparency^22^. Both *DCN* and *KERA* RNAscope results demonstrate staining throughout the stroma resembling the expected locations of keratocytes (Fig. 3b). *DCN* transcript was also found lightly staining basal cells in the limbus and peripheral cornea (Fig. 3b), which agrees with our observation that the expression of these genes is highly enriched in but not unique to the keratocytes (Fig. 3a). Similarly, in human skin *DCN* is found both in keratocytes and suprabasal epidermis layer^23^.

Stro marker genes were analyzed for pathway enrichment against MSigDB Canonical Pathways database (Fig. 3c,d). Collagen related genes *COL12A1*, *COL6A3*, *COL1A2* and *PCOLCE2* are expressed at higher levels in keratocytes than in other cell types, reflecting the collagen-rich composition of the stroma. *MMP2* and *MMP3* are metalloproteinases that break down extracellular matrix proteins; while *TIMP1* and *TIMP2* are metalloproteinase inhibitors. The high expression of these functionally opposing genes implies tight regulation of the corneal extracellular matrix. Key keratocyte markers *DCN, KERA, LUM* and *SDC2* are proteoglycans. Negatively charged glycosaminoglycans linked to these proteins can attract water and their interactions with collagens help to organize the extracellular matrix to maintain corneal transparency^24^. The high expression of focal adhesion molecules *ITGB1, ITGB4, THSB1* and *THSB4* ensures the interaction and communication between keratocytes and the extracellular environment.

### Mapping the location of subtypes of epithelial cells in human cornea

**Figure 4:**
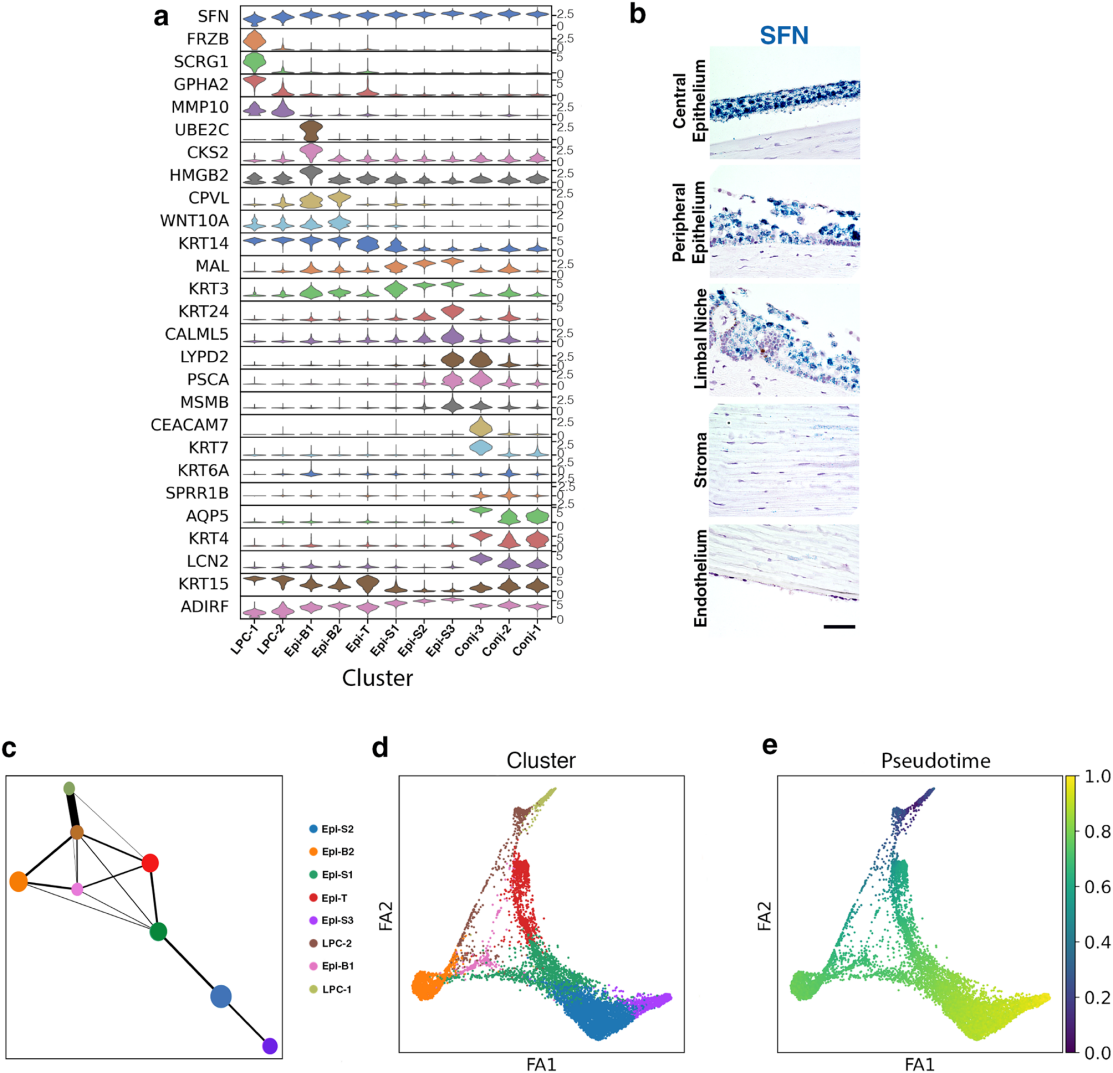
Molecular characteristics of epithelial cell clusters. **(a)** Violin plots of epithelial cell cluster (LPC-1, 2, Epi-B1, B2, T, S1-3, Conj-1-3) marker gene expression. **(b)** RNAScope on human cornea of pan-epithelial cell marker gene *SFN* (scale bar: 50uM). **(c)** Ball-and-stick representation of partition-based graph abstraction (PAGA) connectivity among corneal epithelial clusters with ball size reflecting cluster size and stick thickness representing the connectivity strength. **(d)** A PAGA-initialized force-directed single-cell embedding. Force atlas 1 (FA1) and 2 (FA2) are used for graphical presentation. Cells are colored by cluster identity. **(e)** PAGA pseudotime inferences using LPC-1 as the root for corneal epithelial cell clusters. SCANPY 1.7.1 in Python was used to generate violin plots and (**c**), (**d**) and (**e**) as indicted in “Materials and methods” (https://pypi.org/project/scanpy/).

The epithelial cell clusters were defined by high expression of known epithelial markers *SFN*, *KRT5*, *TACSTD2*, and *S100A14*; they represent the most diverse cell types with eleven clusters identified (Fig. 1c, Fig. 4a,b; heatmap: Fig. S4)^25–28^. Furthermore, three clusters represent conjunctival epithelial cells (Conj-1,2,3) and eight clusters represent corneal epithelial cells (LPC-1,2,Epi-B1,B2,T,S1,S2,S3). Analyses of epithelial sub-cluster marker genes revealed both uniquely expressed and shared genes among sub-sets of clusters (Fig. 4a). The observation of both distinctive and highly overlapping gene expression patterns is in agreement with some epithelial cells having distinct functions, while overlapping genes is consistent with the continuous epithelial structure from basal to superficial layer and from limbal to central cornea region^29^. Corneal epithelial cell development progresses from the limbus into the periphery and then the central region both laterally and vertically. PAGA and pseudotime analyses using LPC-1 as time 0 suggest that there are two major trajectories of corneal epithelial differentiation, one towards superficial cells (Epi-S1,2,3) and one towards basal cells (Epi-B1,2,T) (Fig. 4c–e).

#### Molecular signature of limbal progenitor cells (LPC), transiently amplifying cells, and other basal epithelial cells

One of the characteristics of cornea epithelial cells is the ability to continually self-renew. Previous bulk transcriptomics of peripheral cornea and limbal niche report upregulated genes for cell cycling and proliferation in the cornea which is reflective of maintaining epithelial turn over^6^. The limbal epithelial stem cells (LESCs) reside within the basal layer palisades of Vogt and have been challenging to characterize within the heterogeneous limbal niche.

**Figure 5:**
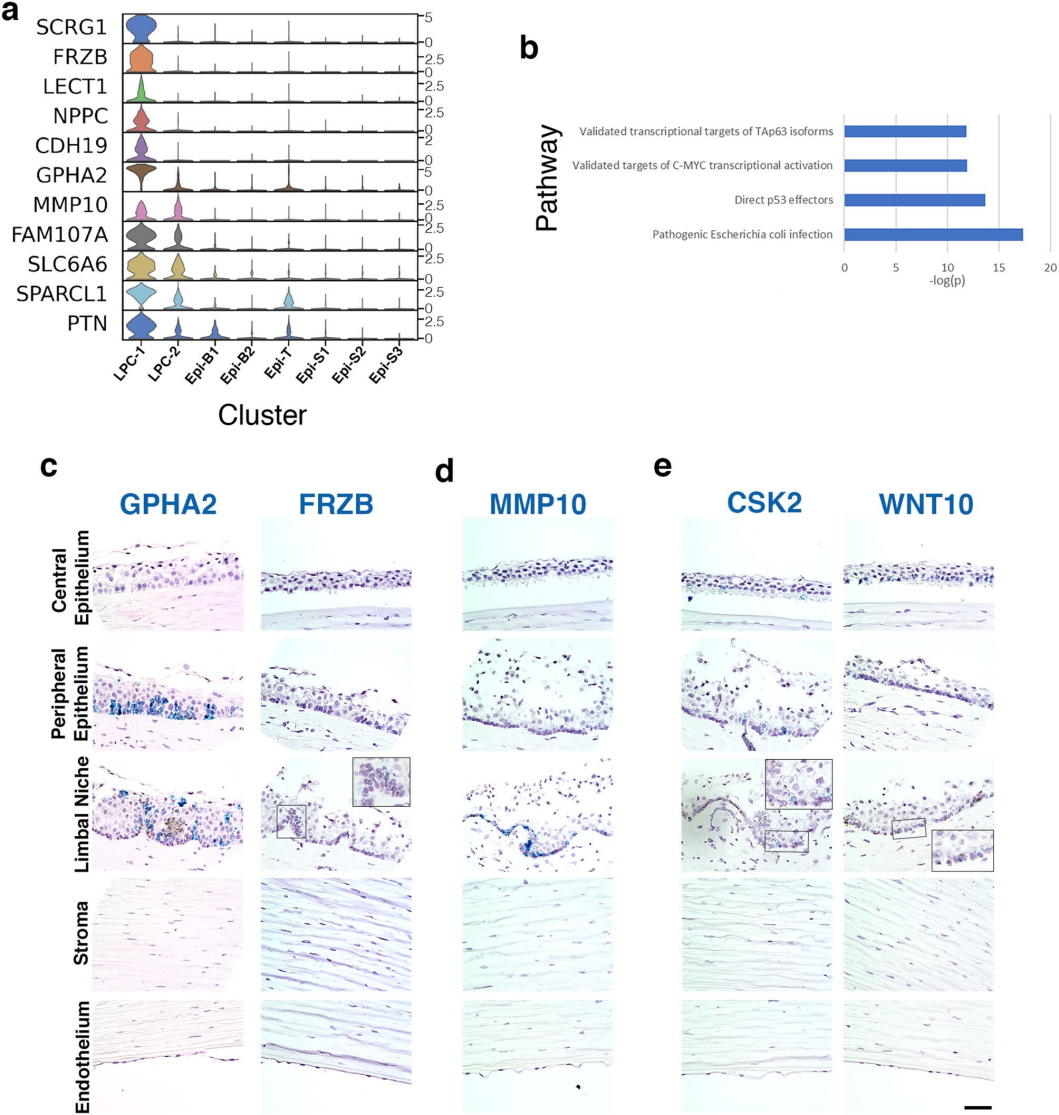
Renewal of corneal epithelial cells: Limbal early progenitor cells, late progenitor cells, basal, and transiently amplifying cells. **(a)** Violin plots of LPC-1 marker gene expression. SCANPY 1.7.1 in Python was used to generate violin plots as indicted in “Materials and methods” (https://pypi.org/project/scanpy/). (**b**) Topmost enriched MSigDB Canonical Pathways of genes differentially expressed between LPC-1 and 2. These top pathways correspond to genes down-regulated in LPC-1. The number of overlap genes are indicated next to each bar. RNAscope of selected markers from **(a)** on human cornea of **(c)** early limbal progenitor cells (LPC-1) *GPHA2* and *FRZB*, **(d)** early and late limbal progenitor cells (LPC-1 and 2) *MMP10*, and **(e)** mixed basal or transiently amplifying cells (Epi-B1,B2) *CSK2* and *WNT10A* (scale bar: 50 uM).

We identified marker genes for LPC-1 (Table S1), with representative genes presented in Fig. 5a. We hypothesize that LPC-1 are early limbal progenitor cells stemming from LESC differentiation due to their enriched expression of genes expected of LESC and other stem cells from previous reports: *SCRG1, FRZB,* and *GPHA2* (Fig. 5a). Additionally, *LECT1, NPPC*, and *CDH19* are also highly enriched in LPC-1 compared to the remaining epithelial cell clusters (Fig. 5a, heatmap: Fig. S5). *GPHA2* is highly enriched in LPC-1, but also expressed in LPC-2 and Epi-B1 (Fig. 5a) with RNAscope demonstrating expression in basal cells within the limbus and the limbal-adjacent peripheral cornea, with no staining in the basal epithelium of the central cornea (Fig. 5c). We find *FRZB* predominately expressed in the limbus and absent from the central cornea (Fig. 5c). RNAscope suggests that LPC-1 enriched genes can also be expressed in a few clusters which reside in the basal epithelium of the peripheral cornea. Therefore, LPC-1 was chosen as the origin point of the other corneal epithelial cell clusters in our PAGA and pseudotime analyses (Fig. 4c–e).

LPC-2 is most similar in gene expression to LPC-1 (Fig. 4a,c,d) and may be the next transitional state of late limbal progenitor cells. For instance, *MMP10* is found diffusely staining in the basal cell layer of the limbal niche and is enriched in both LPC-1 and 2 (Fig. 5a,d). To better capture the unique signaling pathways defining LPC-1 and LPC-2, we performed pathway analysis on differentially expressed genes. The top-most enriched signaling pathways included p53, p63 and c-Myc (Fig. 5b). These pathways are important for cell growth, differentiation and apoptosis and were down-regulated in early progenitors (LPC-1) as compared to late progenitors (LPC-2) (Fig. 5b).

Stem and early progenitor cells in healthy corneas infrequently divide whereas transiently amplifying cells are often dividing and can be identified by proliferation markers. Epi-B1 have distinctive expression of cell cycle-related genes, such as *PCNA, TOP2A, MKI67* and *UBE2C* (Fig. 1c). *CKS2*, is highly enriched in Epi-B2 (Fig. 4a) with expression predominately located along clusters of basal limbal and peripheral cornea cells and minimal staining in central basal cell clusters (Fig. 5e). Epi-B2 represents a trajectory stemming from related limbal and basal cell clusters (Fig. 4c–e). *WNT10A* is expressed in LPC-1,2,Epi-B1,B2 and is most enriched in Epi-B2 (Fig. 4a). RNAscope for *WNT10A* demonstrates staining in basal cells beginning within the limbal region, continuing into the periphery, and extending into the central cornea (Fig. 5e). *WNT10A* is reported to be upregulated in central cornea compared to the limbal niche^30^. Therefore, Epi-B1 are likely transiently amplifying cells which move from the limbus towards central cornea contributing to basal epithelial cells along with Epi-B2.

#### Molecular characteristics of conjunctival and corneal epithelial cells

**Figure 6:**
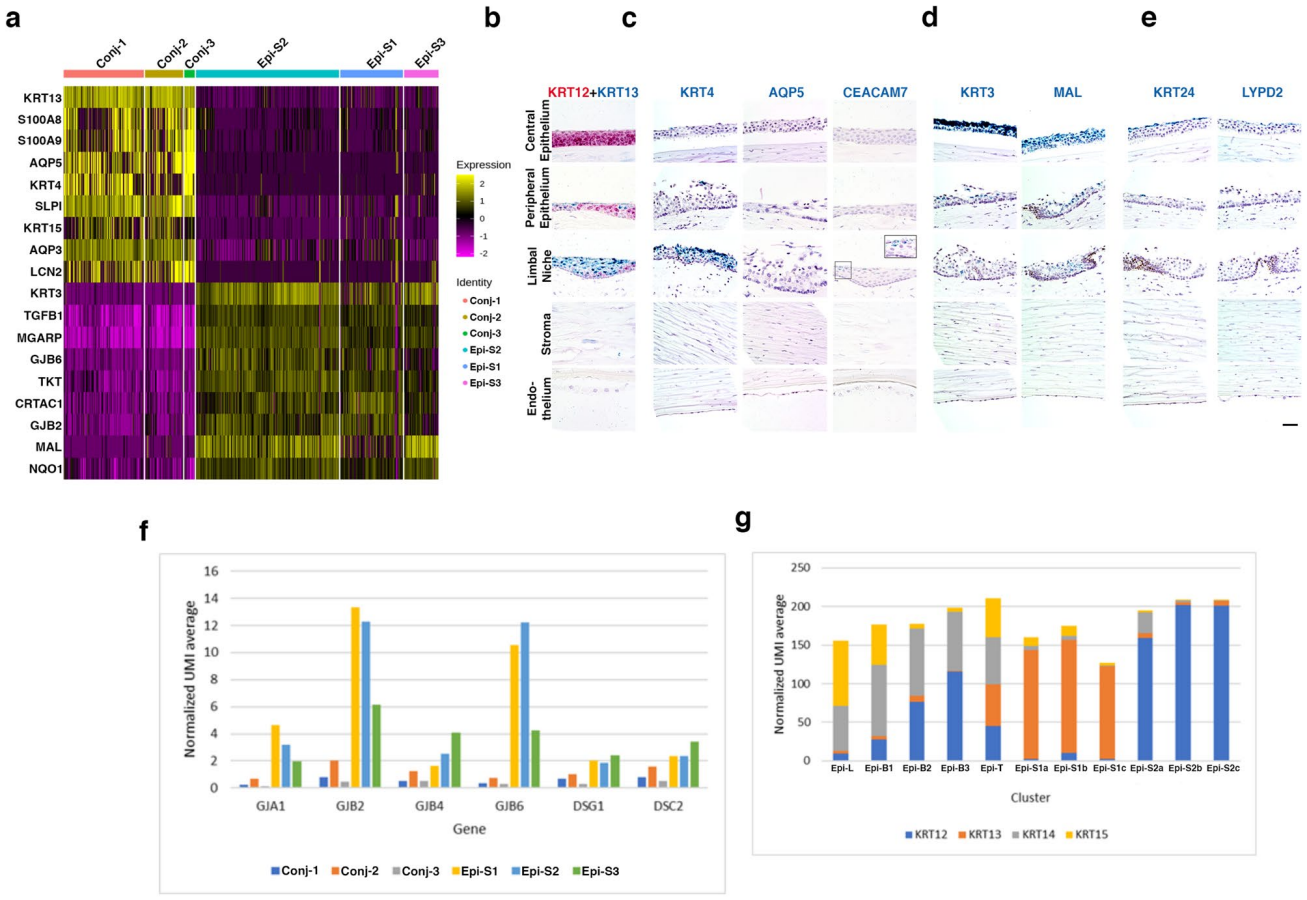
Molecular characteristics of conjunctival and corneal epithelial cells. **(a)** Heatmap of the top-most significantly differentially expressed genes between conjunctiva (Conj-1-3) and cornea (Epi-S1-3) that also overlap with cornea and conjunctiva comparison (from Fig. S6). RNAscope of selected markers from Fig. 4a. on human cornea of **(b)** *KRT12* (red) as mature cornea epithelium marker and *KRT13* (green) as conjunctiva marker, **(c)** conjunctival epithelial cell group Conj-1-3 genes *KRT4, AQP5*, and *CEACAM7* **(d)** superficial mature corneal epithelial cell group Epi-S1-3 genes *KRT3* and *MAL*, **(e)** cluster Conj-3 and Epi-S3 epithelial cell genes *KRT24* and *LYPD2* (scale bar: 50 uM)*.* **(f)** Significant differentially expressed genes encoding cell junction proteins between groups conjunctival (Conj-1-3) and corneal (Epi-S1-3) epithelium. **(g)** Expression patterns of keratins *KRT12*, *KRT13*, *KRT14*, and *KRT15* in the conjunctival and corneal epithelial cell clusters.

Genes differentially expressed between Epi-S1,2,3 and Conj-1,2,3 were identified and subjected to NextBio correlation analysis which identified a cornea and conjunctiva comparison in the database (Fig. S6)^31^. The top overlap genes distinguishing the two types of epithelial are demonstrated in a heatmap (Fig. 6a). *KRT13* and *KRT12* staining was used to confirm the locations of the bulbar conjunctival epithelium and the start of the superficial mature corneal epithelium respectively, as previously reported^31,32^. *KRT13* is enriched in the conjunctiva and extends over the superficial layer of the limbal region whereas *KRT12* is enriched in the mature cornea and is seen from the peripheral and extending throughout the central cornea (Figs. 4a and 6b).

The bulbar conjunctival epithelium (Conj-1,2,3) extends into the corneal region and resides superficially within the limbal region and peripheral-most cornea (Figs. 4a, 6b,c). *KRT4* and *AQP5* are enriched within the superficial epithelial layer of the limbal region with a few cells found within the peripheral cornea (Fig. 6c). *CEACAM7* is highly enriched in the Conj-3 cluster compared to Conj-1,2 and marks cells on the further edge of the superficial bulbar conjunctiva above the limbus (Fig. 6c). These conjunctival markers are used to distinguish from adjacent corneal epithelial cells^31,33^.

Epi-S1,2,3 represent the central mature superficial corneal epithelium and comprise a developmental branch in the corneal epithelial trajectory (Figs. 4a, 6b,d). RNAscope of enriched marker gene *KRT3* demonstrates strong staining within the superficial layers of the central cornea with sparse staining in the limbus and peripheral cornea, and no staining in basal cells or palisades of Vogt (Fig. 6d). *KRT3* expression is associated with the differentiation of transient amplifying cells into mature superficial epithelium and is absent from basal and limbal epithelium^34–37^. *MAL* staining was also enriched in the superficial cells of the central cornea, with more positive cells in the limbus and peripheral cornea than *KRT3* (Fig. 6c). MAL plays a role in lipid-raft-mediated apical sorting of epithelium proteins^38^.

RNAscope staining of *LYPD2* (Epi-S3, Conj-3) and *KRT24* (Epi-S3) demonstrate that these are expressed within the superficial-most epithelial layers (Fig. 6e). *KRT24* has a few positive cells in the superficial layer of the peripheral cornea, but no staining in the limbus (Fig. 6e) which agrees with a report of high expression in superficial cornea epithelium^37^ rather than conjunctiva.

Finally, Epi-T cells are defined by the lack of unique marker genes and sharing the expression of marker genes of many other clusters, suggestive of a transitional epithelial state (Fig. 4a,c).

To further characterize the epithelial clusters, we examined the expression of genes key to epithelial functions. Multiple cellular junction genes were found to be expressed at lower levels in Conj-1,2,3 conjunctival than in Epi-S1,2,3 corneal epithelium (Fig. 6f). Several connexins (*GJA2*, *GJB2, GJB4, GJB6)* were significantly differentially expressed which may be important in coordinating cell-to-cell communication and signals among epithelial cells^39^. Desmosome components, *DSG1* and *DSC2,* which provide structural strength to adhere neighboring cells together, are enriched in the corneal epithelial layer (Fig. 6f). *DSG1* is known to be enriched in the skin’s superficial epithelial layer and we demonstrate a similar pattern in the cornea (Fig. 6f). This reflects the more prominent function of maintaining a tight physical barrier and cell–cell adhesion in the corneal as compared to the conjunctival epithelium.

A wide range of keratins have been reported to be highly expressed in cornea and contribute to the global corneal signature in comparison to other ocular regions^2,37^ and are the most abundant proteins expressed by corneal epithelial cells^4^. Keratins play important roles in maintaining corneal transparency by organizing extracellular collagen proteins and retaining water in the cornea through negatively charged glycosaminoglycans. Keratin expression patterns are also utilized to identify epithelial differentiation states, as well as residing in the conjunctiva or cornea. We examined the expression patterns of the top four keratins across epithelial subtypes, and similar to the gene expression profiles outlined above, the relative expression of keratins also defined related clusters based on anatomical location and biological functions (Fig. 6g). *KRT13* is enriched in the conjunctiva (Conj-1–3) while *KRT12* dominates the mature corneal epithelial cells (Epi-S1-3) (Fig. 6b,g). *KRT14* and *KRT15* are expressed predominately in clusters residing in the basal epithelial layer across all cornea regions (LPC-1,2,Epi-B1,B2,T) (Fig. 6g). Transitional Epi-T has homogenous expression of all four keratins (Fig. 6g). Together with the physical locations of epithelial sub-clusters we obtained from RNAScope, our observation provides a spatial location of keratin expression patterns.

### Other cell types

Among the 16 clusters, three represented supportive cells known to reside in the cornea. Langerhans cells (LC) are characterized by expression of *MSR1, VSIG4,* and *PTPRC* (Fig. 1c) which collectively identify these as resident dendritic cells. Melanocytes (Mela) are characterized by classical markers including *TYRP1, DCT,* and *PMEL* (Fig. 1c). Vascular endothelial cells (VEC) expresses characteristic markers *CLDN5, PECAM1,* and *COL4A2* (Fig. 1c), presumably representing the limbal vasculature*.*

### Corneal dystrophies

**Figure 7:**
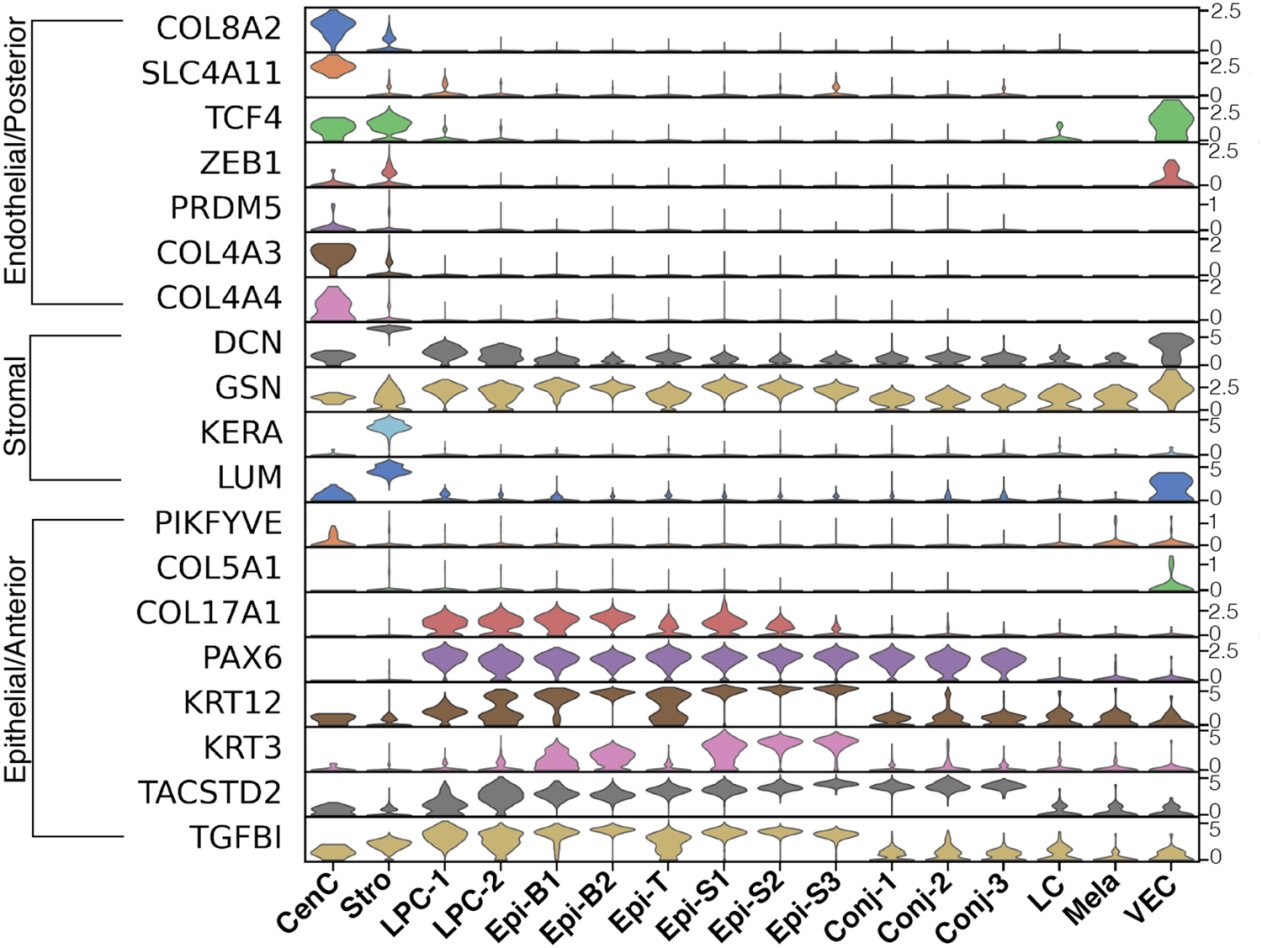
Violin plot with expression of corneal disease associated genes across all cell clusters. Predominant corneal regions affected by mutations are denoted on the left. SCANPY 1.7.1 in Python was used to generate violin plots as indicted in “Materials and methods” (https://pypi.org/project/scanpy/).

Each of the three major cellular regions can be affected by corneal dystrophies^40–42^. The majority of monogenic diseases display pathology in a single layer and require corneal grafts for treatment. Reported causal genes for monogenic corneal dystrophies and their expression patterns among the clusters is shown in Fig. 7 (heatmap: Fig. S7). As expected, the causal genes are predominately enriched in clusters where the pathology occurs.

There is a category of seven corneal dystrophies, epithelial-stromal TGFBI dystrophies, that are all caused by 70 reported mutations in *TGFBI*^42–44^. *TGFBI* (kerato-epithelin) is an extracellular protein enriched in the epithelium. It is a critical protein for maintaining structure in the corneal epithelial and stromal layers through its cell adhesion and cell-collagen interactions. Expression of *TGFBI* is widespread among the clusters which corresponds to the wide range of mutation-associated pathologies (Fig. 7).

## Discussion

A strength of this study is that it provides a deep single-cell-based transcriptomic-based identification of 16 cell clusters from six healthy adult human corneas and adjacent bulbar conjunctiva. We were able to generate whole-genome transcriptomic profile for each cell type identified and map out relationships between epithelial cell clusters. We are confident in our assignment of the small but important LPC-1 and CenC cells as early limbal progenitor and corneal endothelial cells, but the paucity of them in our population prevented further in-depth characterization of their transcriptome. Early LPC cells are challenging to distinguish from LESC, due to the absence of exclusive markers or sufficient cell numbers, making it difficult to assert LESC with great confidence. Future studies including additional healthy corneas to collect larger cell numbers for each cluster would be advantageous to increase the chances of detecting low-expression genes particularly in rare cell types. A recent report of single-cell RNAseq which included four healthy adult human corneas also revealed a diverse set of unique clusters from the cornea and conjunctiva, identifying similar key genes and cell types^45^. Collectively, these two studies strengthen the transcriptomic map foundation of the healthy adult human cornea.

**Figure 8:**
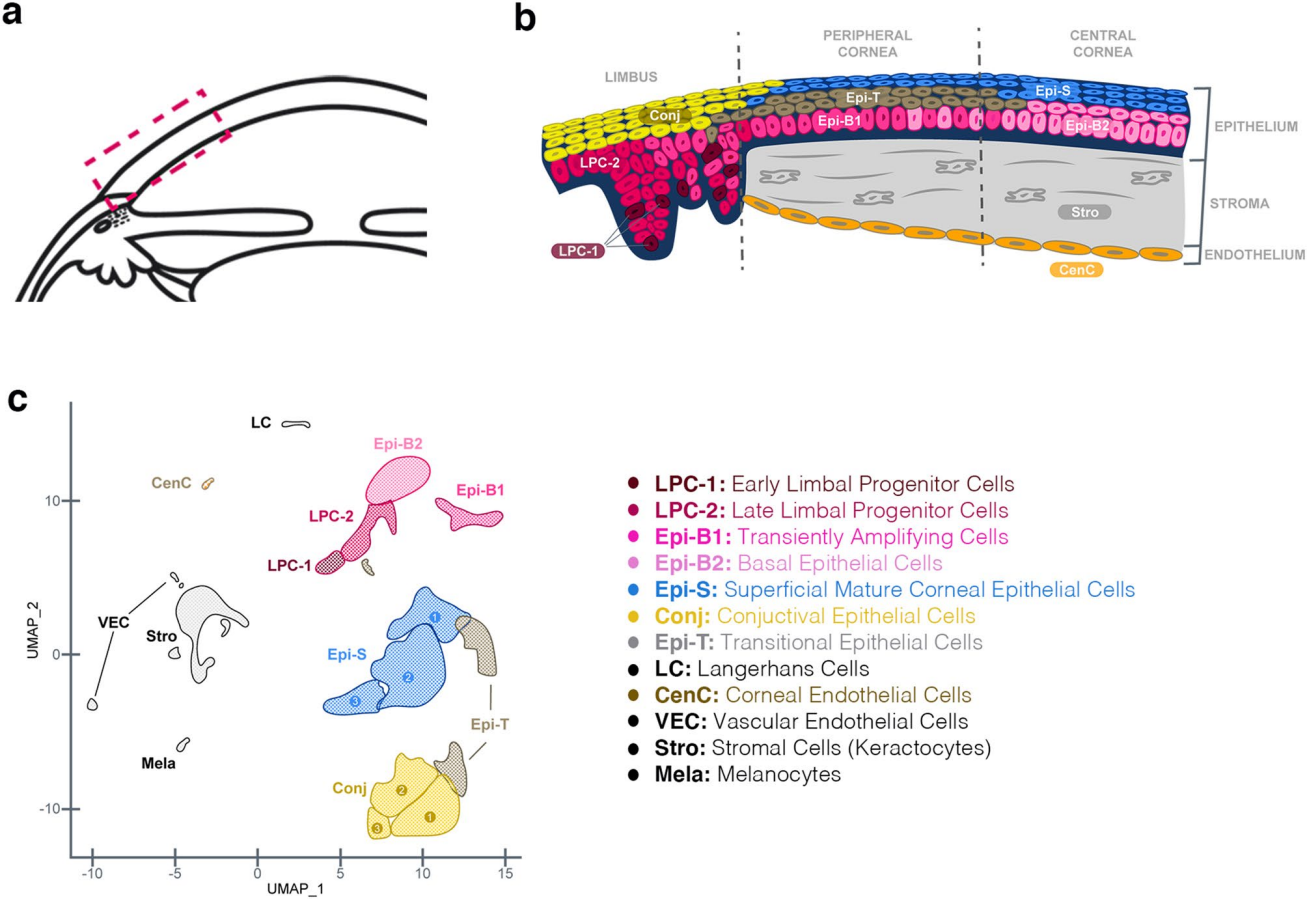
Graphical summary of cellular cluster locations in human cornea. **(a)** Diagram of cross-section of anterior segment of human eye with the box highlighting the area show in **(b)**. **(b)** Summary of the s.c. RNAseq clusters locations along the corneal cross-section with colors and cell types represented in the **(c)** color-coded uMAP plot and **(b)** cell type legend.

The location of the clusters in human cornea based on gene expression profiles and as shown by RNAscope is graphically summarized in Fig. 8. This single-cell transcriptomic map offers further detailed insight into the complexity of the human cornea which is best reflected in the wide heterogeneity of classified epithelial cells. There are two major superficial/suprabasal epithelial groups, the conjunctival (Conj-1–3) and the corneal (Epi-S1-3) epithelium. The peripheral cornea is a transition zone of dividing and migrating cells which is reflected by the gradient of cell types and genes shared among the peripheral cornea clusters. We propose a trajectory of epithelial cells originating from the early LPC (LPC-1), transitioning to late LPC (LPC-2) and transiently amplifying cells (Epi-B1) and then to transitional cells (Epi-T) and centrally enriched basal epithelial cells (Epi-B2), and ultimately to the mature superficial corneal epithelium (Epi-S1-3) (Figs. 4c,d, 8b). These cellular transitions represent a complex development and migratory process underlying the remodeling of the epithelial cells.

We aimed to obtain whole-genome transcriptomic profile of primary unmanipulated CenCs. Despite only obtaining 28 CenCs, the identification is clear based on the unique transcriptome. Since the CenC is a monolayer, this population is considerably smaller than the epithelial or stroma populations. Due to the small cell number and low detection sensitivity of scRNASeq technology, we most likely failed to detect many low-level expression genes in CenCs. Nevertheless, the detection of 11,706 genes provided a better insight of primary CenC biology than what was previously available. For instance, manipulation WNT or FGF pathways was utilized in CenC culturing due to high gene expression of family members^20^. Our CenC signature provides molecular characteristics that can be used for monitoring the quality of cultured and expanded CenCs for potential cellular therapy.

The LESC are essential for maintaining cornea epithelial homeostasis and wound healing. Wnt signaling is essential for LESC proliferation and maintenance^46,47^ and we found expression of members in LPC and basal layer cells. Limbal epithelial stem cell deficiencies (LSCD) result in abnormal epithelial wound healing, neovascularization, and vision loss^48^. Limbal transplants offer transient restoration due to allograft failure at a higher rate than central corneal transplants^49^. Due to the heterogenous nature of the limbal region and diverse types of cells present, it has been challenging to identify resting primary LESC and LPC markers. Numerous genes have been suggested to be LESC/LPC markers, but many can also be detected in other corneal cells^6,7,34,50–53^. The enrichment of a gene in the limbal niche does not indicate that it is exclusive to LESC or surrounding cells. We propose that the enriched genes comprising the signature of LPC-1 indicates they are the earliest progenitor cells arising from LESC differentiation. Expression of *CDH19*, *GPHA2*, and *FRZB* have been reported in several gene profiling studies of human LESC or limbal niche^6,7,52,54^. Interestingly, a recent study independently also identified *GPHA2* as a novel putative LPC marker using scRNAseq and found its expression to be lost as LPC cells are expanded *in vitro*^45^. *FRZB* has been found enriched in the palisades of Vogt with staining in spots along basal peripheral, but not central, cornea^6,30^. Additionally, we find *SCRG1*, *LECT1, NPPC*, and *CDH19* to also be highly enriched in LPC-1 and these genes are reported in other stem and early-progenitor cell populations of the bone and peripheral nervous system^55–59^. A more comprehensive understanding of unique genes in the LPC, limbal stem cell niche, and transient amplifying cells may offer insight into the underlying pathological origin of corneal diseases and extended damage from injury.

Apart from identifying unique markers for the regions of the epithelium, analysis of differential expression of gene families reveals patterns associated with conjunctival and corneal epithelial regions. Connexins were highly expressed in the superficial corneal epithelium. These gap junction proteins permit cell-to-cell communication and are found in various epithelial tissues, have differential cell and tissue isoform distributions, and are involved with wound healing responses^39^. Mutations in *GJB2*, *GJB6*, and *GJA1* result in keratitis, corneal opacity, skin disorders, and hearing loss^60,61^. Multiple connexin isoforms are found with differential spatial expression in corneal epithelium and display altered patterns in diseased or injured corneas^62–64^. Desmosomes maintain strong attachments between cells and the weak corneal epithelial attachment in vitamin-D receptor knockout mice has been attributed to decreased expression of *DSG1* and *DSC2*^65^. We find enrichment of both gene families in corneal epithelium as compared to the conjunctival epithelium highlighting the critical need for tight regulation of cell connections in maintaining the cornea epithelial barrier function in an avascular environment.

The top four expressed keratins, *KRT12, KRT13, KRT14, KRT15,* showed differential expression patterns among the epithelial clusters (Fig. 6g). *KRT13* was enriched in the bulbar conjunctival epithelium and *KRT12* was enriched in the corneal epithelium, a pattern consistent with previous reports^31,32,66,67^. Meesmann dystrophy presents with unstable and fragile corneal epithelium, and is associated with *KRT3* or *KRT12* mutations, both of which are corneal epithelial differentiation markers and not highly-expressed in the basal or limbal epithelium^37,68^. *KRT14* and *KRT15* have previously been reported by several groups as potential LESC markers^26,52,53,69^, presumably due to expression in the basal layers as we find in this present study. *KRT14* is present in basal cells of stratified epithelium in skin and cornea^37^. Furthermore, we see that *KRT15* is absent from the central cornea clusters while *KRT14* is maintained to a small degree which corresponds to findings from the bovine and mouse cornea^70,71^.

A meta-analysis of whole cornea tissue transcriptome compared to other ocular tissues revealed enrichment for collagen metabolism and extracellular matrix organization^1^ which is not surprising due to the relatively large stromal contribution to the cornea thickness. Over 15% of cells in our analysis are keratocytes within a single cluster. Keratocytes are fibroblasts which are normally quiescent and are stimulated by injury to repair the cornea by transitioning to different phenotypes^72^. Keratoconus is a multi-factorial disease where keratocyte and stromal pathology are evident. Keratocyte apoptosis and stress-related dysfunctions have been associated with keratoconus and transcriptional alterations in many functional pathways are altered in keratoconus stromal cells^73–75^. We found *COL12A1* as the highest collagen gene expressed in keratocytes, which plays an important role in establishing and maintaining stromal structure and function^76^. We also find matrix metalloproteinases *MMP2* and *MMP3* and their inhibitors *TIMP1* and *TIMP2* highly enriched in keratocytes which contributes to their function in regulating extracellular matrix composition.

Many genes found in the various epithelial groups are also shared with the skin epithelium as well, suggesting shared mechanisms in the barrier structures. There were several key relevant genes with biological functions in wound healing that helped define cell clusters. In addition to the well-known and severe retinal complications in diabetic retinopathy and systemic wound healing deficiencies in diabetics, there is a wide range of frequent but underdiagnosed corneal abnormalities in diabetics^77,78^. One such example is that *MMP10* expression is elevated in diabetic retinopathy patients’ corneas and implicated in the abnormal wound repair response^79^. *Wnt10A* deficient mice have delayed skin wound healing and reduced synthesis of collagen^80^. Prominent markers of the conjunctiva epithelium are associated with wound healing responses. *S100A8* and *S100A9* are important host defense and wound healing proteins within the body, including epithelial layers^81,82^. Additionally, *AQP5* knock-out mice have reduced corneal wound healing^83^. The cornea and conjunctiva epithelium, like the skin, is in direct contact with the exterior world thus rapid and effective wound healing responses are critical for maintaining functional integrity. Treatments focused on key wound healing genes may facilitate reversal of corneal pathology.

Vascular endothelium, melanocytes, and Langerhans cells, while not common, are present in the cornea. These supportive cells are necessary for the healthy functioning of the limbal niche and subsequently the corneal epithelium.

Corneal dystrophies are typically progressive and those that result in visual loss are still predominately treated by corneal transplantation. Transplants can be complicated by disease recurrence, graft failures, the need for repeated transplant over the patient’s lifetime, and accessibility of graft tissue in some regions of the world^42,84^. Therefore, there is a clinical need to better understand these dystrophies from a genetic, cellular, and molecular level to advance novel gene-editing and cell-based therapies.

The aim of this study was to provide a reference single-cell transcriptomic and spatial map of the healthy adult human cornea. Identification of cellular subsets within the cornea by using transcriptional information can help inform future cellular studies. For example, with the success of iPSC differentiation into terminally differentiated cells for research or transplantation, there is a need for well-defined characterization markers to ensure the correct cell has been generated in vitro to match its intended in vivo counterpart. These results contribute to that effort.

## Materials and methods

### Human tissue

Corneas from deceased human donors were approved for research purposes with informed consent obtained by The Eye-Bank for Sight Restoration (New York City) and all identifiable information removed. Ethical approval for research use of the donor corneas is approved by The Eye-Bank for Sight Restoration Research Review Committee. The guidelines of removing all identifiable information and ethical handling of the donor corneas were set by The Eye-Bank for Sight Restoration Research Review Committee. These research corneas were used in accordance with guidelines and regulations outlined by The Eye-Bank for Sight Restoration Research Review Committee. The corneas were collected a median of 12 h postmortem and placed into Optisol medium and stored at 4C until processing. One cornea from each of the six donors (2 male, 4 female; median age 57) with no noted ocular or corneal disease history were processed for FACS and single-cell RNAseq using 10X Genomics v2 platform.

### Cornea tissue dissociation and cell sorting

Corneas were individually dissociated. Briefly, corneas were finely chopped in the storage Optisol media and transferred to DMEM containing a digestion mix of Collagenase A, Dispase II, and DNAse I. The samples were incubated at 37 °C for 15 min with 1–2 intervals of pipette mixing to aid in tissue disruption. Samples were centrifuged and the cell pellets were further digested in 0.5% Trypsin for 5 min at 37 °C. Hoeschst nuclear dye was added to each sample with a small portion of unstained sample reserved for FACS gating. Cells were washes in 5%PBS and 2 mM EDTA to stop digestion and pelleted. Topro3 viability dye was added to each sample. Viable cells were identified as Hoechst positive and Topro3 negative and sorted with the MoFlo Astrios (Beckman Coulter). Samples were collected and pelleted for 10X Genomics v2 preparation and processing.

### Single-cell RNA-seq

Cells counts were generated using the Nucleocounter NC-250 Chromium v2 platform (10X Genomics). Single cells suspended in PBS with 0.04% BSA were loaded on a Chromium Single Cell Instrument (10X Genomics). RNASeq libraries were prepared using Chromium Single Cell 3’ Library, Gel Beads & Multiplex Kit, v2 (10X Genomics). RNASeq libraries were prepared using Chromium. Paired-end sequencing was performed on Illumina NextSeq500 (Read 1 26-bp for unique molecular identifier (UMI) and cell barcode, 8-bp i7 sample index, 0-bp i5, and Read 2 55-bp transcript read). Cell Ranger Single-Cell Software Suite (10X Genomics, v2.0.0) was used to perform sample de-multiplexing, alignment, filtering, and UMI counting. The Human b37.3 Genome assembly and UCSC gene model for human were used for the alignment.

### Data quality assessment, normalization and integration

Data quality of each cell was assessed using three parameters: total UMI, number of genes detected and the ratio between mitochondria gene UMI and total UMI. To avoid cell doublets and possibly unhealthy cells due to the experiment manipulation, cells with number of genes detected over 5000 or mitochondrial vs. total UMI ratio over 0.2 were removed. Seurat 3.0.1 program^85,86^ using R (R Core Team, 2013) was then used to perform all further single cell transcriptome analyses for this project unless mentioned otherwise. Seurat program scaled the total UMI to 10,000 for each cell followed by UMI natural log transformation. Data integration across the 6 samples was performed using two Seurat functions: FindIntegrationAnchors (based on top 2000 variable genes identified by FindVariableFeatures) and IntegrateData. In both functions, 30 scaling dimensions of variables were used. Figure S8 outlines the cell filtering based on QC.

### Cell cluster generation and identification of cluster marker genes or genes differentially expressed between two cell populations

Once the data were integrated from the 6 samples, data were rescaled using Seurat function ScaleData. Principal component analysis was performed on the variables (genes) using RunPCA. Cell clusters were identified by invoking functions FindNeighbors (using 30 scaling dimensions of variables) and FindClusters (using resolution 0.6). Cluster IDs were given to cell clusters in an ascending order based on the number of cells in each cluster: the smallest cluster ID was given to the largest cell cluster.

Cluster marker genes were identified by FindAllMarkers of Seurat using three cutoff thresholds: detection rate over 25% cells in the cluster of interest, natural log transformed fold change over 0.25, and a p values smaller than 0.01 in Wilcoxon Rank Sum test. Genes differentially expressed between clusters were identified using FindMarkers with the same significant cutoff criteria as used in FindAllMarkers, except that the gene expression detection rate over 25% is required in at least one of the 2 clusters in comparison.

Genes differentially expressed between 2 cell populations were identified by the function FindMarkers of Seurat. The cutoff thresholds used were the same as mentioned above for FindAllMarkers, except that the fold change can be either increase or decrease.

### Cell cluster connectivity and pseudotime analyses

Cell cluster connectivity was analyzed by PAGA (Partition-based graph abstraction) program developed by Wolf et al.^29^ and pseudotime reconstruction^87^ using SCANPY package^88^ in Python^89^.

### Single cell RNASeq data visualization

For QC assessment, histograms and scatter plots were generated using R. Seurat RunUMAP function was used to generate UMAP (Uniform Manifold Approximation and Projection) plots for the visualization of cell cluster ID of or gene expression in each individual cell in conjunction with Seurat function DimPlot or FeaturePlot, respectively. Seurat DotPlot was used to visualize the average expression and percentage of cells expressing a gene in each cluster. Stacked violin plots of the expression of multiple genes across multiple clusters were generated using stacked_violin function of SCANPY in Python^88^. Heatmap was generated using Seurat function DoHeatmap, except in the supplemental figures, heatmaps were generated by using the average of normalized UMI of each gene in each cluster and colored based on the value range of each gene across cell types using Excel conditional formatting (Microsoft Excel 2016, Microsoft Corporation, Redmond, WA).

### Pathway enrichment analysis

Pathway enrichment analyses were performed using NextBio software (Illlumina, San Diego, CA). Bar plots of resulted p values were generated by Excel bar chart (Microsoft Excel 2016, Microsoft Corporation, Redmond, WA).

### NextBio correlation analysis

NextBio correlation analysis was performed using genes differentially expressed between Conj-1, 2, 3 and Epi-S1, 2, 3 (Illlumina, San Diego, CA). Bar plots of resulted p values were generated by Excel bar chart (Microsoft Excel 2016, Microsoft Corporation, Redmond, WA).

### RNAscope

Human corneas were received from The Eye-Bank for Sight Restoration and were immediately placed in proprietary fixative and shipped to Excalibur Pathology Inc. The samples were then paraffin embedded and sectioned at a thickness of 6 mm. The RNAscope 2.5 Duplex kit (ACD Bio) was used and the protocol from ACD Bio was followed. Briefly, slides were incubated at 60 °C for 1 h prior to deparaffination, which consisted of 2–5 min Xylene washes and 2-1 min 100% ethanol washes. Slides were left to dry at RT for 5 min. Afterwards, samples were incubated in hydrogen peroxide (ACD Bio) at RT for 10 min, incubated in RNAscope Target Retrieval Reagent (ACD Bio) at 100 °C for 15 min, and incubated in protease III (ACD Bio) at 40 °C for 15 min, with diH2O washes in between. The slides we dried at 60 °C for 15 min, cooled at RT for 5 min, a barrier was drawn around the samples and stored at RT overnight. Samples were washed in RNAscope Wash Buffer Reagent (ACD Bio) 2 × 2 min and then probes were added to the sections and incubated at 40 °C for 2 h within a humidified chamber. Signal amplification and detection reagents (ACD Bio) were applied sequentially and incubated in AMP 1, AMP 2, AMP 3, AMP 4, AMP 5, AMP 6, AMP 7, AMP 8, Amp 9, and AMP 10 reagents (AMP 1–4,7,8 at 40 °C; AMP 5,6,9,10 at RT), for 30, 15, 30, 15, 30, 15, 15, 30, 30, 15 min, respectively. Before adding each AMP reagent, samples were washed 2 × 2 min with wash buffer. The samples were then counterstained with 50% Gill's hematoxylin I (Vector Laboratories) for 6–8 s at room temperature and briefly rinsed with tap water 2x. Mounting media (VectaMount Permanent Mounting Media; Vector Laboratories) and cover slips were then added to slides for imaging. Images were captured using a Keyence BZX-700 microscope.

## Supplementary Information

Supplementary Information.

## References

[1] Bryan JM (2018). Identifying core biological processes distinguishing human eye tissues with precise systems-level gene expression analyses and weighted correlation networks. Hum. Mol. Genet..

[2] Diehn JJ, Diehn M, Marmor MF, Brown PO (2005). Differential gene expression in anatomical compartments of the human eye. Genome Biol..

[3] Chen Y (2013). Identification of novel molecular markers through transcriptomic analysis in human fetal and adult corneal endothelial cells. Hum. Mol. Genet..

[4] Dyrlund TF (2012). Human cornea proteome: Identification and quantitation of the proteins of the three main layers including epithelium, stroma, and endothelium. J. Proteome Res..

[5] Frausto RF, Wang C, Aldave AJ (2014). Transcriptome analysis of the human corneal endothelium. Invest. Ophthalmol. Vis. Sci..

[6] Kulkarni BB (2010). Comparative transcriptional profiling of the limbal epithelial crypt demonstrates its putative stem cell niche characteristics. BMC Genom..

[7] Takacs L (2011). Differentially expressed genes associated with human limbal epithelial phenotypes: New molecules that potentially facilitate selection of stem cell-enriched populations. Invest. Ophthalmol. Vis. Sci..

[8] Jun AS (2001). Microarray analysis of gene expression in human donor corneas. Arch. Ophthalmol..

[9] Kaplan N (2019). Single-cell RNA transcriptome helps define the limbal/corneal epithelial stem/early transit amplifying cells and how autophagy affects this population. Invest. Ophthalmol. Vis. Sci..

[10] Patel G (2020). Molecular taxonomy of human ocular outflow tissues defined by single-cell transcriptomics. Proc. Natl. Acad. Sci. USA.

[11] Menon M (2019). Single-cell transcriptomic atlas of the human retina identifies cell types associated with age-related macular degeneration. Nat. Commun..

[12] Voigt AP (2019). Molecular characterization of foveal versus peripheral human retina by single-cell RNA sequencing. Exp. Eye Res..

[13] Macosko EZ (2015). Highly parallel genome-wide expression profiling of individual cells using nanoliter droplets. Cell.

[14] Yoshihara M (2015). Discovery of molecular markers to discriminate corneal endothelial cells in the human body. PLoS ONE.

[15] Frausto RF, Le DJ, Aldave AJ (2016). Transcriptomic analysis of cultured corneal endothelial cells as a validation for their use in cell replacement therapy. Cell Transplant..

[16] Frausto RF (2020). Phenotypic and functional characterization of corneal endothelial cells during in vitro expansion. Sci. Rep..

[17] He Z (2016). 3D map of the human corneal endothelial cell. Sci. Rep..

[18] Cheong YK (2013). Identification of cell surface markers glypican-4 and CD200 that differentiate human corneal endothelium from stromal fibroblasts. Invest. Ophthalmol. Vis. Sci..

[19] Chng Z (2013). High throughput gene expression analysis identifies reliable expression markers of human corneal endothelial cells. PLoS ONE.

[20] Liu Y (2017). Human corneal endothelial cells expanded in vitro are a powerful resource for tissue engineering. Int. J. Med. Sci..

[21] Liu CY, Birk DE, Hassell JR, Kane B, Kao WW (2003). Keratocan-deficient mice display alterations in corneal structure. J. Biol. Chem..

[22] Quantock AJ, Meek KM, Chakravarti S (2001). An x-ray diffraction investigation of corneal structure in lumican-deficient mice. Invest. Ophthalmol. Vis. Sci..

[23] Velez-DelValle C, Marsch-Moreno M, Castro-Munozledo F, Kuri-Harcuch W (2011). Decorin gene expression and its regulation in human keratinocytes. Biochem. Biophys. Res. Commun..

[24] Bi Y, Patra P, Faezipour M (2014). Structure of collagen-glycosaminoglycan matrix and the influence to its integrity and stability. Conf. Proc. IEEE Eng. Med. Biol. Soc..

[25] Leffers H (1993). Molecular cloning and expression of the transformation sensitive epithelial marker stratifin. A member of a protein family that has been involved in the protein kinase C signalling pathway. J. Mol. Biol..

[26] Nieto-Miguel T (2011). A comparison of stem cell-related gene expression in the progenitor-rich limbal epithelium and the differentiating central corneal epithelium. Mol. Vis..

[27] Nakatsukasa M (2010). Tumor-associated calcium signal transducer 2 is required for the proper subcellular localization of claudin 1 and 7: Implications in the pathogenesis of gelatinous drop-like corneal dystrophy. Am. J. Pathol..

[28] Pietas A (2002). Molecular cloning and characterization of the human S100A14 gene encoding a novel member of the S100 family. Genomics.

[29] Wolf FA (2019). PAGA: graph abstraction reconciles clustering with trajectory inference through a topology preserving map of single cells. Genome Biol..

[30] Nakatsu MN (2011). Wnt/beta-catenin signaling regulates proliferation of human cornea epithelial stem/progenitor cells. Invest. Ophthalmol. Vis. Sci..

[31] Ramirez-Miranda A, Nakatsu MN, Zarei-Ghanavati S, Nguyen CV, Deng SX (2011). Keratin 13 is a more specific marker of conjunctival epithelium than keratin 19. Mol. Vis..

[32] Poli M (2011). Keratin 13 immunostaining in corneal impression cytology for the diagnosis of limbal stem cell deficiency. Invest. Ophthalmol. Vis. Sci..

[33] Turner HC, Budak MT, Akinci MA, Wolosin JM (2007). Comparative analysis of human conjunctival and corneal epithelial gene expression with oligonucleotide microarrays. Invest. Ophthalmol. Vis. Sci..

[34] Chen Z (2004). Characterization of putative stem cell phenotype in human limbal epithelia. Stem Cells.

[35] Kolli S, Lako M, Figueiredo F, Mudhar H, Ahmad S (2008). Loss of corneal epithelial stem cell properties in outgrowths from human limbal explants cultured on intact amniotic membrane. Regen. Med..

[36] Latta L (2018). Human aniridia limbal epithelial cells lack expression of keratins K3 and K12. Exp. Eye Res..

[37] Ehrlich F (2019). Comparative genomics suggests loss of keratin K24 in three evolutionary lineages of mammals. Sci. Rep..

[38] Magal LG (2009). Clustering and lateral concentration of raft lipids by the MAL protein. Mol. Biol. Cell.

[39] Wong P (2016). The role of connexins in wound healing and repair: Novel therapeutic approaches. Front. Physiol..

[40] Klintworth GK (2009). Corneal dystrophies. Orphanet J. Rare Dis..

[41] Moore CBT, Christie KA, Marshall J, Nesbit MA (2018). Personalised genome editing: The future for corneal dystrophies. Prog Retin Eye Res.

[42] Soh YQ (2020). Corneal dystrophies. Nat. Rev. Dis. Primers.

[43] Sacchetti M (2016). Pathophysiology of corneal dystrophies: From cellular genetic alteration to clinical findings. J. Cell Physiol..

[44] Chao-Shern C (2019). Evaluation of TGFBI corneal dystrophy and molecular diagnostic testing. Eye (Lond).

[45] Collin J (2021). A single cell atlas of human cornea that defines its development, limbal progenitor cells and their interactions with the immune cells. Ocul. Surf..

[46] Gonzalez S, Oh D, Baclagon ER, Zheng JJ, Deng SX (2019). Wnt signaling is required for the maintenance of human limbal stem/progenitor cells in vitro. Invest. Ophthalmol. Vis. Sci..

[47] Seyed-Safi AG, Daniels JT (2020). The limbus: Structure and function. Exp. Eye Res..

[48] Deng SX (2019). Global consensus on definition, classification, diagnosis, and staging of limbal stem cell deficiency. Cornea.

[49] Le Q, Chauhan T, Yung M, Tseng CH, Deng SX (2020). Outcomes of limbal stem cell transplant: A meta-analysis. JAMA Ophthalmol..

[50] Gonzalez G, Sasamoto Y, Ksander BR, Frank MH, Frank NY (2018). Limbal stem cells: Identity, developmental origin, and therapeutic potential. Wiley Interdiscip. Rev. Dev. Biol..

[51] Guo ZH (2018). An insight into the difficulties in the discovery of specific biomarkers of limbal stem cells. Int. J. Mol. Sci..

[52] Figueira EC, Di Girolamo N, Coroneo MT, Wakefield D (2007). The phenotype of limbal epithelial stem cells. Invest. Ophthalmol. Vis. Sci..

[53] Sartaj R (2017). Characterization of slow cycling corneal limbal epithelial cells identifies putative stem cell markers. Sci. Rep..

[54] Nakatsu MN (2013). Preferential biological processes in the human limbus by differential gene profiling. PLoS ONE.

[55] Aomatsu E (2014). Novel SCRG1/BST1 axis regulates self-renewal, migration, and osteogenic differentiation potential in mesenchymal stem cells. Sci. Rep..

[56] Xing S (2012). Establishment of rat bone mesenchymal stem cell lines stably expressing Chondromodulin I. Int. J. Clin. Exp. Med..

[57] Funaki H (2001). Expression and localization of angiogenic inhibitory factor, chondromodulin-I, in adult rat eye. Invest. Ophthalmol. Vis. Sci..

[58] Shi Q (2017). Maintaining the phenotype stability of chondrocytes derived from MSCs by C-type natriuretic peptide. Front. Physiol..

[59] Kim HS (2017). Schwann cell precursors from human pluripotent stem cells as a potential therapeutic target for myelin repair. Stem Cell Rep..

[60] Kutkowska-Kazmierczak A (2015). Phenotypic variability in gap junction syndromic skin disorders: Experience from KID and Clouston syndromes' clinical diagnostics. J. Appl. Genet..

[61] Shibayama J (2005). Functional characterization of connexin43 mutations found in patients with oculodentodigital dysplasia. Circ. Res..

[62] Yuan X (2009). Expression pattern of connexins in the corneal and limbal epithelium of a primate. Cornea.

[63] Shurman DL, Glazewski L, Gumpert A, Zieske JD, Richard G (2005). In vivo and in vitro expression of connexins in the human corneal epithelium. Invest. Ophthalmol. Vis. Sci..

[64] Zhai J, Wang Q, Tao L (2014). Connexin expression patterns in diseased human corneas. Exp. Ther. Med..

[65] Lu X, Watsky MA (2019). Influence of vitamin D on corneal epithelial cell desmosomes and hemidesmosomes. Invest. Ophthalmol. Vis. Sci..

[66] Jaworski CJ (2009). Expression analysis of human pterygium shows a predominance of conjunctival and limbal markers and genes associated with cell migration. Mol. Vis..

[67] Ding Z, Dong J, Liu J, Deng SX (2008). Preferential gene expression in the limbus of the vervet monkey. Mol. Vis..

[68] Irvine AD (1997). Mutations in cornea-specific keratin K3 or K12 genes cause Meesmann's corneal dystrophy. Nat. Genet..

[69] Zhao B (2008). Targeted cornea limbal stem/progenitor cell transfection in an organ culture model. Invest. Ophthalmol. Vis. Sci..

[70] Chen B, Mi S, Wright B, Connon CJ (2010). Investigation of K14/K5 as a stem cell marker in the limbal region of the bovine cornea. PLoS ONE.

[71] Richardson A (2017). Keratin-14-positive precursor cells spawn a population of migratory corneal epithelia that maintain tissue mass throughout life. Stem Cell Rep..

[72] West-Mays JA, Dwivedi DJ (2006). The keratocyte: Corneal stromal cell with variable repair phenotypes. Int. J. Biochem. Cell Biol..

[73] Kim WJ, Rabinowitz YS, Meisler DM, Wilson SE (1999). Keratocyte apoptosis associated with keratoconus. Exp. Eye Res..

[74] Sharif R, Khaled ML, McKay TB, Liu Y, Karamichos D (2019). Transcriptional profiling of corneal stromal cells derived from patients with keratoconus. Sci. Rep..

[75] Foster JW (2018). Integrated stress response and decreased ECM in cultured stromal cells from keratoconus corneas. Invest. Ophthalmol. Vis. Sci..

[76] Sun M (2020). Collagen XII is a regulator of corneal stroma structure and function. Invest. Ophthalmol. Vis. Sci..

[77] Ljubimov AV (2017). Diabetic complications in the cornea. Vis. Res..

[78] Priyadarsini S (2016). Complete metabolome and lipidome analysis reveals novel biomarkers in the human diabetic corneal stroma. Exp. Eye Res..

[79] Saghizadeh M (2013). Enhanced wound healing, kinase and stem cell marker expression in diabetic organ-cultured human corneas upon MMP-10 and cathepsin F gene silencing. Invest. Ophthalmol. Vis. Sci..

[80] Wang KY (2018). Critical in vivo roles of WNT10A in wound healing by regulating collagen expression/synthesis in WNT10A-deficient mice. PLoS ONE.

[81] Kerkhoff C (2012). Novel insights into the role of S100A8/A9 in skin biology. Exp. Dermatol..

[82] Trostrup H (2011). S100A8/A9 deficiency in nonhealing venous leg ulcers uncovered by multiplexed antibody microarray profiling. Br. J. Dermatol..

[83] Kumari SS, Varadaraj M, Menon AG, Varadaraj K (2018). Aquaporin 5 promotes corneal wound healing. Exp. Eye Res..

[84] Gain P (2016). Global survey of corneal transplantation and eye banking. JAMA Ophthalmol..

[85] Butler A, Hoffman P, Smibert P, Papalexi E, Satija R (2018). Integrating single-cell transcriptomic data across different conditions, technologies, and species. Nat. Biotechnol..

[86] Stuart T (2019). Comprehensive integration of single-cell data. Cell.

[87] Haghverdi L, Buttner M, Wolf FA, Buettner F, Theis FJ (2016). Diffusion pseudotime robustly reconstructs lineage branching. Nat. Methods.

[88] Wolf FA, Angerer P, Theis FJ (2018). SCANPY: Large-scale single-cell gene expression data analysis. Genome Biol..

[89] Rossum GV, Drake FL (2009). Python 3 Reference Manual.

