# Supplementary material for: Molecular characteristics and spatial distribution of adult human corneal cell subtypes
Source: Sci Rep. 2021 Aug 11;11:16323. doi: 10.1038/s41598-021-94933-8 (PMC8357950; doi:10.1038/s41598-021-94933-8)
Supplement: Supplementary file 1 — Supplementary Information. [file 41598_2021_94933_MOESM1_ESM.pdf]

Supplementary File

Molecular characteristics and spatial distribution of adult human corneal cell subtypes

Ann J Ligocki, Wen Fury, Christian Gutierrez, Christina Adler, Tao Yang, Min Ni, Yu Bai, Yi Wei, Guillermo L Lehmann, Carmelo Romano

Supplementary Figure 1.

Gene	LPC-1	LPC-2	Epi-B1	Epi-B2	Epi-T	Conj-1	Conj-2	Conj-3	Epi-S1	Epi-S2	Epi-S3	CenC	Stro	LC	Mela	VEC
SFN	7.15	21.56	21.98	16.53	26.64	27.95	40.33	19.43	21.04	19.85	23.41	0.57	0.48	0.91	0.83	1.07
TACSTD2	6.51	24.50	22.07	18.40	32.14	52.66	57.60	55.35	37.89	46.45	71.45	1.09	0.86	1.43	1.82	0.96
KRT5	16.65	32.50	40.01	46.27	62.48	46.16	52.29	14.86	75.99	55.20	57.62	0.58	0.61	1.65	2.26	1.42
S100A4	18.45	31.34	68.48	92.17	80.21	65.72	107.31	94.62	113.04	132.38	146.83	20.93	36.46	22.20	3.83	15.92
PCNA	0.55	0.79	3.20	0.96	0.86	0.84	0.66	0.77	0.90	0.99	0.88	0.48	0.61	0.75	0.71	0.29
TOP2A	0.01	0.02	1.49	0.01	0.01	0.01	0.01	0.01	0.01	0.00	0.00	0.03	0.01	0.07	0.00	0.00
MKI67	0.00	0.00	1.02	0.01	0.01	0.00	0.00	0.00	0.01	0.00	0.00	0.00	0.00	0.05	0.00	0.00
CA3	0.01	0.01	0.01	0.00	0.01	0.03	0.02	0.05	0.00	0.01	0.01	59.38	0.01	0.02	0.00	0.00
MSMP	0.00	0.00	0.00	0.00	0.00	0.00	0.01	0.00	0.00	0.00	0.00	7.79	0.00	0.02	0.00	0.06
EDN3	0.00	0.00	0.00	0.00	0.00	0.00	0.00	0.00	0.00	0.00	0.00	3.67	0.07	0.00	0.00	0.00
KERA	0.10	0.07	0.11	0.09	0.16	0.17	0.22	0.22	0.11	0.11	0.09	0.10	68.86	0.23	0.05	0.18
LUM	0.68	0.39	0.59	0.30	0.45	0.37	0.56	0.54	0.36	0.34	0.29	1.51	117.91	0.27	0.10	13.64
DCN	15.97	8.00	2.51	1.35	3.57	2.37	3.18	3.23	1.95	1.77	1.40	4.37	720.53	1.98	1.36	62.57
PTPRC	0.00	0.00	0.00	0.00	0.00	0.00	0.00	0.00	0.00	0.00	0.00	0.00	0.00	1.56	0.00	0.00
VSIG4	0.00	0.00	0.00	0.00	0.00	0.00	0.00	0.00	0.00	0.00	0.00	0.00	0.00	1.74	0.00	0.00
MSR1	0.00	0.00	0.00	0.00	0.00	0.00	0.00	0.00	0.00	0.00	0.00	0.00	0.00	1.29	0.00	0.00
DCT	0.02	0.12	0.00	0.00	0.02	0.03	0.01	0.00	0.00	0.00	0.00	0.00	0.01	0.17	90.04	0.00
PMEL	0.04	0.07	0.02	0.01	0.02	0.03	0.02	0.03	0.01	0.01	0.01	0.00	0.02	0.14	50.68	0.07
TYRP1	0.03	0.16	0.00	0.00	0.01	0.02	0.00	0.00	0.00	0.00	0.00	0.00	0.00	0.05	40.25	0.00
CLDN5	0.00	0.00	0.00	0.00	0.00	0.00	0.00	0.00	0.00	0.00	0.00	0.02	0.00	0.00	0.00	9.41
PECAM1	0.00	0.00	0.00	0.00	0.00	0.00	0.00	0.00	0.00	0.00	0.00	0.00	0.00	0.33	0.00	1.02
COL4A1	0.01	0.00	0.00	0.00	0.00	0.00	0.00	0.00	0.00	0.00	0.00	0.05	0.00	0.00	0.00	0.58

High

Low

Supplementary Figure 1. Heatmap of cluster marker gene expression. This heatmap corresponds to the stacked violin plot in Figure 1. The genes are the marker genes of each cluster. The value in each cell is the average of normalized UMI of each gene in each cluster and the color is based on the range of values in each row.

Supplementary Figure 2.

Gene	CenC	LPC-1	LPC-2	Epi-B1	Epi-B2	Epi-T	Conj-1	Conj-2	Conj-3	Epi-S1	Epi-S2	Epi-S3	Stro	LC	Mela	VEC
CA3	59.38	0.01	0.01	0.01	0.00	0.01	0.03	0.02	0.05	0.00	0.01	0.01	0.01	0.02	0.00	0.00
RGS5	17.77	0.02	0.02	0.04	0.04	0.02	0.03	0.03	0.02	0.03	0.06	0.04	0.02	0.00	0.00	0.44
FGF7	8.29	0.01	0.00	0.01	0.00	0.01	0.00	0.00	0.00	0.00	0.00	0.00	0.12	0.00	0.00	0.43
MSMP	7.79	0.00	0.00	0.00	0.00	0.00	0.00	0.01	0.00	0.00	0.00	0.00	0.00	0.02	0.00	0.06
SFRP1	8.01	0.03	0.08	0.00	0.01	0.02	0.00	0.00	0.00	0.00	0.00	0.00	0.69	0.00	0.00	0.24
TNNC1	7.15	0.00	0.01	0.00	0.01	0.01	0.02	0.03	0.07	0.01	0.02	0.08	0.17	0.00	0.00	0.00
CA12	7.99	0.15	0.48	0.18	0.02	0.73	0.48	0.26	0.17	0.10	0.02	0.01	0.14	0.02	0.00	0.08
SCD	6.68	0.01	0.05	0.12	0.11	0.03	0.03	0.04	0.03	0.17	0.09	0.16	0.06	0.03	0.54	0.00
TSPAN6	8.28	0.45	0.32	0.29	0.26	0.30	0.48	0.46	0.73	0.25	0.24	0.30	0.79	0.04	0.21	0.31
TMEM178A	3.71	0.01	0.00	0.00	0.00	0.00	0.00	0.00	0.01	0.00	0.00	0.00	0.01	0.00	0.00	0.00
EDN3	3.67	0.00	0.00	0.00	0.00	0.00	0.00	0.00	0.00	0.00	0.00	0.00	0.07	0.00	0.00	0.00
CDH2	0.72	0.00	0.02	0.00	0.00	0.00	0.00	0.00	0.00	0.00	0.00	0.00	0.00	0.00	0.00	0.00
FZD2	0.75	0.01	0.01	0.00	0.00	0.00	0.00	0.00	0.00	0.00	0.00	0.00	0.02	0.08	0.00	0.00
FGFR1	0.70	0.19	0.12	0.01	0.00	0.02	0.08	0.05	0.06	0.01	0.00	0.01	1.43	0.10	0.03	1.52
SLC4A11	9.51	0.37	0.13	0.05	0.06	0.05	0.05	0.05	0.11	0.09	0.11	0.31	0.28	0.00	0.00	0.00
SLC4A4	2.84	0.01	0.02	0.00	0.00	0.00	0.02	0.01	0.02	0.00	0.00	0.00	0.20	0.00	0.00	0.00
ATP1B2	0.63	0.06	0.04	0.00	0.00	0.01	0.03	0.01	0.06	0.01	0.00	0.00	0.01	0.01	0.08	0.09
ATP1B1	4.24	1.47	0.64	0.22	0.20	0.55	0.69	0.51	0.88	0.36	0.22	0.31	0.01	0.74	1.32	0.06
ATP1A1	5.12	2.66	1.86	1.33	1.26	1.75	1.71	1.38	1.80	1.25	1.07	0.70	0.63	0.99	3.66	1.53

High

Low

Supplementary Figure 2. Heatmap of cornea endothelial cluster marker gene expression across all clusters. This heatmap corresponds to the stacked violin plot in Figure 2. The value in each cell is the average of normalized UMI of each gene in each cluster and the color is based on the range of values in each row.

Supplementary Figure 3.

Gene	Stro	LPC-1	LPC-2	Epi-B1	Epi-B2	Epi-T	Conj-1	Conj-2	Conj-3	Epi-S1	Epi-S2	Epi-S3	CenC	LC	Mela	VEC
DCN	720.53	15.97	8.00	2.51	1.35	3.57	2.37	3.18	3.23	1.95	1.77	1.40	4.37	1.98	1.36	62.57
SAA1	125.74	0.70	0.26	1.56	0.24	0.51	0.43	0.63	0.85	0.33	0.35	0.34	0.61	0.66	0.68	0.55
LUM	117.91	0.68	0.39	0.59	0.30	0.45	0.37	0.56	0.54	0.36	0.34	0.29	1.51	0.27	0.10	13.64
KERA	68.86	0.10	0.07	0.11	0.09	0.16	0.17	0.22	0.22	0.11	0.11	0.09	0.10	0.23	0.05	0.18
ANGPTL7	131.98	0.37	0.79	2.23	4.85	0.47	0.39	0.60	0.55	1.06	0.98	0.62	0.96	0.65	0.45	4.70
ITGBL1	20.75	0.44	0.13	0.04	0.02	0.07	0.05	0.08	0.12	0.04	0.04	0.04	1.34	0.12	0.16	1.89
PLAC9	14.00	0.16	0.12	0.09	0.06	0.14	0.11	0.15	0.07	0.10	0.06	0.06	0.00	0.12	0.00	1.51
GRP	8.25	0.08	0.01	0.02	0.02	0.03	0.02	0.03	0.03	0.01	0.02	0.03	0.00	0.06	0.10	0.10
THBS4	6.42	0.02	0.01	0.02	0.01	0.01	0.02	0.02	0.01	0.01	0.02	0.01	0.09	0.00	0.00	0.86
HTRA1	8.79	0.06	0.36	1.11	1.50	0.16	0.11	0.11	0.13	0.54	0.30	0.15	1.01	0.23	0.00	0.31
ECM1	8.62	0.11	0.25	0.83	1.25	0.28	0.15	0.45	0.15	0.92	1.11	1.49	0.00	0.10	0.12	1.04
F13A1	3.59	0.01	0.00	0.01	0.01	0.00	0.01	0.02	0.05	0.01	0.01	0.00	0.19	0.08	0.00	0.38

High

Low

Supplementary Figure 3. Heatmap of stromal cluster marker gene expression across all clusters. This heatmap corresponds to the stacked violin plot in Figure 3. The value in each cell is the average of normalized UMI of each gene in each cluster and the color is based on the range of values in each row.

Supplementary Figure 4.

Gene	LPC-1	LPC-2	Epi-B1	Epi-B2	Epi-T	Epi-S1	Epi-S2	Epi-S3	Conj-3	Conj-2	Conj-1
SFN	7.15	21.56	21.98	16.53	26.64	21.04	19.85	23.41	19.43	40.33	27.95
FRZB	9.91	0.15	0.02	0.00	0.05	0.00	0.00	0.00	0.00	0.01	0.00
SCRG1	29.33	0.15	0.13	0.02	0.19	0.02	0.01	0.01	0.01	0.01	0.02
GPHA2	182.09	5.58	0.62	0.11	12.56	0.13	0.12	0.10	0.08	0.09	0.22
MMP10	2.30	2.97	0.04	0.03	0.07	0.05	0.01	0.01	0.02	0.10	0.03
UBE2C	0.00	0.01	4.74	0.01	0.01	0.01	0.01	0.00	0.01	0.00	0.01
CKS2	0.18	0.53	6.37	0.47	0.58	0.46	0.36	0.30	0.67	0.58	0.93
HMGB2	1.29	1.26	8.88	0.94	1.13	0.87	0.57	0.31	0.64	0.67	1.05
CPVL	0.05	0.78	1.87	3.49	0.04	0.43	0.15	0.03	0.01	0.03	0.02
WNT10A	0.56	0.65	0.48	1.26	0.05	0.16	0.03	0.01	0.00	0.01	0.02
KRT14	58.30	92.25	87.35	76.97	61.79	27.75	2.53	0.83	1.00	5.98	4.64
MAL	0.06	0.18	1.07	1.42	0.42	3.25	7.93	11.74	0.34	1.00	0.11
KRT3	0.44	1.31	4.64	5.83	0.83	19.55	33.07	35.08	0.42	2.06	0.47
KRT24	0.07	0.10	0.34	0.34	0.30	0.62	1.65	33.26	0.17	4.47	0.22
CALML5	0.13	0.20	0.31	0.19	0.53	0.52	1.02	3.45	0.50	0.90	0.29
MSMB	0.01	0.02	0.03	0.02	0.06	0.15	0.72	3.37	0.92	0.43	0.22
LYPD2	0.03	0.01	0.04	0.03	0.04	0.15	0.30	8.80	13.02	2.36	0.28
PSCA	0.01	0.01	0.03	0.02	0.05	0.17	0.64	6.14	10.86	1.17	0.59
CEACAM7	0.00	0.00	0.00	0.00	0.00	0.01	0.00	0.01	3.49	0.18	0.08
KRT7	0.05	0.06	0.09	0.07	0.09	0.04	0.03	0.05	3.46	0.32	0.66
KRT6A	0.02	0.40	1.11	0.10	1.09	0.75	0.36	0.49	0.80	5.58	1.18
SPRR1B	0.00	0.11	0.12	0.01	0.30	0.46	0.15	0.18	2.36	6.85	0.97
AQP5	0.09	0.23	0.34	0.07	0.32	0.17	0.13	0.79	57.13	13.18	11.51
KRT4	0.07	0.07	0.36	0.06	0.25	0.13	0.09	0.70	20.97	7.61	9.29
LCN2	0.15	0.21	0.31	0.20	0.50	0.24	0.09	0.30	17.83	4.88	2.91
KRT15	84.26	52.86	6.15	5.10	49.79	2.51	0.44	0.28	3.28	12.25	11.79
ADIRF	6.02	17.24	39.91	58.76	35.91	107.19	193.87	267.73	54.00	68.36	46.19

High

Low

Supplementary Figure 4. Heatmap of epithelial cluster marker gene expression across epithelial clusters. This heatmap corresponds to the stacked violin plot in Figure 4. The value in each cell is the average of normalized UMI of each gene in each cluster and the color is based on the range of values in each row.

Supplementary Figure 5.

Gene	LPC-1	LPC-2	Epi-B1	Epi-B2	Epi-T	Epi-S1	Epi-S2	Epi-S3
SCRG1	29.33	0.15	0.13	0.02	0.19	0.02	0.01	0.01
FRZB	9.91	0.15	0.02	0.00	0.05	0.00	0.00	0.00
LECT1	2.35	0.01	0.01	0.00	0.01	0.00	0.00	0.00
NPPC	2.09	0.12	0.00	0.00	0.02	0.00	0.00	0.00
CDH19	1.27	0.02	0.00	0.00	0.02	0.00	0.00	0.00
GPHA2	182.09	5.58	0.62	0.11	12.56	0.13	0.12	0.10
MMP10	2.30	2.97	0.04	0.03	0.07	0.05	0.01	0.01
FAM107A	2.79	1.39	0.10	0.03	0.13	0.03	0.01	0.01
SLC6A6	3.10	1.93	0.16	0.19	0.17	0.12	0.08	0.07
SPARCL1	8.22	1.60	0.13	0.05	1.39	0.13	0.02	0.02
PTN	4.47	0.96	0.83	0.18	0.87	0.21	0.01	0.00

High

Low

Supplementary Figure 5. Heatmap of limbal epithelial stem cell cluster marker gene expression across all cornea epithelial clusters. This heatmap corresponds to the stacked violin plot in Figure 5. The value in each cell is the average of normalized UMI of each gene in each cluster and the color is based on the range of values in each row.

Supplementary Figure 6.

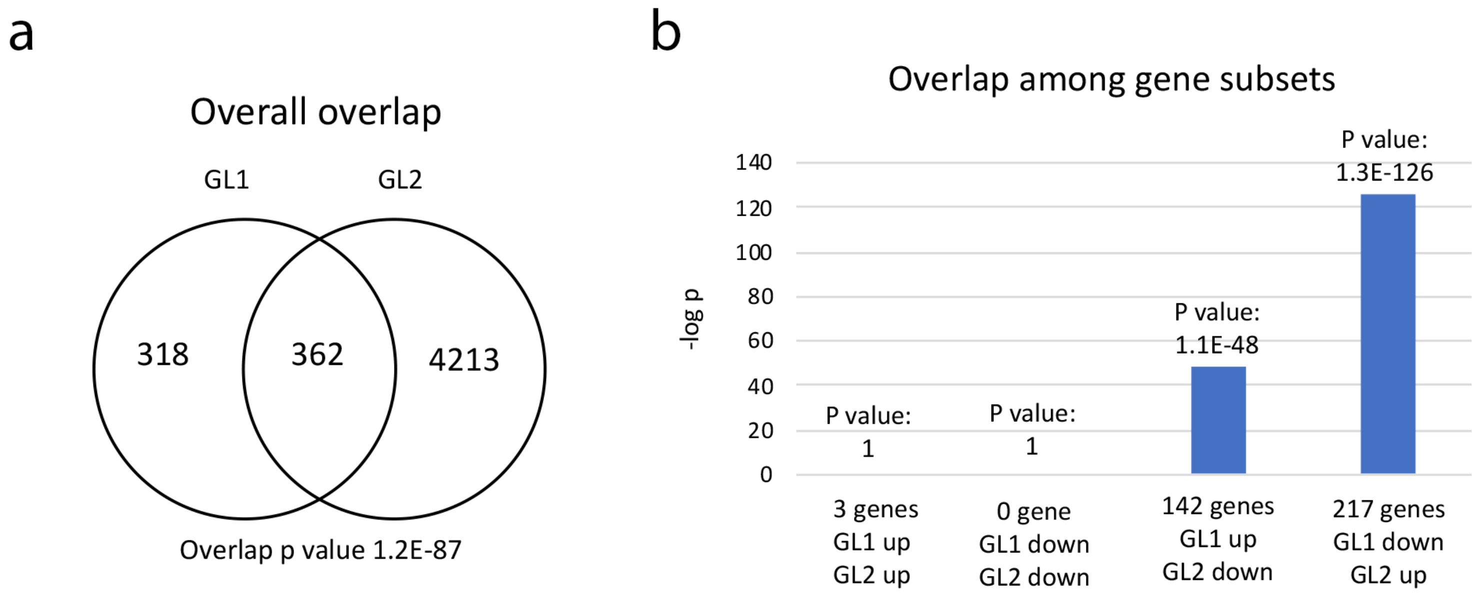

Supplementary Figure 6. NextBio correlation analysis suggesting Conj-1, 2 and 3 as conjunctiva epithelial cells. Genes significantly differentially expressed in Conj-1, 2 and 3 vs. Epi-S1, 2 and 3 comparison (GL1) was used as input for NextBio correlation analysis (BaseSpace Correlation Engine) of their curated studies. The topmost correlated gene list was generated from cornea vs. conjunctiva epithelial cell comparison (GL2) (Ramierz-Miranda et al, 2011; Mol Vis 17:1652-1661). A. Overall overlap between the 2 gene lists and associated p value. B. Overlap among gene subsets and associated p value.

Supplementary Figure 7.

Gene	CenC	Stro	LPC-1	LPC-2	Epi-B1	Epi-B2	Epi-T	Conj-1	Conj-2	Conj-3	Epi-S1	Epi-S2	Epi-S3	LC	Mela	VEC
COL8A2	4.21	0.67	0.00	0.00	0.00	0.00	0.00	0.00	0.00	0.00	0.00	0.00	0.00	0.01	0.00	0.00
SLC4A11	9.51	0.28	0.37	0.13	0.05	0.06	0.05	0.05	0.05	0.11	0.09	0.11	0.31	0.00	0.00	0.00
TCF4	1.92	2.63	0.24	0.16	0.02	0.00	0.03	0.03	0.01	0.01	0.01	0.01	0.01	0.35	0.00	5.23
ZEB1	0.13	0.77	0.00	0.01	0.00	0.00	0.00	0.01	0.01	0.01	0.00	0.00	0.00	0.00	0.00	0.65
PRDM5	0.12	0.12	0.00	0.01	0.00	0.01	0.01	0.01	0.01	0.01	0.00	0.00	0.00	0.00	0.00	0.00
COL4A3	2.51	0.38	0.01	0.01	0.01	0.02	0.01	0.00	0.00	0.00	0.01	0.01	0.01	0.00	0.00	0.00
COL4A4	1.02	0.18	0.01	0.01	0.05	0.06	0.01	0.00	0.00	0.00	0.03	0.01	0.01	0.00	0.00	0.00
DCN	4.37	720.53	15.97	8.00	2.51	1.35	3.57	2.37	3.18	3.23	1.95	1.77	1.40	1.98	1.36	62.57
GSN	2.94	4.83	10.09	6.05	12.79	11.95	4.56	2.62	2.89	3.91	13.34	11.89	8.58	3.24	2.98	17.41
KERA	0.10	68.86	0.10	0.07	0.11	0.09	0.16	0.17	0.22	0.22	0.11	0.11	0.09	0.23	0.05	0.18
LUM	1.51	117.91	0.68	0.39	0.59	0.30	0.45	0.37	0.56	0.54	0.36	0.34	0.29	0.27	0.10	13.64
PIKFYVE	0.20	0.09	0.03	0.06	0.05	0.04	0.04	0.02	0.02	0.02	0.06	0.04	0.02	0.05	0.14	0.12
COL5A1	0.00	0.08	0.03	0.04	0.02	0.02	0.00	0.00	0.00	0.00	0.00	0.00	0.00	0.00	0.00	0.25
COL17A1	0.00	0.04	2.79	3.21	4.10	5.63	1.55	0.14	0.21	0.06	3.67	1.56	0.49	0.10	0.05	0.06
PAX6	0.00	0.06	7.13	4.50	5.49	4.98	6.29	5.67	4.31	4.81	6.00	6.83	7.04	0.16	0.29	0.25
KRT12	1.83	1.41	9.20	27.96	76.57	115.39	44.90	2.55	10.29	2.60	159.21	202.24	201.14	6.11	5.99	3.33
KRT3	0.20	0.20	0.44	1.31	4.64	5.83	0.83	0.47	2.06	0.42	19.55	33.07	35.08	0.73	0.81	1.32
TACSTD2	1.09	0.86	6.51	24.50	22.07	18.40	32.14	52.66	57.60	55.35	37.89	46.45	71.45	1.43	1.82	0.96
TGFBI	2.57	8.22	38.78	22.51	39.63	62.92	16.15	1.86	3.39	1.56	44.08	47.40	32.28	2.85	1.01	1.90

High

Low

Supplementary Figure 7. Heatmap of the expression of cornea disease-related genes across all clusters. This heatmap corresponds to the stacked violin plot in Figure 7. The value in each cell is the average of normalized UMI of each gene in each cluster and the color is based on the range of values in each row.

Supplementary Figure 8.

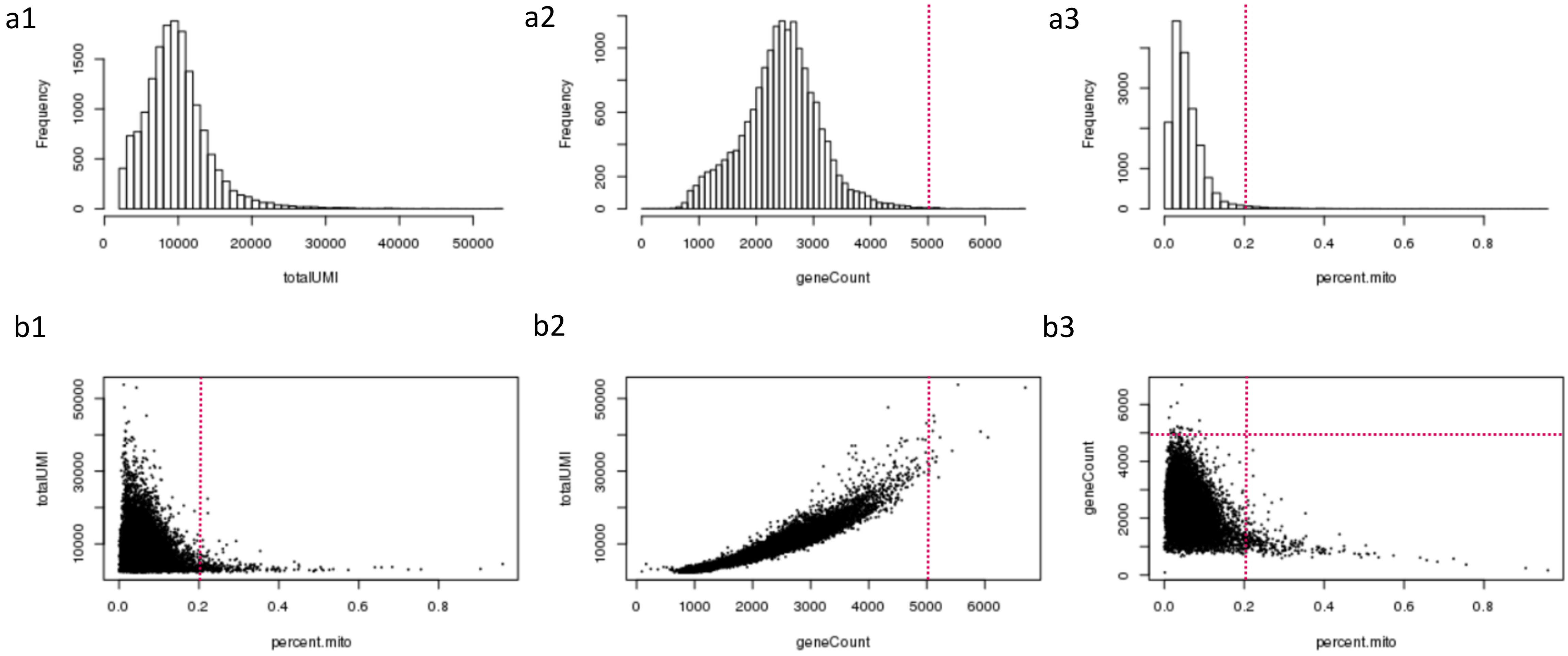

Supplementary Figure 8. Cell filtering based on QC. **a(1-3),** Histogram of total UMI, number of genes detected or mitochondrial to total read count ratio across all cells, respectively. **b(1-3),** Scatter plots of QC parameters. Red lines are the QC filtering criteria: cells that have less than 5000 genes detected and less than 20% mitochondria reads are kept for further analysis.

Molecular characteristics and spatial distribution of adult human corneal cell subtypes

Ann J Ligocki, Wen Fury, Christian Gutierrez, Christina Adler, Tao Yang, Min Ni, Yu Bai, Yi Wei ,Guillermo L Lehmann, Carmelo Romano

Supplementary Table 1. Gene expression values of cell cluster markers

p_val: p value
avg_LogFC: natural log transformed average UMI ratio between the cells of the specified cluster (column G) and the cells outside the cluster
pct.1: percentage of cells in the specific cluster (column G) that the expression of a gene is detected
pct.2: percentage of cells outside the specified cluster that the expression of a gene is detected
p_val_adj: multiple test adjusted p value
cluster: the cluster for which the corresponding gene in the same row is a marker for

gene	p_val	avg_logFC	pct.1	pct.2	p_val_adj	cluster
KRT3	0	1.533842	0.981	0.461	0	Epi-S2
MAL	0	1.221952	0.982	0.319	0	Epi-S2
KRT12	0	1.190519	0.995	0.781	0	Epi-S2
ADIRF	0	1.121309	1	0.982	0	Epi-S2
DAPL1	0	1.074493	0.999	0.792	0	Epi-S2
UPK1B	0	1.010959	0.995	0.653	0	Epi-S2
TKT	0	0.937521	0.999	0.83	0	Epi-S2
MGARP	0	0.898707	0.994	0.78	0	Epi-S2
RB1	0	0.877901	0.899	0.417	0	Epi-S2
MRPL33	0	0.874903	0.999	0.877	0	Epi-S2
MAL2	0	0.812855	0.875	0.396	0	Epi-S2
CTSL2	0	0.792462	0.991	0.555	0	Epi-S2
C8orf47	0	0.768472	0.99	0.691	0	Epi-S2
PIR	0	0.757	0.858	0.437	0	Epi-S2
CTSC	0	0.731736	0.858	0.499	0	Epi-S2
CLDN7	0	0.720699	0.972	0.523	0	Epi-S2
ADH7	0	0.714521	0.944	0.537	0	Epi-S2
GJB2	0	0.703694	0.995	0.587	0	Epi-S2
ALDH1A1	0	0.696412	0.999	0.84	0	Epi-S2
CALM2	0	0.691704	1	0.974	0	Epi-S2
GJB6	0	0.691147	0.988	0.521	0	Epi-S2
AGR2	0	0.683944	0.928	0.568	0	Epi-S2
LYPD3	0	0.682973	1	0.802	0	Epi-S2
C12orf75	0	0.681754	0.948	0.565	0	Epi-S2
TUBA4A	0	0.678015	0.966	0.684	0	Epi-S2
TGFBI	0	0.661449	0.997	0.876	0	Epi-S2
ENO1	0	0.644201	1	0.993	0	Epi-S2
GJB4	0	0.629215	0.814	0.37	0	Epi-S2
GYG1	0	0.613534	0.86	0.516	0	Epi-S2
NQO1	0	0.61087	1	0.932	0	Epi-S2
IER3IP1	0	0.59582	0.924	0.701	0	Epi-S2
CYP26A1	0	0.590006	0.366	0.082	0	Epi-S2
ADRB2	0	0.589218	0.96	0.673	0	Epi-S2
HCAR3	0	0.585014	0.829	0.514	0	Epi-S2

CA6	0	0.57693	0.391	0.067	0	Epi-S2
LAD1	0	0.561617	0.803	0.38	0	Epi-S2
LOC729966	0	0.553276	0.718	0.286	0	Epi-S2
PERP	0	0.548075	1	0.899	0	Epi-S2
TSTD1	0	0.545269	0.97	0.726	0	Epi-S2
CSRP2	0	0.541309	0.996	0.849	0	Epi-S2
SCD5	0	0.534089	0.751	0.375	0	Epi-S2
PMVK	0	0.530344	0.93	0.68	0	Epi-S2
S100A4	0	0.529959	1	0.995	0	Epi-S2
AIM1	0	0.518762	0.777	0.372	0	Epi-S2
SLURP1	0	0.518758	0.546	0.158	0	Epi-S2
HCAR2	0	0.516907	0.922	0.624	0	Epi-S2
DSC2	0	0.499943	0.843	0.443	0	Epi-S2
MT1X	0	0.496367	1	0.969	0	Epi-S2
BCL2L13	0	0.495767	0.951	0.714	0	Epi-S2
CRTAC1	0	0.493243	0.993	0.617	0	Epi-S2
TRIM36	0	0.485929	0.717	0.304	0	Epi-S2
MRPS10	0	0.484851	0.856	0.574	0	Epi-S2
LDHB	0	0.48483	0.949	0.807	0	Epi-S2
IL20RA	0	0.478574	0.704	0.353	0	Epi-S2
CLU	0	0.470969	1	0.989	0	Epi-S2
MUC15	0	0.468372	0.745	0.377	0	Epi-S2
GSN	0	0.467291	0.995	0.882	0	Epi-S2
CAPG	0	0.466127	0.97	0.789	0	Epi-S2
SDC1	0	0.461734	0.994	0.914	0	Epi-S2
C4orf19	0	0.460507	0.617	0.223	0	Epi-S2
AGPAT2	0	0.46004	0.819	0.526	0	Epi-S2
CLCA4	0	0.458842	0.594	0.243	0	Epi-S2
PRDX1	0	0.458833	1	0.984	0	Epi-S2
TPD52	0	0.44719	0.903	0.61	0	Epi-S2
DSG1	0	0.445545	0.767	0.409	0	Epi-S2
COMT	0	0.438718	0.977	0.83	0	Epi-S2
LINC00937	0	0.437519	0.465	0.129	0	Epi-S2
PPIA	0	0.429215	0.995	0.95	0	Epi-S2
SRP68	0	0.427307	0.766	0.475	0	Epi-S2
RCBTB2	0	0.427204	0.57	0.23	0	Epi-S2
TP53I3	0	0.423754	0.749	0.43	0	Epi-S2
ATP6V0B	0	0.418276	0.973	0.845	0	Epi-S2
SEC16B	0	0.415994	0.456	0.108	0	Epi-S2
LGALSL	0	0.41411	0.616	0.264	0	Epi-S2
NDUFV2	0	0.409118	0.954	0.801	0	Epi-S2
GSTP1	0	0.407836	1	0.996	0	Epi-S2
VAMP8	0	0.406028	0.963	0.708	0	Epi-S2
PPP1R14C	0	0.398256	0.659	0.306	0	Epi-S2
TAGLN2	0	0.397018	0.996	0.944	0	Epi-S2
PPDPF	0	0.395975	1	0.958	0	Epi-S2
SERBP1	0	0.394937	0.959	0.839	0	Epi-S2
DSP	0	0.392781	0.956	0.706	0	Epi-S2

PVRL4	0	0.388466	0.79	0.426	0	Epi-S2
CHCHD2	0	0.388257	0.997	0.977	0	Epi-S2
ANXA2	0	0.385765	1	0.993	0	Epi-S2
MYO5A	0	0.385737	0.411	0.111	0	Epi-S2
COX7B	0	0.38385	0.991	0.921	0	Epi-S2
NDUFB3	0	0.381807	0.941	0.792	0	Epi-S2
POLR2J2	0	0.371511	1	0.848	0	Epi-S2
POLR2J3	0	0.36897	0.999	0.838	0	Epi-S2
LGALS3	0	0.36357	1	0.986	0	Epi-S2
PRDX5	0	0.358293	0.998	0.97	0	Epi-S2
MFSD4	0	0.35621	0.559	0.241	0	Epi-S2
FSIP1	0	0.35414	0.444	0.128	0	Epi-S2
LOC100130476	0	0.344948	0.413	0.099	0	Epi-S2
DBI	0	0.343157	0.991	0.901	0	Epi-S2
RAB6B	0	0.340133	0.344	0.068	0	Epi-S2
COX6A1	0	0.326384	0.999	0.972	0	Epi-S2
COX6C	0	0.322123	0.996	0.959	0	Epi-S2
ACTG1	0	0.319103	1	0.993	0	Epi-S2
DCDC5	0	0.317473	0.428	0.142	0	Epi-S2
SH3GL3	0	0.315423	0.385	0.113	0	Epi-S2
COX7C	0	0.312682	0.999	0.98	0	Epi-S2
COX7A2	0	0.297634	0.998	0.97	0	Epi-S2
MTUS2	0	0.292735	0.351	0.091	0	Epi-S2
ITM2B	0	0.282651	1	0.995	0	Epi-S2
PRPSAP1	6.52E-308	0.396843	0.642	0.345	1.19E-303	Epi-S2
ENSG0000019871[illegible]	2.16E-302	0.32566	1	0.996	3.94E-298	Epi-S2
RELL1	2.96E-302	0.44993	0.522	0.221	5.38E-298	Epi-S2
ELP4	8.47E-302	0.402976	0.585	0.276	1.54E-297	Epi-S2
DAAM1	8.60E-301	0.443238	0.857	0.577	1.57E-296	Epi-S2
VSNL1	7.57E-300	0.468858	0.823	0.542	1.38E-295	Epi-S2
RPA3	1.19E-298	0.402149	0.801	0.519	2.16E-294	Epi-S2
MYL12B	9.53E-296	0.279843	0.998	0.966	1.74E-291	Epi-S2
GHITM	2.60E-295	0.367829	0.952	0.826	4.73E-291	Epi-S2
COX5A	6.33E-291	0.342917	0.96	0.834	1.15E-286	Epi-S2
GALNT18	6.40E-291	0.320167	0.473	0.187	1.17E-286	Epi-S2
HSPB1	3.19E-290	0.379953	1	0.981	5.81E-286	Epi-S2
SCIN	5.35E-288	0.364211	0.551	0.248	9.74E-284	Epi-S2
NSG1	6.03E-288	0.376596	0.695	0.385	1.10E-283	Epi-S2
GNB2	1.35E-284	0.357089	0.945	0.813	2.46E-280	Epi-S2
C1QBP	3.26E-284	0.334757	0.978	0.846	5.93E-280	Epi-S2
MYH14	2.41E-283	0.336384	0.601	0.288	4.39E-279	Epi-S2
LYPLAL1	9.34E-283	0.390975	0.724	0.445	1.70E-278	Epi-S2
FTH1	1.37E-281	0.272528	1	1	2.49E-277	Epi-S2
SH3BGRL3	3.10E-281	0.322538	0.996	0.941	5.65E-277	Epi-S2
SPINT2	1.43E-280	0.344692	0.995	0.793	2.60E-276	Epi-S2
PEBP1	2.09E-276	0.278847	0.997	0.965	3.81E-272	Epi-S2
CLC	3.89E-272	0.262637	0.293	0.076	7.09E-268	Epi-S2
PAX6	2.15E-271	0.370862	0.983	0.75	3.92E-267	Epi-S2

COX5B	2.21E-270	0.278752	0.992	0.954	4.03E-266	Epi-S2
C14orf2	1.31E-269	0.296191	0.988	0.943	2.39E-265	Epi-S2
C10orf54	1.55E-269	0.402313	0.892	0.686	2.81E-265	Epi-S2
TBCB	1.34E-268	0.365272	0.852	0.633	2.44E-264	Epi-S2
NAGK	3.05E-268	0.369092	0.676	0.391	5.56E-264	Epi-S2
FXYD3	1.14E-267	0.344034	1	0.827	2.08E-263	Epi-S2
BBOX1	2.93E-267	0.330143	0.58	0.275	5.33E-263	Epi-S2
TACSTD2	1.51E-265	0.381182	1	0.874	2.76E-261	Epi-S2
FA2H	1.92E-263	0.345359	0.618	0.3	3.50E-259	Epi-S2
SPINT1	2.22E-263	0.357585	0.859	0.591	4.04E-259	Epi-S2
ATP5I	1.56E-262	0.311372	0.979	0.902	2.83E-258	Epi-S2
USMG5	4.01E-262	0.281543	0.986	0.94	7.29E-258	Epi-S2
UQCR11	4.71E-261	0.276083	0.996	0.957	8.58E-257	Epi-S2
CLDN4	4.69E-260	0.453226	0.902	0.541	8.54E-256	Epi-S2
RBX1	1.77E-257	0.319896	0.958	0.865	3.22E-253	Epi-S2
KIF21A	5.58E-257	0.359554	0.672	0.377	1.02E-252	Epi-S2
OSTF1	3.98E-256	0.357102	0.826	0.582	7.24E-252	Epi-S2
POLR2I	6.09E-254	0.351497	0.888	0.71	1.11E-249	Epi-S2
TIMM8B	2.14E-251	0.337606	0.894	0.697	3.89E-247	Epi-S2
ASPH	4.89E-251	0.383158	0.951	0.812	8.90E-247	Epi-S2
RER1	7.60E-250	0.347459	0.903	0.708	1.38E-245	Epi-S2
GALNT3	1.09E-249	0.337638	0.539	0.259	1.98E-245	Epi-S2
ULBP2	2.13E-244	0.304071	0.487	0.216	3.88E-240	Epi-S2
PSMB6	2.03E-242	0.311181	0.94	0.824	3.70E-238	Epi-S2
TCEB2	1.22E-241	0.266189	0.992	0.952	2.23E-237	Epi-S2
TRIM29	6.17E-241	0.324893	0.959	0.728	1.12E-236	Epi-S2
NDUFB9	6.63E-241	0.322043	0.949	0.829	1.21E-236	Epi-S2
CAPS	4.60E-239	0.382783	0.737	0.449	8.38E-235	Epi-S2
DKK3	2.14E-238	0.303653	0.89	0.577	3.89E-234	Epi-S2
PSMB3	3.48E-237	0.307525	0.955	0.838	6.34E-233	Epi-S2
UQCR10	3.47E-235	0.290775	0.976	0.882	6.33E-231	Epi-S2
SPHK1	5.60E-235	0.274981	0.389	0.148	1.02E-230	Epi-S2
LOC728554	1.48E-234	0.34897	0.834	0.587	2.69E-230	Epi-S2
RAB25	4.61E-233	0.332532	0.819	0.521	8.40E-229	Epi-S2
SNRPG	1.07E-231	0.283601	0.968	0.902	1.94E-227	Epi-S2
UXS1	5.43E-231	0.336714	0.64	0.37	9.90E-227	Epi-S2
S100A14	1.45E-229	0.326378	0.972	0.724	2.64E-225	Epi-S2
GIPC1	2.81E-228	0.326283	0.877	0.654	5.12E-224	Epi-S2
SCCPDH	2.96E-228	0.286632	0.454	0.2	5.38E-224	Epi-S2
ATPIF1	3.67E-228	0.301476	0.977	0.878	6.69E-224	Epi-S2
EML2	2.87E-225	0.322715	0.658	0.393	5.22E-221	Epi-S2
TMSB4X	2.87E-225	0.283354	1	0.998	5.23E-221	Epi-S2
PHLDA3	8.87E-225	0.321042	0.915	0.707	1.62E-220	Epi-S2
SLIRP	1.45E-224	0.297672	0.956	0.86	2.64E-220	Epi-S2
CAPNS1	4.05E-223	0.354507	0.813	0.591	7.38E-219	Epi-S2
C4orf3	8.29E-223	0.296101	0.978	0.918	1.51E-218	Epi-S2
ATP5J2	4.86E-222	0.27154	0.981	0.904	8.85E-218	Epi-S2
GCHFR	2.36E-221	0.375465	0.682	0.426	4.30E-217	Epi-S2

MAPK13	6.88E-221	0.302083	0.491	0.239	1.25E-216	Epi-S2
ATP6V1F	1.45E-219	0.28533	0.972	0.871	2.65E-215	Epi-S2
UBL5	1.70E-219	0.258435	0.989	0.945	3.09E-215	Epi-S2
RHOD	2.88E-218	0.324539	0.657	0.393	5.25E-214	Epi-S2
RAET1G	4.68E-218	0.322587	0.66	0.382	8.52E-214	Epi-S2
POMP	5.28E-218	0.266647	0.978	0.914	9.62E-214	Epi-S2
NDUFB4	9.97E-218	0.276256	0.976	0.9	1.81E-213	Epi-S2
TUFT1	1.36E-215	0.337755	0.582	0.323	2.47E-211	Epi-S2
NDUFC1	8.27E-213	0.276013	0.975	0.898	1.51E-208	Epi-S2
FKBP1A	2.93E-212	0.29967	0.942	0.817	5.34E-208	Epi-S2
ATP5J	3.63E-211	0.259092	0.985	0.928	6.61E-207	Epi-S2
MOCS2	1.52E-210	0.336496	0.677	0.432	2.76E-206	Epi-S2
MGST3	1.85E-210	0.264646	0.981	0.913	3.37E-206	Epi-S2
ECHS1	1.50E-209	0.299475	0.906	0.75	2.74E-205	Epi-S2
FAM169A	6.69E-209	0.256242	0.41	0.171	1.22E-204	Epi-S2
SDR16C5	1.60E-208	0.295127	0.565	0.301	2.92E-204	Epi-S2
MKRN1	6.08E-206	0.337076	0.73	0.504	1.11E-201	Epi-S2
DCXR	1.60E-205	0.329804	0.851	0.666	2.92E-201	Epi-S2
PID1	6.50E-205	0.297224	0.574	0.302	1.18E-200	Epi-S2
POF1B	2.44E-204	0.252185	0.373	0.151	4.44E-200	Epi-S2
ALDH3A2	1.80E-203	0.344459	0.806	0.604	3.28E-199	Epi-S2
CALML5	6.93E-202	0.275925	0.448	0.202	1.26E-197	Epi-S2
NMU	9.74E-202	0.259579	0.461	0.216	1.77E-197	Epi-S2
NDUFAB1	2.42E-200	0.295445	0.908	0.773	4.41E-196	Epi-S2
ATP5G3	2.17E-199	0.276077	0.965	0.884	3.94E-195	Epi-S2
WLS	2.67E-199	0.303829	0.699	0.44	4.86E-195	Epi-S2
SNCG	3.57E-199	0.320433	0.58	0.317	6.50E-195	Epi-S2
SCEL	2.44E-197	0.29484	0.636	0.368	4.44E-193	Epi-S2
DSC3	9.71E-197	0.29343	0.717	0.459	1.77E-192	Epi-S2
JUP	2.39E-194	0.289658	0.841	0.611	4.35E-190	Epi-S2
ATP5B	5.39E-193	0.278928	0.969	0.902	9.81E-189	Epi-S2
RHOV	2.26E-192	0.276688	0.909	0.566	4.11E-188	Epi-S2
THOC7	7.11E-192	0.298233	0.855	0.673	1.29E-187	Epi-S2
KRT18	1.69E-191	0.315566	0.93	0.69	3.07E-187	Epi-S2
MPZL2	5.51E-191	0.305439	0.886	0.629	1.00E-186	Epi-S2
EMP2	1.54E-188	0.303508	0.858	0.67	2.81E-184	Epi-S2
XRCC6BP1	3.16E-188	0.281287	0.566	0.315	5.75E-184	Epi-S2
SERPINB5	6.67E-188	0.362838	0.902	0.673	1.22E-183	Epi-S2
TMEM19	1.50E-187	0.283253	0.514	0.274	2.73E-183	Epi-S2
RNF39	7.98E-185	0.346495	0.651	0.401	1.45E-180	Epi-S2
TOM1L1	1.59E-181	0.308241	0.478	0.253	2.89E-177	Epi-S2
ATP5F1	7.47E-181	0.272496	0.938	0.81	1.36E-176	Epi-S2
SOD1	1.19E-179	0.275948	0.967	0.881	2.17E-175	Epi-S2
ANP32A	3.08E-179	0.290946	0.735	0.511	5.62E-175	Epi-S2
SYNGR2	5.07E-179	0.300394	0.803	0.586	9.23E-175	Epi-S2
TIMM13	2.43E-178	0.282092	0.904	0.743	4.42E-174	Epi-S2
PSMC5	3.63E-177	0.280529	0.9	0.766	6.61E-173	Epi-S2
EPHX2	1.07E-176	0.280567	0.518	0.291	1.94E-172	Epi-S2

HM13	4.19E-176	0.274747	0.765	0.541	7.62E-172	Epi-S2
SLC25A3	5.92E-176	0.271982	0.965	0.895	1.08E-171	Epi-S2
YPEL5	2.76E-175	0.321971	0.956	0.856	5.03E-171	Epi-S2
THOC3	5.31E-175	0.295454	0.666	0.424	9.67E-171	Epi-S2
NDUFB6	3.66E-174	0.279913	0.898	0.753	6.66E-170	Epi-S2
KRT5	4.86E-174	0.294706	1	0.848	8.85E-170	Epi-S2
PPP1CA	1.49E-173	0.268568	0.916	0.747	2.71E-169	Epi-S2
ATP6V1E1	3.29E-173	0.285073	0.867	0.701	6.00E-169	Epi-S2
TMPRSS4	1.18E-171	0.273572	0.615	0.376	2.15E-167	Epi-S2
SSBP1	3.04E-171	0.266002	0.914	0.773	5.54E-167	Epi-S2
ID1	1.17E-170	0.262103	0.998	0.948	2.12E-166	Epi-S2
MRPL3	1.86E-169	0.289422	0.719	0.515	3.39E-165	Epi-S2
DYNLRB1	9.51E-169	0.268357	0.915	0.789	1.73E-164	Epi-S2
NIPSNAP1	1.10E-165	0.255115	0.46	0.244	2.00E-161	Epi-S2
PAIP2	3.18E-164	0.280237	0.879	0.744	5.79E-160	Epi-S2
FAM162A	1.32E-160	0.266614	0.864	0.716	2.41E-156	Epi-S2
HSBP1L1	4.86E-160	0.279328	0.699	0.473	8.84E-156	Epi-S2
GPX2	1.17E-159	0.338084	0.569	0.336	2.13E-155	Epi-S2
PDHB	5.44E-159	0.268299	0.711	0.5	9.90E-155	Epi-S2
AP1S1	7.50E-159	0.281918	0.704	0.491	1.37E-154	Epi-S2
MRPL20	1.30E-158	0.275	0.871	0.719	2.37E-154	Epi-S2
YWHAZ	3.29E-158	0.252919	0.927	0.817	6.00E-154	Epi-S2
NDUFA2	4.37E-158	0.266039	0.885	0.746	7.96E-154	Epi-S2
TXNL4A	6.67E-158	0.279938	0.688	0.492	1.21E-153	Epi-S2
UBAC1	8.97E-157	0.268361	0.586	0.369	1.63E-152	Epi-S2
SLC26A2	4.84E-156	0.264966	0.475	0.258	8.82E-152	Epi-S2
PKP3	1.99E-154	0.259838	0.715	0.475	3.62E-150	Epi-S2
RNF149	5.41E-154	0.264713	0.604	0.379	9.85E-150	Epi-S2
REEP4	9.15E-154	0.258666	0.576	0.351	1.67E-149	Epi-S2
UQCRC1	1.51E-153	0.270165	0.875	0.721	2.74E-149	Epi-S2
HSD17B8	2.85E-152	0.262906	0.61	0.392	5.20E-148	Epi-S2
LSMD1	3.62E-152	0.267283	0.911	0.785	6.60E-148	Epi-S2
WDR61	7.50E-151	0.26829	0.774	0.581	1.36E-146	Epi-S2
CLEC7A	8.14E-151	0.25154	0.518	0.297	1.48E-146	Epi-S2
VPS28	4.12E-150	0.271054	0.819	0.64	7.50E-146	Epi-S2
TUBB6	1.52E-149	0.310279	0.681	0.479	2.76E-145	Epi-S2
BTF3L4	2.25E-149	0.272609	0.776	0.597	4.10E-145	Epi-S2
PGD	9.60E-149	0.261645	0.75	0.536	1.75E-144	Epi-S2
CAPN1	3.02E-148	0.257848	0.843	0.655	5.50E-144	Epi-S2
COPS8	1.34E-144	0.266739	0.812	0.632	2.44E-140	Epi-S2
GJB5	1.64E-144	0.263778	0.723	0.493	2.99E-140	Epi-S2
MRPL52	3.79E-144	0.259669	0.814	0.645	6.90E-140	Epi-S2
IFITM10	2.72E-143	0.257529	0.454	0.248	4.95E-139	Epi-S2
DNAJB2	1.15E-142	0.267776	0.643	0.44	2.10E-138	Epi-S2
ATP5A1	3.39E-142	0.263655	0.912	0.805	6.17E-138	Epi-S2
MT1H	1.02E-141	0.278224	0.352	0.164	1.85E-137	Epi-S2
NAPRT1	1.70E-141	0.25651	0.654	0.446	3.10E-137	Epi-S2
TNFSF10	7.27E-141	0.250343	0.836	0.6	1.32E-136	Epi-S2

POLE4	2.69E-140	0.272047	0.621	0.419	4.91E-136	Epi-S2
PFN2	2.11E-139	0.251638	0.806	0.614	3.84E-135	Epi-S2
HIGD1A	1.57E-138	0.258471	0.794	0.616	2.85E-134	Epi-S2
CCND1	3.62E-138	0.255865	0.822	0.62	6.59E-134	Epi-S2
TIMM17A	2.27E-136	0.25286	0.782	0.603	4.14E-132	Epi-S2
PYGL	1.99E-135	0.256329	0.649	0.437	3.63E-131	Epi-S2
ZFYVE21	5.50E-134	0.258001	0.788	0.62	1.00E-129	Epi-S2
MTCH2	3.08E-127	0.250701	0.635	0.445	5.61E-123	Epi-S2
DUSP23	8.21E-126	0.259893	0.9	0.773	1.50E-121	Epi-S2
SDC4	8.96E-120	0.376756	0.754	0.572	1.63E-115	Epi-S2
MT1G	4.93E-117	0.326559	0.432	0.244	8.98E-113	Epi-S2
CDC42	4.01E-99	0.265623	0.873	0.753	7.31E-95	Epi-S2
RND3	5.28E-71	0.275813	0.728	0.588	9.61E-67	Epi-S2
DCN	0	5.261803	1	0.696	0	Stro
APOD	0	5.061727	1	0.589	0	Stro
SAA1	0	4.493617	0.883	0.248	0	Stro
PTGDS	0	4.465475	0.996	0.343	0	Stro
LUM	0	4.433742	1	0.235	0	Stro
KERA	0	4.126191	0.992	0.091	0	Stro
ANGPTL7	0	4.027056	0.993	0.473	0	Stro
SOD3	0	3.272922	0.998	0.162	0	Stro
VIM	0	3.101935	1	0.212	0	Stro
ITGBL1	0	3.016256	0.996	0.046	0	Stro
LGALS1	0	2.661328	1	0.208	0	Stro
PLAC9	0	2.619341	0.986	0.076	0	Stro
MMP3	0	2.507459	0.476	0.025	0	Stro
SERPING1	0	2.461318	0.995	0.104	0	Stro
SERPINF1	0	2.410457	0.998	0.441	0	Stro
AQP1	0	2.385669	0.981	0.042	0	Stro
CFH	0	2.317969	0.938	0.029	0	Stro
NNMT	0	2.309553	0.972	0.028	0	Stro
TSC22D1	0	2.287772	0.99	0.515	0	Stro
GRP	0	2.200506	0.88	0.021	0	Stro
COL12A1	0	2.19659	0.969	0.054	0	Stro
SDC2	0	2.189829	0.981	0.028	0	Stro
IFITM3	0	2.129328	1	0.514	0	Stro
RARRES1	0	1.987473	0.901	0.073	0	Stro
THBS4	0	1.985573	0.941	0.015	0	Stro
IFITM1	0	1.984277	0.964	0.216	0	Stro
PMP22	0	1.942263	0.974	0.042	0	Stro
TIMP3	0	1.924118	0.926	0.244	0	Stro
HTRA1	0	1.910974	0.959	0.269	0	Stro
CYTL1	0	1.909616	0.595	0.024	0	Stro
OLFML3	0	1.896219	0.95	0.027	0	Stro
MT2A	0	1.885386	0.999	0.884	0	Stro
IGFBP6	0	1.873543	0.999	0.815	0	Stro
TIMP1	0	1.848038	0.996	0.578	0	Stro
COL6A1	0	1.828531	0.953	0.026	0	Stro

CRABP2	0	1.822021	0.945	0.167	0	Stro
CST3	0	1.789627	1	0.991	0	Stro
SELM	0	1.769246	0.984	0.228	0	Stro
C1S	0	1.735681	0.922	0.027	0	Stro
SPARCL1	0	1.732347	0.884	0.159	0	Stro
CTGF	0	1.72153	0.8	0.094	0	Stro
SEPP1	0	1.71248	0.97	0.38	0	Stro
GNG11	0	1.69266	0.948	0.069	0	Stro
ECM1	0	1.68568	0.978	0.405	0	Stro
CXCL1	0	1.671857	0.361	0.092	0	Stro
FMOD	0	1.641864	0.843	0.016	0	Stro
IFITM2	0	1.637647	0.931	0.054	0	Stro
CXCL2	0	1.622186	0.689	0.154	0	Stro
C1R	0	1.601823	0.866	0.058	0	Stro
ANGPTL5	0	1.595967	0.88	0.007	0	Stro
COL1A2	0	1.593246	0.89	0.028	0	Stro
MGP	0	1.587745	0.768	0.031	0	Stro
TSC22D3	0	1.554278	0.988	0.802	0	Stro
EMP3	0	1.553625	0.966	0.157	0	Stro
MYOC	0	1.537255	0.578	0.013	0	Stro
CCL2	0	1.523184	0.594	0.013	0	Stro
F13A1	0	1.513697	0.835	0.008	0	Stro
GLT8D2	0	1.509901	0.903	0.009	0	Stro
LOX	0	1.493041	0.798	0.043	0	Stro
PPAP2B	0	1.473804	0.898	0.077	0	Stro
FAM3C	0	1.467667	0.935	0.248	0	Stro
FBLN5	0	1.460689	0.839	0.011	0	Stro
COL6A3	0	1.459169	0.877	0.008	0	Stro
CNN3	0	1.44337	0.929	0.26	0	Stro
ANXA5	0	1.431954	0.968	0.318	0	Stro
STEAP4	0	1.426728	0.826	0.021	0	Stro
LOC100506421	0	1.422205	0.815	0.017	0	Stro
CALD1	0	1.411217	0.929	0.175	0	Stro
FIBIN	0	1.409713	0.801	0.01	0	Stro
PRELP	0	1.407074	0.809	0.018	0	Stro
PRRX1	0	1.386009	0.863	0.01	0	Stro
TIMP2	0	1.3696	0.849	0.02	0	Stro
PGRMC1	0	1.364459	0.972	0.588	0	Stro
FTL	0	1.355099	1	0.995	0	Stro
DKK2	0	1.352322	0.854	0.005	0	Stro
C2orf40	0	1.333211	0.677	0.014	0	Stro
CTSF	0	1.329974	0.94	0.284	0	Stro
AKR1C2	0	1.314022	0.785	0.226	0	Stro
IFI6	0	1.311589	0.781	0.101	0	Stro
PDGFRL	0	1.30337	0.822	0.007	0	Stro
PTRF	0	1.298478	0.947	0.367	0	Stro
PRSS23	0	1.255217	0.812	0.123	0	Stro
TCF4	0	1.246746	0.822	0.023	0	Stro

QPCT	0	1.244766	0.815	0.031	0 Stro
LY6E	0	1.23342	0.954	0.47	0 Stro
MAMDC2	0	1.212191	0.814	0.056	0 Stro
RNASE4	0	1.207651	0.888	0.187	0 Stro
ID3	0	1.201012	0.968	0.723	0 Stro
AXL	0	1.182298	0.676	0.034	0 Stro
CPQ	0	1.17894	0.82	0.026	0 Stro
SPOCK1	0	1.176567	0.869	0.244	0 Stro
IL8	0	1.171191	0.383	0.059	0 Stro
TFAP2B	0	1.155708	0.776	0.015	0 Stro
CD99	0	1.155526	0.997	0.881	0 Stro
CCNI	0	1.139251	0.92	0.473	0 Stro
FHL1	0	1.135113	0.761	0.053	0 Stro
PDGFD	0	1.131396	0.794	0.048	0 Stro
CD34	0	1.122946	0.775	0.006	0 Stro
PAM	0	1.121463	0.748	0.047	0 Stro
SERPINE2	0	1.114948	0.747	0.037	0 Stro
HOXB-AS1	0	1.113225	0.693	0.004	0 Stro
IGFBP4	0	1.112119	0.746	0.107	0 Stro
LHFP	0	1.08858	0.825	0.146	0 Stro
SOCS3	0	1.084278	0.707	0.267	0 Stro
AKR1C1	0	1.083589	0.682	0.145	0 Stro
AEBP1	0	1.081855	0.75	0.01	0 Stro
EFHD1	0	1.08116	0.689	0.011	0 Stro
CYBRD1	0	1.077814	0.856	0.214	0 Stro
MT1M	0	1.059006	0.711	0.13	0 Stro
CLDN11	0	1.049512	0.582	0.006	0 Stro
MFGE8	0	1.045453	0.834	0.234	0 Stro
LARP6	0	1.044199	0.752	0.03	0 Stro
MAFB	0	1.039212	0.937	0.555	0 Stro
MMP2	0	1.024664	0.709	0.006	0 Stro
DAB2	0	1.023157	0.742	0.007	0 Stro
MME	0	1.011902	0.698	0.034	0 Stro
SBDS	0	0.999314	0.972	0.714	0 Stro
PITX2	0	0.995666	0.708	0.007	0 Stro
ASIP	0	0.99206	0.6	0.006	0 Stro
TUBA1A	0	0.991806	0.929	0.671	0 Stro
NDNF	0	0.988638	0.692	0.017	0 Stro
WBP5	0	0.982109	0.887	0.41	0 Stro
HSD11B1	0	0.977998	0.661	0.002	0 Stro
FXYD1	0	0.969635	0.668	0.014	0 Stro
GAS6	0	0.967836	0.759	0.17	0 Stro
TMEM204	0	0.967827	0.699	0.059	0 Stro
MFAP4	0	0.967207	0.652	0.006	0 Stro
LSAMP	0	0.963555	0.674	0.008	0 Stro
NUCB2	0	0.963072	0.811	0.252	0 Stro
TNFRSF11B	0	0.961262	0.579	0.003	0 Stro
B3GNT7	0	0.957562	0.505	0.027	0 Stro

RGCC	0	0.957453	0.546	0.041	0	Stro
LOC541471	0	0.9442	0.701	0.072	0	Stro
PCOLCE	0	0.943083	0.649	0.046	0	Stro
SMIM3	0	0.928059	0.729	0.088	0	Stro
LINC00152	0	0.92494	0.658	0.015	0	Stro
IFI27	0	0.922044	0.918	0.461	0	Stro
SEPT11	0	0.918669	0.682	0.034	0	Stro
JAM3	0	0.909508	0.675	0.035	0	Stro
CD59	0	0.900244	0.956	0.695	0	Stro
SDCBP	0	0.899343	0.916	0.502	0	Stro
PTGS2	0	0.893431	0.546	0.046	0	Stro
KDR	0	0.890961	0.602	0.004	0	Stro
COL8A1	0	0.890435	0.589	0.009	0	Stro
TWIST2	0	0.890101	0.654	0.005	0	Stro
SBDSP1	0	0.888681	0.934	0.589	0	Stro
NTN4	0	0.880939	0.628	0.007	0	Stro
MXRA8	0	0.878288	0.663	0.006	0	Stro
COLGALT2	0	0.877747	0.632	0.004	0	Stro
COL6A2	0	0.876948	0.656	0.005	0	Stro
COLEC12	0	0.868102	0.694	0.069	0	Stro
FXYD5	0	0.861098	0.699	0.059	0	Stro
GLUL	0	0.860305	1	0.947	0	Stro
FGFR1	0	0.855053	0.68	0.028	0	Stro
DPYSL2	0	0.850691	0.645	0.043	0	Stro
ACSL3	0	0.846352	0.769	0.268	0	Stro
PRKCDBP	0	0.843005	0.744	0.174	0	Stro
ABI3BP	0	0.841216	0.604	0.046	0	Stro
CD63	0	0.839619	0.997	0.977	0	Stro
ALDH3A1	0	0.83579	1	0.973	0	Stro
IRS2	0	0.83562	0.678	0.107	0	Stro
SPARC	0	0.835227	0.93	0.368	0	Stro
ARRDC3	0	0.81635	0.653	0.223	0	Stro
CTSK	0	0.809464	0.619	0.023	0	Stro
C1QTNF3	0	0.809171	0.473	0.008	0	Stro
GGT5	0	0.806618	0.528	0.005	0	Stro
GSTM3	0	0.800725	0.71	0.146	0	Stro
THBS1	0	0.794393	0.611	0.099	0	Stro
CEBPD	0	0.793532	0.825	0.562	0	Stro
PCOLCE2	0	0.781054	0.541	0.038	0	Stro
ID2	0	0.780884	0.852	0.507	0	Stro
SLC2A3	0	0.780516	0.564	0.026	0	Stro
LAPTM4A	0	0.77428	0.998	0.966	0	Stro
SUN2	0	0.773436	0.727	0.24	0	Stro
FSTL1	0	0.76922	0.583	0.052	0	Stro
PLSCR4	0	0.769038	0.602	0.016	0	Stro
ARPC5	0	0.766546	0.77	0.281	0	Stro
VMO1	0	0.766421	0.387	0.032	0	Stro
WASF2	0	0.763578	0.764	0.307	0	Stro

UBL3	0	0.754353	0.702	0.236	0	Stro
MAFF	0	0.751442	0.778	0.379	0	Stro
APLP2	0	0.750057	0.965	0.793	0	Stro
PSAP	0	0.749464	0.997	0.946	0	Stro
MT1E	0	0.74886	0.881	0.607	0	Stro
COL5A2	0	0.744953	0.564	0.027	0	Stro
ZFP36L2	0	0.737201	0.845	0.465	0	Stro
FKBP9	0	0.736059	0.571	0.084	0	Stro
RGS16	0	0.735956	0.468	0.131	0	Stro
CCK	0	0.732662	0.47	0.056	0	Stro
PIGT	0	0.724329	0.759	0.335	0	Stro
ARF4	0	0.722875	0.962	0.804	0	Stro
AK057379	0	0.721277	0.539	0.017	0	Stro
PLD3	0	0.715654	0.856	0.492	0	Stro
CRIP2	0	0.711597	0.662	0.14	0	Stro
XBP1	0	0.707324	0.793	0.452	0	Stro
RCN3	0	0.705096	0.566	0.009	0	Stro
ZFHX4	0	0.701958	0.544	0.007	0	Stro
FBLN2	0	0.694961	0.542	0.066	0	Stro
EIF5	0	0.690084	0.902	0.688	0	Stro
ARHGAP29	0	0.689139	0.569	0.04	0	Stro
C7orf10	0	0.688958	0.662	0.231	0	Stro
AHR	0	0.688627	0.609	0.134	0	Stro
BGN	0	0.68621	0.523	0.187	0	Stro
TMEM55A	0	0.683033	0.576	0.06	0	Stro
DSTN	0	0.680598	0.986	0.926	0	Stro
TRAM1	0	0.666719	0.762	0.386	0	Stro
AKR1B1	0	0.666457	0.821	0.404	0	Stro
SVIL	0	0.666329	0.649	0.22	0	Stro
FKBP10	0	0.665933	0.544	0.012	0	Stro
CYR61	0	0.663731	0.521	0.052	0	Stro
WNT5A	0	0.657189	0.523	0.031	0	Stro
TMEM106C	0	0.656138	0.65	0.199	0	Stro
COX7A1	0	0.655855	0.878	0.413	0	Stro
HPGD	0	0.654308	0.472	0.116	0	Stro
CTNNAL1	0	0.650835	0.625	0.157	0	Stro
AMD1	0	0.649976	0.714	0.382	0	Stro
ITSN1	0	0.649178	0.568	0.086	0	Stro
ZBTB20	0	0.645292	0.573	0.077	0	Stro
ZFHX3	0	0.643019	0.553	0.064	0	Stro
ACTN1	0	0.635706	0.569	0.098	0	Stro
TSPAN4	0	0.634968	0.638	0.187	0	Stro
APP	0	0.634256	0.868	0.514	0	Stro
XG	0	0.631817	0.621	0.192	0	Stro
ABCA6	0	0.631304	0.49	0.003	0	Stro
CDC42EP2	0	0.63049	0.474	0.018	0	Stro
C19orf10	0	0.629701	0.838	0.554	0	Stro
EMX2	0	0.627816	0.66	0.202	0	Stro

DPP7	0	0.627702	0.775	0.422	0	Stro
ENPP1	0	0.627105	0.453	0.004	0	Stro
SNAI1	0	0.626013	0.49	0.073	0	Stro
TIPARP	0	0.618975	0.654	0.329	0	Stro
DAP	0	0.616559	0.778	0.431	0	Stro
SSR4	0	0.616171	0.954	0.885	0	Stro
LOC100505806	0	0.615827	0.69	0.29	0	Stro
PDLIM2	0	0.614044	0.583	0.135	0	Stro
VAMP5	0	0.613247	0.542	0.061	0	Stro
PARVA	0	0.612096	0.568	0.104	0	Stro
MAP1B	0	0.610503	0.455	0.005	0	Stro
SERPINI1	0	0.607887	0.524	0.069	0	Stro
CDC42EP3	0	0.60757	0.532	0.069	0	Stro
APCDD1	0	0.605674	0.442	0.028	0	Stro
RTN4	0	0.604401	0.972	0.882	0	Stro
CTSL1	0	0.60388	0.778	0.444	0	Stro
C1QTNF7	0	0.5978	0.423	0.003	0	Stro
FN1	0	0.595496	0.365	0.006	0	Stro
CALU	0	0.592912	0.643	0.248	0	Stro
SERPINA3	0	0.592287	0.3	0.003	0	Stro
ANXA1	0	0.591452	0.999	0.981	0	Stro
TFPI	0	0.590008	0.456	0.004	0	Stro
CHEK2	0	0.586033	0.485	0.048	0	Stro
CRNDE	0	0.585889	0.726	0.289	0	Stro
PLOD1	0	0.585528	0.529	0.094	0	Stro
RRBP1	0	0.5823	0.669	0.29	0	Stro
FKBP7	0	0.580821	0.51	0.046	0	Stro
SEC63	0	0.580291	0.768	0.442	0	Stro
TWIST1	0	0.57672	0.404	0.002	0	Stro
TCN2	0	0.571166	0.468	0.043	0	Stro
ZEB1	0	0.567484	0.448	0.004	0	Stro
ITGB1	0	0.563661	0.854	0.558	0	Stro
DPYSL3	0	0.561265	0.475	0.032	0	Stro
PHLDA1	0	0.56027	0.537	0.148	0	Stro
FOXO3	0	0.558632	0.523	0.081	0	Stro
LIMA1	0	0.557199	0.628	0.278	0	Stro
GTF3C5	0	0.551342	0.614	0.271	0	Stro
SEMA5A	0	0.551105	0.409	0.02	0	Stro
ITGB1BP1	0	0.545037	0.688	0.369	0	Stro
CLEC11A	0	0.544711	0.583	0.211	0	Stro
ICAM1	0	0.542071	0.391	0.015	0	Stro
ANGPTL2	0	0.540315	0.451	0.042	0	Stro
SNRPN	0	0.539484	0.813	0.543	0	Stro
HOXB2	0	0.536689	0.397	0.002	0	Stro
SEC62	0	0.536289	0.952	0.826	0	Stro
PDK4	0	0.536123	0.355	0.01	0	Stro
MSRB3	0	0.535395	0.434	0.008	0	Stro
EID1	0	0.535304	0.974	0.867	0	Stro

PDIA3	0	0.532665	0.938	0.803	0	Stro
OLFML1	0	0.531163	0.37	0.002	0	Stro
A1BG	0	0.530965	0.447	0.013	0	Stro
PTMA	0	0.529733	1	0.995	0	Stro
KCTD12	0	0.529266	0.427	0.02	0	Stro
EEF2	0	0.528244	0.964	0.858	0	Stro
ALCAM	0	0.527832	0.439	0.036	0	Stro
LRP1	0	0.526555	0.56	0.199	0	Stro
C1orf21	0	0.526514	0.673	0.344	0	Stro
TCF12	0	0.523766	0.504	0.098	0	Stro
HSP90B1	0	0.522851	0.958	0.8	0	Stro
DNAJB9	0	0.522687	0.687	0.369	0	Stro
BCAP29	0	0.521966	0.569	0.215	0	Stro
LRPAP1	0	0.521584	0.859	0.655	0	Stro
F2R	0	0.521554	0.367	0.03	0	Stro
VASN	0	0.521064	0.414	0.045	0	Stro
MAGED2	0	0.516984	0.692	0.376	0	Stro
HSD17B12	0	0.51543	0.633	0.292	0	Stro
SEPT7	0	0.515288	0.918	0.749	0	Stro
VKORC1	0	0.513482	0.674	0.364	0	Stro
RAB32	0	0.513082	0.496	0.102	0	Stro
SERPINB6	0	0.512144	0.59	0.211	0	Stro
HSD17B11	0	0.510908	0.484	0.051	0	Stro
ANPEP	0	0.50939	0.399	0.003	0	Stro
GPX8	0	0.508826	0.493	0.099	0	Stro
CTSZ	0	0.508312	0.486	0.077	0	Stro
CD163L1	0	0.507205	0.419	0.002	0	Stro
PLA2G5	0	0.504384	0.366	0.003	0	Stro
EMCN	0	0.504301	0.391	0.003	0	Stro
IFI27L2	0	0.503325	0.679	0.322	0	Stro
SFRP1	0	0.501787	0.345	0.009	0	Stro
COL8A2	0	0.501537	0.413	0.004	0	Stro
ACYP1	0	0.499995	0.626	0.302	0	Stro
RHOBTB3	0	0.499575	0.527	0.154	0	Stro
QSOX1	0	0.49801	0.469	0.075	0	Stro
SNHG6	0	0.497678	0.901	0.735	0	Stro
HEXB	0	0.496365	0.693	0.384	0	Stro
LOC284998	0	0.496184	0.37	0.002	0	Stro
INMT	0	0.495967	0.303	0.005	0	Stro
LOXL1	0	0.49592	0.416	0.048	0	Stro
MYL9	0	0.495391	0.425	0.029	0	Stro
AMOTL2	0	0.493249	0.432	0.076	0	Stro
SERTAD4-AS1	0	0.492682	0.495	0.126	0	Stro
DNAJC3	0	0.489069	0.558	0.232	0	Stro
AK4	0	0.488846	0.46	0.07	0	Stro
RAB11FIP2	0	0.488778	0.423	0.057	0	Stro
CCDC151	0	0.487855	0.406	0.02	0	Stro
WFDC1	0	0.487618	0.368	0.002	0	Stro

C14orf132	0	0.485567	0.429	0.033	0	Stro
AKAP13	0	0.48463	0.498	0.123	0	Stro
LAMB2	0	0.481684	0.453	0.092	0	Stro
SEMA3B	0	0.481171	0.411	0.029	0	Stro
ITGB4	0	0.480731	0.499	0.17	0	Stro
HIBADH	0	0.475383	0.574	0.243	0	Stro
GLT8D1	0	0.474553	0.548	0.224	0	Stro
EDNRB	0	0.472991	0.39	0.065	0	Stro
IL32	0	0.468359	0.364	0.024	0	Stro
TCEAL3	0	0.466154	0.489	0.139	0	Stro
FAM126A	0	0.46575	0.398	0.028	0	Stro
CHPT1	0	0.465501	0.405	0.024	0	Stro
CERS2	0	0.46518	0.546	0.201	0	Stro
MGAT1	0	0.463107	0.593	0.267	0	Stro
COL1A1	0	0.460908	0.354	0.012	0	Stro
SLC26A4	0	0.459917	0.322	0.005	0	Stro
TMEM45A	0	0.459516	0.469	0.11	0	Stro
FAM229B	0	0.458485	0.472	0.109	0	Stro
HLA-E	0	0.454788	0.957	0.872	0	Stro
SAA2	0	0.451555	0.261	0.003	0	Stro
MAGI2-AS3	0	0.449531	0.391	0.018	0	Stro
PLOD2	0	0.448966	0.378	0.023	0	Stro
IL6ST	0	0.444468	0.46	0.113	0	Stro
NPC2	0	0.44025	0.955	0.854	0	Stro
NT5E	0	0.436656	0.369	0.007	0	Stro
F10	0	0.435533	0.337	0.002	0	Stro
NANOS1	0	0.434427	0.35	0.006	0	Stro
PDLIM4	0	0.434114	0.553	0.202	0	Stro
CXCL3	0	0.432901	0.292	0.051	0	Stro
SLC39A13	0	0.428399	0.435	0.087	0	Stro
TMEM98	0	0.427999	0.462	0.116	0	Stro
CHST6	0	0.427951	0.341	0.006	0	Stro
TMEM59	0	0.426355	0.988	0.95	0	Stro
ARMCX3	0	0.425338	0.415	0.06	0	Stro
ALDH1A3	0	0.424998	0.366	0.064	0	Stro
CDKN1C	0	0.424016	0.449	0.11	0	Stro
TMEM64	0	0.42377	0.377	0.022	0	Stro
SYNE1	0	0.418725	0.395	0.072	0	Stro
P4HA2	0	0.418712	0.503	0.177	0	Stro
PGM2L1	0	0.418511	0.387	0.053	0	Stro
ANKH	0	0.415984	0.415	0.095	0	Stro
SUSD2	0	0.415148	0.325	0.004	0	Stro
FBLN7	0	0.41216	0.342	0.005	0	Stro
ZBTB16	0	0.407778	0.357	0.028	0	Stro
GPC4	0	0.406841	0.345	0.019	0	Stro
MSN	0	0.406721	0.392	0.051	0	Stro
LSP1	0	0.404947	0.703	0.263	0	Stro
CYB5R2	0	0.404163	0.353	0.069	0	Stro

PLIN2	0	0.403281	0.395	0.105	0	Stro
LDHA	0	0.402992	0.999	0.989	0	Stro
C1orf198	0	0.402987	0.363	0.047	0	Stro
CHN1	0	0.402018	0.299	0.002	0	Stro
CHSY1	0	0.401969	0.357	0.036	0	Stro
DCLK1	0	0.400362	0.279	0.005	0	Stro
ADAM33	0	0.400244	0.328	0.004	0	Stro
RECK	0	0.399994	0.336	0.007	0	Stro
ISLR	0	0.398994	0.308	0.006	0	Stro
SEC22C	0	0.397277	0.44	0.115	0	Stro
KDELR3	0	0.395454	0.328	0.009	0	Stro
CHST7	0	0.394067	0.335	0.026	0	Stro
AK125699	0	0.393835	0.335	0.015	0	Stro
CMTM3	0	0.391222	0.361	0.039	0	Stro
NR3C1	0	0.390616	0.422	0.118	0	Stro
HGF	0	0.388816	0.288	0.003	0	Stro
CFL2	0	0.388808	0.369	0.051	0	Stro
FERMT2	0	0.384627	0.312	0.005	0	Stro
IL13RA1	0	0.383904	0.387	0.077	0	Stro
NTRK3	0	0.383259	0.314	0.002	0	Stro
MXRA5	0	0.382583	0.288	0.034	0	Stro
ANG	0	0.376882	0.415	0.115	0	Stro
SLC9A3R2	0	0.376801	0.343	0.033	0	Stro
EPB41L2	0	0.375814	0.33	0.021	0	Stro
SLC22A4	0	0.37579	0.323	0.011	0	Stro
FLNA	0	0.375626	0.397	0.102	0	Stro
CCDC107	0	0.373319	0.415	0.116	0	Stro
ZNF106	0	0.370837	0.384	0.077	0	Stro
SYBU	0	0.370664	0.307	0.01	0	Stro
PFKFB3	0	0.370328	0.331	0.073	0	Stro
CRY1	0	0.369783	0.333	0.068	0	Stro
MAGEH1	0	0.368605	0.389	0.083	0	Stro
C4orf48	0	0.367945	0.404	0.111	0	Stro
VOPP1	0	0.364095	0.367	0.067	0	Stro
NFIX	0	0.363767	0.375	0.089	0	Stro
C12orf23	0	0.362868	0.36	0.073	0	Stro
ADAMTS1	0	0.362058	0.273	0.013	0	Stro
SLC44A1	0	0.358926	0.357	0.068	0	Stro
SRPX2	0	0.357931	0.293	0.023	0	Stro
CREB3L2	0	0.354529	0.393	0.11	0	Stro
IL11RA	0	0.3542	0.308	0.025	0	Stro
MGLL	0	0.353273	0.374	0.075	0	Stro
SASH1	0	0.350898	0.386	0.103	0	Stro
G0S2	0	0.350373	0.341	0.029	0	Stro
LRIG1	0	0.349729	0.329	0.056	0	Stro
CFB	0	0.349562	0.325	0.07	0	Stro
EFEMP2	0	0.348787	0.324	0.032	0	Stro
GYPC	0	0.347228	0.325	0.018	0	Stro

TWSG1	0	0.346716	0.348	0.065	0	Stro
CD97	0	0.346657	0.32	0.035	0	Stro
GRK5	0	0.342114	0.291	0.004	0	Stro
GSTM5	0	0.341758	0.28	0.002	0	Stro
LY96	0	0.341319	0.295	0.01	0	Stro
WBP1L	0	0.338992	0.346	0.076	0	Stro
ITGB5	0	0.3384	0.321	0.066	0	Stro
COPZ2	0	0.337764	0.34	0.074	0	Stro
HEG1	0	0.337707	0.292	0.018	0	Stro
MAP1A	0	0.337178	0.274	0.005	0	Stro
NLGN4X	0	0.337084	0.306	0.03	0	Stro
SESN3	0	0.336427	0.31	0.034	0	Stro
SLC39A14	0	0.336189	0.305	0.05	0	Stro
PDGFRA	0	0.335669	0.277	0.006	0	Stro
NEBL	0	0.334948	0.353	0.08	0	Stro
GALNT6	0	0.334948	0.292	0.019	0	Stro
CDC42BPA	0	0.334649	0.32	0.047	0	Stro
CDON	0	0.331753	0.273	0.009	0	Stro
TPM2	0	0.33056	0.303	0.018	0	Stro
MRGPRF	0	0.330486	0.274	0.003	0	Stro
GOLIM4	0	0.326337	0.314	0.062	0	Stro
ERG	0	0.322533	0.272	0.003	0	Stro
TP53I11	0	0.322011	0.289	0.024	0	Stro
SEPT4	0	0.316276	0.267	0.008	0	Stro
CD320	0	0.313048	0.309	0.051	0	Stro
ARMCX1	0	0.312917	0.298	0.036	0	Stro
IRX1	0	0.311233	0.265	0.001	0	Stro
B3GNT1	0	0.311117	0.323	0.068	0	Stro
PLCD3	0	0.309447	0.3	0.053	0	Stro
COL4A3	0	0.308037	0.27	0.01	0	Stro
AXIN2	0	0.307966	0.256	0.006	0	Stro
LURAP1L	0	0.306766	0.255	0.008	0	Stro
FAM65B	0	0.306318	0.262	0.004	0	Stro
ADAMTS12	0	0.304064	0.253	0.002	0	Stro
BMP4	0	0.303982	0.264	0.013	0	Stro
AP1S2	0	0.30254	0.331	0.075	0	Stro
DIO2	0	0.301981	0.253	0.041	0	Stro
ANXA6	0	0.301618	0.274	0.007	0	Stro
SEC23A	0	0.301429	0.306	0.058	0	Stro
P4HA1	0	0.299887	0.3	0.057	0	Stro
ST3GAL5	0	0.297561	0.269	0.012	0	Stro
RBMS3	0	0.296488	0.253	0.008	0	Stro
LOC728392	0	0.293594	0.252	0.031	0	Stro
NCAM1	0	0.293114	0.251	0.011	0	Stro
NAAA	0	0.276465	0.283	0.052	0	Stro
TGFBR3	0	0.261219	0.251	0.025	0	Stro
TULP3	2.19E-301	0.332157	0.355	0.093	3.98E-297	Stro
COX11	4.79E-301	0.50097	0.63	0.327	8.72E-297	Stro

NDN	1.40E-300	0.450119	0.607	0.287	2.55E-296	Stro
RAB34	4.09E-299	0.468945	0.696	0.39	7.44E-295	Stro
IRX2	1.24E-298	0.282622	0.287	0.058	2.26E-294	Stro
KLF9	4.02E-297	0.429874	0.509	0.194	7.32E-293	Stro
MT1A	1.71E-291	0.442873	0.279	0.057	3.11E-287	Stro
TMED2	9.65E-291	0.470597	0.906	0.761	1.76E-286	Stro
ETV5	9.87E-288	0.268705	0.27	0.053	1.80E-283	Stro
BC016015	3.31E-285	0.334885	0.334	0.084	6.03E-281	Stro
ABL2	1.32E-284	0.302803	0.286	0.061	2.41E-280	Stro
ADD3	4.67E-284	0.374925	0.428	0.142	8.51E-280	Stro
CPXM2	1.57E-282	0.521308	0.703	0.396	2.86E-278	Stro
IGFBP2	1.26E-281	0.677868	0.766	0.518	2.29E-277	Stro
TMEM9	1.48E-280	0.46669	0.631	0.335	2.69E-276	Stro
SOD2	3.85E-280	0.772157	0.57	0.277	7.02E-276	Stro
ZFAND5	1.54E-279	0.555055	0.85	0.631	2.80E-275	Stro
KIAA1033	1.61E-276	0.268314	0.281	0.06	2.93E-272	Stro
SGCB	1.31E-275	0.274177	0.288	0.064	2.38E-271	Stro
INSIG2	2.21E-274	0.339665	0.374	0.112	4.03E-270	Stro
REXO2	3.74E-274	0.491321	0.804	0.589	6.80E-270	Stro
NENF	5.78E-274	0.460297	0.854	0.687	1.05E-269	Stro
CANX	4.22E-273	0.487448	0.803	0.574	7.68E-269	Stro
RECQL	4.15E-272	0.313897	0.338	0.09	7.56E-268	Stro
RP9P	4.54E-270	0.272643	0.255	0.05	8.26E-266	Stro
ERRFI1	6.40E-270	0.518098	0.557	0.254	1.17E-265	Stro
SLC20A1	4.26E-267	0.482775	0.697	0.382	7.76E-263	Stro
HSPA13	2.77E-266	0.250672	0.264	0.055	5.05E-262	Stro
SYNC	7.73E-266	0.337408	0.419	0.142	1.41E-261	Stro
MID1IP1	3.71E-265	0.471621	0.561	0.27	6.76E-261	Stro
AKR1C3	4.93E-265	0.32775	0.319	0.081	8.97E-261	Stro
SEC61G	8.39E-263	0.402052	0.965	0.896	1.53E-258	Stro
EEF1A1	1.46E-262	0.292258	1	0.999	2.65E-258	Stro
METTL7A	3.25E-262	0.423935	0.538	0.239	5.91E-258	Stro
DNAJC10	1.07E-259	0.258964	0.269	0.058	1.95E-255	Stro
FNDC3A	1.13E-258	0.279705	0.311	0.08	2.06E-254	Stro
POLR2L	1.36E-258	0.366916	0.987	0.962	2.47E-254	Stro
TTC3	2.62E-257	0.420157	0.53	0.237	4.77E-253	Stro
TTC28	1.42E-256	0.378845	0.467	0.186	2.58E-252	Stro
PPAPDC1B	1.25E-255	0.373131	0.433	0.16	2.27E-251	Stro
GLG1	6.52E-254	0.342489	0.39	0.13	1.19E-249	Stro
HSP90AB1	3.60E-252	0.382652	0.988	0.959	6.55E-248	Stro
ERLEC1	2.16E-248	0.42787	0.559	0.273	3.94E-244	Stro
TMCO3	3.00E-248	0.309852	0.349	0.105	5.46E-244	Stro
CDK5RAP2	4.71E-247	0.270273	0.29	0.072	8.57E-243	Stro
STMN1	4.88E-246	0.260124	0.45	0.164	8.89E-242	Stro
OAT	1.02E-245	0.418678	0.633	0.337	1.86E-241	Stro
RBMS1	7.06E-245	0.332291	0.396	0.137	1.29E-240	Stro
OS9	1.30E-244	0.453652	0.804	0.613	2.37E-240	Stro
C16orf45	8.27E-244	0.284562	0.299	0.078	1.51E-239	Stro

C9orf3	5.32E-243	0.410818	0.567	0.281	9.68E-239	Stro
CHPF	2.04E-242	0.438933	0.538	0.26	3.72E-238	Stro
TSPYL2	3.10E-242	0.405163	0.484	0.202	5.64E-238	Stro
PTPRS	3.55E-242	0.403318	0.284	0.071	6.47E-238	Stro
TMBIM1	5.74E-242	0.458235	0.781	0.571	1.05E-237	Stro
PPT1	4.57E-241	0.453986	0.785	0.546	8.33E-237	Stro
B4GALT1	1.04E-240	0.484295	0.327	0.096	1.90E-236	Stro
FCGRT	1.35E-240	0.367561	0.562	0.266	2.45E-236	Stro
CRTAP	3.32E-240	0.37424	0.51	0.227	6.05E-236	Stro
NUDT4	7.06E-238	0.365542	0.435	0.169	1.29E-233	Stro
YBX1	4.15E-237	0.319537	0.995	0.98	7.56E-233	Stro
MBNL1	2.23E-236	0.309252	0.344	0.106	4.06E-232	Stro
C6orf62	3.98E-236	0.319012	0.35	0.111	7.25E-232	Stro
PRDX4	3.33E-235	0.44104	0.668	0.414	6.06E-231	Stro
CCNG2	9.82E-232	0.28952	0.311	0.088	1.79E-227	Stro
CD81	2.26E-230	0.389915	0.459	0.189	4.12E-226	Stro
TACC1	1.92E-226	0.354343	0.449	0.182	3.49E-222	Stro
SERPINH1	2.00E-222	0.327601	0.393	0.142	3.63E-218	Stro
LEPROT	5.09E-222	0.443099	0.701	0.471	9.27E-218	Stro
HSPA8	5.87E-222	0.414543	0.916	0.77	1.07E-217	Stro
NRP2	2.28E-221	0.307043	0.325	0.1	4.15E-217	Stro
RPS27L	2.56E-220	0.432082	0.936	0.829	4.67E-216	Stro
NDUFA4L2	2.91E-217	0.538991	0.869	0.617	5.30E-213	Stro
NGFRAP1	4.49E-216	0.465046	0.886	0.742	8.17E-212	Stro
GFPT1	1.31E-213	0.281104	0.33	0.105	2.38E-209	Stro
SLITRK6	2.00E-213	0.267816	0.252	0.062	3.64E-209	Stro
TMEM43	2.01E-213	0.376346	0.553	0.283	3.65E-209	Stro
RUNX1	1.06E-212	0.299833	0.322	0.101	1.93E-208	Stro
CHCHD10	3.90E-212	0.317951	0.549	0.237	7.11E-208	Stro
PKIG	8.82E-212	0.311158	0.381	0.14	1.61E-207	Stro
PIGK	1.25E-211	0.331041	0.415	0.166	2.28E-207	Stro
TXNDC15	2.10E-211	0.36969	0.541	0.278	3.82E-207	Stro
PELI1	3.00E-211	0.359724	0.456	0.188	5.46E-207	Stro
TMEM50B	8.90E-210	0.410425	0.659	0.411	1.62E-205	Stro
SLC16A3	4.20E-209	0.306379	0.335	0.109	7.64E-205	Stro
OSTC	2.77E-208	0.420136	0.791	0.609	5.04E-204	Stro
ARL2	3.63E-206	0.405184	0.666	0.427	6.60E-202	Stro
EXTL2	4.49E-206	0.252852	0.299	0.089	8.18E-202	Stro
SNHG5	2.18E-204	0.391811	0.975	0.908	3.96E-200	Stro
SSR1	3.52E-203	0.38847	0.619	0.369	6.40E-199	Stro
SSR3	5.05E-203	0.410816	0.69	0.464	9.19E-199	Stro
LOC654342	2.72E-201	0.354617	0.437	0.189	4.95E-197	Stro
CTNNB1	2.48E-199	0.508084	0.428	0.182	4.51E-195	Stro
AKIRIN2	1.21E-198	0.421849	0.722	0.511	2.20E-194	Stro
MORF4L2	1.60E-195	0.395898	0.896	0.797	2.92E-191	Stro
SRM	5.53E-194	0.327983	0.368	0.14	1.01E-189	Stro
MRC2	2.64E-192	0.279016	0.311	0.101	4.81E-188	Stro
LMAN1	1.37E-190	0.386705	0.615	0.367	2.50E-186	Stro

CD40	1.40E-190	0.259426	0.299	0.094	2.54E-186	Stro
C6orf48	4.44E-190	0.429885	0.802	0.624	8.08E-186	Stro
AK1	8.82E-190	0.352478	0.511	0.262	1.61E-185	Stro
PRKAR1A	2.10E-189	0.39043	0.677	0.438	3.83E-185	Stro
FAM63B	8.25E-189	0.33469	0.508	0.253	1.50E-184	Stro
HERPUD1	1.06E-187	0.397786	0.972	0.937	1.93E-183	Stro
C5orf38	7.85E-187	0.27485	0.335	0.118	1.43E-182	Stro
NFIC	9.31E-186	0.374597	0.614	0.365	1.70E-181	Stro
MCFD2	6.08E-185	0.328515	0.416	0.183	1.11E-180	Stro
EHD1	6.96E-185	0.381212	0.425	0.191	1.27E-180	Stro
SORBS3	9.79E-185	0.299575	0.408	0.173	1.78E-180	Stro
MLEC	1.87E-184	0.33238	0.462	0.221	3.41E-180	Stro
SLC39A1	4.62E-184	0.392178	0.713	0.513	8.42E-180	Stro
STRAP	1.73E-183	0.38347	0.739	0.535	3.16E-179	Stro
GADD45G	3.09E-182	0.533804	0.621	0.374	5.63E-178	Stro
SNHG8	5.34E-182	0.366298	0.844	0.675	9.73E-178	Stro
NFIB	7.33E-182	0.293799	0.355	0.133	1.33E-177	Stro
ARL5A	8.30E-182	0.38879	0.696	0.49	1.51E-177	Stro
GPAA1	1.01E-180	0.353421	0.525	0.284	1.83E-176	Stro
CSDE1	4.79E-180	0.377175	0.871	0.722	8.72E-176	Stro
TMEM30A	1.14E-178	0.284049	0.366	0.143	2.08E-174	Stro
ALKBH5	1.33E-178	0.361097	0.529	0.285	2.42E-174	Stro
TMED10	9.24E-178	0.350366	0.901	0.801	1.68E-173	Stro
FHL2	9.82E-176	0.356903	0.592	0.328	1.79E-171	Stro
SEC61A1	2.55E-174	0.311183	0.425	0.196	4.65E-170	Stro
LGMN	2.60E-174	0.330595	0.48	0.241	4.73E-170	Stro
UBE2J1	1.03E-172	0.356601	0.475	0.243	1.88E-168	Stro
DDX3X	8.75E-172	0.443024	0.767	0.598	1.59E-167	Stro
STOM	1.29E-170	0.364989	0.585	0.349	2.34E-166	Stro
SLC25A33	1.41E-170	0.366065	0.472	0.243	2.56E-166	Stro
USP22	1.83E-169	0.258963	0.298	0.103	3.33E-165	Stro
CMPK1	8.90E-167	0.363833	0.74	0.529	1.62E-162	Stro
CAPZA2	3.64E-165	0.368997	0.77	0.595	6.63E-161	Stro
NFIA	1.22E-164	0.340038	0.49	0.257	2.23E-160	Stro
GNAI1	3.57E-163	0.352569	0.528	0.3	6.50E-159	Stro
PNRC1	4.74E-163	0.384695	0.929	0.857	8.62E-159	Stro
PPFIBP1	5.11E-163	0.34395	0.54	0.305	9.31E-159	Stro
IDS	5.79E-163	0.349577	0.595	0.367	1.05E-158	Stro
PTTG1IP	2.23E-162	0.362509	0.738	0.56	4.06E-158	Stro
SDF4	2.99E-162	0.366969	0.648	0.443	5.44E-158	Stro
S100A10	4.96E-162	0.278035	0.999	0.942	9.03E-158	Stro
MAGED1	5.11E-162	0.294493	0.398	0.179	9.31E-158	Stro
DAD1	7.26E-162	0.318736	0.905	0.827	1.32E-157	Stro
NTAN1	3.26E-161	0.296683	0.439	0.215	5.94E-157	Stro
SPG20	6.53E-161	0.326026	0.442	0.22	1.19E-156	Stro
TMEM120A	7.56E-161	0.295503	0.424	0.203	1.38E-156	Stro
TMX4	8.91E-161	0.30002	0.425	0.199	1.62E-156	Stro
PCNP	4.05E-159	0.360483	0.815	0.66	7.38E-155	Stro

EGR1	4.83E-159	0.45916	0.94	0.853	8.80E-155	Stro
SPTBN1	5.31E-158	0.280605	0.382	0.166	9.66E-154	Stro
PSIP1	5.76E-158	0.304257	0.456	0.227	1.05E-153	Stro
JMJD6	8.79E-157	0.347219	0.488	0.262	1.60E-152	Stro
DEGS1	1.65E-155	0.267099	0.613	0.338	3.00E-151	Stro
CCNL1	6.97E-155	0.382353	0.901	0.789	1.27E-150	Stro
CASC4	1.11E-154	0.26922	0.346	0.144	2.02E-150	Stro
MAP1LC3A	2.01E-153	0.274492	0.381	0.169	3.66E-149	Stro
ATP6AP2	2.30E-152	0.354038	0.852	0.749	4.19E-148	Stro
C12orf57	1.51E-151	0.349112	0.839	0.725	2.74E-147	Stro
CCDC85B	2.00E-151	0.360704	0.723	0.52	3.63E-147	Stro
SMPD1	5.18E-151	0.268952	0.335	0.139	9.43E-147	Stro
C16orf80	1.95E-149	0.382777	0.588	0.384	3.56E-145	Stro
LAMP1	4.57E-149	0.310732	0.892	0.801	8.32E-145	Stro
NFKBIZ	6.67E-149	0.411212	0.53	0.31	1.21E-144	Stro
LMNA	6.83E-149	0.354653	0.978	0.965	1.24E-144	Stro
KLF6	7.00E-149	0.310102	0.812	0.652	1.27E-144	Stro
RCN1	1.04E-148	0.350781	0.584	0.377	1.90E-144	Stro
PBXIP1	1.07E-148	0.264897	0.375	0.165	1.95E-144	Stro
NTRK2	6.86E-148	0.25327	0.538	0.26	1.25E-143	Stro
SELK	4.69E-146	0.264826	0.938	0.847	8.54E-142	Stro
SSPN	3.63E-145	0.295615	0.39	0.183	6.62E-141	Stro
PDIA6	4.67E-144	0.329599	0.849	0.752	8.51E-140	Stro
UGDH	1.11E-143	0.256742	0.346	0.149	2.02E-139	Stro
PDLIM5	1.67E-143	0.275397	0.488	0.259	3.04E-139	Stro
BOC	3.01E-143	0.266406	0.36	0.158	5.49E-139	Stro
GADD45A	4.05E-143	0.429829	0.748	0.575	7.37E-139	Stro
CSRNP1	4.60E-143	0.369008	0.599	0.384	8.37E-139	Stro
EIF5B	3.37E-142	0.337736	0.766	0.613	6.15E-138	Stro
TSPAN6	1.12E-141	0.290582	0.475	0.261	2.05E-137	Stro
DHRS7	3.59E-141	0.320905	0.599	0.396	6.54E-137	Stro
CEBPB	5.71E-140	0.301528	0.802	0.62	1.04E-135	Stro
GUSB	1.03E-138	0.307173	0.469	0.258	1.87E-134	Stro
TXLNG	8.01E-138	0.260206	0.298	0.119	1.46E-133	Stro
PRAF2	1.21E-137	0.262467	0.359	0.163	2.21E-133	Stro
MLF1	3.36E-137	0.351978	0.474	0.261	6.12E-133	Stro
COPRS	9.27E-137	0.274965	0.395	0.196	1.69E-132	Stro
HEXIM1	6.83E-135	0.337736	0.585	0.376	1.24E-130	Stro
TMED7	1.02E-134	0.274432	0.379	0.183	1.85E-130	Stro
ARL6IP5	1.87E-134	0.299019	0.883	0.776	3.40E-130	Stro
TCTN3	9.43E-134	0.261707	0.419	0.212	1.72E-129	Stro
IFRD1	4.27E-133	0.350074	0.587	0.38	7.78E-129	Stro
PQLC3	4.47E-133	0.2882	0.465	0.255	8.13E-129	Stro
PGM1	6.28E-133	0.275404	0.434	0.231	1.14E-128	Stro
KCTD10	1.09E-131	0.263425	0.394	0.196	1.98E-127	Stro
NTF3	1.99E-129	0.290755	0.4	0.196	3.62E-125	Stro
NAP1L1	9.63E-129	0.273782	0.843	0.716	1.75E-124	Stro
TGOLN2	2.07E-128	0.283029	0.452	0.25	3.77E-124	Stro

UNC50	2.10E-128	0.300772	0.594	0.397	3.81E-124	Stro
RABAC1	4.72E-128	0.311839	0.843	0.741	8.59E-124	Stro
MYADM	2.28E-127	0.297366	0.448	0.238	4.14E-123	Stro
MED10	3.51E-127	0.31843	0.701	0.538	6.40E-123	Stro
DLST	4.90E-127	0.255518	0.383	0.189	8.92E-123	Stro
SERINC3	1.12E-125	0.280628	0.502	0.3	2.05E-121	Stro
SEPT2	8.33E-125	0.319288	0.656	0.477	1.52E-120	Stro
CMSS1	3.45E-123	0.257273	0.38	0.187	6.28E-119	Stro
ITFG1	3.96E-122	0.288036	0.513	0.317	7.21E-118	Stro
TMEM248	1.74E-121	0.297447	0.536	0.347	3.17E-117	Stro
SON	2.07E-121	0.313979	0.801	0.67	3.77E-117	Stro
EPHX1	4.92E-121	0.301984	0.656	0.466	8.97E-117	Stro
SURF4	1.57E-120	0.307215	0.581	0.397	2.86E-116	Stro
RABGEF1	1.40E-119	0.268175	0.349	0.168	2.56E-115	Stro
PITPNB	1.94E-118	0.292712	0.509	0.312	3.54E-114	Stro
KLF4	4.99E-118	0.347819	0.952	0.911	9.09E-114	Stro
PELO	1.49E-117	0.302712	0.441	0.255	2.71E-113	Stro
TMEM109	6.25E-117	0.292351	0.672	0.5	1.14E-112	Stro
FBXO21	1.84E-116	0.250075	0.394	0.202	3.35E-112	Stro
EIF4B	1.55E-115	0.304598	0.64	0.465	2.82E-111	Stro
C1orf122	7.24E-114	0.282736	0.499	0.311	1.32E-109	Stro
PTP4A2	8.26E-114	0.288045	0.556	0.364	1.50E-109	Stro
PHACTR2	1.14E-113	0.278272	0.428	0.239	2.07E-109	Stro
RSL24D1	1.10E-111	0.295983	0.775	0.649	2.00E-107	Stro
DKK3	4.79E-110	0.284714	0.843	0.612	8.71E-106	Stro
TPBG	1.63E-109	0.289715	0.52	0.334	2.96E-105	Stro
VAMP2	2.38E-109	0.26991	0.838	0.736	4.34E-105	Stro
MTDH	1.84E-108	0.308217	0.705	0.551	3.34E-104	Stro
KLC1	2.22E-108	0.277164	0.503	0.325	4.04E-104	Stro
S100A13	3.01E-108	0.289133	0.748	0.592	5.49E-104	Stro
ACAT1	2.39E-107	0.271922	0.481	0.295	4.35E-103	Stro
CTSB	3.74E-107	0.323149	0.837	0.772	6.82E-103	Stro
BSG	7.04E-107	0.279367	0.908	0.839	1.28E-102	Stro
SVIP	7.16E-107	0.279466	0.497	0.305	1.30E-102	Stro
H1F0	3.29E-104	0.291388	0.477	0.285	5.99E-100	Stro
WSB1	4.97E-104	0.286614	0.735	0.584	9.06E-100	Stro
SPG21	8.53E-103	0.266857	0.547	0.364	1.55E-98	Stro
KDELR2	2.11E-102	0.290565	0.754	0.644	3.85E-98	Stro
MCL1	3.15E-102	0.285911	0.806	0.68	5.74E-98	Stro
BRD2	4.59E-101	0.302826	0.716	0.575	8.35E-97	Stro
KIF5B	1.47E-100	0.2712	0.8	0.694	2.68E-96	Stro
SERINC1	2.23E-100	0.260153	0.673	0.506	4.06E-96	Stro
SLC39A7	3.78E-100	0.250291	0.414	0.24	6.88E-96	Stro
EIF1B	7.72E-100	0.295962	0.789	0.7	1.41E-95	Stro
ESD	1.18E-99	0.270196	0.671	0.51	2.14E-95	Stro
SLC44A2	1.02E-98	0.262682	0.484	0.309	1.86E-94	Stro
NXF1	1.07E-98	0.298406	0.386	0.218	1.96E-94	Stro
MGME1	2.80E-96	0.264461	0.464	0.293	5.09E-92	Stro

MPHOSPH8	4.21E-93	0.264897	0.617	0.459	7.66E-89	Stro
RBM7	4.40E-93	0.264089	0.447	0.279	8.01E-89	Stro
ADI1	2.54E-90	0.269704	0.852	0.771	4.63E-86	Stro
RPN2	3.06E-90	0.259653	0.821	0.734	5.58E-86	Stro
EIF4G2	4.12E-89	0.251349	0.878	0.789	7.50E-85	Stro
BZW1	7.00E-89	0.273019	0.815	0.699	1.28E-84	Stro
VIMP	4.79E-87	0.272915	0.839	0.768	8.72E-83	Stro
CCDC47	5.06E-87	0.263985	0.669	0.529	9.22E-83	Stro
RASD1	1.20E-84	0.270354	0.598	0.415	2.19E-80	Stro
MATR3	4.39E-83	0.25349	0.652	0.512	7.99E-79	Stro
CNOT2	1.61E-82	0.26911	0.448	0.298	2.93E-78	Stro
INSIG1	5.63E-82	0.265873	0.36	0.204	1.02E-77	Stro
SDF2L1	6.20E-82	0.262	0.533	0.374	1.13E-77	Stro
DDX24	1.92E-81	0.265404	0.746	0.641	3.49E-77	Stro
ARID5B	9.64E-81	0.38467	0.543	0.41	1.76E-76	Stro
SCPEP1	1.32E-80	0.25534	0.578	0.431	2.40E-76	Stro
MAP1LC3B	5.58E-79	0.293258	0.862	0.806	1.02E-74	Stro
MAT2A	2.24E-78	0.265048	0.355	0.205	4.08E-74	Stro
IRF2BPL	6.51E-73	0.258705	0.432	0.287	1.19E-68	Stro
DNTTIP2	3.45E-69	0.260622	0.594	0.467	6.29E-65	Stro
GRPEL1	2.90E-67	0.282682	0.63	0.527	5.28E-63	Stro
OSER1	7.90E-59	0.259919	0.6	0.486	1.44E-54	Stro
KRT13	0	1.961534	0.998	0.604	0	Conj-1
KRT4	0	1.609484	0.759	0.121	0	Conj-1
SLPI	0	1.396151	0.998	0.384	0	Conj-1
AQP5	0	1.368298	0.756	0.13	0	Conj-1
AQP3	0	1.295185	0.997	0.621	0	Conj-1
HIST2H2AA4	0	1.290604	0.97	0.683	0	Conj-1
OSR2	0	1.240673	0.89	0.17	0	Conj-1
CXCL17	0	1.207503	0.921	0.331	0	Conj-1
S100A9	0	1.193149	0.786	0.347	0	Conj-1
IGFBP3	0	1.018637	0.642	0.147	0	Conj-1
ELF3	0	0.990826	0.997	0.784	0	Conj-1
F3	0	0.978395	0.741	0.178	0	Conj-1
S100A11	0	0.972731	1	0.984	0	Conj-1
KRT8	0	0.969159	0.829	0.336	0	Conj-1
HOPX	0	0.947441	0.971	0.504	0	Conj-1
CFD	0	0.936228	0.813	0.296	0	Conj-1
HIST1H2AC	0	0.891263	0.617	0.123	0	Conj-1
LSP1	0	0.888146	0.822	0.259	0	Conj-1
DDIT4	0	0.882586	0.855	0.408	0	Conj-1
FAM213A	0	0.85451	0.862	0.271	0	Conj-1
NUPR1	0	0.826364	0.975	0.649	0	Conj-1
RHOV	0	0.800939	0.98	0.594	0	Conj-1
SYT8	0	0.782984	0.987	0.728	0	Conj-1
FMO1	0	0.780868	0.598	0.12	0	Conj-1
LCN2	0	0.752909	0.65	0.161	0	Conj-1
ZFP36L1	0	0.751756	0.978	0.829	0	Conj-1

PHLDA2	0	0.749422	0.998	0.891	0	Conj-1
HIST1H2BC	0	0.739327	0.492	0.088	0	Conj-1
MDK	0	0.696436	0.737	0.22	0	Conj-1
CLEC2B	0	0.691921	0.935	0.6	0	Conj-1
LMO4	0	0.688849	0.872	0.534	0	Conj-1
C9orf16	0	0.688617	0.977	0.843	0	Conj-1
KLF5	0	0.684676	0.962	0.647	0	Conj-1
TNNI2	0	0.675073	0.682	0.212	0	Conj-1
B2M	0	0.669085	1	0.997	0	Conj-1
HIST2H2BE	0	0.667654	0.655	0.202	0	Conj-1
ID1	0	0.654483	0.996	0.954	0	Conj-1
BTG1	0	0.652022	0.993	0.947	0	Conj-1
CAPNS2	0	0.647519	0.874	0.551	0	Conj-1
FAM3B	0	0.646291	0.613	0.111	0	Conj-1
SERTAD1	0	0.644603	0.856	0.562	0	Conj-1
HSPB1	0	0.613099	1	0.983	0	Conj-1
S100A16	0	0.60601	0.953	0.689	0	Conj-1
RARRES3	0	0.595588	0.758	0.365	0	Conj-1
CSTA	0	0.594707	0.982	0.732	0	Conj-1
A4GALT	0	0.593256	0.839	0.486	0	Conj-1
POLR2J2	0	0.586182	1	0.865	0	Conj-1
HIST1H2BD	0	0.571612	0.508	0.111	0	Conj-1
CHCHD10	0	0.570555	0.682	0.228	0	Conj-1
CD74	0	0.563339	0.805	0.373	0	Conj-1
BAALC	0	0.555794	0.487	0.08	0	Conj-1
BAG1	0	0.549953	0.985	0.894	0	Conj-1
TNNT3	0	0.546974	0.806	0.405	0	Conj-1
HES4	0	0.544704	0.772	0.347	0	Conj-1
BARX2	0	0.542924	0.535	0.073	0	Conj-1
CP	0	0.542859	0.503	0.049	0	Conj-1
DEFB1	0	0.537387	0.77	0.35	0	Conj-1
RERG	0	0.517091	0.594	0.146	0	Conj-1
KLK11	0	0.513991	0.706	0.305	0	Conj-1
ABRACL	0	0.511724	0.748	0.359	0	Conj-1
HIST1H2BK	0	0.476313	0.52	0.136	0	Conj-1
HIST1H2AE	0	0.475341	0.418	0.091	0	Conj-1
HIST1H2BG	0	0.471135	0.401	0.063	0	Conj-1
RPS29	0	0.450481	0.998	0.996	0	Conj-1
ZNF296	0	0.44936	0.487	0.123	0	Conj-1
RPS6	0	0.437619	0.999	0.999	0	Conj-1
CTSS	0	0.423727	0.573	0.189	0	Conj-1
RPL10	0	0.420168	1	1	0	Conj-1
RPLP1	0	0.418952	1	1	0	Conj-1
RPS15A	0	0.417398	0.999	0.996	0	Conj-1
PRSS22	0	0.403029	0.473	0.133	0	Conj-1
RPS28	0	0.391504	1	0.999	0	Conj-1
RPL18A	0	0.388289	0.999	0.997	0	Conj-1
RPL29	0	0.387061	0.997	0.992	0	Conj-1

IFT172	0	0.386226	0.423	0.082	0	Conj-1
RPL13	0	0.384935	1	0.999	0	Conj-1
RHCG	0	0.383754	0.323	0.054	0	Conj-1
RPS27	0	0.380355	1	1	0	Conj-1
RPL41	0	0.372537	1	1	0	Conj-1
RPS18	0	0.370506	1	1	0	Conj-1
RPL27A	0	0.359315	1	0.998	0	Conj-1
RPL12	0	0.351972	0.999	0.998	0	Conj-1
RPL10A	0	0.351785	0.998	0.994	0	Conj-1
MX1	0	0.349703	0.4	0.092	0	Conj-1
RPS14	0	0.34951	1	1	0	Conj-1
RPL23A	0	0.338191	0.999	0.999	0	Conj-1
RPL13A	0	0.335813	1	0.999	0	Conj-1
RPL21	0	0.334296	0.999	0.999	0	Conj-1
PPP1R1B	0	0.329836	0.33	0.04	0	Conj-1
RPL34	0	0.325606	1	1	0	Conj-1
LRG1	0	0.322319	0.319	0.03	0	Conj-1
CYP4B1	0	0.295533	0.307	0.043	0	Conj-1
SOX7	0	0.29307	0.331	0.062	0	Conj-1
RPS13	0	0.291561	0.997	0.998	0	Conj-1
TF	0	0.272037	0.328	0.062	0	Conj-1
HLA-DRA	0	0.255964	0.545	0.163	0	Conj-1
DNAJB1	1.78E-307	0.500605	0.987	0.902	3.25E-303	Conj-1
DUSP2	1.98E-307	0.537872	0.473	0.138	3.60E-303	Conj-1
RPL32	5.79E-304	0.305581	0.998	0.999	1.05E-299	Conj-1
SLC9A3R1	2.19E-302	0.518386	0.807	0.457	3.99E-298	Conj-1
GBP2	1.17E-299	0.273384	0.308	0.058	2.13E-295	Conj-1
S100A8	2.59E-295	1.010454	0.695	0.379	4.72E-291	Conj-1
RPL8	9.18E-294	0.309989	0.999	0.998	1.67E-289	Conj-1
POLR2J3	6.59E-287	0.556196	0.999	0.856	1.20E-282	Conj-1
ZNF385A	1.07E-286	0.47119	0.768	0.411	1.94E-282	Conj-1
RPLP0	6.61E-285	0.44406	0.998	0.991	1.20E-280	Conj-1
RPS8	1.59E-280	0.33865	0.999	0.999	2.89E-276	Conj-1
HLA-B	1.82E-274	0.587566	0.983	0.917	3.32E-270	Conj-1
SOX15	3.59E-273	0.510353	0.957	0.754	6.54E-269	Conj-1
RPS19	1.33E-272	0.373249	0.999	0.998	2.42E-268	Conj-1
TACSTD2	2.31E-271	0.479957	1	0.888	4.20E-267	Conj-1
AX747171	6.26E-270	0.395679	0.469	0.152	1.14E-265	Conj-1
CLIC1	1.87E-269	0.424861	0.982	0.904	3.41E-265	Conj-1
SOCS1	2.53E-269	0.41499	0.431	0.131	4.60E-265	Conj-1
RPS9	7.30E-268	0.294271	0.999	0.998	1.33E-263	Conj-1
KRT7	1.96E-266	0.382467	0.289	0.057	3.58E-262	Conj-1
RPL28	1.98E-263	0.43228	0.999	0.991	3.60E-259	Conj-1
ARL4A	2.29E-259	0.561618	0.864	0.582	4.16E-255	Conj-1
TXNIP	1.84E-257	0.716335	0.882	0.647	3.35E-253	Conj-1
WFDC2	4.73E-257	0.341938	0.295	0.061	8.60E-253	Conj-1
PLK2	3.52E-251	0.535866	0.827	0.49	6.42E-247	Conj-1
CALML3	2.02E-250	0.575513	0.929	0.67	3.67E-246	Conj-1

MARCKSL1	1.63E-249	0.483417	0.545	0.207	2.97E-245	Conj-1
NPL	8.23E-247	0.304635	0.392	0.115	1.50E-242	Conj-1
H2AFX	1.45E-243	0.390048	0.482	0.172	2.63E-239	Conj-1
MUC20	1.44E-239	0.419361	0.693	0.325	2.63E-235	Conj-1
TMEM40	3.04E-239	0.417191	0.639	0.297	5.53E-235	Conj-1
ZFP36	9.16E-239	0.466542	0.996	0.973	1.67E-234	Conj-1
RPL19	2.50E-238	0.27039	0.999	0.999	4.54E-234	Conj-1
FAM3D	2.87E-237	0.307008	0.489	0.162	5.23E-233	Conj-1
MAOA	4.52E-237	0.389226	0.534	0.212	8.23E-233	Conj-1
RPS27A	7.39E-236	0.268261	0.998	0.997	1.35E-231	Conj-1
KCNK6	7.99E-236	0.312292	0.427	0.137	1.46E-231	Conj-1
RPS4X	7.67E-235	0.305956	0.999	0.999	1.40E-230	Conj-1
CSRP2	1.35E-234	0.533625	0.993	0.866	2.46E-230	Conj-1
RPL35	6.51E-234	0.266226	0.999	0.999	1.18E-229	Conj-1
C1orf63	1.26E-233	0.508037	0.771	0.477	2.30E-229	Conj-1
TNFRSF21	2.00E-228	0.268342	0.293	0.068	3.64E-224	Conj-1
RPL14	3.49E-227	0.274107	0.998	0.995	6.36E-223	Conj-1
CLINT1	8.14E-227	0.431084	0.721	0.395	1.48E-222	Conj-1
ZC3H12A	8.53E-227	0.437002	0.552	0.229	1.55E-222	Conj-1
PABPC1	9.02E-225	0.449387	0.964	0.893	1.64E-220	Conj-1
RPS10	5.39E-224	0.350613	0.99	0.977	9.81E-220	Conj-1
FABP5	7.79E-222	0.554111	1	0.937	1.42E-217	Conj-1
RPL18	8.47E-222	0.291793	0.999	0.996	1.54E-217	Conj-1
RPL36	3.59E-219	0.266131	0.998	0.997	6.53E-215	Conj-1
RPL37A	1.51E-217	0.29375	0.998	0.998	2.75E-213	Conj-1
PPAP2C	2.18E-214	0.303419	0.414	0.137	3.96E-210	Conj-1
ADM	5.62E-214	0.495308	0.988	0.875	1.02E-209	Conj-1
S100A14	9.10E-214	0.438581	0.987	0.749	1.66E-209	Conj-1
CCDC80	1.30E-213	0.353027	0.345	0.098	2.37E-209	Conj-1
ZFAS1	1.43E-213	0.411209	0.956	0.854	2.60E-209	Conj-1
RPS17L	2.18E-213	0.289858	0.998	0.996	3.97E-209	Conj-1
EHF	3.13E-212	0.505369	0.627	0.294	5.70E-208	Conj-1
PTGR1	1.52E-211	0.543647	0.653	0.367	2.77E-207	Conj-1
BCAS1	7.03E-211	0.260958	0.313	0.082	1.28E-206	Conj-1
RPS3A	8.49E-211	0.254146	0.998	0.998	1.55E-206	Conj-1
RPS20	1.29E-210	0.274887	0.998	0.996	2.34E-206	Conj-1
GPNMB	6.06E-208	0.418689	0.846	0.51	1.10E-203	Conj-1
RPL5	1.74E-205	0.265554	0.998	0.992	3.17E-201	Conj-1
HES1	2.00E-205	0.657611	0.902	0.738	3.64E-201	Conj-1
CLDN4	2.61E-204	0.407709	0.941	0.576	4.75E-200	Conj-1
MGST2	6.68E-204	0.402324	0.926	0.701	1.22E-199	Conj-1
PMAIP1	8.37E-204	0.484525	0.936	0.672	1.52E-199	Conj-1
RPS24	1.06E-202	0.29465	1	0.998	1.93E-198	Conj-1
DDIT3	2.88E-202	0.529069	0.788	0.515	5.25E-198	Conj-1
FAM46B	7.93E-202	0.403008	0.647	0.326	1.44E-197	Conj-1
SERINC2	1.01E-201	0.44645	0.806	0.519	1.85E-197	Conj-1
RPL17	8.01E-201	0.306653	0.993	0.984	1.46E-196	Conj-1
C19orf33	3.10E-200	0.389404	0.996	0.833	5.65E-196	Conj-1

GADD45G	7.91E-199	0.475151	0.68	0.373	1.44E-194	Conj-1
SERPINB2	2.16E-198	0.748274	0.321	0.09	3.93E-194	Conj-1
UAP1	3.39E-197	0.395896	0.643	0.32	6.17E-193	Conj-1
GSTK1	3.11E-196	0.401174	0.834	0.604	5.66E-192	Conj-1
RASD1	1.56E-193	0.463174	0.718	0.404	2.85E-189	Conj-1
PITPNC1	3.94E-192	0.253894	0.394	0.134	7.18E-188	Conj-1
CDKN1A	1.49E-191	0.394186	0.911	0.661	2.71E-187	Conj-1
RPL36A	1.38E-188	0.303559	0.995	0.988	2.51E-184	Conj-1
ENSG0000021145	8.93E-187	0.552305	1	0.997	1.63E-182	Conj-1
NUAK2	1.96E-186	0.41152	0.711	0.377	3.58E-182	Conj-1
NRARP	1.15E-185	0.299074	0.416	0.151	2.09E-181	Conj-1
CALM1	2.13E-185	0.330073	0.982	0.957	3.88E-181	Conj-1
RPS14P3	1.38E-184	0.313826	0.985	0.958	2.52E-180	Conj-1
SFN	4.30E-183	0.429477	0.997	0.86	7.83E-179	Conj-1
BTG2	6.28E-182	0.463131	0.87	0.621	1.14E-177	Conj-1
MIDN	1.36E-181	0.429126	0.739	0.461	2.48E-177	Conj-1
CRNDE	7.22E-181	0.30464	0.66	0.311	1.31E-176	Conj-1
JUNB	1.27E-180	0.379864	0.998	0.995	2.31E-176	Conj-1
DEGS1	2.23E-179	0.40263	0.656	0.34	4.06E-175	Conj-1
RPS2	5.42E-179	0.271587	0.998	0.997	9.86E-175	Conj-1
BBC3	6.38E-179	0.285352	0.394	0.142	1.16E-174	Conj-1
SLC1A5	7.85E-179	0.391124	0.71	0.425	1.43E-174	Conj-1
ENSG0000021008	7.22E-178	0.403337	1	1	1.31E-173	Conj-1
PDLIM1	3.03E-176	0.386217	0.928	0.75	5.51E-172	Conj-1
RPLP2	4.74E-176	0.259865	0.999	0.999	8.63E-172	Conj-1
EEF1G	1.12E-175	0.267416	0.993	0.988	2.04E-171	Conj-1
GADD45B	2.81E-175	0.404461	0.981	0.92	5.11E-171	Conj-1
MYLIP	1.24E-174	0.380557	0.602	0.317	2.25E-170	Conj-1
CTAGE5	5.07E-174	0.277185	0.426	0.166	9.23E-170	Conj-1
COX7A1	7.08E-174	0.440603	0.761	0.444	1.29E-169	Conj-1
IRF1	4.01E-173	0.401164	0.633	0.341	7.30E-169	Conj-1
LINC00673	5.56E-170	0.271521	0.379	0.135	1.01E-165	Conj-1
ID3	7.92E-168	0.324222	0.923	0.737	1.44E-163	Conj-1
H2AFJ	3.63E-167	0.376015	0.785	0.521	6.61E-163	Conj-1
HLA-C	4.68E-165	0.359684	0.983	0.952	8.52E-161	Conj-1
ENSG0000019876	9.12E-165	0.419668	0.999	0.981	1.66E-160	Conj-1
HBEGF	3.28E-162	0.328833	0.576	0.28	5.98E-158	Conj-1
NDRG2	6.55E-162	0.334061	0.615	0.321	1.19E-157	Conj-1
KRT17	1.19E-161	0.780389	0.673	0.386	2.16E-157	Conj-1
PITX1	2.40E-161	0.264175	0.291	0.088	4.36E-157	Conj-1
AREG	1.10E-159	0.477017	0.71	0.408	2.01E-155	Conj-1
EVA1A	1.34E-159	0.261742	0.418	0.167	2.44E-155	Conj-1
IL20RB	4.57E-159	0.378583	0.776	0.504	8.32E-155	Conj-1
RRAD	4.31E-158	0.305826	0.323	0.107	7.84E-154	Conj-1
NFE2L2	7.20E-158	0.444191	0.895	0.799	1.31E-153	Conj-1
OVOL1	2.25E-156	0.334691	0.631	0.327	4.09E-152	Conj-1
RPS16	9.53E-156	0.266542	0.997	0.994	1.74E-151	Conj-1
ZNF593	2.40E-155	0.333484	0.584	0.298	4.36E-151	Conj-1

TXN	3.19E-155	0.46864	0.998	0.979	5.80E-151	Conj-1
RAC1	7.57E-155	0.283559	0.979	0.951	1.38E-150	Conj-1
EHBP1	2.30E-153	0.275693	0.441	0.188	4.19E-149	Conj-1
TNFSF10	7.85E-153	0.419326	0.851	0.624	1.43E-148	Conj-1
FAM83A	3.66E-152	0.381045	0.804	0.553	6.66E-148	Conj-1
HNRNPA1	3.82E-147	0.263748	0.984	0.968	6.96E-143	Conj-1
SRSF5	5.30E-147	0.319357	0.979	0.933	9.66E-143	Conj-1
GPRC5C	7.39E-142	0.304871	0.53	0.273	1.35E-137	Conj-1
TUBB2A	1.88E-140	0.374087	0.916	0.746	3.42E-136	Conj-1
AES	1.38E-138	0.331197	0.835	0.618	2.50E-134	Conj-1
AKR1A1	2.61E-137	0.342615	0.835	0.64	4.76E-133	Conj-1
ARPC3	3.86E-137	0.302765	0.948	0.872	7.03E-133	Conj-1
ALDH2	4.86E-137	0.305241	0.627	0.349	8.85E-133	Conj-1
TUBB4B	2.69E-135	0.376644	0.972	0.915	4.91E-131	Conj-1
ZNF750	1.82E-134	0.278522	0.561	0.283	3.31E-130	Conj-1
MDH2	5.39E-134	0.315776	0.81	0.596	9.82E-130	Conj-1
FXYD3	1.73E-131	0.314606	0.995	0.847	3.16E-127	Conj-1
RIPK4	1.64E-124	0.317308	0.615	0.354	3.00E-120	Conj-1
GLTSCR2	4.61E-124	0.28251	0.909	0.816	8.39E-120	Conj-1
BHLHE40	6.10E-124	0.34065	0.889	0.711	1.11E-119	Conj-1
SLC25A5	2.50E-123	0.316186	0.94	0.865	4.54E-119	Conj-1
HIST1H1C	3.67E-123	0.329441	0.427	0.197	6.68E-119	Conj-1
EFNA1	1.05E-122	0.343627	0.871	0.677	1.90E-118	Conj-1
PSMB8	1.06E-122	0.312896	0.694	0.452	1.93E-118	Conj-1
ATF3	3.15E-122	0.265345	0.897	0.672	5.74E-118	Conj-1
MGST1	5.26E-121	0.491798	0.451	0.229	9.57E-117	Conj-1
COX8A	2.11E-120	0.27602	0.935	0.838	3.85E-116	Conj-1
ETS2	1.68E-118	0.270441	0.585	0.326	3.07E-114	Conj-1
TCEA3	2.55E-117	0.289092	0.726	0.467	4.64E-113	Conj-1
PIM3	3.25E-117	0.315207	0.746	0.515	5.91E-113	Conj-1
CDKN2B	5.92E-117	0.273778	0.498	0.253	1.08E-112	Conj-1
VAV3	1.33E-115	0.311846	0.645	0.399	2.42E-111	Conj-1
GPR87	1.50E-114	0.273989	0.528	0.29	2.73E-110	Conj-1
PSMB10	3.19E-111	0.30523	0.671	0.44	5.81E-107	Conj-1
GAS5	1.62E-110	0.253196	0.98	0.928	2.95E-106	Conj-1
NOTCH2NL	9.83E-109	0.302437	0.749	0.528	1.79E-104	Conj-1
NFKBIA	1.10E-106	0.305084	0.978	0.938	2.00E-102	Conj-1
NFKBIZ	1.43E-103	0.271445	0.549	0.314	2.60E-99	Conj-1
HS3ST1	8.65E-102	0.348913	0.333	0.146	1.57E-97	Conj-1
EIF6	1.27E-101	0.286746	0.902	0.753	2.31E-97	Conj-1
RAB25	6.07E-99	0.258585	0.813	0.555	1.11E-94	Conj-1
PRSS8	1.13E-97	0.254983	0.592	0.359	2.06E-93	Conj-1
NOL3	2.23E-97	0.260857	0.547	0.323	4.06E-93	Conj-1
ARL4D	2.15E-96	0.319302	0.938	0.807	3.92E-92	Conj-1
ENSG00000198888	3.99E-96	0.312626	0.997	0.955	7.27E-92	Conj-1
CCDC12	7.92E-96	0.280636	0.8	0.586	1.44E-91	Conj-1
CITED2	1.75E-95	0.274378	0.809	0.627	3.19E-91	Conj-1
POLR1D	7.59E-93	0.253713	0.935	0.814	1.38E-88	Conj-1

CYBA	5.54E-92	0.265772	0.728	0.53	1.01E-87	Conj-1
CHMP1B	1.45E-89	0.297988	0.877	0.722	2.63E-85	Conj-1
L32131	1.89E-88	0.260601	0.878	0.726	3.44E-84	Conj-1
TOB2	3.39E-87	0.25217	0.562	0.348	6.18E-83	Conj-1
PDCD4	3.97E-85	0.294789	0.854	0.691	7.23E-81	Conj-1
PTP4A1	7.75E-83	0.298103	0.782	0.596	1.41E-78	Conj-1
ARL6IP1	1.53E-82	0.25236	0.878	0.765	2.79E-78	Conj-1
MPZL2	1.57E-81	0.292729	0.858	0.662	2.87E-77	Conj-1
RPL22L1	7.47E-80	0.253057	0.742	0.56	1.36E-75	Conj-1
THRB	2.17E-79	0.264548	0.764	0.559	3.95E-75	Conj-1
IL1RN	2.42E-79	0.630563	0.698	0.542	4.41E-75	Conj-1
SAT1	2.11E-72	0.25746	1	0.989	3.85E-68	Conj-1
ENSG00000198840	3.87E-71	0.28373	0.975	0.87	7.05E-67	Conj-1
APOBEC3A	5.34E-68	0.636582	0.518	0.321	9.73E-64	Conj-1
SERPINB1	1.73E-61	0.281932	0.735	0.583	3.14E-57	Conj-1
FOSL1	9.50E-49	0.42678	0.453	0.298	1.73E-44	Conj-1
CRIP1	9.47E-30	0.437973	0.794	0.746	1.72E-25	Conj-1
GADD45A	4.20E-28	0.253936	0.685	0.59	7.64E-24	Conj-1
PLAUR	4.65E-24	0.417961	0.38	0.278	8.46E-20	Conj-1
FGFBP1	7.18E-08	0.264509	0.479	0.429	0.001307	Conj-1
TM4SF1	6.51E-07	0.399774	0.722	0.682	0.011848	Conj-1
CXCL14	0	1.591442	1	0.876	0	Epi-B1
KRT14	0	1.5182	0.933	0.531	0	Epi-B1
CPVL	0	1.327566	0.902	0.095	0	Epi-B1
IGFBP7	0	1.304826	0.998	0.611	0	Epi-B1
ALDH3A1	0	1.199679	0.999	0.974	0	Epi-B1
BCAM	0	1.156444	0.927	0.249	0	Epi-B1
GJB6	0	1.1312	0.998	0.573	0	Epi-B1
SPARC	0	1.100211	0.977	0.381	0	Epi-B1
COL17A1	0	1.079008	0.979	0.42	0	Epi-B1
NQO1	0	1.068249	0.999	0.94	0	Epi-B1
CAV1	0	1.041249	0.878	0.179	0	Epi-B1
CTSL2	0	0.990495	0.998	0.604	0	Epi-B1
NTRK2	0	0.986706	0.885	0.221	0	Epi-B1
TGFBI	0	0.918198	0.999	0.89	0	Epi-B1
FTH1	0	0.874095	1	1	0	Epi-B1
S100A2	0	0.839896	0.803	0.379	0	Epi-B1
CRTAC1	0	0.834616	0.992	0.661	0	Epi-B1
S100A10	0	0.813292	1	0.944	0	Epi-B1
HOMER3	0	0.791025	0.867	0.368	0	Epi-B1
MOXD1	0	0.782163	0.705	0.106	0	Epi-B1
DST	0	0.768071	0.828	0.3	0	Epi-B1
EFEMP1	0	0.766986	0.823	0.244	0	Epi-B1
CLU	0	0.759112	1	0.99	0	Epi-B1
IVNS1ABP	0	0.746211	0.859	0.405	0	Epi-B1
GAPDH	0	0.738834	1	0.998	0	Epi-B1
WNT10A	0	0.738677	0.668	0.051	0	Epi-B1
GJB2	0	0.73511	0.997	0.633	0	Epi-B1

ATP1B3	0	0.710723	0.983	0.802	0	Epi-B1
SUCO	0	0.702307	0.925	0.494	0	Epi-B1
RASSF6	0	0.681604	0.761	0.278	0	Epi-B1
TKT	0	0.676103	0.999	0.849	0	Epi-B1
KCNN4	0	0.670544	0.814	0.292	0	Epi-B1
VIT	0	0.656052	0.683	0.099	0	Epi-B1
TSPAN1	0	0.655157	0.847	0.497	0	Epi-B1
HSD17B2	0	0.636309	0.612	0.103	0	Epi-B1
IGFBP6	0	0.612162	1	0.821	0	Epi-B1
ASCL2	0	0.606588	0.598	0.117	0	Epi-B1
SLC3A2	0	0.604098	0.967	0.763	0	Epi-B1
C1QBP	0	0.592019	0.989	0.859	0	Epi-B1
CLTB	0	0.576101	0.986	0.89	0	Epi-B1
HSPA2	0	0.574617	0.764	0.359	0	Epi-B1
DAAM1	0	0.57385	0.929	0.599	0	Epi-B1
FTH1P3	0	0.572356	0.887	0.547	0	Epi-B1
AKR1B1	0	0.571277	0.832	0.417	0	Epi-B1
PKM	0	0.563074	0.999	0.978	0	Epi-B1
CAV2	0	0.557461	0.698	0.226	0	Epi-B1
LGALS7	0	0.553182	0.682	0.276	0	Epi-B1
PYCR2	0	0.54835	0.753	0.364	0	Epi-B1
TMEM100	0	0.542387	0.665	0.245	0	Epi-B1
CLCA2	0	0.518869	0.796	0.398	0	Epi-B1
HMGN3	0	0.512332	0.998	0.921	0	Epi-B1
GDPD2	0	0.487692	0.615	0.208	0	Epi-B1
SOD1	0	0.480129	0.988	0.888	0	Epi-B1
LDHA	0	0.477725	1	0.989	0	Epi-B1
MMP28	0	0.46633	0.541	0.115	0	Epi-B1
GPX1	0	0.462709	0.995	0.94	0	Epi-B1
SLC7A8	0	0.449605	0.608	0.2	0	Epi-B1
AGL	0	0.40738	0.566	0.188	0	Epi-B1
TGM2	0	0.40471	0.525	0.157	0	Epi-B1
CDH13	0	0.38239	0.461	0.099	0	Epi-B1
CRLF1	0	0.312884	0.397	0.091	0	Epi-B1
LAMA3	0	0.275625	0.346	0.06	0	Epi-B1
PDPN	0	0.264539	0.349	0.065	0	Epi-B1
BASP1	0	0.263751	0.3	0.051	0	Epi-B1
GSTA4	4.13E-302	0.531623	0.901	0.571	7.51E-298	Epi-B1
RGS20	2.15E-301	0.255419	0.306	0.057	3.91E-297	Epi-B1
PTRF	3.98E-293	0.359777	0.862	0.399	7.25E-289	Epi-B1
TNNT1	4.04E-291	0.280119	0.297	0.056	7.36E-287	Epi-B1
GJA1	3.08E-289	0.537322	0.957	0.658	5.60E-285	Epi-B1
TSPAN15	2.56E-284	0.37524	0.499	0.163	4.66E-280	Epi-B1
HCFC1R1	2.63E-280	0.47438	0.795	0.447	4.79E-276	Epi-B1
LGALS7B	1.14E-277	0.584203	0.649	0.277	2.07E-273	Epi-B1
ENSG0000019880	2.41E-273	0.381867	0.998	0.998	4.39E-269	Epi-B1
ARHGDIB	1.51E-270	0.493917	0.755	0.395	2.74E-266	Epi-B1
LAMB1	6.45E-270	0.26325	0.327	0.072	1.17E-265	Epi-B1

PTGES	2.66E-269	0.495845	0.929	0.688	4.85E-265	Epi-B1
RDX	9.92E-268	0.476204	0.872	0.57	1.81E-263	Epi-B1
SLC2A1	1.92E-264	0.512526	0.974	0.844	3.50E-260	Epi-B1
P4HA2	8.04E-261	0.371329	0.529	0.185	1.46E-256	Epi-B1
S100A6	1.72E-252	0.3116	1	0.999	3.13E-248	Epi-B1
ENSG0000019889	1.99E-250	0.410931	0.998	0.986	3.62E-246	Epi-B1
ENSG0000019888	4.70E-250	0.382133	0.999	0.998	8.55E-246	Epi-B1
ENO1	1.57E-249	0.415972	1	0.993	2.87E-245	Epi-B1
LAMB3	5.77E-242	0.361231	0.576	0.225	1.05E-237	Epi-B1
GSN	5.99E-242	0.406887	0.996	0.894	1.09E-237	Epi-B1
SOSTDC1	6.79E-242	0.272574	0.339	0.086	1.24E-237	Epi-B1
HN1	1.89E-240	0.437659	0.854	0.542	3.44E-236	Epi-B1
SLC31A2	2.44E-240	0.337541	0.469	0.163	4.44E-236	Epi-B1
ENSG0000021145	3.20E-238	0.505893	0.997	0.997	5.82E-234	Epi-B1
TNFRSF12A	5.79E-236	0.497631	0.703	0.342	1.05E-231	Epi-B1
GAS1	9.69E-235	0.349112	0.452	0.15	1.77E-230	Epi-B1
FAM134B	2.56E-232	0.391631	0.615	0.267	4.67E-228	Epi-B1
RAB4A	1.97E-231	0.414239	0.922	0.712	3.59E-227	Epi-B1
PLLP	2.84E-231	0.329545	0.493	0.182	5.17E-227	Epi-B1
IMPA2	7.27E-229	0.37336	0.58	0.246	1.32E-224	Epi-B1
SNAI2	8.19E-226	0.421472	0.671	0.319	1.49E-221	Epi-B1
WDR54	7.28E-225	0.309302	0.472	0.166	1.33E-220	Epi-B1
ENSG0000019893	5.26E-222	0.362702	0.997	0.998	9.58E-218	Epi-B1
LYPD6B	1.05E-215	0.35191	0.614	0.273	1.90E-211	Epi-B1
ETV4	1.30E-215	0.254509	0.349	0.1	2.36E-211	Epi-B1
ALDH1A1	1.27E-205	0.423387	0.997	0.858	2.31E-201	Epi-B1
S100A13	1.42E-205	0.406539	0.852	0.583	2.58E-201	Epi-B1
APP	1.70E-198	0.395581	0.827	0.531	3.10E-194	Epi-B1
C16orf74	8.17E-197	0.29999	0.498	0.2	1.49E-192	Epi-B1
PHGDH	2.15E-196	0.366667	0.67	0.348	3.92E-192	Epi-B1
ENSG0000019878	8.87E-193	0.380523	0.994	0.972	1.62E-188	Epi-B1
CAPS	4.58E-190	0.402706	0.796	0.474	8.34E-186	Epi-B1
CBR1	5.91E-190	0.392913	0.892	0.663	1.08E-185	Epi-B1
PDLIM1	1.31E-189	0.357509	0.946	0.748	2.38E-185	Epi-B1
FHOD3	2.54E-186	0.278829	0.452	0.171	4.63E-182	Epi-B1
KRT18	1.37E-184	0.426378	0.944	0.716	2.49E-180	Epi-B1
ARL4C	8.82E-183	0.311516	0.424	0.159	1.61E-178	Epi-B1
ENSG0000019872	1.26E-179	0.309664	0.998	0.993	2.30E-175	Epi-B1
G6PD	1.08E-178	0.357835	0.635	0.337	1.97E-174	Epi-B1
NTF3	9.03E-177	0.289106	0.481	0.192	1.64E-172	Epi-B1
GPX4	1.07E-175	0.305748	0.992	0.962	1.94E-171	Epi-B1
BHLHE41	9.77E-172	0.351189	0.598	0.298	1.78E-167	Epi-B1
GYG1	3.08E-169	0.360337	0.857	0.556	5.60E-165	Epi-B1
NGFRAP1	2.84E-168	0.344745	0.932	0.74	5.18E-164	Epi-B1
ACTG1	3.97E-168	0.264527	0.999	0.994	7.22E-164	Epi-B1
CNTN4	6.26E-166	0.261964	0.413	0.156	1.14E-161	Epi-B1
CRYAB	4.17E-163	0.370698	0.982	0.867	7.60E-159	Epi-B1
C17orf76-AS1	5.50E-160	0.284579	0.997	0.96	1.00E-155	Epi-B1

CFI	3.23E-159	0.271075	0.494	0.222	5.88E-155	Epi-B1
NUDT14	4.13E-158	0.299986	0.601	0.309	7.52E-154	Epi-B1
ENSG0000021008:	5.63E-156	0.342173	0.999	1	1.02E-151	Epi-B1
EXOSC7	3.25E-153	0.33818	0.815	0.572	5.93E-149	Epi-B1
DUSP14	1.57E-150	0.321291	0.623	0.347	2.86E-146	Epi-B1
UACA	5.13E-149	0.308722	0.485	0.229	9.34E-145	Epi-B1
TMEM14A	3.51E-147	0.335463	0.747	0.478	6.40E-143	Epi-B1
MATN2	8.76E-146	0.271832	0.532	0.251	1.60E-141	Epi-B1
ZBED2	9.92E-146	0.25912	0.404	0.165	1.81E-141	Epi-B1
RNF39	5.08E-145	0.38095	0.705	0.422	9.25E-141	Epi-B1
SRPK2	3.35E-143	0.309613	0.687	0.414	6.10E-139	Epi-B1
CCDC51	4.62E-143	0.313848	0.571	0.3	8.40E-139	Epi-B1
DDB2	4.67E-139	0.292381	0.608	0.33	8.50E-135	Epi-B1
BSG	1.06E-138	0.302059	0.95	0.836	1.92E-134	Epi-B1
TUBB	9.04E-138	0.322273	0.936	0.814	1.65E-133	Epi-B1
ADH5	2.35E-135	0.326466	0.799	0.565	4.27E-131	Epi-B1
TSPAN3	2.76E-134	0.296892	0.632	0.361	5.03E-130	Epi-B1
TPD52L1	4.45E-133	0.29667	0.794	0.521	8.10E-129	Epi-B1
PRNP	2.03E-132	0.364509	0.897	0.735	3.69E-128	Epi-B1
IL18	5.15E-131	0.303637	0.741	0.463	9.38E-127	Epi-B1
DUSP23	3.78E-130	0.296268	0.938	0.782	6.89E-126	Epi-B1
ALDH7A1	2.84E-128	0.303722	0.819	0.578	5.17E-124	Epi-B1
TNFRSF1A	1.07E-127	0.310279	0.795	0.577	1.94E-123	Epi-B1
TP53TG1	2.10E-126	0.322448	0.871	0.683	3.81E-122	Epi-B1
PLP2	8.25E-125	0.268092	0.986	0.887	1.50E-120	Epi-B1
CRYZ	1.02E-123	0.280428	0.536	0.284	1.85E-119	Epi-B1
CCDC109B	4.45E-123	0.262151	0.538	0.288	8.11E-119	Epi-B1
ENSG0000021290	3.57E-119	0.306417	0.951	0.824	6.50E-115	Epi-B1
RHBDD2	3.03E-118	0.31606	0.775	0.555	5.51E-114	Epi-B1
ITGA6	2.38E-117	0.25377	0.497	0.251	4.34E-113	Epi-B1
ENSG0000019884(	6.53E-117	0.320293	0.98	0.87	1.19E-112	Epi-B1
RAET1G	1.19E-115	0.275552	0.679	0.411	2.16E-111	Epi-B1
PID1	7.11E-114	0.271001	0.594	0.33	1.29E-109	Epi-B1
ENSG0000019888:	2.54E-112	0.268313	0.994	0.955	4.62E-108	Epi-B1
RNASET2	5.09E-112	0.253395	0.654	0.408	9.27E-108	Epi-B1
OCIAD2	1.98E-111	0.286038	0.838	0.609	3.60E-107	Epi-B1
SMARCA2	5.83E-111	0.272096	0.599	0.354	1.06E-106	Epi-B1
ITGB1	1.26E-109	0.298486	0.803	0.575	2.29E-105	Epi-B1
MAT2B	6.10E-108	0.258576	0.646	0.398	1.11E-103	Epi-B1
HCAR2	4.27E-107	0.511448	0.884	0.663	7.78E-103	Epi-B1
BCL2L13	1.62E-106	0.270834	0.937	0.743	2.94E-102	Epi-B1
MED24	1.01E-105	0.292855	0.449	0.237	1.84E-101	Epi-B1
AGR2	1.56E-104	0.259962	0.88	0.616	2.84E-100	Epi-B1
IER3	6.67E-104	0.308562	0.953	0.815	1.21E-99	Epi-B1
MRPS6	8.35E-104	0.28494	0.773	0.562	1.52E-99	Epi-B1
IL20RB	1.86E-102	0.280079	0.753	0.508	3.38E-98	Epi-B1
GSTO1	9.19E-102	0.273307	0.882	0.755	1.67E-97	Epi-B1
BLCAP	1.49E-101	0.262758	0.731	0.505	2.72E-97	Epi-B1

LIPA	8.69E-99	0.259365	0.469	0.256	1.58E-94	Epi-B1
AVPI1	1.66E-93	0.3129	0.72	0.505	3.02E-89	Epi-B1
C7orf73	7.20E-92	0.265207	0.818	0.643	1.31E-87	Epi-B1
NEDD9	3.54E-75	0.280616	0.319	0.154	6.44E-71	Epi-B1
KRT12	3.39E-74	0.255655	0.997	0.805	6.17E-70	Epi-B1
PLK2	1.92E-72	0.281674	0.713	0.507	3.50E-68	Epi-B1
ARL4D	2.86E-68	0.254558	0.962	0.804	5.21E-64	Epi-B1
SRSF7	4.08E-65	0.290193	0.935	0.843	7.43E-61	Epi-B1
HSPA5	3.35E-51	0.303191	0.968	0.958	6.09E-47	Epi-B1
CITED2	8.48E-43	0.38709	0.748	0.636	1.54E-38	Epi-B1
HCAR3	2.54E-27	0.369616	0.675	0.571	4.62E-23	Epi-B1
CRTAC1	0	0.784068	0.977	0.671	0	Epi-S1
KRT5	1.22E-286	0.615947	1	0.869	2.23E-282	Epi-S1
GJB2	2.83E-278	0.666073	0.981	0.645	5.16E-274	Epi-S1
MT1X	4.30E-271	0.616215	0.997	0.974	7.82E-267	Epi-S1
DSP	1.37E-262	0.617343	0.977	0.738	2.49E-258	Epi-S1
GSN	9.29E-217	0.51302	0.99	0.898	1.69E-212	Epi-S1
KRT12	2.13E-196	0.62124	0.978	0.812	3.88E-192	Epi-S1
GJA1	6.71E-193	0.54498	0.922	0.669	1.22E-188	Epi-S1
ALDH1A1	4.15E-181	0.473721	0.993	0.863	7.55E-177	Epi-S1
IGFBP7	2.23E-179	0.438207	0.933	0.628	4.07E-175	Epi-S1
COL17A1	1.32E-177	0.592557	0.823	0.452	2.41E-173	Epi-S1
FAM84A	4.15E-168	0.511948	0.675	0.383	7.56E-164	Epi-S1
VSNL1	6.03E-168	0.497798	0.826	0.581	1.10E-163	Epi-S1
LYPD1	3.71E-162	0.34328	0.346	0.11	6.75E-158	Epi-S1
ENSG0000019880	4.32E-156	0.470343	1	0.998	7.86E-152	Epi-S1
CXCL14	4.48E-156	0.337688	0.973	0.882	8.16E-152	Epi-S1
LY6D	2.48E-155	0.688238	0.699	0.409	4.51E-151	Epi-S1
SEC16B	4.60E-149	0.373227	0.42	0.16	8.38E-145	Epi-S1
CCND1	1.68E-142	0.489817	0.842	0.646	3.07E-138	Epi-S1
GJB6	8.87E-140	0.40466	0.952	0.589	1.61E-135	Epi-S1
TGFBI	6.97E-139	0.455446	0.986	0.894	1.27E-134	Epi-S1
ELP4	1.70E-134	0.423219	0.586	0.319	3.10E-130	Epi-S1
FABP5	4.19E-134	0.417464	0.997	0.939	7.64E-130	Epi-S1
AIM1	2.12E-126	0.422012	0.725	0.434	3.86E-122	Epi-S1
ENSG0000019889	2.57E-125	0.429685	1	0.986	4.67E-121	Epi-S1
ENSG0000019871	2.85E-125	0.435254	1	0.996	5.20E-121	Epi-S1
TKT	8.85E-123	0.400131	0.986	0.855	1.61E-118	Epi-S1
LDHB	4.80E-122	0.467964	0.891	0.832	8.74E-118	Epi-S1
SUCO	2.47E-121	0.395825	0.781	0.522	4.50E-117	Epi-S1
DSC2	2.94E-119	0.415015	0.786	0.505	5.35E-115	Epi-S1
DSC3	9.77E-119	0.474357	0.738	0.493	1.78E-114	Epi-S1
KRT3	5.15E-117	0.495363	0.857	0.547	9.38E-113	Epi-S1
CSRP2	1.41E-113	0.410877	0.977	0.871	2.56E-109	Epi-S1
FA2H	6.56E-113	0.406157	0.604	0.346	1.19E-108	Epi-S1
ENSG0000019888	9.59E-113	0.38719	1	0.998	1.75E-108	Epi-S1
MRPL33	1.13E-110	0.324407	0.966	0.897	2.06E-106	Epi-S1
DSG1	2.45E-106	0.422958	0.719	0.464	4.46E-102	Epi-S1

MYH14	1.08E-105	0.383246	0.587	0.333	1.96E-101	Epi-S1
TRIM36	2.02E-99	0.333803	0.636	0.37	3.67E-95	Epi-S1
C8orf47	1.22E-93	0.306208	0.943	0.737	2.22E-89	Epi-S1
ENO1	2.31E-93	0.283237	0.997	0.994	4.20E-89	Epi-S1
UPK1B	3.16E-91	0.278139	0.937	0.707	5.76E-87	Epi-S1
LAMB3	5.53E-90	0.337851	0.47	0.246	1.01E-85	Epi-S1
ENSG0000019872	4.68E-88	0.359963	1	0.993	8.51E-84	Epi-S1
S100A4	2.75E-87	0.256706	1	0.995	5.01E-83	Epi-S1
TNFAIP3	6.22E-87	0.460954	0.494	0.283	1.13E-82	Epi-S1
PSAT1	6.65E-87	0.368723	0.735	0.543	1.21E-82	Epi-S1
CLCA2	7.74E-87	0.354895	0.648	0.425	1.41E-82	Epi-S1
RB1	1.06E-86	0.269867	0.777	0.497	1.93E-82	Epi-S1
CLDN1	3.49E-86	0.36778	0.775	0.554	6.36E-82	Epi-S1
CTSL2	2.36E-85	0.274016	0.957	0.619	4.30E-81	Epi-S1
NDUFA4L2	6.62E-85	0.341703	0.806	0.639	1.21E-80	Epi-S1
S100A10	5.86E-81	0.279197	0.973	0.948	1.07E-76	Epi-S1
MGARP	7.13E-79	0.3037	0.972	0.812	1.30E-74	Epi-S1
YBX3	3.06E-78	0.25847	0.936	0.918	5.57E-74	Epi-S1
ENSG0000019893	1.12E-77	0.348926	1	0.997	2.03E-73	Epi-S1
ENSG0000019878	7.81E-76	0.351654	0.997	0.973	1.42E-71	Epi-S1
DKK3	1.73E-75	0.317446	0.828	0.627	3.16E-71	Epi-S1
PIR	2.47E-73	0.26713	0.714	0.511	4.49E-69	Epi-S1
SERPINB5	7.95E-68	0.318771	0.901	0.705	1.45E-63	Epi-S1
TRIM29	2.43E-67	0.27898	0.938	0.762	4.42E-63	Epi-S1
TSPAN1	3.59E-67	0.277511	0.711	0.521	6.54E-63	Epi-S1
CAPG	8.55E-67	0.262634	0.892	0.823	1.56E-62	Epi-S1
ASPH	4.37E-64	0.289902	0.913	0.836	7.96E-60	Epi-S1
FRMD4B	6.34E-63	0.294722	0.531	0.344	1.15E-58	Epi-S1
ARHGEF3	3.87E-62	0.272454	0.523	0.338	7.06E-58	Epi-S1
PHLDA3	2.77E-61	0.25714	0.865	0.741	5.04E-57	Epi-S1
MT1G	4.63E-61	0.40507	0.452	0.267	8.43E-57	Epi-S1
LGALS7	1.08E-60	0.260178	0.502	0.306	1.97E-56	Epi-S1
SPINK5	3.55E-58	0.28661	0.43	0.257	6.46E-54	Epi-S1
CKAP4	1.37E-57	0.287172	0.602	0.424	2.50E-53	Epi-S1
SCD5	3.27E-57	0.258806	0.638	0.439	5.95E-53	Epi-S1
CTSC	5.35E-57	0.274759	0.748	0.561	9.73E-53	Epi-S1
JUP	8.20E-57	0.308298	0.821	0.645	1.49E-52	Epi-S1
NUP93	2.50E-53	0.25499	0.488	0.317	4.55E-49	Epi-S1
SIK1	1.27E-50	0.403325	0.61	0.448	2.32E-46	Epi-S1
DMRTA2	1.83E-50	0.266102	0.372	0.217	3.34E-46	Epi-S1
MARCKS	9.67E-49	0.29405	0.601	0.454	1.76E-44	Epi-S1
CDH1	8.11E-44	0.318081	0.389	0.244	1.48E-39	Epi-S1
IRF6	7.89E-43	0.270103	0.643	0.493	1.44E-38	Epi-S1
WNK1	1.23E-42	0.252071	0.422	0.28	2.25E-38	Epi-S1
PPL	4.99E-39	0.277308	0.457	0.318	9.09E-35	Epi-S1
GPNMB	8.06E-39	0.361344	0.658	0.54	1.47E-34	Epi-S1
RHOB	1.42E-38	0.269255	0.853	0.766	2.58E-34	Epi-S1
RAP2B	2.36E-36	0.283279	0.532	0.391	4.30E-32	Epi-S1

MALAT1	1.88E-35	0.327476	1	0.998	3.43E-31	Epi-S1
NEAT1	3.75E-33	0.388641	0.96	0.93	6.83E-29	Epi-S1
DSG3	5.91E-33	0.260317	0.289	0.172	1.08E-28	Epi-S1
ENSG0000021290	1.25E-30	0.259722	0.889	0.834	2.27E-26	Epi-S1
MBD2	2.75E-30	0.272572	0.386	0.269	5.00E-26	Epi-S1
RND3	1.60E-27	0.342187	0.713	0.609	2.91E-23	Epi-S1
FOSB	8.28E-27	0.355357	0.875	0.827	1.51E-22	Epi-S1
JUND	2.86E-18	0.495937	0.705	0.634	5.21E-14	Epi-S1
NR4A1	1.13E-16	0.297666	0.519	0.447	2.06E-12	Epi-S1
EGR1	3.08E-13	0.27295	0.901	0.862	5.60E-09	Epi-S1
KRT15	0	1.85377	0.872	0.401	0	Epi-T
RPS18	1.86E-289	0.533636	1	1	3.39E-285	Epi-T
RPS6	7.70E-271	0.516318	0.999	0.999	1.40E-266	Epi-T
RPS14	5.16E-250	0.400304	1	1	9.39E-246	Epi-T
RPL10	1.47E-247	0.448496	1	1	2.67E-243	Epi-T
RPL13	6.01E-246	0.492865	0.999	0.999	1.09E-241	Epi-T
RPL12	2.89E-245	0.498371	0.998	0.998	5.26E-241	Epi-T
RPL41	3.18E-240	0.350663	1	1	5.79E-236	Epi-T
RPLP1	7.09E-234	0.434466	1	1	1.29E-229	Epi-T
RPL32	1.16E-233	0.435756	0.998	0.999	2.12E-229	Epi-T
NUPR1	1.54E-223	0.705847	0.96	0.67	2.81E-219	Epi-T
CA12	5.27E-220	0.396756	0.42	0.101	9.60E-216	Epi-T
RPS15A	1.39E-219	0.467822	0.998	0.997	2.54E-215	Epi-T
RPL39	1.69E-211	0.384705	0.998	0.999	3.08E-207	Epi-T
AQP3	2.66E-211	0.731519	0.979	0.645	4.84E-207	Epi-T
RPL26	5.69E-210	0.388186	0.998	0.999	1.04E-205	Epi-T
RPL36	1.65E-209	0.386777	0.996	0.997	3.01E-205	Epi-T
RPS15	1.96E-208	0.337344	1	0.999	3.58E-204	Epi-T
RPS3A	6.13E-207	0.408202	0.999	0.998	1.12E-202	Epi-T
RPL10A	6.14E-207	0.406092	0.999	0.995	1.12E-202	Epi-T
FMO1	3.67E-205	0.53705	0.514	0.156	6.68E-201	Epi-T
RPL21	7.84E-204	0.386025	1	0.999	1.43E-199	Epi-T
RPL35	1.65E-202	0.363917	0.999	0.999	3.00E-198	Epi-T
RPL34	6.49E-202	0.387399	1	1	1.18E-197	Epi-T
RPL8	1.67E-201	0.399753	0.998	0.998	3.05E-197	Epi-T
RPL18A	5.82E-199	0.442175	0.997	0.997	1.06E-194	Epi-T
RPS19	4.02E-198	0.480441	0.998	0.998	7.32E-194	Epi-T
RPS28	1.19E-194	0.378678	1	0.999	2.18E-190	Epi-T
NDUFA4L2	1.91E-194	0.744605	0.929	0.635	3.48E-190	Epi-T
RPS27	7.65E-192	0.37741	1	1	1.39E-187	Epi-T
RPL11	8.51E-187	0.343048	0.999	0.999	1.55E-182	Epi-T
RPS13	3.11E-185	0.334204	1	0.998	5.67E-181	Epi-T
RPL29	7.26E-184	0.438326	0.995	0.992	1.32E-179	Epi-T
GPNMB	1.94E-183	0.639859	0.871	0.529	3.53E-179	Epi-T
RPS2	4.25E-182	0.447149	0.997	0.997	7.74E-178	Epi-T
LY6D	1.26E-180	1.003864	0.77	0.413	2.29E-176	Epi-T
RPL35A	1.63E-177	0.314706	1	0.998	2.96E-173	Epi-T
BTG1	2.88E-176	0.506623	0.995	0.95	5.25E-172	Epi-T

MT1X	6.40E-176	0.58486	1	0.975	1.17E-171	Epi-T
RPL27A	6.78E-172	0.344214	0.998	0.998	1.23E-167	Epi-T
RPL15	8.14E-172	0.287259	0.999	0.998	1.48E-167	Epi-T
RPL36A	2.37E-170	0.422248	0.994	0.989	4.31E-166	Epi-T
ARL4A	5.40E-169	0.560809	0.901	0.596	9.83E-165	Epi-T
RPL13A	2.80E-161	0.359479	1	0.999	5.10E-157	Epi-T
CFD	5.41E-161	0.645025	0.704	0.336	9.84E-157	Epi-T
RPS8	2.57E-159	0.387591	0.998	0.999	4.67E-155	Epi-T
RPS7	1.99E-157	0.34595	1	0.993	3.63E-153	Epi-T
DEFB1	1.49E-156	0.632673	0.742	0.378	2.71E-152	Epi-T
RPS4X	1.98E-155	0.38913	1	0.999	3.60E-151	Epi-T
RPS25	1.08E-154	0.291722	0.999	0.998	1.97E-150	Epi-T
RPL31	3.11E-154	0.338174	0.999	0.998	5.66E-150	Epi-T
RPL23A	5.47E-153	0.301222	0.999	0.998	9.97E-149	Epi-T
RPL3	8.05E-153	0.345265	0.999	0.999	1.47E-148	Epi-T
RPS9	3.83E-151	0.334901	0.998	0.998	6.97E-147	Epi-T
RPS10	1.11E-147	0.414551	0.988	0.978	2.01E-143	Epi-T
SLPI	1.96E-147	0.514974	0.818	0.434	3.57E-143	Epi-T
B2M	3.78E-147	0.418873	1	0.997	6.89E-143	Epi-T
LGALS7B	1.65E-146	0.568864	0.64	0.299	3.00E-142	Epi-T
RPL5	5.39E-145	0.363858	0.997	0.993	9.82E-141	Epi-T
RPL37A	5.14E-144	0.336793	0.998	0.998	9.35E-140	Epi-T
RPS29	7.88E-143	0.337917	0.995	0.996	1.44E-138	Epi-T
S100A16	8.97E-143	0.58301	0.902	0.709	1.63E-138	Epi-T
MSS51	1.01E-141	0.381979	0.991	0.978	1.84E-137	Epi-T
RPL30	7.32E-141	0.325678	0.999	0.998	1.33E-136	Epi-T
RPS17L	1.27E-140	0.335957	0.996	0.996	2.32E-136	Epi-T
RPL19	6.47E-139	0.296679	1	0.998	1.18E-134	Epi-T
RPS14P3	1.30E-136	0.383411	0.984	0.96	2.37E-132	Epi-T
NACA	2.78E-136	0.297852	0.999	0.996	5.07E-132	Epi-T
KRT14	5.31E-133	1.061753	0.834	0.562	9.66E-129	Epi-T
RPL14	1.57E-131	0.307195	0.998	0.995	2.86E-127	Epi-T
PTN	7.43E-130	0.435235	0.298	0.08	1.35E-125	Epi-T
RPS24	3.30E-128	0.322268	0.998	0.998	6.01E-124	Epi-T
RPL28	4.45E-127	0.375567	0.998	0.992	8.10E-123	Epi-T
EEF1G	1.54E-126	0.347449	0.995	0.988	2.80E-122	Epi-T
RPL37	6.98E-125	0.347675	0.996	0.995	1.27E-120	Epi-T
LGALS7	1.06E-124	0.490982	0.614	0.304	1.93E-120	Epi-T
RPL18	5.41E-123	0.316864	0.998	0.996	9.84E-119	Epi-T
CLEC2B	2.18E-122	0.514903	0.885	0.624	3.97E-118	Epi-T
RPS20	2.66E-119	0.287736	0.996	0.997	4.84E-115	Epi-T
RPS3	1.00E-117	0.321536	0.996	0.996	1.83E-113	Epi-T
HLA-B	1.43E-117	0.475572	0.985	0.921	2.60E-113	Epi-T
GAS5	4.97E-117	0.418932	0.981	0.931	9.05E-113	Epi-T
RPL17	1.04E-116	0.360363	0.988	0.985	1.90E-112	Epi-T
RPS12	9.93E-116	0.359366	0.998	0.999	1.81E-111	Epi-T
RPL7	1.30E-114	0.300045	0.998	0.999	2.36E-110	Epi-T
RPS21	7.57E-114	0.308008	0.995	0.989	1.38E-109	Epi-T

RPL27	1.52E-108	0.27271	0.998	0.994	2.77E-104	Epi-T
CSRP2	1.59E-103	0.463047	0.993	0.874	2.89E-99	Epi-T
DEGS1	1.74E-100	0.475418	0.636	0.361	3.16E-96	Epi-T
MGST1	3.42E-100	0.607614	0.498	0.239	6.23E-96	Epi-T
RPS23	2.23E-99	0.256535	1	0.998	4.07E-95	Epi-T
FABP5	6.28E-97	0.45159	0.997	0.941	1.14E-92	Epi-T
RPLP2	3.55E-96	0.277335	1	0.999	6.46E-92	Epi-T
RPL6	2.32E-95	0.295941	0.995	0.991	4.23E-91	Epi-T
KRT13	2.95E-94	0.481467	0.854	0.639	5.37E-90	Epi-T
RPL7A	2.08E-93	0.316903	0.997	0.987	3.79E-89	Epi-T
GPX2	1.89E-91	0.476009	0.63	0.37	3.43E-87	Epi-T
RPL4	3.65E-90	0.291869	0.992	0.986	6.64E-86	Epi-T
HLA-C	9.46E-89	0.361577	0.987	0.954	1.72E-84	Epi-T
FOXP1	6.84E-86	0.290043	0.974	0.943	1.25E-81	Epi-T
PSAT1	4.55E-85	0.440507	0.776	0.546	8.28E-81	Epi-T
RPS11	5.24E-85	0.298436	0.997	0.984	9.54E-81	Epi-T
BAG1	6.65E-85	0.304736	0.987	0.899	1.21E-80	Epi-T
L32131	1.54E-84	0.383563	0.872	0.736	2.80E-80	Epi-T
IGFBP3	2.32E-84	0.488569	0.437	0.192	4.22E-80	Epi-T
RPL23	5.84E-83	0.267787	0.994	0.984	1.06E-78	Epi-T
RPS5	1.91E-82	0.276419	0.995	0.989	3.48E-78	Epi-T
PCP4L1	5.15E-82	0.306499	0.475	0.231	9.37E-78	Epi-T
HIST2H2AA4	4.64E-81	0.286156	0.91	0.705	8.45E-77	Epi-T
GAPDH	1.66E-78	0.279475	1	0.998	3.03E-74	Epi-T
ALDH2	2.57E-78	0.317988	0.627	0.366	4.68E-74	Epi-T
KRT5	8.55E-78	0.373143	1	0.874	1.56E-73	Epi-T
IMPA2	3.42E-77	0.294254	0.509	0.27	6.22E-73	Epi-T
RPLP0	9.88E-77	0.317748	0.998	0.992	1.80E-72	Epi-T
PABPC1	2.01E-74	0.356252	0.964	0.898	3.66E-70	Epi-T
RPS16	3.98E-74	0.259011	0.997	0.994	7.26E-70	Epi-T
EVA1A	1.47E-70	0.255356	0.392	0.184	2.68E-66	Epi-T
DBI	2.25E-68	0.319183	0.971	0.917	4.10E-64	Epi-T
GLTSCR2	4.08E-68	0.30403	0.914	0.822	7.44E-64	Epi-T
TMSB4X	3.64E-65	0.273734	1	0.998	6.64E-61	Epi-T
GNB2L1	1.08E-64	0.254934	0.991	0.99	1.96E-60	Epi-T
MIF	4.89E-62	0.258739	0.996	0.993	8.91E-58	Epi-T
MGST2	1.35E-60	0.288486	0.876	0.718	2.47E-56	Epi-T
EPAS1	3.61E-59	0.30737	0.497	0.293	6.57E-55	Epi-T
S100A2	4.87E-59	0.826011	0.629	0.416	8.87E-55	Epi-T
TPT1	2.94E-58	0.256837	0.997	0.997	5.35E-54	Epi-T
RHOV	5.47E-58	0.31621	0.872	0.626	9.96E-54	Epi-T
NPM1	2.10E-55	0.268715	0.971	0.925	3.83E-51	Epi-T
SLC2A1	1.11E-54	0.303864	0.942	0.854	2.02E-50	Epi-T
VAV3	1.44E-53	0.286727	0.623	0.416	2.62E-49	Epi-T
C17orf76-AS1	2.32E-53	0.296627	0.978	0.963	4.22E-49	Epi-T
TMEM123	6.57E-51	0.30663	0.741	0.573	1.20E-46	Epi-T
POLR1D	9.25E-51	0.253164	0.922	0.822	1.68E-46	Epi-T
ADM	3.09E-49	0.320574	0.967	0.884	5.63E-45	Epi-T

SCGB2A1	5.40E-49	0.400859	0.655	0.432	9.84E-45	Epi-T
HLA-A	1.84E-47	0.298486	0.983	0.96	3.35E-43	Epi-T
MT1G	1.88E-46	0.531682	0.458	0.273	3.42E-42	Epi-T
TXN	1.75E-44	0.313808	0.987	0.981	3.19E-40	Epi-T
SNORA33	4.97E-44	0.266652	0.851	0.761	9.04E-40	Epi-T
CCND1	8.77E-42	0.338999	0.795	0.656	1.60E-37	Epi-T
CBR1	1.18E-39	0.303266	0.822	0.681	2.15E-35	Epi-T
TXNIP	4.69E-37	0.256707	0.803	0.667	8.53E-33	Epi-T
S100A14	5.19E-34	0.259086	0.938	0.768	9.46E-30	Epi-T
NEAT1	5.42E-30	0.277682	0.946	0.932	9.86E-26	Epi-T
SFN	1.46E-29	0.34585	0.995	0.868	2.65E-25	Epi-T
CRIP1	4.33E-19	0.394096	0.778	0.75	7.89E-15	Epi-T
MYC	1.59E-16	0.254205	0.656	0.56	2.90E-12	Epi-T
SPRR1B	0	1.796826	0.439	0.053	0	Conj-2
SPRR2A	0	1.684953	0.392	0.053	0	Conj-2
KRT13	0	1.648261	0.985	0.632	0	Conj-2
SPRR1A	0	1.58991	0.501	0.075	0	Conj-2
SLPI	0	1.491691	0.962	0.429	0	Conj-2
AQP5	0	1.335695	0.659	0.18	0	Conj-2
CXCL17	0	1.104292	0.885	0.375	0	Conj-2
S100A11	0	1.042838	1	0.985	0	Conj-2
FAM3B	0	0.787783	0.661	0.143	0	Conj-2
PITX1	0	0.619437	0.476	0.09	0	Conj-2
AQP3	5.07E-301	1.049623	0.994	0.647	9.22E-297	Conj-2
CSTA	8.31E-301	1.35931	0.984	0.75	1.51E-296	Conj-2
F3	6.90E-290	1.058783	0.701	0.22	1.26E-285	Conj-2
LCN2	5.79E-286	1.15106	0.655	0.195	1.05E-281	Conj-2
SERPINB2	1.93E-259	1.541231	0.453	0.097	3.51E-255	Conj-2
FAM3D	5.82E-255	0.663844	0.609	0.177	1.06E-250	Conj-2
FAM213A	5.82E-240	0.698542	0.781	0.317	1.06E-235	Conj-2
DEFB1	3.03E-238	0.906303	0.813	0.377	5.52E-234	Conj-2
S100A8	3.25E-232	2.364239	0.772	0.397	5.93E-228	Conj-2
S100A16	4.50E-230	0.808188	0.945	0.708	8.19E-226	Conj-2
APOBEC3A	1.53E-229	1.900504	0.724	0.322	2.78E-225	Conj-2
MDK	1.16E-225	0.667017	0.699	0.259	2.11E-221	Conj-2
BARX2	6.40E-222	0.452283	0.465	0.11	1.17E-217	Conj-2
BAALC	1.96E-219	0.521325	0.457	0.111	3.57E-215	Conj-2
IVL	2.13E-218	0.42342	0.32	0.055	3.88E-214	Conj-2
KLK11	7.50E-217	0.720902	0.743	0.331	1.37E-212	Conj-2
HSPB1	2.40E-213	0.620557	1	0.984	4.37E-209	Conj-2
TMSB4X	3.85E-213	0.52484	1	0.998	7.02E-209	Conj-2
HOPX	1.64E-211	0.85455	0.91	0.54	2.99E-207	Conj-2
S100A9	1.02E-208	1.88624	0.742	0.381	1.86E-204	Conj-2
HIST2H2AA4	1.36E-208	0.878399	0.957	0.704	2.47E-204	Conj-2
KRT6A	2.28E-204	1.483632	0.425	0.109	4.16E-200	Conj-2
RPL41	1.11E-190	0.334041	1	1	2.03E-186	Conj-2
POLR2J2	1.23E-189	0.746871	1	0.874	2.24E-185	Conj-2
KRT23	3.28E-189	0.582906	0.284	0.049	5.96E-185	Conj-2

POLR2J3	3.75E-189	0.77105	0.999	0.866	6.83E-185	Conj-2
PHLDA2	3.38E-188	0.831203	0.998	0.898	6.15E-184	Conj-2
B2M	3.84E-184	0.571309	0.999	0.997	6.99E-180	Conj-2
TMEM45B	7.59E-172	0.261003	0.307	0.061	1.38E-167	Conj-2
ISG20	4.47E-171	0.783585	0.451	0.139	8.14E-167	Conj-2
IL1RN	4.28E-165	1.060239	0.84	0.544	7.79E-161	Conj-2
HIST1H2AC	6.14E-160	0.442196	0.513	0.164	1.12E-155	Conj-2
C19orf33	5.70E-159	0.562721	0.997	0.844	1.04E-154	Conj-2
SULT2B1	4.42E-158	0.596992	0.665	0.287	8.04E-154	Conj-2
HS3ST1	1.54E-156	0.697533	0.459	0.151	2.80E-152	Conj-2
PDZK1IP1	2.02E-152	0.335412	0.288	0.062	3.67E-148	Conj-2
EIF6	1.86E-151	0.531823	0.925	0.762	3.39E-147	Conj-2
AREG	2.97E-151	1.376118	0.762	0.425	5.41E-147	Conj-2
SCGB2A1	9.56E-149	0.913916	0.781	0.426	1.74E-144	Conj-2
SFN	3.48E-146	0.789475	1	0.869	6.33E-142	Conj-2
TMSB10	4.60E-145	0.43795	0.999	0.997	8.38E-141	Conj-2
SAT1	5.46E-144	0.599987	0.999	0.99	9.94E-140	Conj-2
RPS29	5.27E-143	0.386363	0.999	0.996	9.60E-139	Conj-2
RHCG	1.05E-142	0.667088	0.31	0.074	1.92E-138	Conj-2
TMEM40	1.05E-141	0.493629	0.667	0.319	1.92E-137	Conj-2
HIST1H2BK	1.19E-141	0.418774	0.485	0.165	2.16E-137	Conj-2
KRT4	1.17E-140	1.144315	0.505	0.182	2.14E-136	Conj-2
RPS18	3.92E-139	0.366238	1	1	7.13E-135	Conj-2
CFD	1.23E-138	0.618997	0.706	0.339	2.25E-134	Conj-2
LOC645638	3.96E-138	0.877372	0.511	0.187	7.20E-134	Conj-2
MYL6	6.12E-138	0.386593	0.999	0.987	1.11E-133	Conj-2
CDKN2B	1.20E-137	0.568566	0.595	0.264	2.18E-133	Conj-2
TXN	5.67E-136	0.742685	0.999	0.98	1.03E-131	Conj-2
RAC1	1.18E-134	0.405459	0.967	0.954	2.15E-130	Conj-2
BAG1	1.13E-133	0.515906	0.968	0.901	2.06E-129	Conj-2
FABP5	6.92E-130	0.616748	0.999	0.941	1.26E-125	Conj-2
FXYD3	1.18E-124	0.436997	0.999	0.857	2.14E-120	Conj-2
CLIC1	1.54E-123	0.443943	0.956	0.911	2.81E-119	Conj-2
CDKN2A	2.19E-122	0.3334	0.253	0.058	3.99E-118	Conj-2
PRSS22	1.82E-117	0.394542	0.441	0.159	3.31E-113	Conj-2
BTG1	3.15E-117	0.665266	0.971	0.952	5.74E-113	Conj-2
DBI	5.38E-117	0.560089	0.944	0.919	9.79E-113	Conj-2
SMIM5	8.02E-117	0.591302	0.586	0.257	1.46E-112	Conj-2
S100A14	8.21E-115	0.619632	0.965	0.767	1.49E-110	Conj-2
MUC20	2.87E-114	0.458317	0.695	0.351	5.22E-110	Conj-2
RPS28	7.81E-113	0.305487	1	0.999	1.42E-108	Conj-2
TACSTD2	1.84E-112	0.534851	1	0.896	3.36E-108	Conj-2
HIST1H2BC	2.65E-111	0.456043	0.379	0.123	4.83E-107	Conj-2
RPS14	1.32E-109	0.263647	0.999	1	2.40E-105	Conj-2
BCAS1	2.22E-109	0.26906	0.329	0.097	4.05E-105	Conj-2
RPL36	9.39E-108	0.317401	0.996	0.998	1.71E-103	Conj-2
RPL10	2.07E-107	0.257041	1	1	3.77E-103	Conj-2
LYPD3	2.49E-107	0.492206	0.992	0.837	4.53E-103	Conj-2

CLDN4	6.55E-105	0.611549	0.911	0.603	1.19E-100	Conj-2
NUPR1	7.22E-105	0.606875	0.895	0.677	1.32E-100	Conj-2
FGFBP1	9.73E-105	0.86015	0.688	0.42	1.77E-100	Conj-2
PLAUR	2.06E-103	1.247657	0.527	0.275	3.74E-99	Conj-2
OSR2	1.66E-102	0.325335	0.578	0.24	3.03E-98	Conj-2
CSTB	2.67E-102	0.84831	0.978	0.936	4.85E-98	Conj-2
EHF	7.03E-102	0.668376	0.603	0.319	1.28E-97	Conj-2
HIST1H2BD	1.53E-101	0.344546	0.405	0.145	2.79E-97	Conj-2
MUC21	2.20E-101	0.522551	0.373	0.131	4.00E-97	Conj-2
LGALS3	8.15E-101	0.394834	0.999	0.988	1.48E-96	Conj-2
C9orf16	1.84E-99	0.40193	0.947	0.854	3.35E-95	Conj-2
CD55	1.07E-97	0.630197	0.768	0.554	1.94E-93	Conj-2
HES4	2.62E-97	0.519763	0.68	0.382	4.77E-93	Conj-2
COX7A1	7.67E-96	0.591908	0.744	0.467	1.40E-91	Conj-2
RPL35	1.24E-93	0.258972	1	0.998	2.26E-89	Conj-2
RPL39	2.56E-93	0.278477	1	0.998	4.66E-89	Conj-2
ELF3	2.63E-91	0.50861	0.98	0.8	4.79E-87	Conj-2
EVA1A	4.61E-91	0.326311	0.441	0.183	8.40E-87	Conj-2
RPL27A	3.14E-90	0.279209	0.999	0.998	5.72E-86	Conj-2
ABRACL	3.54E-89	0.414186	0.669	0.391	6.44E-85	Conj-2
ZNF593	6.10E-89	0.470812	0.587	0.318	1.11E-84	Conj-2
CLEC2B	1.68E-88	0.486696	0.868	0.628	3.05E-84	Conj-2
THRB	3.89E-87	0.544696	0.778	0.573	7.08E-83	Conj-2
CTSS	1.00E-86	0.307207	0.498	0.22	1.83E-82	Conj-2
PRDX6	2.45E-86	0.489136	0.906	0.852	4.45E-82	Conj-2
GSTP1	3.28E-86	0.293775	1	0.996	5.98E-82	Conj-2
IGFBP3	6.93E-86	0.707693	0.443	0.194	1.26E-81	Conj-2
CALML3	1.23E-84	0.566473	0.897	0.69	2.24E-80	Conj-2
MARCKSL1	1.50E-84	0.568842	0.492	0.234	2.74E-80	Conj-2
CDKN1A	2.09E-84	0.482502	0.852	0.683	3.81E-80	Conj-2
CD68	2.65E-84	0.250582	0.254	0.075	4.83E-80	Conj-2
ARPC3	1.36E-83	0.340892	0.922	0.879	2.47E-79	Conj-2
FOSL1	3.11E-83	0.720485	0.549	0.303	5.67E-79	Conj-2
MGST1	8.92E-83	0.462349	0.507	0.241	1.62E-78	Conj-2
CD9	9.42E-83	0.3166	0.988	0.992	1.72E-78	Conj-2
LSP1	5.57E-82	0.388461	0.616	0.312	1.01E-77	Conj-2
H2AFJ	3.34E-81	0.410848	0.767	0.541	6.08E-77	Conj-2
SERPINB1	4.27E-81	0.739567	0.745	0.593	7.78E-77	Conj-2
SLC9A3R1	4.73E-81	0.383747	0.727	0.487	8.61E-77	Conj-2
RPL12	1.54E-79	0.267976	0.998	0.998	2.81E-75	Conj-2
ATP6V1G1	1.63E-78	0.302722	0.967	0.965	2.97E-74	Conj-2
RPS15A	2.79E-78	0.281106	1	0.997	5.08E-74	Conj-2
RPS14P3	7.55E-78	0.303301	0.988	0.96	1.37E-73	Conj-2
KRT17	1.93E-77	1.06818	0.65	0.408	3.51E-73	Conj-2
PCP4L1	6.99E-77	0.321467	0.492	0.232	1.27E-72	Conj-2
BID	1.08E-75	0.355016	0.53	0.274	1.97E-71	Conj-2
SH3BGRL3	1.69E-75	0.484698	0.994	0.95	3.07E-71	Conj-2
A4GALT	6.59E-75	0.402322	0.762	0.515	1.20E-70	Conj-2

GNA15	7.51E-75	0.292247	0.501	0.247	1.37E-70	Conj-2
RPL29	1.82E-74	0.264783	0.998	0.992	3.31E-70	Conj-2
HIST2H2BE	4.95E-74	0.363162	0.501	0.244	9.01E-70	Conj-2
LINC00673	1.66E-72	0.268282	0.373	0.153	3.03E-68	Conj-2
EZR	2.10E-71	0.446299	0.942	0.839	3.83E-67	Conj-2
RARRES3	7.33E-71	0.47828	0.638	0.4	1.33E-66	Conj-2
ZNF385A	2.30E-70	0.326039	0.691	0.441	4.19E-66	Conj-2
UNC5B-AS1	1.23E-69	0.58234	0.541	0.306	2.23E-65	Conj-2
RAB25	1.70E-69	0.360847	0.789	0.574	3.09E-65	Conj-2
TMA7	1.88E-69	0.279312	0.988	0.978	3.43E-65	Conj-2
DSG3	4.28E-69	0.286579	0.391	0.171	7.79E-65	Conj-2
DEGS2	1.02E-68	0.25175	0.262	0.089	1.86E-64	Conj-2
COX8A	2.44E-67	0.318249	0.927	0.845	4.45E-63	Conj-2
RPL36A	3.71E-67	0.303007	0.995	0.989	6.76E-63	Conj-2
RPL24	1.55E-66	0.285392	0.999	0.993	2.82E-62	Conj-2
RAB11FIP1	6.90E-66	0.314116	0.322	0.13	1.26E-61	Conj-2
KLF5	8.83E-66	0.350531	0.882	0.674	1.61E-61	Conj-2
PPDPF	1.00E-65	0.303143	0.993	0.966	1.82E-61	Conj-2
CYB5A	3.31E-65	0.414087	0.684	0.487	6.03E-61	Conj-2
TGM1	4.25E-65	0.28887	0.344	0.146	7.73E-61	Conj-2
GUK1	4.26E-65	0.308191	0.928	0.898	7.76E-61	Conj-2
PFN1	4.97E-61	0.263904	0.995	0.985	9.04E-57	Conj-2
RRAD	8.51E-61	0.380124	0.305	0.123	1.55E-56	Conj-2
BIK	1.29E-60	0.374872	0.526	0.307	2.34E-56	Conj-2
HEBP2	2.08E-60	0.36499	0.763	0.604	3.79E-56	Conj-2
CAST	2.24E-60	0.337061	0.871	0.788	4.08E-56	Conj-2
EHBP1	3.15E-60	0.270136	0.425	0.207	5.74E-56	Conj-2
CLINT1	1.05E-59	0.312817	0.67	0.421	1.91E-55	Conj-2
SERINC2	4.26E-59	0.358301	0.747	0.543	7.75E-55	Conj-2
LRRC8A	1.66E-57	0.28313	0.37	0.174	3.03E-53	Conj-2
ANXA1	3.01E-57	0.56778	0.996	0.983	5.48E-53	Conj-2
MALL	1.00E-56	0.352823	0.849	0.715	1.83E-52	Conj-2
MGST2	2.23E-56	0.347816	0.868	0.72	4.06E-52	Conj-2
SELK	5.74E-55	0.426533	0.916	0.858	1.05E-50	Conj-2
CHMP4B	7.46E-55	0.309641	0.87	0.76	1.36E-50	Conj-2
TPM4	8.77E-55	0.399149	0.666	0.482	1.60E-50	Conj-2
CCDC12	3.58E-53	0.374982	0.769	0.603	6.53E-49	Conj-2
DNAJB1	4.85E-53	0.285567	0.963	0.909	8.83E-49	Conj-2
RHOV	6.03E-53	0.288354	0.928	0.624	1.10E-48	Conj-2
LMO4	6.09E-53	0.360874	0.737	0.567	1.11E-48	Conj-2
ATP6V1F	7.11E-53	0.261141	0.959	0.89	1.29E-48	Conj-2
UQCRQ	1.75E-52	0.254522	0.971	0.949	3.18E-48	Conj-2
CRIP1	1.92E-52	1.000894	0.823	0.748	3.50E-48	Conj-2
HIST1H1C	8.48E-52	0.376581	0.416	0.213	1.54E-47	Conj-2
SMAGP	2.05E-50	0.297119	0.584	0.372	3.73E-46	Conj-2
TM4SF1	5.18E-50	0.977043	0.757	0.682	9.43E-46	Conj-2
SEPW1	5.47E-50	0.31185	0.594	0.38	9.95E-46	Conj-2
ZFAS1	5.60E-50	0.285717	0.922	0.863	1.02E-45	Conj-2

CAPNS2	1.13E-49	0.355851	0.746	0.582	2.05E-45	Conj-2
KRT8	2.87E-48	0.341826	0.605	0.384	5.23E-44	Conj-2
RPS26	4.02E-47	0.268888	0.962	0.942	7.32E-43	Conj-2
PERP	2.52E-46	0.280215	1	0.916	4.59E-42	Conj-2
C9orf169	5.06E-46	0.405935	0.298	0.137	9.21E-42	Conj-2
GPR87	1.05E-45	0.259181	0.518	0.307	1.91E-41	Conj-2
CHMP1B	2.38E-45	0.35649	0.838	0.735	4.33E-41	Conj-2
MAP3K8	6.24E-45	0.36124	0.44	0.255	1.14E-40	Conj-2
SERP1	1.95E-44	0.290099	0.896	0.831	3.55E-40	Conj-2
CRB3	2.82E-44	0.27734	0.426	0.236	5.14E-40	Conj-2
ST14	7.06E-44	0.252045	0.359	0.184	1.28E-39	Conj-2
SYT8	8.19E-44	0.314976	0.939	0.749	1.49E-39	Conj-2
IRF2BP2	1.25E-43	0.313031	0.543	0.342	2.28E-39	Conj-2
UAP1	7.88E-43	0.303799	0.546	0.349	1.43E-38	Conj-2
ETS2	2.36E-42	0.268844	0.553	0.346	4.30E-38	Conj-2
ZFP36L1	3.08E-42	0.32912	0.914	0.843	5.60E-38	Conj-2
POLR1D	6.21E-42	0.261444	0.907	0.824	1.13E-37	Conj-2
NUAK2	2.88E-41	0.413064	0.601	0.407	5.25E-37	Conj-2
HBEGF	5.90E-41	0.290325	0.496	0.306	1.07E-36	Conj-2
SOX15	6.86E-40	0.273561	0.901	0.772	1.25E-35	Conj-2
SRA1	1.07E-39	0.370065	0.639	0.483	1.95E-35	Conj-2
TXNDC17	2.07E-39	0.262721	0.869	0.792	3.77E-35	Conj-2
TUBB2A	3.23E-39	0.324533	0.851	0.762	5.88E-35	Conj-2
ARHGDIB	3.75E-39	0.267194	0.626	0.427	6.83E-35	Conj-2
CYBA	3.95E-39	0.310301	0.703	0.545	7.18E-35	Conj-2
NDRG2	2.24E-38	0.266728	0.537	0.347	4.08E-34	Conj-2
LYPD2	4.22E-38	0.583204	0.279	0.134	7.68E-34	Conj-2
CLIC3	1.42E-37	0.304367	0.346	0.186	2.58E-33	Conj-2
C11orf31	4.29E-37	0.263628	0.867	0.782	7.81E-33	Conj-2
NOTCH2NL	4.59E-37	0.276925	0.702	0.547	8.36E-33	Conj-2
DDIT4	1.08E-36	0.309928	0.643	0.452	1.96E-32	Conj-2
TNNT3	1.17E-36	0.283877	0.628	0.445	2.13E-32	Conj-2
ST3GAL4	1.02E-35	0.2607	0.506	0.325	1.86E-31	Conj-2
PLAU	1.39E-35	0.628505	0.615	0.49	2.52E-31	Conj-2
PMAIP1	1.50E-35	0.336535	0.84	0.697	2.74E-31	Conj-2
MAP2K3	1.60E-35	0.274278	0.621	0.449	2.91E-31	Conj-2
H19	3.38E-35	0.254011	0.269	0.131	6.15E-31	Conj-2
MIDN	6.69E-35	0.320611	0.654	0.485	1.22E-30	Conj-2
PDLIM5	1.34E-34	0.318867	0.443	0.285	2.44E-30	Conj-2
BCL10	2.83E-34	0.271371	0.395	0.234	5.15E-30	Conj-2
KRT10	3.12E-34	0.460016	0.955	0.927	5.68E-30	Conj-2
CKB	1.48E-33	0.255229	0.522	0.345	2.70E-29	Conj-2
EPHA2	2.00E-32	0.319927	0.459	0.297	3.64E-28	Conj-2
FAM46B	2.40E-31	0.268951	0.526	0.357	4.36E-27	Conj-2
HMGA1	1.13E-30	0.425344	0.563	0.426	2.06E-26	Conj-2
OVOL1	3.15E-30	0.289659	0.532	0.354	5.73E-26	Conj-2
GLTP	3.33E-30	0.300852	0.659	0.513	6.07E-26	Conj-2
CYSTM1	2.92E-29	0.360072	0.585	0.457	5.32E-25	Conj-2

PRELID1	5.59E-27	0.321462	0.815	0.775	1.02E-22	Conj-2
ARL4A	1.17E-26	0.337535	0.725	0.61	2.13E-22	Conj-2
USP53	1.56E-25	0.283268	0.286	0.169	2.84E-21	Conj-2
CD24	9.78E-25	0.5396	0.316	0.198	1.78E-20	Conj-2
IFI27	3.55E-24	0.424647	0.649	0.523	6.47E-20	Conj-2
MSLN	6.40E-24	0.261222	0.385	0.25	1.16E-19	Conj-2
TRIM16	2.31E-23	0.251626	0.541	0.408	4.21E-19	Conj-2
CFLAR	7.10E-23	0.285465	0.414	0.293	1.29E-18	Conj-2
PFDN2	1.66E-22	0.27551	0.866	0.817	3.02E-18	Conj-2
GADD45A	1.55E-20	0.482058	0.666	0.598	2.83E-16	Conj-2
TXNIP	3.20E-18	0.30977	0.729	0.673	5.83E-14	Conj-2
LY6D	9.95E-18	0.423783	0.538	0.431	1.81E-13	Conj-2
RAP2B	2.39E-16	0.337631	0.5	0.399	4.35E-12	Conj-2
TNFRSF12A	1.99E-14	0.262372	0.47	0.381	3.63E-10	Conj-2
CLDN1	1.76E-13	0.485315	0.624	0.573	3.21E-09	Conj-2
HES1	5.56E-13	0.282231	0.813	0.755	1.01E-08	Conj-2
KRT24	2.82E-07	0.452764	0.33	0.262	0.005133	Conj-2
MT1G	2.96E-05	0.275192	0.336	0.282	0.539412	Conj-2
KRT24	0	2.916824	0.944	0.227	0	Epi-S3
LYPD2	0	1.863657	0.87	0.101	0	Epi-S3
LOC645638	0	1.847968	0.802	0.172	0	Epi-S3
PSCA	0	1.541166	0.743	0.163	0	Epi-S3
MAL	0	1.280111	0.991	0.437	0	Epi-S3
MSMB	0	1.238666	0.564	0.111	0	Epi-S3
ADIRF	0	1.177503	1	0.985	0	Epi-S3
LYPD3	0	1.011797	1	0.838	0	Epi-S3
CLIC3	0	1.003253	0.783	0.162	0	Epi-S3
LGALS3	0	0.998977	1	0.988	0	Epi-S3
SULT2B1	0	0.977619	0.882	0.276	0	Epi-S3
ASPG	0	0.967246	0.592	0.045	0	Epi-S3
SMIM5	0	0.954378	0.897	0.241	0	Epi-S3
UPK1B	0	0.951006	0.994	0.714	0	Epi-S3
CLDN7	0	0.946088	0.998	0.602	0	Epi-S3
SLURP1	0	0.921053	0.764	0.215	0	Epi-S3
SCEL	0	0.892187	0.9	0.401	0	Epi-S3
FAM3D	0	0.846544	0.741	0.172	0	Epi-S3
MUC21	0	0.824903	0.654	0.117	0	Epi-S3
C9orf169	0	0.764162	0.6	0.12	0	Epi-S3
LGALS9C	0	0.665625	0.589	0.073	0	Epi-S3
EPS8L1	0	0.658543	0.676	0.175	0	Epi-S3
LIPH	0	0.531145	0.604	0.132	0	Epi-S3
CD36	0	0.520629	0.451	0.061	0	Epi-S3
MYEOV	0	0.458269	0.358	0.026	0	Epi-S3
GDPD3	0	0.295225	0.292	0.023	0	Epi-S3
RELL1	2.32E-305	0.897741	0.776	0.26	4.23E-301	Epi-S3
LAD1	7.22E-305	0.792771	0.932	0.448	1.32E-300	Epi-S3
C4orf19	6.86E-300	0.722644	0.797	0.283	1.25E-295	Epi-S3
TACSTD2	9.76E-300	0.766041	1	0.897	1.78E-295	Epi-S3

LOC729966	8.51E-298	0.763058	0.882	0.354	1.55E-293	Epi-S3
MAL2	1.58E-296	0.941333	0.946	0.478	2.88E-292	Epi-S3
GIPC1	1.73E-291	0.802396	0.982	0.688	3.16E-287	Epi-S3
CALM2	1.57E-290	0.707787	1	0.979	2.85E-286	Epi-S3
POLR2J2	1.70E-289	0.779499	1	0.875	3.10E-285	Epi-S3
PPDPF	3.71E-288	0.625825	1	0.966	6.75E-284	Epi-S3
SDC1	3.75E-287	0.686625	1	0.928	6.83E-283	Epi-S3
HM13	5.89E-286	0.819108	0.934	0.572	1.07E-281	Epi-S3
MUC16	6.50E-286	0.372665	0.374	0.054	1.18E-281	Epi-S3
GJB4	2.38E-285	0.882291	0.926	0.443	4.33E-281	Epi-S3
C4orf3	1.09E-275	0.662757	0.997	0.928	1.98E-271	Epi-S3
ADH7	1.21E-274	0.847758	0.983	0.607	2.20E-270	Epi-S3
APOBEC3A	3.05E-267	0.813192	0.855	0.317	5.56E-263	Epi-S3
PRDX5	3.20E-267	0.571486	1	0.975	5.83E-263	Epi-S3
CD24	9.35E-267	0.777802	0.638	0.181	1.70E-262	Epi-S3
MUC20	2.67E-265	0.690756	0.855	0.344	4.86E-261	Epi-S3
KRT3	1.46E-260	1.099835	0.965	0.555	2.67E-256	Epi-S3
PRSS27	5.74E-258	0.305272	0.303	0.039	1.04E-253	Epi-S3
S100A4	1.59E-257	0.524402	1	0.996	2.90E-253	Epi-S3
POLR2J3	2.59E-253	0.781009	1	0.867	4.71E-249	Epi-S3
B3GNT3	1.28E-251	0.352237	0.384	0.064	2.34E-247	Epi-S3
C12orf75	1.75E-251	0.801513	0.963	0.633	3.19E-247	Epi-S3
HOMER2	7.47E-251	0.480054	0.571	0.147	1.36E-246	Epi-S3
CALML5	8.20E-246	1.103203	0.682	0.232	1.49E-241	Epi-S3
MSLN	1.20E-243	0.693136	0.706	0.232	2.19E-239	Epi-S3
PERP	1.97E-241	0.559196	1	0.917	3.58E-237	Epi-S3
ANXA11	4.38E-240	0.689875	0.977	0.762	7.98E-236	Epi-S3
MRPL33	8.77E-239	0.720348	0.997	0.899	1.60E-234	Epi-S3
FXYD3	5.25E-230	0.568691	0.999	0.858	9.57E-226	Epi-S3
GPR110	3.56E-226	0.438294	0.508	0.127	6.48E-222	Epi-S3
DSC2	3.64E-224	0.686089	0.924	0.51	6.63E-220	Epi-S3
IL20RA	6.65E-220	0.717049	0.842	0.407	1.21E-215	Epi-S3
TSTD1	1.02E-219	0.635809	0.989	0.769	1.86E-215	Epi-S3
HRASLS2	1.60E-217	0.619879	0.565	0.168	2.91E-213	Epi-S3
GLRX	2.37E-217	0.703637	0.859	0.424	4.32E-213	Epi-S3
ST3GAL4	1.06E-216	0.63518	0.753	0.312	1.94E-212	Epi-S3
KRT12	1.12E-216	0.84141	0.991	0.819	2.04E-212	Epi-S3
C10orf54	2.24E-216	0.698441	0.966	0.719	4.09E-212	Epi-S3
RAB25	1.41E-215	0.660703	0.946	0.567	2.58E-211	Epi-S3
SCGB2A1	8.98E-213	0.780583	0.876	0.423	1.63E-208	Epi-S3
RB1	1.47E-212	0.844102	0.918	0.502	2.67E-208	Epi-S3
GOLM1	2.20E-211	0.368896	0.37	0.071	4.01E-207	Epi-S3
RHOD	1.58E-209	0.641512	0.847	0.43	2.88E-205	Epi-S3
LGALSL	2.10E-204	0.561885	0.776	0.318	3.83E-200	Epi-S3
LMO7	5.45E-204	0.657393	0.73	0.302	9.92E-200	Epi-S3
ACTG1	2.64E-203	0.517453	1	0.995	4.81E-199	Epi-S3
MSMO1	3.25E-202	0.641961	0.756	0.333	5.93E-198	Epi-S3
HSPB1	1.69E-201	0.588187	1	0.984	3.07E-197	Epi-S3

CLDN4	1.89E-201	0.652101	0.986	0.601	3.44E-197	Epi-S3
PRDX1	1.27E-198	0.458798	0.999	0.987	2.31E-194	Epi-S3
AGR2	8.60E-194	0.806224	0.954	0.631	1.57E-189	Epi-S3
OAS1	1.47E-189	0.635025	0.614	0.226	2.67E-185	Epi-S3
MVK	3.87E-189	0.486178	0.64	0.233	7.04E-185	Epi-S3
CRYAB	2.38E-187	0.586132	0.991	0.875	4.33E-183	Epi-S3
CTSL2	1.44E-186	0.753083	0.992	0.633	2.62E-182	Epi-S3
CSTA	1.26E-185	0.573141	0.987	0.751	2.30E-181	Epi-S3
MUC15	3.97E-184	0.60894	0.852	0.437	7.24E-180	Epi-S3
PVRL4	1.45E-179	0.581795	0.899	0.484	2.64E-175	Epi-S3
SPINT1	5.28E-179	0.582024	0.949	0.633	9.61E-175	Epi-S3
SHMT1	5.53E-178	0.479048	0.676	0.269	1.01E-173	Epi-S3
ULBP2	1.12E-175	0.488174	0.66	0.255	2.03E-171	Epi-S3
PKIB	1.25E-175	0.469502	0.585	0.198	2.28E-171	Epi-S3
CLC	5.80E-175	0.365528	0.429	0.107	1.06E-170	Epi-S3
NQO1	1.35E-172	0.636404	0.999	0.944	2.45E-168	Epi-S3
NUAK2	2.92E-170	0.566003	0.83	0.395	5.33E-166	Epi-S3
SQLE	3.65E-167	0.584186	0.741	0.351	6.64E-163	Epi-S3
PHLDA2	3.86E-165	0.546759	0.999	0.899	7.04E-161	Epi-S3
PLBD1	1.57E-163	0.433404	0.543	0.182	2.86E-159	Epi-S3
SPINT2	3.50E-163	0.460939	1	0.829	6.37E-159	Epi-S3
C19orf33	3.73E-162	0.45409	1	0.845	6.78E-158	Epi-S3
DSG1	1.05E-160	0.536458	0.863	0.468	1.91E-156	Epi-S3
NLN	2.32E-159	0.310443	0.392	0.1	4.23E-155	Epi-S3
ID1	3.73E-159	0.583475	1	0.957	6.79E-155	Epi-S3
MYL12B	1.62E-158	0.374733	0.998	0.972	2.95E-154	Epi-S3
NDUFA4	1.66E-156	0.438416	0.998	0.959	3.03E-152	Epi-S3
ACTB	1.37E-155	0.39757	1	0.999	2.50E-151	Epi-S3
CD55	6.83E-155	0.546935	0.891	0.548	1.24E-150	Epi-S3
RAET1E	2.97E-154	0.369992	0.501	0.159	5.41E-150	Epi-S3
SNCG	2.16E-153	0.59746	0.744	0.354	3.94E-149	Epi-S3
VAMP8	1.62E-152	0.489314	0.969	0.753	2.95E-148	Epi-S3
RAB11A	2.23E-152	0.468158	0.979	0.851	4.06E-148	Epi-S3
S100A6	3.73E-152	0.330728	1	0.999	6.79E-148	Epi-S3
SLC25A3	5.53E-148	0.455625	0.984	0.906	1.01E-143	Epi-S3
HOPX	7.46E-148	0.792045	0.905	0.543	1.36E-143	Epi-S3
C8orf47	8.96E-148	0.539652	0.979	0.745	1.63E-143	Epi-S3
EVPL	1.07E-146	0.430657	0.612	0.241	1.95E-142	Epi-S3
MYO5A	3.64E-145	0.427416	0.491	0.16	6.63E-141	Epi-S3
LMTK3	9.26E-145	0.286006	0.281	0.058	1.69E-140	Epi-S3
EMP1	9.45E-144	0.524655	0.997	0.921	1.72E-139	Epi-S3
SRP68	1.57E-143	0.53073	0.843	0.522	2.86E-139	Epi-S3
CYP26A1	1.93E-143	0.582105	0.438	0.129	3.51E-139	Epi-S3
TRIM16	4.75E-140	0.542115	0.751	0.397	8.64E-136	Epi-S3
KRT27	5.16E-138	0.447667	0.41	0.124	9.40E-134	Epi-S3
GGT6	2.78E-137	0.356546	0.514	0.179	5.07E-133	Epi-S3
CD9	5.17E-137	0.379686	1	0.991	9.42E-133	Epi-S3
CD82	1.10E-135	0.50491	0.856	0.54	2.00E-131	Epi-S3

TUBA4A	5.89E-135	0.505129	0.967	0.734	1.07E-130	Epi-S3
HSP90AA1	4.51E-134	0.409464	0.998	0.974	8.21E-130	Epi-S3
C1orf116	6.87E-134	0.377635	0.6	0.244	1.25E-129	Epi-S3
ATP6V0B	1.30E-133	0.424378	0.986	0.867	2.37E-129	Epi-S3
IL1RN	6.90E-133	0.356315	0.916	0.541	1.26E-128	Epi-S3
CRB3	6.97E-133	0.377063	0.581	0.228	1.27E-128	Epi-S3
TRIM29	1.82E-132	0.459285	0.989	0.767	3.31E-128	Epi-S3
FGFBP1	1.15E-130	0.57894	0.783	0.416	2.09E-126	Epi-S3
EIF6	4.91E-130	0.457918	0.951	0.761	8.95E-126	Epi-S3
CCDC64B	1.01E-129	0.376158	0.559	0.215	1.83E-125	Epi-S3
FBP1	4.39E-129	0.271592	0.355	0.096	7.98E-125	Epi-S3
DAPL1	1.84E-128	0.490481	0.997	0.829	3.34E-124	Epi-S3
LINC00937	4.99E-127	0.368685	0.521	0.186	9.09E-123	Epi-S3
MYH14	5.15E-127	0.435248	0.706	0.338	9.37E-123	Epi-S3
NDUFV2	3.46E-126	0.415364	0.969	0.828	6.29E-122	Epi-S3
COX6A1	2.40E-125	0.339077	1	0.976	4.37E-121	Epi-S3
DENND2D	8.26E-125	0.308782	0.483	0.172	1.50E-120	Epi-S3
COX7A2	1.45E-124	0.322899	0.999	0.975	2.63E-120	Epi-S3
RCBTB2	2.92E-124	0.417164	0.642	0.287	5.31E-120	Epi-S3
PDCD4	4.49E-123	0.490365	0.929	0.699	8.17E-119	Epi-S3
TMSB4X	8.41E-121	0.373911	1	0.998	1.53E-116	Epi-S3
GALNT3	4.08E-120	0.425622	0.654	0.303	7.43E-116	Epi-S3
PPP1R14C	2.76E-119	0.396575	0.725	0.365	5.03E-115	Epi-S3
TPD52	1.22E-118	0.447089	0.93	0.661	2.23E-114	Epi-S3
COX6C	3.08E-118	0.33672	0.993	0.966	5.61E-114	Epi-S3
ANXA2	1.52E-117	0.292666	1	0.994	2.77E-113	Epi-S3
GPR115	1.57E-116	0.349171	0.491	0.184	2.86E-112	Epi-S3
CNGA1	5.34E-115	0.291272	0.413	0.132	9.72E-111	Epi-S3
FDFT1	5.32E-114	0.443196	0.786	0.465	9.69E-110	Epi-S3
SH2D4A	6.04E-114	0.375076	0.603	0.271	1.10E-109	Epi-S3
MFSD4	4.33E-113	0.38736	0.635	0.294	7.89E-109	Epi-S3
AGPAT2	1.21E-111	0.481798	0.841	0.577	2.21E-107	Epi-S3
MRPS10	3.90E-109	0.458027	0.882	0.623	7.09E-105	Epi-S3
SLC12A8	2.13E-108	0.350038	0.407	0.143	3.88E-104	Epi-S3
TM7SF2	3.42E-107	0.298203	0.471	0.174	6.23E-103	Epi-S3
B4GALT5	6.74E-107	0.336394	0.5	0.197	1.23E-102	Epi-S3
FA2H	5.86E-106	0.425192	0.701	0.352	1.07E-101	Epi-S3
NAPRT1	2.40E-105	0.402828	0.788	0.475	4.36E-101	Epi-S3
EZR	7.84E-104	0.404583	0.994	0.836	1.43E-99	Epi-S3
ZFAND6	8.44E-104	0.412734	0.857	0.614	1.54E-99	Epi-S3
TP53I3	1.07E-102	0.40267	0.801	0.484	1.95E-98	Epi-S3
BID	1.29E-102	0.356425	0.589	0.272	2.34E-98	Epi-S3
TMSB10	1.69E-101	0.306353	0.999	0.997	3.09E-97	Epi-S3
BNIPL	2.06E-101	0.388101	0.683	0.361	3.76E-97	Epi-S3
GSTP1	7.09E-100	0.310878	1	0.996	1.29E-95	Epi-S3
DAAM1	1.14E-99	0.494989	0.898	0.625	2.08E-95	Epi-S3
MGARP	7.98E-99	0.442636	0.981	0.819	1.45E-94	Epi-S3
PMVK	8.11E-99	0.390908	0.937	0.724	1.48E-94	Epi-S3

COX7B	1.32E-98	0.332559	0.995	0.933	2.40E-94	Epi-S3
PPL	3.79E-98	0.35544	0.641	0.314	6.90E-94	Epi-S3
CHMP4B	4.75E-98	0.367212	0.949	0.756	8.64E-94	Epi-S3
SH3BGRL3	2.62E-97	0.348819	0.998	0.95	4.77E-93	Epi-S3
EML2	1.20E-96	0.380143	0.744	0.435	2.18E-92	Epi-S3
ABHD11	1.75E-96	0.383805	0.664	0.35	3.19E-92	Epi-S3
RAC1	2.04E-96	0.319928	0.994	0.952	3.72E-92	Epi-S3
COX5A	2.04E-96	0.337956	0.977	0.856	3.72E-92	Epi-S3
ELF3	2.07E-96	0.429251	0.998	0.8	3.77E-92	Epi-S3
SERF2	1.15E-95	0.2747	0.999	0.991	2.09E-91	Epi-S3
GNB2	5.36E-95	0.360747	0.963	0.836	9.75E-91	Epi-S3
CSTB	7.72E-95	0.315387	0.994	0.935	1.41E-90	Epi-S3
CLU	2.20E-94	0.374014	1	0.991	4.01E-90	Epi-S3
PIR	6.91E-94	0.404678	0.825	0.514	1.26E-89	Epi-S3
DSP	8.23E-94	0.395924	0.974	0.749	1.50E-89	Epi-S3
GADD45B	2.28E-92	0.424627	0.985	0.925	4.16E-88	Epi-S3
NOP10	3.24E-92	0.357402	0.932	0.757	5.90E-88	Epi-S3
S100A14	3.02E-91	0.390712	0.986	0.767	5.50E-87	Epi-S3
MBD2	4.54E-91	0.333448	0.561	0.265	8.26E-87	Epi-S3
CALM1	1.14E-90	0.346809	0.994	0.959	2.08E-86	Epi-S3
BCL2L13	3.91E-90	0.387185	0.953	0.756	7.12E-86	Epi-S3
PPIA	4.13E-90	0.336736	0.994	0.958	7.52E-86	Epi-S3
SDR16C5	4.97E-90	0.352966	0.661	0.343	9.05E-86	Epi-S3
SH3GL3	9.19E-89	0.268375	0.421	0.16	1.67E-84	Epi-S3
PHLDB3	1.01E-88	0.283448	0.403	0.154	1.83E-84	Epi-S3
BOLA2	1.40E-88	0.35582	0.866	0.625	2.55E-84	Epi-S3
CCDC12	3.02E-88	0.360923	0.864	0.599	5.50E-84	Epi-S3
AP1M2	3.37E-87	0.320825	0.603	0.307	6.14E-83	Epi-S3
RER1	7.65E-87	0.356888	0.936	0.741	1.39E-82	Epi-S3
PSAP	2.21E-86	0.381609	0.998	0.951	4.03E-82	Epi-S3
TUBB4B	2.25E-86	0.338973	0.995	0.918	4.10E-82	Epi-S3
MAPK13	1.11E-85	0.315807	0.574	0.279	2.02E-81	Epi-S3
PGD	1.98E-85	0.380888	0.832	0.57	3.60E-81	Epi-S3
ATP6V1E1	2.73E-85	0.369207	0.91	0.728	4.97E-81	Epi-S3
ARPC2	3.50E-85	0.30991	0.993	0.941	6.37E-81	Epi-S3
SPHK1	6.25E-85	0.26553	0.46	0.187	1.14E-80	Epi-S3
C6orf132	7.76E-85	0.309685	0.633	0.326	1.41E-80	Epi-S3
AMPD3	1.05E-84	0.321258	0.486	0.216	1.92E-80	Epi-S3
IER3IP1	1.93E-84	0.36563	0.934	0.74	3.52E-80	Epi-S3
KIF21A	2.85E-84	0.348298	0.739	0.426	5.18E-80	Epi-S3
ACAT2	3.34E-84	0.349344	0.59	0.303	6.07E-80	Epi-S3
CAPNS1	3.77E-84	0.371625	0.873	0.627	6.87E-80	Epi-S3
EPS8L2	6.10E-84	0.343491	0.662	0.37	1.11E-79	Epi-S3
SCNN1A	6.39E-84	0.270044	0.465	0.2	1.16E-79	Epi-S3
ST14	8.83E-84	0.268718	0.443	0.18	1.61E-79	Epi-S3
CAMK2N1	9.44E-84	0.277714	0.487	0.21	1.72E-79	Epi-S3
CAPN1	9.50E-83	0.354478	0.904	0.685	1.73E-78	Epi-S3
AKR1A1	3.46E-82	0.358368	0.884	0.652	6.31E-78	Epi-S3

RAET1G	5.49E-82	0.363535	0.733	0.428	1.00E-77	Epi-S3
NSG1	1.47E-81	0.355458	0.74	0.438	2.67E-77	Epi-S3
GPR137B	4.83E-81	0.290784	0.446	0.192	8.80E-77	Epi-S3
LOC100130476	1.04E-80	0.269064	0.402	0.156	1.90E-76	Epi-S3
YWHAZ	1.07E-79	0.32102	0.967	0.835	1.95E-75	Epi-S3
SCD5	1.92E-79	0.376597	0.739	0.443	3.49E-75	Epi-S3
PIM1	3.11E-79	0.370259	0.8	0.528	5.67E-75	Epi-S3
POLE4	4.19E-79	0.359042	0.72	0.45	7.63E-75	Epi-S3
TALDO1	8.52E-79	0.346305	0.953	0.793	1.55E-74	Epi-S3
ARF1	1.46E-78	0.282601	0.986	0.919	2.66E-74	Epi-S3
DYNLRB1	5.15E-78	0.332518	0.958	0.809	9.38E-74	Epi-S3
NDUFB9	1.26E-77	0.309643	0.96	0.849	2.29E-73	Epi-S3
TINCR	1.64E-77	0.307232	0.511	0.244	2.99E-73	Epi-S3
AIM1	3.27E-77	0.326081	0.764	0.445	5.96E-73	Epi-S3
INPP1	7.67E-77	0.368009	0.604	0.341	1.40E-72	Epi-S3
EPCAM	1.35E-75	0.267742	0.394	0.157	2.46E-71	Epi-S3
CBLC	1.78E-74	0.269103	0.441	0.196	3.25E-70	Epi-S3
IL18	2.31E-74	0.353468	0.751	0.483	4.20E-70	Epi-S3
ZNF750	2.96E-74	0.29726	0.59	0.302	5.38E-70	Epi-S3
REEP6	1.06E-73	0.280564	0.514	0.245	1.92E-69	Epi-S3
OBFC1	4.20E-73	0.348823	0.627	0.368	7.64E-69	Epi-S3
EMP2	1.46E-72	0.324706	0.912	0.701	2.66E-68	Epi-S3
RASSF7	1.86E-72	0.308219	0.588	0.318	3.38E-68	Epi-S3
PID1	5.53E-72	0.317854	0.642	0.347	1.01E-67	Epi-S3
TIMM17A	3.22E-71	0.31924	0.839	0.632	5.85E-67	Epi-S3
PYGL	3.52E-71	0.323004	0.746	0.469	6.41E-67	Epi-S3
RIOK3	6.92E-71	0.359961	0.705	0.465	1.26E-66	Epi-S3
YPEL5	2.30E-70	0.320525	0.967	0.873	4.18E-66	Epi-S3
TECR	1.13E-69	0.290223	0.973	0.883	2.06E-65	Epi-S3
REEP4	1.30E-69	0.313334	0.656	0.386	2.37E-65	Epi-S3
NDUFB3	2.76E-69	0.297736	0.951	0.818	5.02E-65	Epi-S3
PKP3	3.83E-69	0.346256	0.773	0.514	6.97E-65	Epi-S3
GHITM	4.29E-69	0.305248	0.957	0.848	7.80E-65	Epi-S3
A4GALT	2.44E-68	0.294425	0.789	0.515	4.45E-64	Epi-S3
CMAS	4.48E-68	0.320579	0.69	0.45	8.15E-64	Epi-S3
FKBP4	1.17E-67	0.303084	0.682	0.407	2.14E-63	Epi-S3
SIRT7	4.20E-67	0.289054	0.594	0.328	7.65E-63	Epi-S3
NDUFC1	1.88E-66	0.267254	0.974	0.912	3.43E-62	Epi-S3
EML3	1.96E-66	0.3509	0.578	0.326	3.56E-62	Epi-S3
NECAP2	6.31E-66	0.294867	0.54	0.29	1.15E-61	Epi-S3
ESRP1	1.20E-65	0.273692	0.542	0.285	2.19E-61	Epi-S3
CCND1	2.60E-65	0.327222	0.895	0.652	4.74E-61	Epi-S3
SYT8	5.63E-65	0.307104	0.984	0.747	1.03E-60	Epi-S3
BIK	5.81E-65	0.304186	0.568	0.306	1.06E-60	Epi-S3
JUP	1.52E-64	0.310405	0.873	0.65	2.76E-60	Epi-S3
SLC50A1	1.20E-63	0.271611	0.532	0.283	2.18E-59	Epi-S3
PGAM1	1.21E-62	0.285946	0.968	0.879	2.20E-58	Epi-S3
AP1S1	1.30E-62	0.32127	0.759	0.526	2.37E-58	Epi-S3

TMPRSS4	2.07E-62	0.298509	0.683	0.415	3.76E-58	Epi-S3
PAX6	2.90E-62	0.32182	0.983	0.792	5.27E-58	Epi-S3
RNF149	1.74E-61	0.278917	0.68	0.415	3.16E-57	Epi-S3
NR4A1	8.52E-61	0.341396	0.693	0.44	1.55E-56	Epi-S3
COX17	2.07E-60	0.289758	0.808	0.58	3.77E-56	Epi-S3
PHLDA3	5.73E-60	0.287545	0.934	0.743	1.04E-55	Epi-S3
LAPTM4B	1.36E-59	0.307295	0.653	0.406	2.47E-55	Epi-S3
RBX1	5.68E-59	0.261411	0.969	0.881	1.03E-54	Epi-S3
TRIP10	6.67E-59	0.28406	0.589	0.338	1.21E-54	Epi-S3
CAP1	2.41E-58	0.299026	0.868	0.707	4.40E-54	Epi-S3
HMGCS1	3.08E-58	0.280932	0.561	0.308	5.62E-54	Epi-S3
POLR2I	7.30E-58	0.287302	0.902	0.741	1.33E-53	Epi-S3
RTCB	1.18E-57	0.290641	0.743	0.518	2.15E-53	Epi-S3
WIBG	2.93E-57	0.289795	0.67	0.43	5.34E-53	Epi-S3
PEA15	4.92E-57	0.282113	0.676	0.421	8.96E-53	Epi-S3
HEBP2	2.87E-56	0.29285	0.831	0.601	5.22E-52	Epi-S3
OSTF1	1.05E-55	0.285007	0.83	0.625	1.91E-51	Epi-S3
HTATIP2	1.88E-55	0.286273	0.689	0.45	3.43E-51	Epi-S3
SERBP1	3.07E-55	0.264118	0.961	0.86	5.59E-51	Epi-S3
GABARAPL2	4.09E-55	0.259188	0.966	0.872	7.44E-51	Epi-S3
TIMM8B	4.73E-55	0.28036	0.907	0.732	8.61E-51	Epi-S3
SSBP1	5.20E-55	0.261995	0.923	0.798	9.46E-51	Epi-S3
NDUFAB1	1.77E-54	0.276784	0.907	0.797	3.23E-50	Epi-S3
ELOVL1	1.92E-54	0.259328	0.578	0.341	3.49E-50	Epi-S3
PPP4C	2.49E-54	0.280498	0.872	0.677	4.53E-50	Epi-S3
PRPSAP1	4.75E-54	0.287092	0.626	0.399	8.65E-50	Epi-S3
TNNT3	1.25E-53	0.259453	0.717	0.441	2.28E-49	Epi-S3
SMAGP	3.19E-53	0.253152	0.618	0.371	5.81E-49	Epi-S3
NAGK	4.22E-53	0.270601	0.682	0.442	7.69E-49	Epi-S3
MMAB	2.64E-52	0.25835	0.525	0.302	4.81E-48	Epi-S3
LOC728554	4.94E-52	0.291065	0.841	0.631	9.00E-48	Epi-S3
DCXR	9.60E-52	0.280237	0.882	0.697	1.75E-47	Epi-S3
TBCB	1.13E-51	0.264476	0.859	0.671	2.06E-47	Epi-S3
IDI1	2.91E-51	0.292119	0.693	0.464	5.31E-47	Epi-S3
FDX1	1.33E-50	0.287462	0.769	0.583	2.43E-46	Epi-S3
COX14	1.45E-50	0.260085	0.903	0.751	2.64E-46	Epi-S3
FAM162A	1.86E-50	0.269198	0.891	0.741	3.39E-46	Epi-S3
TMEM141	1.97E-50	0.283818	0.697	0.476	3.59E-46	Epi-S3
DYNLT1	3.50E-50	0.263095	0.937	0.799	6.38E-46	Epi-S3
UNC5B-AS1	4.35E-50	0.297602	0.535	0.308	7.91E-46	Epi-S3
NHP2	5.78E-50	0.271073	0.906	0.766	1.05E-45	Epi-S3
PRSS8	8.27E-50	0.26226	0.606	0.376	1.51E-45	Epi-S3
BTF3L4	1.62E-49	0.269452	0.808	0.627	2.95E-45	Epi-S3
TUFT1	2.31E-49	0.265062	0.606	0.368	4.21E-45	Epi-S3
WLS	2.60E-49	0.267885	0.719	0.485	4.74E-45	Epi-S3
ALDH3A2	3.34E-49	0.27848	0.843	0.638	6.08E-45	Epi-S3
YWHAB	2.84E-48	0.280923	0.916	0.79	5.16E-44	Epi-S3
SDC4	3.43E-47	0.311079	0.827	0.601	6.24E-43	Epi-S3

MTCH1	6.40E-47	0.270397	0.889	0.758	1.16E-42	Epi-S3
KRT5	9.26E-46	0.280535	1	0.876	1.69E-41	Epi-S3
ARPC5L	3.34E-45	0.258107	0.778	0.575	6.08E-41	Epi-S3
BAD	1.86E-44	0.27235	0.77	0.588	3.38E-40	Epi-S3
MRPL20	2.33E-44	0.250457	0.897	0.745	4.24E-40	Epi-S3
THOC3	2.83E-44	0.258508	0.682	0.467	5.16E-40	Epi-S3
SERINC2	1.43E-43	0.252596	0.769	0.543	2.60E-39	Epi-S3
FDPS	3.09E-43	0.265639	0.698	0.504	5.63E-39	Epi-S3
GLO1	1.26E-41	0.258143	0.804	0.612	2.29E-37	Epi-S3
SLC20A1	2.75E-41	0.283622	0.642	0.418	5.00E-37	Epi-S3
MALL	5.03E-41	0.258495	0.929	0.711	9.16E-37	Epi-S3
RND3	1.14E-39	0.417101	0.775	0.611	2.07E-35	Epi-S3
HSPA8	1.44E-39	0.259988	0.911	0.786	2.63E-35	Epi-S3
KRT18	1.94E-38	0.259991	0.934	0.733	3.54E-34	Epi-S3
NDFIP2	1.02E-37	0.250511	0.614	0.414	1.86E-33	Epi-S3
ASPH	1.13E-37	0.263904	0.945	0.838	2.06E-33	Epi-S3
TUBB6	1.79E-37	0.281398	0.701	0.514	3.25E-33	Epi-S3
RHOC	1.44E-36	0.270251	0.842	0.72	2.61E-32	Epi-S3
PLS3	5.45E-36	0.253275	0.566	0.375	9.93E-32	Epi-S3
FAM83A	1.30E-34	0.260273	0.771	0.574	2.36E-30	Epi-S3
HBEGF	4.78E-34	0.318441	0.489	0.308	8.70E-30	Epi-S3
SIK1	7.07E-34	0.257908	0.646	0.453	1.29E-29	Epi-S3
AREG	9.53E-22	0.297394	0.603	0.436	1.74E-17	Epi-S3
MMP10	0	1.306833	0.425	0.034	0	LPC-2
S100A2	2.48E-303	1.737867	0.989	0.411	4.52E-299	LPC-2
KRT14	1.58E-232	1.438166	0.998	0.566	2.88E-228	LPC-2
KRT15	9.06E-221	1.747129	0.923	0.416	1.65E-216	LPC-2
GAS5	6.18E-195	0.896267	0.993	0.933	1.13E-190	LPC-2
C17orf76-AS1	1.03E-177	0.757055	0.998	0.963	1.87E-173	LPC-2
SLC6A6	6.13E-174	0.88018	0.543	0.143	1.12E-169	LPC-2
RPL13A	3.62E-172	0.5409	1	0.999	6.58E-168	LPC-2
FAM107A	1.89E-169	0.748462	0.387	0.071	3.43E-165	LPC-2
EEF1G	3.13E-169	0.619398	0.996	0.988	5.70E-165	LPC-2
RPS27	5.21E-166	0.549975	1	1	9.49E-162	LPC-2
EEF1A1	4.68E-162	0.678445	1	0.999	8.52E-158	LPC-2
RPS27A	1.21E-160	0.628176	1	0.997	2.20E-156	LPC-2
RPLP1	1.16E-159	0.495589	1	1	2.12E-155	LPC-2
COL18A1	6.94E-159	0.427833	0.365	0.065	1.26E-154	LPC-2
RPS3	1.99E-156	0.614485	1	0.996	3.62E-152	LPC-2
CXCL14	2.35E-153	1.033948	0.998	0.887	4.29E-149	LPC-2
CH25H	7.54E-153	0.485949	0.258	0.034	1.37E-148	LPC-2
RPL11	1.43E-152	0.440287	1	0.999	2.60E-148	LPC-2
RPL3	5.34E-150	0.525546	1	0.999	9.72E-146	LPC-2
RPL4	8.80E-149	0.585187	1	0.986	1.60E-144	LPC-2
RPL34	2.24E-145	0.572091	1	1	4.08E-141	LPC-2
GNB2L1	4.50E-145	0.515988	0.998	0.99	8.19E-141	LPC-2
RPS9	2.46E-143	0.54624	1	0.998	4.48E-139	LPC-2
RPS19	7.73E-143	0.581255	1	0.998	1.41E-138	LPC-2

RPL35A	2.35E-142	0.491863	1	0.998	4.28E-138	LPC-2
RPS7	1.52E-137	0.45802	1	0.993	2.77E-133	LPC-2
RPL30	3.41E-137	0.448751	1	0.998	6.22E-133	LPC-2
RPS3A	7.69E-137	0.517404	1	0.998	1.40E-132	LPC-2
RPL13	4.40E-136	0.575447	1	0.999	8.00E-132	LPC-2
RPS12	3.91E-135	0.500393	1	0.999	7.12E-131	LPC-2
RPL5	5.14E-135	0.673274	0.998	0.993	9.36E-131	LPC-2
ENSG0000021008[illegible]	1.23E-134	0.725946	0.996	1	2.24E-130	LPC-2
RPS8	3.12E-133	0.457496	1	0.999	5.67E-129	LPC-2
RPS23	1.91E-130	0.411592	1	0.998	3.48E-126	LPC-2
RPLP2	6.53E-129	0.443424	1	0.999	1.19E-124	LPC-2
RPL14	6.47E-128	0.503643	0.998	0.995	1.18E-123	LPC-2
RPS15A	3.21E-127	0.466118	0.998	0.997	5.84E-123	LPC-2
ENSG0000021145[illegible]	7.16E-125	0.802792	0.996	0.997	1.30E-120	LPC-2
RPS17L	5.54E-124	0.419177	1	0.996	1.01E-119	LPC-2
RPL15	2.63E-123	0.406916	0.998	0.999	4.79E-119	LPC-2
RPL10A	2.27E-122	0.493263	1	0.995	4.14E-118	LPC-2
RPL37	3.68E-122	0.458569	1	0.995	6.70E-118	LPC-2
RPL10	6.10E-122	0.489801	1	1	1.11E-117	LPC-2
ENSG0000019893[illegible]	1.51E-115	0.530276	0.998	0.998	2.75E-111	LPC-2
RPS5	2.18E-114	0.463164	0.996	0.989	3.97E-110	LPC-2
RPL7	8.06E-114	0.441969	1	0.998	1.47E-109	LPC-2
RPS16	1.85E-112	0.436908	1	0.994	3.36E-108	LPC-2
RPS20	2.08E-112	0.427149	1	0.997	3.79E-108	LPC-2
RPS6	2.46E-112	0.521533	1	0.999	4.47E-108	LPC-2
GLTSCR2	4.56E-112	0.755977	0.943	0.824	8.30E-108	LPC-2
RPL17	7.61E-112	0.5345	0.998	0.985	1.38E-107	LPC-2
RPL31	1.36E-111	0.441821	0.998	0.998	2.48E-107	LPC-2
RPL6	3.18E-111	0.466306	1	0.991	5.79E-107	LPC-2
BCAM	1.09E-110	0.695325	0.711	0.318	1.99E-106	LPC-2
RPL32	1.19E-110	0.497507	1	0.999	2.16E-106	LPC-2
RPL7A	7.56E-108	0.425764	0.998	0.987	1.38E-103	LPC-2
RPL27A	8.77E-107	0.385052	1	0.998	1.60E-102	LPC-2
ATP1B3	2.53E-106	0.628519	0.959	0.82	4.60E-102	LPC-2
NAP1L1	4.09E-106	0.693466	0.908	0.73	7.45E-102	LPC-2
RPL26	5.93E-103	0.366691	1	0.999	1.08E-98	LPC-2
CAV1	4.02E-102	0.548933	0.639	0.252	7.32E-98	LPC-2
RPS15	2.02E-101	0.327267	1	0.999	3.67E-97	LPC-2
RPL19	4.01E-100	0.336868	1	0.999	7.31E-96	LPC-2
CXCL1	7.68E-99	0.72067	0.429	0.123	1.40E-94	LPC-2
RPL39	1.11E-96	0.38815	1	0.999	2.03E-92	LPC-2
RPL18	5.49E-95	0.383369	1	0.996	9.99E-91	LPC-2
PTN	1.58E-94	0.466887	0.346	0.087	2.87E-90	LPC-2
RPL22	4.63E-94	0.391081	1	0.994	8.43E-90	LPC-2
RPL21	6.09E-93	0.41044	1	0.999	1.11E-88	LPC-2
RPL29	2.88E-92	0.406275	0.998	0.992	5.24E-88	LPC-2
RPL18A	1.82E-91	0.40997	1	0.997	3.32E-87	LPC-2
RPL8	1.58E-90	0.408495	1	0.998	2.89E-86	LPC-2

RPS25	2.85E-88	0.353339	1	0.998	5.19E-84	LPC-2
RPLP0	4.41E-88	0.39426	1	0.992	8.03E-84	LPC-2
RPL12	1.69E-87	0.487311	1	0.998	3.07E-83	LPC-2
RPS4X	4.82E-87	0.510575	1	0.999	8.77E-83	LPC-2
RPS14	5.30E-87	0.376504	1	1	9.64E-83	LPC-2
RPS11	2.12E-86	0.383407	0.993	0.985	3.85E-82	LPC-2
RPS18	6.77E-86	0.352372	1	1	1.23E-81	LPC-2
RPS24	2.05E-85	0.3394	1	0.998	3.73E-81	LPC-2
PHLDA1	3.26E-85	0.593179	0.517	0.196	5.94E-81	LPC-2
EEF1D	3.12E-84	0.414648	0.993	0.977	5.68E-80	LPC-2
FAM46A	5.65E-84	0.668678	0.722	0.419	1.03E-79	LPC-2
RPL41	2.18E-82	0.267481	1	1	3.97E-78	LPC-2
DST	3.17E-82	0.62209	0.676	0.354	5.76E-78	LPC-2
CITED2	1.72E-80	0.939593	0.86	0.642	3.13E-76	LPC-2
RPS13	3.77E-80	0.336455	1	0.998	6.86E-76	LPC-2
RPSA	1.33E-78	0.45749	0.95	0.868	2.42E-74	LPC-2
EIF3E	1.81E-78	0.63252	0.919	0.819	3.30E-74	LPC-2
LTB4R	9.37E-76	0.378654	0.331	0.099	1.71E-71	LPC-2
HNRNPA1	4.75E-74	0.408205	0.996	0.969	8.65E-70	LPC-2
PDLIM1	6.63E-74	0.479257	0.928	0.767	1.21E-69	LPC-2
RPL36	7.02E-72	0.352438	0.998	0.997	1.28E-67	LPC-2
RPS10	3.51E-71	0.449705	0.996	0.978	6.39E-67	LPC-2
GAPDH	2.97E-70	0.38206	1	0.998	5.40E-66	LPC-2
RPL24	3.98E-70	0.335833	1	0.994	7.25E-66	LPC-2
RPS28	6.73E-70	0.278755	0.998	0.999	1.22E-65	LPC-2
FOS	1.64E-67	0.64408	0.98	0.974	2.99E-63	LPC-2
ZFAS1	2.20E-67	0.464409	0.945	0.864	4.01E-63	LPC-2
RPL23A	1.14E-65	0.29587	1	0.998	2.08E-61	LPC-2
NPM1	3.42E-65	0.624456	0.971	0.927	6.23E-61	LPC-2
PTMA	9.24E-65	0.426102	1	0.996	1.68E-60	LPC-2
RPL23	2.32E-64	0.322094	1	0.984	4.23E-60	LPC-2
NACA	2.58E-64	0.287793	1	0.996	4.69E-60	LPC-2
HSP90AB1	1.05E-63	0.36064	0.987	0.963	1.91E-59	LPC-2
RPL35	2.30E-63	0.2772	1	0.999	4.20E-59	LPC-2
RPS21	2.93E-63	0.332027	1	0.989	5.33E-59	LPC-2
DEGS1	3.87E-63	0.59338	0.646	0.371	7.05E-59	LPC-2
RPL36A	4.88E-63	0.349468	0.994	0.989	8.88E-59	LPC-2
ENSG0000019876[illegible]	6.58E-63	0.410274	0.993	0.983	1.20E-58	LPC-2
RPS2	6.55E-62	0.296934	1	0.997	1.19E-57	LPC-2
TPT1	2.99E-61	0.295065	1	0.997	5.44E-57	LPC-2
WNT10A	1.18E-58	0.312216	0.348	0.118	2.14E-54	LPC-2
BTF3	1.57E-58	0.29729	1	0.993	2.87E-54	LPC-2
RPL9	2.00E-57	0.488701	0.904	0.783	3.64E-53	LPC-2
JUN	4.80E-57	0.730839	0.994	0.978	8.75E-53	LPC-2
LMNA	1.05E-56	0.488116	0.989	0.966	1.92E-52	LPC-2
RGS2	3.00E-56	0.437363	0.525	0.271	5.46E-52	LPC-2
ID2	4.99E-56	0.952556	0.762	0.553	9.08E-52	LPC-2
ENSG0000019888[illegible]	8.85E-56	0.41916	0.991	0.959	1.61E-51	LPC-2

MYC	9.01E-56	0.525317	0.781	0.559	1.64E-51	LPC-2
NFKBIA	2.71E-55	0.536608	0.963	0.942	4.94E-51	LPC-2
HSPA2	3.07E-55	0.504409	0.645	0.4	5.60E-51	LPC-2
SNAI2	3.53E-54	0.5021	0.61	0.353	6.43E-50	LPC-2
SNHG8	4.48E-54	0.511717	0.845	0.696	8.17E-50	LPC-2
SERTAD4	1.63E-53	0.34606	0.278	0.091	2.97E-49	LPC-2
FBL	2.24E-53	0.4825	0.718	0.546	4.09E-49	LPC-2
LAMB3	1.71E-52	0.446144	0.512	0.26	3.11E-48	LPC-2
IER3	1.98E-52	0.542764	0.915	0.829	3.61E-48	LPC-2
KRT17	1.03E-51	0.698818	0.669	0.414	1.88E-47	LPC-2
DUSP6	2.46E-51	0.424211	0.433	0.202	4.47E-47	LPC-2
MSS51	3.41E-51	0.321692	0.994	0.979	6.20E-47	LPC-2
PTRF	5.27E-51	0.336363	0.757	0.445	9.60E-47	LPC-2
ZFP36	1.16E-50	0.511473	0.991	0.975	2.11E-46	LPC-2
FHL2	1.50E-50	0.425475	0.608	0.36	2.73E-46	LPC-2
EIF3L	5.44E-50	0.446861	0.906	0.815	9.90E-46	LPC-2
PLK2	1.44E-49	0.518163	0.755	0.524	2.61E-45	LPC-2
EPB41L4A-AS1	2.04E-49	0.46925	0.705	0.519	3.72E-45	LPC-2
SNORA33	2.73E-48	0.401189	0.866	0.764	4.97E-44	LPC-2
RPL38	7.49E-48	0.286845	0.996	0.989	1.36E-43	LPC-2
COL17A1	3.01E-47	0.430358	0.744	0.479	5.48E-43	LPC-2
ZFP36L2	9.35E-47	0.5063	0.72	0.516	1.70E-42	LPC-2
GPHA2	1.68E-45	0.274404	0.274	0.097	3.05E-41	LPC-2
PPP1R15A	1.98E-45	0.412045	0.967	0.915	3.61E-41	LPC-2
SPATS2	3.82E-45	0.345938	0.346	0.148	6.96E-41	LPC-2
UBA52	1.47E-44	0.250111	0.998	0.987	2.68E-40	LPC-2
PFDN5	1.73E-44	0.334908	0.982	0.974	3.14E-40	LPC-2
PABPC1	4.20E-43	0.473866	0.947	0.901	7.65E-39	LPC-2
EDN1	9.37E-43	0.407176	0.341	0.144	1.71E-38	LPC-2
DUSP1	6.37E-42	0.413359	0.985	0.988	1.16E-37	LPC-2
LGALS3BP	1.92E-41	0.435493	0.681	0.499	3.50E-37	LPC-2
RAB4A	5.51E-41	0.357876	0.843	0.734	1.00E-36	LPC-2
SNHG6	6.01E-41	0.371881	0.862	0.757	1.09E-36	LPC-2
AVPI1	6.64E-41	0.440331	0.694	0.525	1.21E-36	LPC-2
ATF3	7.43E-40	0.455153	0.86	0.695	1.35E-35	LPC-2
SGK1	7.82E-39	0.575529	0.808	0.707	1.42E-34	LPC-2
PMAIP1	1.06E-38	0.480188	0.869	0.7	1.92E-34	LPC-2
MALAT1	1.37E-38	0.329264	0.998	0.998	2.50E-34	LPC-2
EPAS1	2.07E-38	0.388639	0.506	0.3	3.77E-34	LPC-2
KRT8	3.50E-38	0.408049	0.622	0.39	6.37E-34	LPC-2
TRA2B	1.07E-37	0.378893	0.86	0.792	1.95E-33	LPC-2
GLCE	3.39E-37	0.371253	0.42	0.224	6.17E-33	LPC-2
CAV2	1.35E-36	0.405936	0.471	0.277	2.46E-32	LPC-2
CDH13	1.38E-36	0.287455	0.319	0.138	2.52E-32	LPC-2
ARL4A	2.97E-36	0.438483	0.779	0.611	5.41E-32	LPC-2
TXNIP	1.55E-35	0.772578	0.757	0.674	2.81E-31	LPC-2
SNHG5	4.72E-35	0.309851	0.95	0.917	8.59E-31	LPC-2
RPS14P3	8.05E-35	0.291655	0.98	0.961	1.47E-30	LPC-2

C6orf48	9.78E-35	0.398152	0.773	0.647	1.78E-30	LPC-2
SOCS3	2.80E-34	0.444335	0.534	0.326	5.10E-30	LPC-2
EEF1B2	4.48E-34	0.303123	0.943	0.902	8.16E-30	LPC-2
RSL24D1	1.34E-33	0.353083	0.775	0.665	2.44E-29	LPC-2
COMMD6	1.37E-33	0.298248	0.95	0.925	2.50E-29	LPC-2
NTRK2	1.68E-33	0.410619	0.512	0.295	3.06E-29	LPC-2
SLC3A2	2.18E-33	0.377202	0.827	0.787	3.96E-29	LPC-2
EFEMP1	3.43E-33	0.352499	0.508	0.308	6.25E-29	LPC-2
JUNB	3.68E-33	0.376882	0.994	0.996	6.70E-29	LPC-2
EEF2	4.90E-33	0.413012	0.908	0.873	8.92E-29	LPC-2
HNRNPA0	7.68E-33	0.284903	0.904	0.874	1.40E-28	LPC-2
PLTP	2.12E-32	0.317039	0.28	0.123	3.85E-28	LPC-2
FOSB	3.35E-32	0.460686	0.906	0.829	6.11E-28	LPC-2
NCOA7	6.39E-32	0.467991	0.37	0.198	1.16E-27	LPC-2
HMGN3	6.64E-32	0.299463	0.971	0.929	1.21E-27	LPC-2
ZNF503	1.19E-31	0.311556	0.379	0.199	2.16E-27	LPC-2
L32131	8.81E-31	0.339167	0.829	0.742	1.60E-26	LPC-2
IVNS1ABP	9.25E-31	0.364388	0.626	0.454	1.68E-26	LPC-2
C12orf57	6.71E-30	0.349141	0.816	0.74	1.22E-25	LPC-2
PNRC1	1.06E-29	0.29811	0.908	0.866	1.92E-25	LPC-2
KLF4	1.17E-29	0.445131	0.952	0.916	2.14E-25	LPC-2
CXCL2	3.23E-29	0.317553	0.427	0.228	5.88E-25	LPC-2
BOC	3.91E-28	0.303676	0.346	0.183	7.12E-24	LPC-2
IMPA2	6.76E-28	0.286615	0.46	0.281	1.23E-23	LPC-2
NFIB	1.00E-27	0.34963	0.313	0.162	1.83E-23	LPC-2
HOPX	1.68E-27	0.375886	0.737	0.556	3.07E-23	LPC-2
HNRPDL	3.71E-27	0.270443	0.928	0.912	6.75E-23	LPC-2
ESD	1.28E-26	0.315226	0.659	0.53	2.32E-22	LPC-2
SYNE2	1.25E-25	0.372359	0.538	0.384	2.27E-21	LPC-2
APP	3.36E-25	0.313821	0.716	0.562	6.12E-21	LPC-2
IGFBP7	4.83E-25	0.286623	0.869	0.651	8.79E-21	LPC-2
OAT	2.50E-24	0.306901	0.534	0.377	4.54E-20	LPC-2
EIF4A2	2.58E-23	0.358102	0.923	0.902	4.69E-19	LPC-2
ZC3H12A	1.02E-22	0.31557	0.418	0.264	1.85E-18	LPC-2
FCGRT	3.03E-22	0.252183	0.462	0.306	5.51E-18	LPC-2
NFIC	4.31E-22	0.297223	0.534	0.398	7.85E-18	LPC-2
CCNI	1.00E-21	0.305232	0.672	0.537	1.82E-17	LPC-2
PTMS	2.83E-21	0.29307	0.654	0.538	5.16E-17	LPC-2
SNHG7	6.16E-21	0.301263	0.687	0.596	1.12E-16	LPC-2
ATF4	6.17E-21	0.269088	0.786	0.731	1.12E-16	LPC-2
POLR1D	9.82E-21	0.253022	0.902	0.827	1.79E-16	LPC-2
NCL	2.61E-19	0.253263	0.84	0.808	4.76E-15	LPC-2
RSL1D1	1.01E-18	0.315351	0.727	0.687	1.85E-14	LPC-2
MARCKSL1	7.39E-18	0.250232	0.392	0.245	1.34E-13	LPC-2
LUZP1	2.25E-17	0.258175	0.394	0.267	4.10E-13	LPC-2
CFI	3.38E-17	0.252018	0.378	0.251	6.16E-13	LPC-2
FAM134B	1.60E-16	0.251281	0.429	0.305	2.91E-12	LPC-2
MYLIP	4.39E-16	0.25401	0.473	0.348	8.00E-12	LPC-2

IGFBP3	3.65E-15	0.523832	0.337	0.204	6.65E-11	LPC-2
H1FX	2.04E-14	0.314405	0.51	0.42	3.72E-10	LPC-2
IMPDH2	1.22E-13	0.254424	0.549	0.464	2.22E-09	LPC-2
AES	2.06E-13	0.252281	0.692	0.643	3.76E-09	LPC-2
IFRD1	2.15E-12	0.281517	0.492	0.409	3.92E-08	LPC-2
TGIF1	1.32E-11	0.276038	0.696	0.676	2.41E-07	LPC-2
IRF1	2.83E-11	0.271425	0.471	0.375	5.15E-07	LPC-2
TUBA1A	8.83E-11	0.283444	0.731	0.709	1.61E-06	LPC-2
KLF10	3.15E-10	0.321699	0.687	0.672	5.74E-06	LPC-2
HIST1H1C	1.18E-09	0.273818	0.308	0.223	2.14E-05	LPC-2
ID3	3.45E-09	0.354329	0.803	0.759	6.28E-05	LPC-2
C1orf63	5.62E-09	0.260862	0.565	0.512	0.000102	LPC-2
SOX4	1.81E-06	0.260852	0.431	0.381	0.032934	LPC-2
TNFRSF12A	1.91E-06	0.331737	0.435	0.384	0.034817	LPC-2
PLAU	5.39E-06	0.366659	0.516	0.497	0.09813	LPC-2
PLAUR	0.00017	0.421957	0.339	0.289	1	LPC-2
UBE2C	0	1.740535	0.755	0.008	0	Epi-B1
BIRC5	0	1.317444	0.826	0.007	0	Epi-B1
TK1	0	1.237067	0.809	0.015	0	Epi-B1
CCNB1	0	1.230135	0.574	0.024	0	Epi-B1
NUSAP1	0	1.213074	0.809	0.008	0	Epi-B1
CDK1	0	1.130723	0.723	0.005	0	Epi-B1
SMC4	0	1.065916	0.869	0.13	0	Epi-B1
CDC20	0	1.056098	0.613	0.011	0	Epi-B1
CCNB2	0	1.053725	0.713	0.008	0	Epi-B1
PBK	0	1.000525	0.766	0.001	0	Epi-B1
MAD2L1	0	0.985833	0.833	0.035	0	Epi-B1
CENPF	0	0.98198	0.663	0.005	0	Epi-B1
CENPW	0	0.971194	0.872	0.07	0	Epi-B1
TOP2A	0	0.904319	0.621	0.006	0	Epi-B1
ZWINT	0	0.902055	0.833	0.036	0	Epi-B1
CDKN3	0	0.892885	0.677	0.009	0	Epi-B1
UBE2T	0	0.839756	0.798	0.099	0	Epi-B1
RRM2	0	0.787001	0.454	0.003	0	Epi-B1
PRC1	0	0.783337	0.691	0.024	0	Epi-B1
MKI67	0	0.702095	0.624	0.002	0	Epi-B1
HMMR	0	0.686566	0.585	0.005	0	Epi-B1
SGOL1	0	0.6764	0.67	0.006	0	Epi-B1
CENPA	0	0.668042	0.479	0.006	0	Epi-B1
TYMS	0	0.659033	0.504	0.011	0	Epi-B1
TPX2	0	0.627217	0.574	0.005	0	Epi-B1
NUF2	0	0.598865	0.624	0.004	0	Epi-B1
CKAP2L	0	0.541472	0.585	0.005	0	Epi-B1
AURKA	0	0.540103	0.415	0.016	0	Epi-B1
CENPK	0	0.536608	0.603	0.01	0	Epi-B1
ASF1B	0	0.52536	0.571	0.009	0	Epi-B1
KIF23	0	0.518191	0.504	0.006	0	Epi-B1
PLK1	0	0.51816	0.408	0.004	0	Epi-B1

RAD51AP1	0	0.517953	0.539	0.014	0	Epi-B1
KIFC1	0	0.516006	0.528	0.006	0	Epi-B1
AURKB	0	0.515446	0.5	0.002	0	Epi-B1
CCNA2	0	0.49794	0.5	0.001	0	Epi-B1
ASPM	0	0.478081	0.429	0.001	0	Epi-B1
CDCA8	0	0.475258	0.482	0.015	0	Epi-B1
CENPM	0	0.4589	0.468	0.012	0	Epi-B1
OIP5	0	0.445681	0.525	0.005	0	Epi-B1
NDC80	0	0.441372	0.507	0.003	0	Epi-B1
TACC3	0	0.440594	0.507	0.006	0	Epi-B1
ANLN	0	0.424124	0.436	0.004	0	Epi-B1
SGOL2	0	0.411404	0.436	0.019	0	Epi-B1
SPC25	0	0.400438	0.436	0.001	0	Epi-B1
CDCA3	0	0.399613	0.447	0.008	0	Epi-B1
GINS2	0	0.39832	0.387	0.017	0	Epi-B1
TROAP	0	0.390622	0.408	0.003	0	Epi-B1
POC1A	0	0.381238	0.447	0.014	0	Epi-B1
CENPE	0	0.381176	0.358	0.008	0	Epi-B1
MND1	0	0.374321	0.475	0.003	0	Epi-B1
NEK2	0	0.371799	0.365	0.002	0	Epi-B1
DEPDC1	0	0.342754	0.379	0.001	0	Epi-B1
HJURP	0	0.336151	0.383	0.001	0	Epi-B1
RAD51	0	0.315665	0.369	0.009	0	Epi-B1
DLGAP5	0	0.308411	0.348	0.001	0	Epi-B1
KIF2C	0	0.300708	0.309	0.001	0	Epi-B1
FAM64A	0	0.294811	0.362	0.002	0	Epi-B1
CEP55	0	0.292836	0.362	0.002	0	Epi-B1
CLSPN	0	0.290436	0.312	0.005	0	Epi-B1
TTK	0	0.290209	0.376	0.003	0	Epi-B1
CDCA5	0	0.281789	0.369	0.004	0	Epi-B1
MELK	0	0.281104	0.358	0.002	0	Epi-B1
GTSE1	0	0.273224	0.344	0.002	0	Epi-B1
ARHGAP11A	0	0.269046	0.319	0.005	0	Epi-B1
PRR11	0	0.266118	0.305	0.01	0	Epi-B1
KIF4A	0	0.264372	0.33	0.003	0	Epi-B1
DIAPH3	0	0.254144	0.301	0.005	0	Epi-B1
CENPN	1.45E-306	0.387756	0.507	0.037	2.64E-302	Epi-B1
STMN1	1.87E-304	1.938922	0.961	0.194	3.41E-300	Epi-B1
KIAA0101	8.56E-302	1.033035	0.649	0.069	1.56E-297	Epi-B1
ORC6	1.03E-296	0.276017	0.376	0.02	1.88E-292	Epi-B1
CCDC34	1.45E-284	0.586406	0.592	0.057	2.64E-280	Epi-B1
KIF20B	1.28E-278	0.707859	0.681	0.079	2.33E-274	Epi-B1
SMC2	2.23E-261	0.678271	0.78	0.113	4.07E-257	Epi-B1
FAM83D	1.04E-245	0.40816	0.401	0.029	1.90E-241	Epi-B1
PHF19	9.40E-223	0.27617	0.372	0.027	1.71E-218	Epi-B1
FBXO5	4.19E-220	0.310608	0.411	0.034	7.63E-216	Epi-B1
CDC25B	1.05E-219	0.35785	0.454	0.042	1.92E-215	Epi-B1
RACGAP1	6.30E-219	0.330321	0.376	0.028	1.15E-214	Epi-B1

RMI2	5.30E-211	0.253335	0.344	0.025	9.66E-207	Epi-B1
ECT2	6.49E-208	0.276182	0.323	0.022	1.18E-203	Epi-B1
MLF1IP	1.37E-201	0.766787	0.762	0.142	2.49E-197	Epi-B1
CENPH	5.23E-199	0.611652	0.642	0.098	9.52E-195	Epi-B1
PTTG1	1.02E-185	1.556806	0.965	0.378	1.86E-181	Epi-B1
CKS2	5.91E-181	1.596012	0.933	0.327	1.08E-176	Epi-B1
HMGB2	1.59E-172	1.720944	0.965	0.449	2.89E-168	Epi-B1
ATAD2	3.15E-169	0.268904	0.355	0.033	5.74E-165	Epi-B1
VRK1	4.49E-167	0.52056	0.674	0.122	8.18E-163	Epi-B1
HELLS	3.43E-163	0.252676	0.316	0.027	6.24E-159	Epi-B1
HYLS1	7.90E-157	0.293265	0.401	0.045	1.44E-152	Epi-B1
H2AFZ	3.72E-154	1.425429	0.996	0.895	6.78E-150	Epi-B1
CKAP2	8.94E-154	0.442771	0.5	0.073	1.63E-149	Epi-B1
LMNB1	1.25E-148	0.278432	0.397	0.046	2.28E-144	Epi-B1
HMGB1	6.78E-145	0.973745	1	0.982	1.23E-140	Epi-B1
FEN1	2.55E-139	0.511187	0.599	0.113	4.64E-135	Epi-B1
TMPO	2.59E-138	0.394544	0.56	0.096	4.72E-134	Epi-B1
GGH	8.66E-136	0.780621	0.883	0.283	1.58E-131	Epi-B1
CKS1B	6.01E-133	1.028157	0.947	0.519	1.09E-128	Epi-B1
DHFR	1.89E-122	0.275131	0.344	0.042	3.44E-118	Epi-B1
CDCA4	7.79E-122	0.685366	0.738	0.209	1.42E-117	Epi-B1
RNASEH2A	5.32E-117	0.431374	0.571	0.117	9.69E-113	Epi-B1
RRM1	2.36E-115	0.599963	0.713	0.19	4.30E-111	Epi-B1
GMNN	8.44E-114	0.452604	0.56	0.117	1.54E-109	Epi-B1
KNSTRN	3.47E-112	0.316124	0.351	0.048	6.31E-108	Epi-B1
NMU	1.29E-110	0.768136	0.798	0.261	2.35E-106	Epi-B1
BCL2L12	5.57E-110	0.314594	0.514	0.096	1.01E-105	Epi-B1
RFC4	6.56E-109	0.279864	0.443	0.073	1.19E-104	Epi-B1
TUBB	1.51E-105	1.358502	0.982	0.826	2.75E-101	Epi-B1
HMGB3	2.24E-101	0.39694	0.525	0.112	4.09E-97	Epi-B1
NUDT1	1.28E-100	0.448499	0.716	0.197	2.33E-96	Epi-B1
HMGN2	4.04E-98	0.911328	0.996	0.847	7.35E-94	Epi-B1
CHAF1A	5.24E-98	0.262086	0.358	0.054	9.53E-94	Epi-B1
RPL39L	1.19E-97	0.384504	0.571	0.131	2.16E-93	Epi-B1
KRT14	6.06E-97	1.329275	0.968	0.573	1.10E-92	Epi-B1
DNAJC9	3.09E-96	0.587579	0.784	0.274	5.63E-92	Epi-B1
CAV1	1.23E-95	0.67036	0.794	0.255	2.23E-91	Epi-B1
MCM7	1.38E-95	0.450859	0.518	0.116	2.51E-91	Epi-B1
DEK	3.10E-95	0.721789	0.929	0.478	5.65E-91	Epi-B1
S100A2	9.62E-95	1.149985	0.922	0.422	1.75E-90	Epi-B1
H2AFV	9.90E-95	0.720772	0.947	0.533	1.80E-90	Epi-B1
SKA2	1.49E-93	0.491927	0.741	0.234	2.72E-89	Epi-B1
ITGB3BP	1.54E-93	0.432928	0.635	0.166	2.81E-89	Epi-B1
UBE2S	5.12E-93	0.610748	0.684	0.211	9.31E-89	Epi-B1
BASP1	7.66E-91	0.376004	0.408	0.076	1.40E-86	Epi-B1
GPSM2	3.99E-90	0.374031	0.443	0.088	7.27E-86	Epi-B1
GAPDH	1.99E-88	0.611511	1	0.998	3.62E-84	Epi-B1
MIS18A	3.08E-88	0.306689	0.521	0.115	5.61E-84	Epi-B1

KPNA2	2.98E-86	1.066989	0.745	0.28	5.43E-82	Epi-B1
HNRNPA1	1.24E-85	0.640092	0.996	0.969	2.26E-81	Epi-B1
HNRNPA2B1	1.98E-85	0.613922	0.993	0.957	3.60E-81	Epi-B1
PHGDH	2.30E-85	0.664052	0.869	0.379	4.19E-81	Epi-B1
MOXD1	6.61E-85	0.505217	0.628	0.171	1.20E-80	Epi-B1
C19orf48	8.06E-83	0.427542	0.642	0.186	1.47E-78	Epi-B1
TNNT1	9.86E-82	0.318786	0.404	0.079	1.79E-77	Epi-B1
TUBA1A	3.93E-81	0.904596	0.954	0.706	7.16E-77	Epi-B1
CPVL	2.90E-80	0.605044	0.638	0.186	5.28E-76	Epi-B1
MIS18BP1	3.26E-79	0.295824	0.454	0.1	5.93E-75	Epi-B1
CENPQ	7.17E-79	0.260983	0.443	0.094	1.31E-74	Epi-B1
BCAM	2.16E-78	0.656958	0.83	0.323	3.93E-74	Epi-B1
IMPA2	8.69E-73	0.452416	0.77	0.278	1.58E-68	Epi-B1
CRLF1	1.81E-72	0.341536	0.493	0.122	3.30E-68	Epi-B1
LSM5	6.46E-72	0.582917	0.972	0.754	1.18E-67	Epi-B1
MZT2A	1.26E-71	0.53826	0.844	0.391	2.29E-67	Epi-B1
APITD1	4.87E-69	0.286571	0.465	0.114	8.87E-65	Epi-B1
HIST1H4C	3.95E-68	0.954253	0.688	0.284	7.19E-64	Epi-B1
CKLF	4.24E-66	0.520519	0.759	0.308	7.72E-62	Epi-B1
RAN	2.37E-65	0.502593	0.996	0.871	4.32E-61	Epi-B1
HNRNPA3	7.32E-65	0.542119	0.972	0.803	1.33E-60	Epi-B1
BUB3	7.40E-65	0.642567	0.883	0.511	1.35E-60	Epi-B1
RAD21	2.88E-64	0.501551	0.812	0.36	5.25E-60	Epi-B1
PLP2	5.28E-64	0.49973	1	0.897	9.62E-60	Epi-B1
ANP32B	1.15E-63	0.601701	0.961	0.734	2.09E-59	Epi-B1
ARL6IP1	3.26E-63	1.092666	0.954	0.776	5.93E-59	Epi-B1
PTN	2.41E-62	0.385013	0.39	0.09	4.39E-58	Epi-B1
RAD51C	1.83E-61	0.35707	0.628	0.209	3.33E-57	Epi-B1
PTMS	2.89E-61	0.712543	0.879	0.536	5.25E-57	Epi-B1
CDKN2C	3.71E-60	0.328097	0.528	0.157	6.75E-56	Epi-B1
MT2A	8.33E-60	0.612499	0.996	0.899	1.52E-55	Epi-B1
MZT1	1.61E-59	0.359467	0.677	0.243	2.92E-55	Epi-B1
NUCKS1	3.29E-59	0.5854	0.965	0.771	5.98E-55	Epi-B1
CXCL14	3.81E-59	0.803943	0.996	0.889	6.94E-55	Epi-B1
PTMA	1.73E-58	0.681146	1	0.996	3.15E-54	Epi-B1
MZT2B	1.80E-58	0.543577	0.95	0.697	3.28E-54	Epi-B1
HN1	1.01E-57	0.630115	0.908	0.574	1.85E-53	Epi-B1
ANP32E	2.18E-56	0.489598	0.706	0.306	3.98E-52	Epi-B1
CCDC109B	3.98E-55	0.405773	0.727	0.311	7.25E-51	Epi-B1
TGM2	7.85E-54	0.364266	0.582	0.195	1.43E-49	Epi-B1
NASP	1.79E-53	0.50622	0.766	0.367	3.27E-49	Epi-B1
RPA3	1.15E-52	0.628165	0.897	0.576	2.10E-48	Epi-B1
MTHFD2	3.75E-52	0.328048	0.56	0.184	6.83E-48	Epi-B1
COL17A1	2.10E-51	0.618467	0.869	0.482	3.82E-47	Epi-B1
H2AFX	3.35E-51	0.339478	0.582	0.204	6.10E-47	Epi-B1
YEATS4	1.07E-50	0.29168	0.564	0.19	1.94E-46	Epi-B1
VIT	2.03E-50	0.316334	0.514	0.164	3.69E-46	Epi-B1
LSM4	3.12E-50	0.473015	0.954	0.708	5.68E-46	Epi-B1

KIF22	1.18E-48	0.452961	0.801	0.402	2.15E-44	Epi-B1
RHNO1	1.35E-48	0.268493	0.532	0.175	2.46E-44	Epi-B1
MT1X	3.00E-48	0.620659	1	0.976	5.47E-44	Epi-B1
TUBG1	3.51E-48	0.326914	0.61	0.237	6.39E-44	Epi-B1
YWHAH	6.92E-48	0.481975	0.926	0.599	1.26E-43	Epi-B1
DDX39A	1.58E-47	0.365977	0.656	0.275	2.87E-43	Epi-B1
PCNA	4.51E-47	0.824229	0.826	0.523	8.22E-43	Epi-B1
KCNN4	9.91E-47	0.431047	0.748	0.349	1.81E-42	Epi-B1
S100A10	1.64E-46	0.469693	1	0.95	2.99E-42	Epi-B1
RDX	6.15E-46	0.457943	0.922	0.601	1.12E-41	Epi-B1
SNRPB	1.38E-45	0.42788	0.954	0.746	2.51E-41	Epi-B1
ATP1B3	2.55E-45	0.463345	0.972	0.822	4.64E-41	Epi-B1
RFC2	8.89E-45	0.315099	0.525	0.185	1.62E-40	Epi-B1
MRPL51	1.63E-44	0.398846	0.993	0.792	2.96E-40	Epi-B1
CALM3	3.01E-44	0.462693	0.844	0.496	5.47E-40	Epi-B1
TAGLN2	7.65E-44	0.401824	1	0.955	1.39E-39	Epi-B1
RANBP1	1.26E-43	0.499186	0.933	0.65	2.30E-39	Epi-B1
TMEM237	1.60E-42	0.260736	0.574	0.211	2.92E-38	Epi-B1
SAE1	3.39E-42	0.320158	0.706	0.298	6.16E-38	Epi-B1
CAV2	7.35E-41	0.31007	0.667	0.276	1.34E-36	Epi-B1
ILF2	1.07E-40	0.428458	0.911	0.683	1.95E-36	Epi-B1
PKM	1.19E-40	0.364805	1	0.98	2.17E-36	Epi-B1
LSM3	2.19E-40	0.375092	0.975	0.846	3.99E-36	Epi-B1
RUVBL2	2.69E-40	0.324792	0.684	0.304	4.90E-36	Epi-B1
IMMP1L	2.97E-40	0.348227	0.738	0.357	5.41E-36	Epi-B1
ZDHHC12	5.93E-40	0.356172	0.656	0.297	1.08E-35	Epi-B1
IVNS1ABP	9.06E-40	0.50841	0.794	0.454	1.65E-35	Epi-B1
PSIP1	1.41E-39	0.318669	0.621	0.255	2.57E-35	Epi-B1
SAC3D1	2.11E-39	0.261319	0.422	0.14	3.85E-35	Epi-B1
SNRPD1	2.57E-37	0.387196	0.933	0.707	4.68E-33	Epi-B1
TPGS2	2.77E-37	0.338068	0.738	0.361	5.04E-33	Epi-B1
NUP93	3.52E-37	0.37564	0.684	0.327	6.41E-33	Epi-B1
PXMP2	1.21E-36	0.266672	0.543	0.215	2.20E-32	Epi-B1
NUP37	5.24E-36	0.261354	0.55	0.221	9.53E-32	Epi-B1
LDHB	7.83E-36	0.44911	0.989	0.836	1.43E-31	Epi-B1
SMC3	1.14E-35	0.335177	0.784	0.419	2.08E-31	Epi-B1
ACAT2	1.50E-35	0.326016	0.66	0.312	2.74E-31	Epi-B1
SRSF3	2.42E-35	0.367944	0.989	0.914	4.41E-31	Epi-B1
TMEM106C	3.02E-35	0.332623	0.592	0.262	5.50E-31	Epi-B1
C17orf76-AS1	4.47E-35	0.359631	0.993	0.964	8.14E-31	Epi-B1
FBLN1	6.24E-35	0.285781	0.645	0.289	1.14E-30	Epi-B1
PPP1CA	1.48E-34	0.368856	0.965	0.782	2.69E-30	Epi-B1
MCM3	2.39E-34	0.250119	0.362	0.119	4.35E-30	Epi-B1
H2AFY	3.84E-34	0.406938	0.922	0.701	6.98E-30	Epi-B1
KCNJ3	4.73E-34	0.360321	0.908	0.622	8.61E-30	Epi-B1
RPS2	1.14E-33	0.286973	1	0.997	2.07E-29	Epi-B1
BTG3	1.99E-33	0.400427	0.876	0.552	3.62E-29	Epi-B1
IGFBP7	2.03E-33	0.466523	0.95	0.653	3.70E-29	Epi-B1

NTRK2	3.20E-33	0.377934	0.642	0.296	5.82E-29	Epi-B1
GGCT	6.70E-33	0.471658	0.872	0.573	1.22E-28	Epi-B1
PFN1	1.21E-32	0.311633	1	0.985	2.19E-28	Epi-B1
HP1BP3	1.71E-32	0.34797	0.844	0.493	3.11E-28	Epi-B1
RBBP7	4.00E-32	0.320164	0.837	0.499	7.29E-28	Epi-B1
RPSA	5.04E-32	0.369924	0.979	0.869	9.18E-28	Epi-B1
ALDH7A1	5.33E-32	0.389612	0.897	0.602	9.70E-28	Epi-B1
CLCA2	6.92E-32	0.350858	0.798	0.441	1.26E-27	Epi-B1
DUT	8.56E-32	0.521982	0.929	0.748	1.56E-27	Epi-B1
TUBB4B	9.48E-32	0.540731	0.986	0.921	1.73E-27	Epi-B1
CBX3	1.12E-31	0.345482	0.812	0.471	2.03E-27	Epi-B1
PAFAH1B3	1.77E-31	0.274069	0.628	0.281	3.22E-27	Epi-B1
HSP90B1	2.01E-31	0.490933	0.979	0.822	3.66E-27	Epi-B1
ACTG1	2.61E-31	0.31661	1	0.995	4.76E-27	Epi-B1
SLBP	3.05E-31	0.432788	0.784	0.492	5.56E-27	Epi-B1
GSN	3.61E-31	0.419671	0.993	0.905	6.57E-27	Epi-B1
EIF3F	3.63E-31	0.336095	0.961	0.77	6.61E-27	Epi-B1
LOC100505633	5.15E-31	0.262432	0.699	0.326	9.38E-27	Epi-B1
NAP1L1	5.97E-31	0.348069	0.943	0.732	1.09E-26	Epi-B1
LYAR	8.17E-31	0.284211	0.574	0.261	1.49E-26	Epi-B1
SPARC	9.88E-31	0.451355	0.809	0.447	1.80E-26	Epi-B1
MLF2	1.68E-30	0.358928	0.947	0.759	3.07E-26	Epi-B1
TMEM14A	1.48E-29	0.335973	0.823	0.505	2.69E-25	Epi-B1
CLIC1	1.94E-29	0.298323	0.993	0.912	3.53E-25	Epi-B1
GJB2	2.01E-29	0.445883	0.947	0.673	3.67E-25	Epi-B1
HNRNPR	3.61E-29	0.332057	0.887	0.639	6.57E-25	Epi-B1
BOLA3	3.93E-29	0.260468	0.681	0.332	7.15E-25	Epi-B1
HOMER3	1.20E-28	0.39014	0.745	0.423	2.19E-24	Epi-B1
RPS3	1.52E-28	0.252426	1	0.996	2.77E-24	Epi-B1
HNRNPD	3.03E-28	0.315615	0.823	0.518	5.51E-24	Epi-B1
ACTL6A	3.46E-28	0.269785	0.709	0.358	6.31E-24	Epi-B1
HNRPDL	6.82E-28	0.332158	0.982	0.911	1.24E-23	Epi-B1
RPLP0	1.76E-27	0.299553	1	0.992	3.21E-23	Epi-B1
TPRKB	2.78E-27	0.276844	0.787	0.451	5.06E-23	Epi-B1
HAT1	4.08E-27	0.262278	0.652	0.324	7.44E-23	Epi-B1
RASSF6	4.26E-27	0.323098	0.652	0.331	7.75E-23	Epi-B1
TPM4	4.70E-27	0.293803	0.823	0.487	8.56E-23	Epi-B1
CKAP4	5.34E-27	0.291864	0.773	0.435	9.72E-23	Epi-B1
NAP1L4	5.82E-27	0.289034	0.773	0.454	1.06E-22	Epi-B1
GJB6	6.44E-27	0.497472	0.922	0.62	1.17E-22	Epi-B1
POLE3	1.38E-26	0.303642	0.77	0.423	2.51E-22	Epi-B1
GSTO1	1.45E-26	0.317722	0.929	0.768	2.65E-22	Epi-B1
HMGN1	1.52E-26	0.301299	0.968	0.898	2.77E-22	Epi-B1
SLC7A8	1.89E-26	0.251465	0.539	0.244	3.44E-22	Epi-B1
CDV3	3.43E-26	0.253977	0.567	0.262	6.24E-22	Epi-B1
DDB2	3.46E-26	0.324251	0.667	0.358	6.31E-22	Epi-B1
SUMO2	3.99E-26	0.267578	1	0.967	7.27E-22	Epi-B1
HNRNPAB	6.31E-26	0.326421	0.816	0.519	1.15E-21	Epi-B1

MDH1	7.14E-26	0.31357	0.961	0.772	1.30E-21	Epi-B1
CCT5	8.50E-26	0.289972	0.869	0.607	1.55E-21	Epi-B1
GNB2L1	1.06E-25	0.252304	1	0.99	1.93E-21	Epi-B1
NPC2	1.31E-25	0.274648	1	0.867	2.38E-21	Epi-B1
EEF1G	1.37E-25	0.274849	0.996	0.988	2.49E-21	Epi-B1
TPI1	2.39E-25	0.262477	0.996	0.967	4.34E-21	Epi-B1
STRA13	2.76E-25	0.33851	0.83	0.562	5.03E-21	Epi-B1
LYPD6B	3.28E-25	0.26486	0.613	0.31	5.97E-21	Epi-B1
SUPT16H	3.82E-25	0.258266	0.603	0.299	6.96E-21	Epi-B1
TMEM100	4.00E-25	0.309474	0.585	0.291	7.29E-21	Epi-B1
ARHGDIB	5.44E-25	0.324874	0.759	0.433	9.90E-21	Epi-B1
SNRPF	1.27E-24	0.304787	0.926	0.736	2.30E-20	Epi-B1
HSPB11	1.74E-24	0.326665	0.883	0.599	3.17E-20	Epi-B1
PTGES3	1.87E-24	0.293512	0.975	0.846	3.41E-20	Epi-B1
HMGN3	3.55E-24	0.333241	0.989	0.93	6.46E-20	Epi-B1
TUBB6	7.89E-24	0.320065	0.819	0.519	1.44E-19	Epi-B1
SUCO	1.07E-23	0.336008	0.826	0.542	1.94E-19	Epi-B1
EEF1B2	1.49E-23	0.279928	0.986	0.902	2.71E-19	Epi-B1
FUS	1.59E-23	0.270116	0.837	0.52	2.90E-19	Epi-B1
EFEMP1	1.70E-23	0.258746	0.617	0.31	3.10E-19	Epi-B1
SRSF7	4.12E-23	0.335122	0.957	0.852	7.50E-19	Epi-B1
TNFRSF12A	1.01E-22	0.360927	0.67	0.381	1.83E-18	Epi-B1
HNRNPF	1.08E-22	0.275948	0.972	0.819	1.96E-18	Epi-B1
RAB13	2.02E-22	0.252941	0.855	0.588	3.67E-18	Epi-B1
NUDCD2	2.46E-22	0.265109	0.752	0.457	4.48E-18	Epi-B1
FDPS	3.17E-22	0.280442	0.801	0.509	5.77E-18	Epi-B1
SRSF2	3.40E-22	0.284765	0.986	0.881	6.19E-18	Epi-B1
MAGOH	3.41E-22	0.251917	0.883	0.592	6.21E-18	Epi-B1
EBP	3.48E-22	0.256771	0.702	0.395	6.34E-18	Epi-B1
TUBA1C	6.02E-22	0.456737	0.816	0.592	1.10E-17	Epi-B1
PSMA2	7.45E-22	0.257137	0.975	0.883	1.36E-17	Epi-B1
TXNDC12	7.93E-22	0.264716	0.77	0.469	1.44E-17	Epi-B1
GSPT1	1.15E-21	0.270649	0.809	0.508	2.09E-17	Epi-B1
H1FX	1.52E-21	0.331881	0.709	0.418	2.77E-17	Epi-B1
SLC25A5	3.78E-21	0.352681	0.979	0.873	6.88E-17	Epi-B1
CRNDE	4.42E-21	0.332337	0.624	0.35	8.05E-17	Epi-B1
RBMX	6.20E-21	0.277754	0.84	0.618	1.13E-16	Epi-B1
RPA2	1.50E-20	0.281225	0.77	0.48	2.73E-16	Epi-B1
PSMA4	3.07E-20	0.290694	0.95	0.792	5.60E-16	Epi-B1
AVPI1	7.12E-20	0.319846	0.805	0.526	1.30E-15	Epi-B1
CRTAC1	9.90E-20	0.326078	0.95	0.697	1.80E-15	Epi-B1
CAPG	1.09E-19	0.256803	0.957	0.828	1.98E-15	Epi-B1
VPS29	1.12E-19	0.275444	0.954	0.806	2.03E-15	Epi-B1
CALM2	1.33E-19	0.345794	0.996	0.979	2.42E-15	Epi-B1
ANAPC11	1.33E-19	0.25291	0.989	0.855	2.42E-15	Epi-B1
VDAC1	2.05E-19	0.260884	0.954	0.774	3.74E-15	Epi-B1
IMPDH2	2.19E-19	0.264271	0.716	0.462	3.99E-15	Epi-B1
RAB4A	2.26E-19	0.274926	0.936	0.734	4.12E-15	Epi-B1

TKT	2.33E-19	0.342045	0.996	0.865	4.25E-15	Epi-B1
SFPQ	3.26E-19	0.284769	0.872	0.64	5.93E-15	Epi-B1
CTSL2	8.64E-19	0.346614	0.922	0.648	1.57E-14	Epi-B1
GAS5	2.09E-18	0.343632	0.989	0.934	3.80E-14	Epi-B1
ETFB	2.30E-18	0.251607	0.883	0.697	4.19E-14	Epi-B1
RHEB	6.55E-18	0.261918	0.915	0.783	1.19E-13	Epi-B1
SNRPG	5.17E-17	0.257624	0.989	0.915	9.41E-13	Epi-B1
LMNA	9.13E-17	0.291688	1	0.966	1.66E-12	Epi-B1
SIVA1	1.13E-16	0.264872	0.858	0.596	2.06E-12	Epi-B1
GYG1	1.43E-16	0.272616	0.844	0.588	2.61E-12	Epi-B1
NQO1	3.21E-15	0.278013	1	0.946	5.84E-11	Epi-B1
KRT18	1.44E-14	0.275777	0.95	0.74	2.62E-10	Epi-B1
NCL	2.15E-14	0.253018	0.926	0.807	3.92E-10	Epi-B1
TGFBI	2.93E-13	0.302615	0.972	0.902	5.33E-09	Epi-B1
SERPINB5	1.15E-11	0.255266	0.918	0.72	2.10E-07	Epi-B1
ALDH3A1	4.02E-11	0.280628	0.996	0.977	7.32E-07	Epi-B1
LY6D	1.07E-05	0.27757	0.596	0.434	0.194297	Epi-B1
AQP5	0	2.835223	1	0.194	0	Conj-3
CEACAM7	0	1.479685	0.658	0.013	0	Conj-3
KRT7	0	1.366083	0.824	0.073	0	Conj-3
CEACAM6	0	0.864116	0.619	0.037	0	Conj-3
SERPINA1	0	0.823939	0.601	0.029	0	Conj-3
CEACAM5	0	0.81823	0.468	0.019	0	Conj-3
VSIG2	0	0.789629	0.658	0.031	0	Conj-3
GCNT3	0	0.775803	0.612	0.022	0	Conj-3
SLC34A2	0	0.752095	0.579	0.037	0	Conj-3
SLC6A14	0	0.370978	0.288	0.008	0	Conj-3
MB	0	0.339596	0.32	0.013	0	Conj-3
MUC1	6.15E-303	0.681877	0.55	0.046	1.12E-298	Conj-3
MMP7	2.26E-284	0.358682	0.259	0.009	4.12E-280	Conj-3
ATP6V1C2	6.66E-282	0.349171	0.255	0.009	1.21E-277	Conj-3
LRRC26	4.83E-280	0.33296	0.273	0.011	8.79E-276	Conj-3
KRT4	6.56E-272	2.074603	0.946	0.188	1.20E-267	Conj-3
LCN2	2.57E-270	2.343971	0.95	0.21	4.67E-266	Conj-3
SPRR3	1.97E-252	1.129042	0.313	0.016	3.58E-248	Conj-3
FAM3B	1.70E-246	1.187869	0.863	0.162	3.10E-242	Conj-3
F3	1.36E-245	1.6563	0.978	0.236	2.48E-241	Conj-3
DEGS2	1.15E-231	0.84501	0.655	0.09	2.09E-227	Conj-3
SLC40A1	6.09E-225	0.314066	0.299	0.017	1.11E-220	Conj-3
CP	2.99E-223	0.729998	0.676	0.095	5.44E-219	Conj-3
NCCRP1	8.22E-218	0.636873	0.453	0.043	1.50E-213	Conj-3
PSCA	5.90E-207	1.968382	0.853	0.183	1.07E-202	Conj-3
CXCL17	1.21E-197	1.793704	0.996	0.395	2.21E-193	Conj-3
FAM3D	1.74E-196	1.064799	0.853	0.191	3.17E-192	Conj-3
S100A9	2.51E-192	2.799856	0.989	0.392	4.58E-188	Conj-3
ATP10B	3.79E-192	0.297207	0.317	0.023	6.91E-188	Conj-3
WFDC2	9.03E-191	1.080516	0.576	0.082	1.64E-186	Conj-3
LYPD2	2.41E-189	2.077437	0.719	0.133	4.39E-185	Conj-3

ALOX5	1.35E-182	0.399205	0.388	0.037	2.47E-178	Conj-3
PP14571	1.90E-178	0.396068	0.345	0.03	3.46E-174	Conj-3
BCAS1	3.87E-174	0.639658	0.619	0.102	7.04E-170	Conj-3
S100P	1.86E-171	0.814071	0.378	0.038	3.39E-167	Conj-3
GDF15	3.22E-166	0.625738	0.324	0.028	5.86E-162	Conj-3
BARX2	4.53E-164	0.645805	0.676	0.121	8.25E-160	Conj-3
MDK	1.07E-160	1.102435	0.896	0.274	1.94E-156	Conj-3
SLPI	5.69E-149	1.455801	0.996	0.451	1.04E-144	Conj-3
LOC440335	1.42E-148	0.702373	0.576	0.101	2.58E-144	Conj-3
CYP4B1	1.12E-146	0.519262	0.478	0.069	2.04E-142	Conj-3
MUC21	1.43E-146	0.819472	0.673	0.136	2.60E-142	Conj-3
MUC20	5.38E-142	1.037372	0.928	0.361	9.79E-138	Conj-3
FBP1	1.20E-140	0.553875	0.568	0.102	2.19E-136	Conj-3
PRSS22	1.83E-140	0.731957	0.719	0.166	3.33E-136	Conj-3
OSR2	5.94E-140	0.953569	0.874	0.249	1.08E-135	Conj-3
TMC5	1.49E-139	0.412984	0.399	0.051	2.70E-135	Conj-3
C11orf92	2.05E-136	0.305605	0.331	0.035	3.73E-132	Conj-3
S100A11	7.75E-135	1.140769	1	0.985	1.41E-130	Conj-3
MSLN	3.12E-134	1.024596	0.817	0.248	5.69E-130	Conj-3
CTSS	3.13E-130	0.707157	0.799	0.227	5.69E-126	Conj-3
HIST1H2BC	8.77E-128	0.912405	0.615	0.13	1.60E-123	Conj-3
HOPX	1.05E-127	1.360799	0.971	0.555	1.91E-123	Conj-3
CLDN3	1.26E-126	0.613404	0.471	0.077	2.29E-122	Conj-3
S100A8	1.72E-123	2.335563	0.91	0.41	3.12E-119	Conj-3
PDZK1IP1	4.73E-123	0.617362	0.439	0.069	8.61E-119	Conj-3
HIST1H2AC	1.47E-121	0.863004	0.701	0.176	2.68E-117	Conj-3
TJP3	1.12E-119	0.342983	0.414	0.061	2.04E-115	Conj-3
TFPI2	1.47E-119	0.482169	0.403	0.059	2.68E-115	Conj-3
POLR2J2	5.72E-117	1.011655	1	0.88	1.04E-112	Conj-3
FUT3	3.06E-115	0.299589	0.406	0.061	5.57E-111	Conj-3
LRG1	1.01E-113	0.38298	0.399	0.06	1.84E-109	Conj-3
POLR2J3	1.90E-112	1.013823	1	0.872	3.46E-108	Conj-3
NDRG2	1.59E-111	0.84474	0.845	0.349	2.89E-107	Conj-3
FAM105A	8.83E-110	0.496821	0.421	0.069	1.61E-105	Conj-3
RARRES3	3.27E-107	1.004161	0.903	0.406	5.95E-103	Conj-3
KRT13	1.28E-106	1.273444	1	0.647	2.32E-102	Conj-3
HIST1H2BK	1.13E-101	0.639472	0.651	0.176	2.05E-97	Conj-3
A4GALT	2.56E-97	0.923642	0.924	0.523	4.66E-93	Conj-3
SERPINB1	2.88E-97	1.2258	0.932	0.596	5.24E-93	Conj-3
B2M	1.60E-94	0.795659	1	0.997	2.91E-90	Conj-3
KLK11	7.78E-93	0.760161	0.838	0.347	1.42E-88	Conj-3
ST6GALNAC1	2.15E-92	0.363359	0.453	0.09	3.92E-88	Conj-3
CYBA	7.73E-92	0.80926	0.928	0.548	1.41E-87	Conj-3
BAG1	1.21E-91	0.793815	0.989	0.904	2.21E-87	Conj-3
ELF3	1.70E-91	0.935701	1	0.808	3.09E-87	Conj-3
ST3GAL4	3.01E-91	0.630579	0.838	0.327	5.49E-87	Conj-3
CFD	5.43E-91	1.010146	0.849	0.352	9.88E-87	Conj-3
KRT8	1.41E-90	0.938286	0.856	0.389	2.57E-86	Conj-3

AQP3	9.42E-88	0.926714	0.996	0.662	1.71E-83	Conj-3
SMIM5	2.62E-86	0.808904	0.77	0.268	4.78E-82	Conj-3
HIST2H2AA4	2.93E-86	1.088669	0.978	0.714	5.34E-82	Conj-3
LGALS3	3.75E-86	0.74294	0.996	0.989	6.82E-82	Conj-3
UCP2	5.29E-86	0.351706	0.417	0.082	9.63E-82	Conj-3
HIST1H2BG	5.91E-85	0.476269	0.46	0.099	1.08E-80	Conj-3
GBP2	7.32E-84	0.315292	0.421	0.084	1.33E-79	Conj-3
EMILIN2	1.13E-83	0.308482	0.317	0.05	2.06E-79	Conj-3
SLC16A3	5.00E-82	0.514262	0.532	0.137	9.11E-78	Conj-3
RBPMS	3.01E-80	0.251153	0.324	0.054	5.47E-76	Conj-3
LYN	9.38E-79	0.26482	0.338	0.059	1.71E-74	Conj-3
LOC645638	1.02E-78	0.591913	0.647	0.199	1.87E-74	Conj-3
DUSP4	3.34E-78	0.316137	0.32	0.054	6.07E-74	Conj-3
HIST1H2BD	7.83E-77	0.550188	0.554	0.153	1.43E-72	Conj-3
APOBEC3A	7.89E-77	1.126503	0.82	0.338	1.44E-72	Conj-3
FAM213A	1.33E-74	0.625031	0.813	0.337	2.43E-70	Conj-3
AMN	2.40E-73	0.298185	0.342	0.064	4.37E-69	Conj-3
NMB	3.20E-72	0.437648	0.424	0.098	5.83E-68	Conj-3
RERG	2.43E-71	0.469758	0.626	0.195	4.42E-67	Conj-3
MAOA	7.84E-71	0.539951	0.665	0.245	1.43E-66	Conj-3
SERINC2	9.64E-71	0.653624	0.892	0.549	1.76E-66	Conj-3
ZNF296	1.29E-70	0.449823	0.554	0.162	2.36E-66	Conj-3
C19orf33	2.99E-70	0.62213	1	0.85	5.45E-66	Conj-3
IL1RN	4.50E-69	1.419676	0.878	0.556	8.20E-65	Conj-3
CSTA	2.70E-68	0.845669	0.978	0.76	4.91E-64	Conj-3
RAB11FIP1	4.31E-67	0.474858	0.489	0.135	7.85E-63	Conj-3
HIST1H2AE	1.18E-66	0.447089	0.478	0.126	2.15E-62	Conj-3
IFT172	3.61E-66	0.354865	0.464	0.119	6.57E-62	Conj-3
SELENBP1	8.88E-66	0.570131	0.637	0.236	1.62E-61	Conj-3
CYP27A1	1.03E-65	0.328255	0.374	0.082	1.88E-61	Conj-3
MX1	1.26E-65	0.398459	0.475	0.124	2.29E-61	Conj-3
COX7A1	8.65E-65	0.728582	0.885	0.477	1.57E-60	Conj-3
ARPC3	2.86E-64	0.559047	0.953	0.881	5.21E-60	Conj-3
CD74	3.70E-64	0.573681	0.824	0.42	6.73E-60	Conj-3
CLIC1	2.28E-63	0.526387	0.989	0.912	4.14E-59	Conj-3
B3GNT3	4.97E-63	0.27192	0.356	0.077	9.05E-59	Conj-3
TNFRSF21	7.20E-63	0.298377	0.392	0.091	1.31E-58	Conj-3
B4GALT5	2.68E-62	0.509053	0.601	0.207	4.88E-58	Conj-3
HLA-DRA	3.11E-62	0.365193	0.597	0.204	5.66E-58	Conj-3
GGT6	3.72E-62	0.417945	0.579	0.19	6.77E-58	Conj-3
PPDPF	5.16E-62	0.495938	1	0.967	9.40E-58	Conj-3
CDKN2B	6.80E-62	0.576032	0.687	0.277	1.24E-57	Conj-3
RHCG	1.28E-60	0.36915	0.367	0.083	2.32E-56	Conj-3
TMSB4X	3.03E-60	0.512746	1	0.998	5.52E-56	Conj-3
RRAD	3.27E-60	0.507439	0.46	0.128	5.96E-56	Conj-3
HES4	1.29E-57	0.574203	0.835	0.392	2.36E-53	Conj-3
TMEM45B	3.86E-57	0.255755	0.327	0.072	7.03E-53	Conj-3
CLINT1	8.95E-57	0.584821	0.809	0.43	1.63E-52	Conj-3

SYT8	3.06E-56	0.621191	0.986	0.756	5.58E-52	Conj-3
ZFP36L1	7.98E-56	0.62952	0.978	0.845	1.45E-51	Conj-3
DUSP5	1.16E-55	0.517378	0.435	0.123	2.11E-51	Conj-3
NUAK2	2.87E-55	0.672447	0.802	0.412	5.23E-51	Conj-3
C9orf16	4.86E-54	0.535314	0.978	0.858	8.85E-50	Conj-3
C15orf48	9.74E-54	0.54858	0.317	0.072	1.77E-49	Conj-3
CALM1	9.89E-54	0.481116	0.982	0.96	1.80E-49	Conj-3
H2AFJ	1.04E-53	0.621575	0.849	0.549	1.89E-49	Conj-3
SPRR1A	1.43E-53	0.749256	0.374	0.096	2.61E-49	Conj-3
MIDN	2.17E-53	0.64822	0.856	0.489	3.95E-49	Conj-3
RHOV	2.58E-52	0.599991	0.986	0.636	4.70E-48	Conj-3
DDIT4	5.08E-52	0.775725	0.809	0.458	9.26E-48	Conj-3
LGALS9	1.02E-51	0.276305	0.327	0.077	1.85E-47	Conj-3
HIST1H1C	5.24E-51	0.790941	0.568	0.219	9.53E-47	Conj-3
SOX7	5.13E-50	0.255996	0.36	0.091	9.35E-46	Conj-3
HIST2H2BE	6.35E-50	0.510957	0.622	0.253	1.16E-45	Conj-3
CLIC3	8.19E-50	0.485987	0.532	0.189	1.49E-45	Conj-3
SLC9A3R1	3.23E-48	0.472529	0.838	0.495	5.88E-44	Conj-3
DUSP2	6.49E-48	0.528558	0.5	0.174	1.18E-43	Conj-3
S100A16	3.65E-47	0.479681	0.953	0.718	6.65E-43	Conj-3
CNFN	3.69E-47	0.298307	0.414	0.122	6.71E-43	Conj-3
TMSB10	8.40E-47	0.370361	1	0.997	1.53E-42	Conj-3
SMAGP	5.69E-46	0.463243	0.741	0.378	1.04E-41	Conj-3
LMO4	8.30E-46	0.559448	0.838	0.572	1.51E-41	Conj-3
PHLDA2	9.59E-46	0.560294	0.996	0.903	1.75E-41	Conj-3
RAB25	1.44E-45	0.503637	0.878	0.582	2.62E-41	Conj-3
EPCAM	1.34E-44	0.335477	0.482	0.165	2.44E-40	Conj-3
CTSB	1.59E-44	0.460128	0.921	0.78	2.90E-40	Conj-3
KLF5	2.12E-44	0.532042	0.935	0.682	3.85E-40	Conj-3
HSPB1	2.19E-44	0.473435	1	0.985	3.98E-40	Conj-3
MSMB	2.74E-44	0.296753	0.428	0.13	4.99E-40	Conj-3
PPAP2C	5.54E-44	0.345292	0.482	0.166	1.01E-39	Conj-3
PRDX6	1.52E-43	0.463313	0.953	0.854	2.77E-39	Conj-3
COX7A2	4.93E-43	0.351747	0.989	0.976	8.98E-39	Conj-3
CD55	7.34E-43	0.690872	0.835	0.562	1.34E-38	Conj-3
TXNIP	1.50E-42	0.769039	0.906	0.673	2.73E-38	Conj-3
CLDN4	2.63E-42	0.482479	0.971	0.615	4.79E-38	Conj-3
TACSTD2	3.24E-42	0.464655	1	0.9	5.91E-38	Conj-3
CHCHD10	4.39E-42	0.411525	0.644	0.278	7.98E-38	Conj-3
LITAF	5.47E-42	0.513379	0.838	0.563	9.96E-38	Conj-3
GPR98	9.05E-42	0.382149	0.299	0.08	1.65E-37	Conj-3
BID	1.33E-41	0.400576	0.633	0.283	2.42E-37	Conj-3
MYL6	1.34E-41	0.328557	1	0.988	2.44E-37	Conj-3
REEP6	2.94E-41	0.395752	0.59	0.254	5.36E-37	Conj-3
AX747171	3.25E-41	0.339244	0.507	0.186	5.92E-37	Conj-3
ZNF593	5.25E-41	0.443653	0.694	0.328	9.55E-37	Conj-3
RHBDL2	2.21E-40	0.32923	0.536	0.212	4.02E-36	Conj-3
PIM3	2.34E-40	0.506595	0.845	0.539	4.25E-36	Conj-3

PITPNC1	4.65E-40	0.267574	0.464	0.161	8.47E-36	Conj-3
RAC1	4.88E-40	0.355919	0.968	0.954	8.89E-36	Conj-3
TC2N	8.78E-40	0.253757	0.378	0.114	1.60E-35	Conj-3
FXYD3	1.58E-39	0.410968	0.996	0.863	2.88E-35	Conj-3
SPRR1B	2.21E-39	0.70473	0.284	0.073	4.03E-35	Conj-3
BC070363	4.67E-39	0.339975	0.511	0.201	8.50E-35	Conj-3
HEBP2	6.02E-39	0.494393	0.838	0.609	1.10E-34	Conj-3
PRSS8	6.25E-39	0.431008	0.719	0.383	1.14E-34	Conj-3
PSMB10	7.48E-39	0.458533	0.788	0.464	1.36E-34	Conj-3
CRIP2	9.35E-39	0.382388	0.547	0.214	1.70E-34	Conj-3
SULT2B1	1.01E-38	0.358465	0.68	0.303	1.85E-34	Conj-3
AKR1A1	1.42E-38	0.479066	0.871	0.661	2.59E-34	Conj-3
CCDC6	2.93E-37	0.28875	0.432	0.152	5.34E-33	Conj-3
SAT1	3.40E-37	0.45737	1	0.991	6.19E-33	Conj-3
CAPNS2	5.03E-37	0.528864	0.845	0.587	9.15E-33	Conj-3
AES	5.05E-37	0.473261	0.888	0.641	9.20E-33	Conj-3
IVL	1.84E-36	0.290434	0.263	0.067	3.36E-32	Conj-3
ISG20	3.54E-36	0.510024	0.421	0.153	6.44E-32	Conj-3
HES1	6.33E-36	0.756489	0.917	0.756	1.15E-31	Conj-3
TSPAN13	6.91E-36	0.408284	0.597	0.286	1.26E-31	Conj-3
EPS8L1	1.19E-35	0.314597	0.5	0.197	2.17E-31	Conj-3
HLA-B	2.32E-35	0.551979	0.971	0.924	4.23E-31	Conj-3
COX8A	2.65E-35	0.38227	0.942	0.849	4.82E-31	Conj-3
TMC4	2.90E-35	0.335109	0.435	0.165	5.28E-31	Conj-3
NOTCH2NL	7.14E-35	0.422144	0.835	0.551	1.30E-30	Conj-3
HOMER2	8.63E-35	0.305425	0.439	0.166	1.57E-30	Conj-3
PLSCR1	8.64E-35	0.315802	0.507	0.205	1.57E-30	Conj-3
PITX1	9.95E-35	0.271899	0.349	0.109	1.81E-30	Conj-3
CASP4	1.01E-34	0.403062	0.719	0.403	1.85E-30	Conj-3
ABRACL	2.06E-34	0.392164	0.73	0.402	3.74E-30	Conj-3
DEFB1	2.37E-34	0.410504	0.759	0.396	4.32E-30	Conj-3
CTSD	3.04E-34	0.412514	0.964	0.897	5.53E-30	Conj-3
VAMP8	3.54E-34	0.408301	0.95	0.761	6.44E-30	Conj-3
PPM1K	3.70E-34	0.277209	0.392	0.135	6.73E-30	Conj-3
SOX9	4.27E-34	0.30092	0.367	0.121	7.77E-30	Conj-3
FGGY	5.20E-34	0.453716	0.669	0.358	9.47E-30	Conj-3
IGFBP3	7.68E-34	0.487753	0.511	0.203	1.40E-29	Conj-3
CCDC64B	1.22E-33	0.34324	0.529	0.229	2.23E-29	Conj-3
ATP1B1	1.81E-33	0.32862	0.529	0.226	3.30E-29	Conj-3
COX14	3.74E-33	0.396262	0.921	0.756	6.81E-29	Conj-3
CIB1	3.90E-33	0.442162	0.888	0.738	7.10E-29	Conj-3
CLDN7	1.63E-32	0.388733	0.96	0.617	2.97E-28	Conj-3
ERBB2	2.78E-32	0.286841	0.432	0.165	5.06E-28	Conj-3
SERTAD1	3.28E-32	0.439161	0.871	0.594	5.97E-28	Conj-3
C1orf210	4.29E-32	0.254244	0.406	0.151	7.81E-28	Conj-3
ZNF750	2.12E-31	0.340574	0.644	0.312	3.86E-27	Conj-3
LSP1	2.84E-31	0.353865	0.68	0.324	5.17E-27	Conj-3
CHMP4B	7.28E-31	0.384254	0.917	0.764	1.33E-26	Conj-3

CDKN1A	1.12E-30	0.420545	0.892	0.689	2.04E-26	Conj-3
KRT17	4.34E-30	0.759358	0.716	0.417	7.90E-26	Conj-3
SERF2	4.48E-30	0.268661	1	0.992	8.15E-26	Conj-3
SH3GLB2	5.63E-30	0.254068	0.406	0.154	1.03E-25	Conj-3
EIF6	6.84E-30	0.386709	0.939	0.769	1.25E-25	Conj-3
ARHGDIB	6.90E-30	0.410415	0.73	0.434	1.26E-25	Conj-3
TMEM40	9.87E-30	0.390825	0.619	0.335	1.80E-25	Conj-3
ST6GALNAC6	1.51E-29	0.269902	0.41	0.16	2.75E-25	Conj-3
CHMP1B	2.81E-29	0.438283	0.899	0.739	5.12E-25	Conj-3
MALL	2.94E-29	0.448033	0.914	0.72	5.36E-25	Conj-3
SRD5A3	3.34E-29	0.344641	0.576	0.284	6.08E-25	Conj-3
KCNK6	5.17E-29	0.25528	0.428	0.169	9.42E-25	Conj-3
H19	1.80E-28	0.308249	0.367	0.135	3.28E-24	Conj-3
DNAJB1	4.78E-28	0.362989	0.964	0.912	8.70E-24	Conj-3
GPRC5C	6.07E-28	0.350419	0.594	0.3	1.10E-23	Conj-3
ETS2	6.27E-28	0.34431	0.662	0.353	1.14E-23	Conj-3
TOM1	7.26E-28	0.282246	0.486	0.214	1.32E-23	Conj-3
GMDS	7.31E-28	0.331469	0.669	0.37	1.33E-23	Conj-3
EVA1A	9.04E-28	0.278833	0.453	0.194	1.65E-23	Conj-3
TNNI2	1.16E-27	0.348314	0.55	0.266	2.12E-23	Conj-3
LAMTOR5	1.48E-27	0.32446	0.957	0.896	2.69E-23	Conj-3
COX5B	2.56E-27	0.270324	1	0.962	4.66E-23	Conj-3
EHF	3.42E-27	0.44805	0.626	0.331	6.22E-23	Conj-3
CTAGE5	4.02E-27	0.252078	0.457	0.194	7.31E-23	Conj-3
SCGB2A1	4.12E-27	0.545546	0.745	0.442	7.51E-23	Conj-3
HSBP1L1	5.00E-27	0.416329	0.773	0.519	9.11E-23	Conj-3
C1GALT1	1.22E-26	0.286725	0.493	0.228	2.23E-22	Conj-3
ANXA11	1.90E-26	0.339716	0.921	0.771	3.46E-22	Conj-3
UBE2L6	4.42E-26	0.312678	0.594	0.31	8.06E-22	Conj-3
C1orf106	4.80E-26	0.257592	0.403	0.163	8.74E-22	Conj-3
PDLIM5	4.87E-26	0.309994	0.583	0.289	8.87E-22	Conj-3
CALML3	8.82E-26	0.471015	0.896	0.699	1.61E-21	Conj-3
TNFSF10	1.66E-25	0.405869	0.856	0.649	3.02E-21	Conj-3
ZNF503	1.71E-25	0.250082	0.457	0.201	3.12E-21	Conj-3
GADD45B	2.75E-25	0.36874	0.971	0.927	5.01E-21	Conj-3
UQCRB	2.84E-25	0.267365	0.982	0.968	5.18E-21	Conj-3
SLC27A5	6.45E-25	0.293263	0.363	0.146	1.17E-20	Conj-3
CCDC12	6.69E-25	0.377499	0.838	0.609	1.22E-20	Conj-3
DYNLT1	9.16E-25	0.336509	0.935	0.804	1.67E-20	Conj-3
GRN	2.14E-24	0.378479	0.881	0.763	3.89E-20	Conj-3
DDIT3	2.31E-24	0.508844	0.773	0.545	4.21E-20	Conj-3
PDLIM1	2.62E-24	0.358584	0.917	0.77	4.77E-20	Conj-3
RBP1	3.17E-24	0.257759	0.317	0.119	5.76E-20	Conj-3
RHOBTB3	3.62E-24	0.26434	0.464	0.206	6.59E-20	Conj-3
ENSG0000019876	3.98E-24	0.413969	1	0.983	7.26E-20	Conj-3
LOC100505633	4.03E-24	0.301279	0.604	0.328	7.34E-20	Conj-3
BLOC1S1	6.19E-24	0.320425	0.863	0.625	1.13E-19	Conj-3
CRIP1	7.39E-24	0.819881	0.899	0.749	1.35E-19	Conj-3

PTGR1	9.78E-24	0.410935	0.633	0.399	1.78E-19	Conj-3
CCDC80	1.46E-23	0.264439	0.327	0.126	2.65E-19	Conj-3
UAP1	1.56E-23	0.31446	0.647	0.355	2.84E-19	Conj-3
HLA-C	2.54E-23	0.384253	0.957	0.956	4.63E-19	Conj-3
SERPINB2	4.25E-23	0.434208	0.313	0.115	7.74E-19	Conj-3
TNNT3	4.81E-23	0.315371	0.737	0.451	8.76E-19	Conj-3
FAM46B	7.01E-23	0.324647	0.637	0.362	1.28E-18	Conj-3
RARRES1	7.42E-23	0.712824	0.439	0.194	1.35E-18	Conj-3
ID1	8.21E-23	0.403924	0.982	0.959	1.49E-18	Conj-3
SPINT1	8.83E-23	0.31154	0.878	0.646	1.61E-18	Conj-3
ARPC1B	1.09E-22	0.348695	0.791	0.597	1.98E-18	Conj-3
ENSG0000019888	1.16E-22	0.402827	0.996	0.96	2.11E-18	Conj-3
COX6C	1.17E-22	0.29639	0.993	0.967	2.14E-18	Conj-3
TSPAN1	1.20E-22	0.502035	0.781	0.535	2.18E-18	Conj-3
LY6E	2.96E-22	0.294005	0.831	0.538	5.40E-18	Conj-3
ENSG0000019884	5.62E-22	0.436535	0.986	0.882	1.02E-17	Conj-3
SLC35C1	5.66E-22	0.273274	0.453	0.22	1.03E-17	Conj-3
PRDX5	1.45E-21	0.255719	1	0.976	2.65E-17	Conj-3
PIK3IP1	1.70E-21	0.271909	0.633	0.371	3.10E-17	Conj-3
HS3ST1	3.08E-21	0.322923	0.385	0.166	5.60E-17	Conj-3
EFNA1	4.90E-21	0.41525	0.863	0.699	8.92E-17	Conj-3
UQCRQ	4.99E-21	0.270909	0.982	0.949	9.09E-17	Conj-3
ROMO1	8.05E-21	0.341315	0.903	0.78	1.47E-16	Conj-3
CD82	1.05E-20	0.317368	0.777	0.553	1.90E-16	Conj-3
C11orf31	1.28E-20	0.304645	0.914	0.785	2.32E-16	Conj-3
PLK2	1.61E-20	0.400446	0.773	0.528	2.94E-16	Conj-3
TRIP10	2.32E-20	0.287813	0.594	0.347	4.23E-16	Conj-3
ZC3H12A	2.71E-20	0.336261	0.504	0.265	4.93E-16	Conj-3
GADD45G	5.50E-20	0.426611	0.637	0.407	1.00E-15	Conj-3
RASD1	5.58E-20	0.415002	0.673	0.439	1.02E-15	Conj-3
MDH2	6.88E-20	0.260975	0.835	0.619	1.25E-15	Conj-3
NFKBIZ	7.41E-20	0.328944	0.579	0.34	1.35E-15	Conj-3
NRARP	7.99E-20	0.28923	0.396	0.18	1.46E-15	Conj-3
PSMB8	1.46E-19	0.302987	0.701	0.478	2.65E-15	Conj-3
OVOL1	2.16E-19	0.27244	0.629	0.36	3.93E-15	Conj-3
PNKD	2.17E-19	0.29663	0.888	0.743	3.95E-15	Conj-3
P4HB	3.18E-19	0.303582	0.795	0.616	5.80E-15	Conj-3
UQCR10	4.08E-19	0.263625	0.968	0.902	7.43E-15	Conj-3
GSTT1	4.78E-19	0.28205	0.511	0.275	8.71E-15	Conj-3
MGST1	8.24E-19	0.454489	0.468	0.253	1.50E-14	Conj-3
AREG	8.28E-19	0.367867	0.701	0.441	1.51E-14	Conj-3
SRSF5	9.00E-19	0.280992	0.964	0.938	1.64E-14	Conj-3
DHCR24	9.15E-19	0.255816	0.403	0.191	1.67E-14	Conj-3
COX6A1	1.11E-18	0.256751	1	0.977	2.02E-14	Conj-3
CLEC2B	1.75E-18	0.266329	0.871	0.638	3.18E-14	Conj-3
TRAF4	2.70E-18	0.352992	0.604	0.362	4.92E-14	Conj-3
SF3B3	2.73E-18	0.27738	0.295	0.124	4.97E-14	Conj-3
COX6B1	4.73E-18	0.264415	0.975	0.949	8.61E-14	Conj-3

FMO1	5.63E-18	0.296664	0.385	0.176	1.03E-13	Conj-3
S100A14	8.36E-18	0.256674	0.978	0.776	1.52E-13	Conj-3
PYGL	1.00E-17	0.294361	0.701	0.48	1.83E-13	Conj-3
PPIC	1.15E-17	0.276398	0.543	0.309	2.10E-13	Conj-3
CASP3	1.41E-17	0.267187	0.475	0.26	2.57E-13	Conj-3
TALDO1	1.55E-17	0.259262	0.885	0.8	2.83E-13	Conj-3
ZFP36	2.08E-17	0.270261	0.993	0.975	3.78E-13	Conj-3
ENSG0000021008[illegible]	2.13E-17	0.368013	1	1	3.88E-13	Conj-3
GSTK1	3.47E-17	0.308784	0.773	0.63	6.32E-13	Conj-3
TCEA3	4.93E-17	0.282581	0.719	0.496	8.98E-13	Conj-3
IFI27	1.26E-16	0.577322	0.741	0.527	2.30E-12	Conj-3
PGD	2.42E-16	0.271437	0.759	0.581	4.41E-12	Conj-3
SCAND1	4.87E-16	0.287396	0.863	0.711	8.87E-12	Conj-3
ZFAS1	7.16E-16	0.263518	0.942	0.865	1.30E-11	Conj-3
NDUFB2	9.70E-16	0.283756	0.957	0.863	1.77E-11	Conj-3
SERP1	1.00E-15	0.269064	0.903	0.834	1.83E-11	Conj-3
COMTD1	1.40E-15	0.282671	0.482	0.289	2.55E-11	Conj-3
ENSG0000021145[illegible]	1.50E-15	0.432069	1	0.997	2.74E-11	Conj-3
MRPS18A	1.63E-15	0.262305	0.561	0.33	2.96E-11	Conj-3
TMED3	1.98E-15	0.253619	0.874	0.779	3.60E-11	Conj-3
PSME2	2.91E-15	0.271933	0.842	0.679	5.31E-11	Conj-3
PVRL4	2.98E-15	0.257616	0.755	0.502	5.43E-11	Conj-3
ISG15	4.16E-15	0.320522	0.378	0.186	7.58E-11	Conj-3
ARL4A	5.26E-15	0.260467	0.795	0.614	9.57E-11	Conj-3
BBC3	8.36E-15	0.306617	0.342	0.17	1.52E-10	Conj-3
EPHA2	1.33E-14	0.389347	0.514	0.303	2.42E-10	Conj-3
NDUFS7	1.49E-14	0.275822	0.781	0.602	2.72E-10	Conj-3
CEBPB	3.02E-14	0.306979	0.835	0.644	5.49E-10	Conj-3
ASAH1	4.95E-14	0.300515	0.799	0.671	9.02E-10	Conj-3
SHFM1	6.75E-14	0.2657	0.964	0.86	1.23E-09	Conj-3
SEPW1	7.10E-14	0.295972	0.594	0.39	1.29E-09	Conj-3
PDCD4	9.28E-14	0.317192	0.849	0.709	1.69E-09	Conj-3
TPD52L1	1.09E-13	0.25177	0.77	0.551	1.98E-09	Conj-3
SLC1A5	1.51E-13	0.254305	0.651	0.457	2.76E-09	Conj-3
NOL3	2.30E-13	0.252881	0.558	0.347	4.19E-09	Conj-3
UNC5B-AS1	2.57E-13	0.429948	0.514	0.316	4.69E-09	Conj-3
BTG2	6.16E-13	0.282983	0.813	0.649	1.12E-08	Conj-3
TPM4	8.97E-13	0.275951	0.701	0.489	1.63E-08	Conj-3
BIK	4.10E-12	0.253578	0.5	0.317	7.46E-08	Conj-3
HINT2	5.70E-11	0.256546	0.712	0.555	1.04E-06	Conj-3
NR4A1	1.87E-08	0.251323	0.619	0.451	0.000341	Conj-3
ANXA1	4.65E-07	0.25526	0.996	0.983	0.008459	Conj-3
PLAUR	6.48E-06	0.272611	0.406	0.288	0.118048	Conj-3
TM4SF1	0.004687	0.464461	0.745	0.686	1	Conj-3
GPHA2	0	4.430929	0.977	0.089	0	LPC-1
SCRG1	0	3.379752	0.837	0.015	0	LPC-1
FRZB	0	2.374968	0.702	0.006	0	LPC-1
PTN	0	1.529795	0.744	0.085	0	LPC-1

FAM107A	0	1.208996	0.698	0.071	0	LPC-1
LECT1	0	1.205382	0.368	0.001	0	LPC-1
NPPC	0	1.119087	0.519	0.004	0	LPC-1
GPHB5	0	1.05201	0.461	0.002	0	LPC-1
CDH19	0	0.815304	0.465	0.002	0	LPC-1
LINC00681	0	0.579657	0.318	0.007	0	LPC-1
NELL1	0	0.561537	0.364	0.004	0	LPC-1
PDE1A	0	0.483091	0.318	0.009	0	LPC-1
LRRC3B	0	0.420148	0.302	0.006	0	LPC-1
GMPR	3.51E-293	0.931374	0.64	0.065	6.39E-289	LPC-1
MMP10	2.06E-247	1.06567	0.477	0.04	3.75E-243	LPC-1
ITM2A	7.98E-209	0.482828	0.295	0.017	1.45E-204	LPC-1
KRT15	3.03E-175	2.176398	1	0.423	5.51E-171	LPC-1
SPARCL1	1.86E-172	1.332277	0.942	0.258	3.39E-168	LPC-1
SLC6A6	5.58E-169	1.206638	0.705	0.148	1.02E-164	LPC-1
LGALS3BP	8.46E-145	1.419891	0.95	0.498	1.54E-140	LPC-1
OLFM1	1.52E-144	0.58128	0.442	0.059	2.78E-140	LPC-1
GAPDH	6.94E-139	1.164991	1	0.998	1.26E-134	LPC-1
S100A2	1.24E-131	1.309599	0.981	0.422	2.26E-127	LPC-1
FAM46A	1.27E-131	1.48365	0.907	0.422	2.31E-127	LPC-1
RPL5	3.12E-126	0.852979	1	0.993	5.69E-122	LPC-1
RPL34	1.14E-120	0.666029	1	1	2.08E-116	LPC-1
ITM2C	3.65E-118	0.411353	0.291	0.03	6.64E-114	LPC-1
EEF1A1	4.43E-117	0.717079	1	0.999	8.07E-113	LPC-1
DKK3	8.71E-114	1.409494	0.965	0.642	1.59E-109	LPC-1
PLTP	5.28E-113	0.725798	0.554	0.121	9.62E-109	LPC-1
GAS5	2.11E-111	1.039072	0.996	0.934	3.84E-107	LPC-1
RPLP1	1.97E-107	0.581576	1	1	3.59E-103	LPC-1
EGFL7	5.00E-106	0.50783	0.345	0.048	9.10E-102	LPC-1
RPL13	3.00E-105	0.619565	1	0.999	5.47E-101	LPC-1
RPL15	3.97E-105	0.52047	1	0.998	7.23E-101	LPC-1
C17orf76-AS1	2.49E-103	0.812669	0.996	0.964	4.53E-99	LPC-1
IGFBP7	5.85E-103	1.588717	0.981	0.653	1.07E-98	LPC-1
ISYNA1	1.14E-102	0.49552	0.36	0.054	2.08E-98	LPC-1
RPS27	1.79E-102	0.578346	1	1	3.25E-98	LPC-1
RPL35A	3.98E-102	0.555623	0.996	0.998	7.25E-98	LPC-1
RPL3	3.94E-101	0.52855	1	0.999	7.18E-97	LPC-1
EEF1G	2.35E-100	0.670595	1	0.988	4.28E-96	LPC-1
CXCL1	5.16E-99	1.073378	0.562	0.126	9.39E-95	LPC-1
COL9A3	5.87E-99	0.452274	0.302	0.04	1.07E-94	LPC-1
RPS27A	1.40E-98	0.603198	1	0.997	2.55E-94	LPC-1
NPC2	2.47E-98	0.87767	0.977	0.868	4.49E-94	LPC-1
RPL11	6.09E-97	0.493387	1	0.999	1.11E-92	LPC-1
RPL10	1.05E-96	0.558922	1	1	1.91E-92	LPC-1
RPL13A	4.52E-95	0.525373	1	0.999	8.24E-91	LPC-1
RPL31	2.35E-93	0.555972	0.996	0.998	4.28E-89	LPC-1
RPL6	3.03E-92	0.624474	1	0.992	5.52E-88	LPC-1
RPL32	3.95E-92	0.550342	1	0.999	7.19E-88	LPC-1

PTK2B	8.18E-92	0.471418	0.329	0.05	1.49E-87	LPC-1
RPL14	2.41E-91	0.591861	0.996	0.995	4.40E-87	LPC-1
CXCL14	4.96E-91	1.161002	0.996	0.889	9.04E-87	LPC-1
NAP1L1	1.90E-90	0.894836	0.93	0.732	3.46E-86	LPC-1
SNCA	2.17E-90	0.338472	0.26	0.031	3.96E-86	LPC-1
RPS3A	4.68E-90	0.54035	1	0.998	8.52E-86	LPC-1
CERCAM	6.03E-90	0.446298	0.376	0.066	1.10E-85	LPC-1
RPS23	1.81E-88	0.484495	1	0.998	3.30E-84	LPC-1
RPL30	3.37E-88	0.519335	1	0.998	6.14E-84	LPC-1
RPS6	1.00E-86	0.552789	1	0.999	1.83E-82	LPC-1
KRT14	3.44E-86	0.906606	1	0.573	6.27E-82	LPC-1
VIM	2.59E-85	0.791125	0.899	0.322	4.72E-81	LPC-1
RPS12	3.11E-85	0.556936	1	0.999	5.66E-81	LPC-1
HLA-C	2.11E-84	0.743249	0.996	0.956	3.83E-80	LPC-1
TIMP1	1.33E-83	0.899673	0.957	0.637	2.41E-79	LPC-1
NPM1	3.87E-83	0.735164	0.992	0.927	7.05E-79	LPC-1
RPL22	2.16E-81	0.532872	1	0.994	3.94E-77	LPC-1
RPS3	7.89E-80	0.533133	1	0.996	1.44E-75	LPC-1
RPS9	1.46E-79	0.473582	1	0.998	2.65E-75	LPC-1
PLD3	1.80E-79	1.032122	0.857	0.542	3.28E-75	LPC-1
HLA-A	2.20E-79	0.773521	0.992	0.961	4.00E-75	LPC-1
CTSF	9.22E-79	0.776763	0.841	0.376	1.68E-74	LPC-1
GEM	8.84E-78	0.607456	0.31	0.051	1.61E-73	LPC-1
APP	8.95E-78	1.003787	0.884	0.562	1.63E-73	LPC-1
RPL37	1.69E-77	0.512665	1	0.995	3.07E-73	LPC-1
RPL4	1.94E-77	0.542033	0.992	0.986	3.53E-73	LPC-1
ENSG0000019893	2.37E-77	0.713878	1	0.998	4.32E-73	LPC-1
RPS4X	1.28E-76	0.537939	1	0.999	2.33E-72	LPC-1
RPL21	2.01E-76	0.452135	1	0.999	3.67E-72	LPC-1
SOX9	1.86E-75	0.618761	0.477	0.12	3.38E-71	LPC-1
RPLP2	2.74E-75	0.465296	1	0.999	4.99E-71	LPC-1
MEIS2	6.96E-74	0.586993	0.422	0.099	1.27E-69	LPC-1
RPS15	1.24E-73	0.402436	1	0.999	2.26E-69	LPC-1
JUN	1.68E-73	0.824505	0.996	0.979	3.05E-69	LPC-1
RPS14	1.80E-73	0.425585	1	1	3.29E-69	LPC-1
RPL12	1.63E-72	0.467285	1	0.998	2.98E-68	LPC-1
RPL8	3.17E-72	0.423674	1	0.998	5.77E-68	LPC-1
FOS	4.30E-69	0.768013	1	0.974	7.82E-65	LPC-1
RPL17	2.10E-68	0.512484	0.988	0.985	3.82E-64	LPC-1
RPS15A	5.19E-68	0.463381	1	0.997	9.45E-64	LPC-1
RPL23A	6.22E-68	0.416513	1	0.998	1.13E-63	LPC-1
RPL27A	2.10E-67	0.414579	1	0.998	3.83E-63	LPC-1
RPL7	1.04E-66	0.418003	1	0.998	1.89E-62	LPC-1
HLA-B	1.06E-66	0.762842	0.988	0.924	1.94E-62	LPC-1
C6orf48	3.37E-66	0.792712	0.891	0.647	6.14E-62	LPC-1
GLTSCR2	1.88E-65	0.712308	0.95	0.826	3.42E-61	LPC-1
RPS13	2.14E-65	0.388157	1	0.998	3.90E-61	LPC-1
RPL19	4.79E-65	0.384136	1	0.999	8.71E-61	LPC-1

RPL39	1.32E-63	0.381317	1	0.999	2.41E-59	LPC-1
RPL26	2.11E-63	0.401075	1	0.999	3.84E-59	LPC-1
ID2	3.80E-63	1.05513	0.864	0.555	6.92E-59	LPC-1
SPINK2	4.97E-63	0.560919	0.364	0.084	9.05E-59	LPC-1
RPS25	9.94E-63	0.393367	1	0.998	1.81E-58	LPC-1
RPL36	2.03E-62	0.420018	0.996	0.997	3.70E-58	LPC-1
FCGRT	9.52E-62	0.691618	0.671	0.305	1.73E-57	LPC-1
TPM2	2.26E-61	0.422984	0.298	0.057	4.12E-57	LPC-1
COL18A1	5.01E-61	0.394225	0.333	0.071	9.12E-57	LPC-1
ENSG0000021145	9.18E-61	0.796974	1	0.997	1.67E-56	LPC-1
RPS20	1.69E-60	0.406754	0.996	0.997	3.07E-56	LPC-1
SNAI2	3.27E-60	0.804529	0.721	0.356	5.96E-56	LPC-1
PTMA	5.20E-59	0.495292	1	0.996	9.47E-55	LPC-1
CITED2	6.11E-59	0.985729	0.891	0.646	1.11E-54	LPC-1
NDUFA4L2	4.55E-58	0.788232	0.93	0.651	8.28E-54	LPC-1
RPL10A	4.84E-58	0.393542	1	0.995	8.81E-54	LPC-1
RPS8	2.52E-57	0.391296	1	0.999	4.59E-53	LPC-1
EFEMP2	2.74E-56	0.373415	0.326	0.072	5.00E-52	LPC-1
PHLDA1	5.81E-56	0.664788	0.574	0.201	1.06E-51	LPC-1
NENF	1.95E-55	0.69629	0.868	0.71	3.56E-51	LPC-1
WFDC2	6.00E-55	0.485508	0.357	0.086	1.09E-50	LPC-1
FTL	1.29E-54	0.594523	1	0.996	2.34E-50	LPC-1
NACA	1.63E-54	0.3634	1	0.996	2.97E-50	LPC-1
APOE	2.06E-54	0.33212	0.271	0.053	3.74E-50	LPC-1
RBP1	2.20E-54	0.466959	0.411	0.117	4.01E-50	LPC-1
RPS7	6.47E-54	0.410539	1	0.993	1.18E-49	LPC-1
RPL41	7.93E-54	0.309769	1	1	1.44E-49	LPC-1
RPS19	2.95E-53	0.404298	1	0.998	5.37E-49	LPC-1
ENSG0000021008	4.85E-53	0.72293	1	1	8.82E-49	LPC-1
CXCL3	6.28E-53	0.526573	0.349	0.083	1.14E-48	LPC-1
ENSG0000019876	3.27E-52	0.555662	1	0.983	5.95E-48	LPC-1
RPS11	4.33E-52	0.456419	0.996	0.985	7.89E-48	LPC-1
RPS17L	2.14E-51	0.380946	1	0.996	3.89E-47	LPC-1
UBA52	2.13E-50	0.390105	1	0.987	3.88E-46	LPC-1
RPL18	9.73E-50	0.380464	1	0.996	1.77E-45	LPC-1
CD59	1.32E-49	0.580245	0.93	0.731	2.41E-45	LPC-1
SERTAD4	3.23E-49	0.471059	0.349	0.093	5.89E-45	LPC-1
IVNS1ABP	2.24E-48	0.700717	0.756	0.455	4.08E-44	LPC-1
ATP1B1	1.51E-47	0.607316	0.547	0.226	2.74E-43	LPC-1
SNHG6	1.54E-47	0.557629	0.915	0.758	2.80E-43	LPC-1
SERPINF1	1.88E-47	0.403476	0.88	0.52	3.42E-43	LPC-1
SPATS2L	1.31E-46	0.603807	0.647	0.341	2.38E-42	LPC-1
SNHG8	3.42E-46	0.605053	0.857	0.698	6.22E-42	LPC-1
DLX4	3.56E-46	0.297032	0.267	0.059	6.48E-42	LPC-1
C12orf57	4.00E-46	0.566043	0.895	0.74	7.28E-42	LPC-1
RPS21	4.32E-46	0.376575	1	0.989	7.87E-42	LPC-1
GNB2L1	9.27E-45	0.359131	0.996	0.99	1.69E-40	LPC-1
OS9	1.23E-44	0.689755	0.818	0.639	2.24E-40	LPC-1

PFDN5	2.72E-44	0.433933	0.988	0.974	4.95E-40	LPC-1
EPB41L4A-AS1	2.91E-44	0.587652	0.767	0.521	5.30E-40	LPC-1
KRT8	6.22E-44	0.518339	0.748	0.392	1.13E-39	LPC-1
EIF3E	6.44E-44	0.529211	0.907	0.821	1.17E-39	LPC-1
RPS16	6.60E-44	0.354798	1	0.994	1.20E-39	LPC-1
PSAP	6.61E-44	0.556967	0.973	0.953	1.20E-39	LPC-1
EIF3L	2.42E-42	0.512944	0.919	0.817	4.41E-38	LPC-1
RPS5	7.32E-42	0.372883	0.996	0.99	1.33E-37	LPC-1
RPS10	9.03E-42	0.398575	0.992	0.978	1.64E-37	LPC-1
SSR4	3.08E-41	0.534901	0.95	0.894	5.61E-37	LPC-1
ZFAS1	1.80E-40	0.487708	0.957	0.865	3.27E-36	LPC-1
GLCE	2.04E-40	0.555346	0.512	0.226	3.71E-36	LPC-1
RPS28	5.46E-40	0.326486	1	0.999	9.93E-36	LPC-1
SELM	6.53E-40	0.355819	0.729	0.337	1.19E-35	LPC-1
NTRK2	6.87E-40	0.58654	0.636	0.297	1.25E-35	LPC-1
RPL24	1.49E-39	0.35317	0.992	0.994	2.71E-35	LPC-1
RPL7A	3.98E-39	0.340605	0.996	0.987	7.24E-35	LPC-1
MALAT1	1.12E-38	0.47352	1	0.998	2.04E-34	LPC-1
LTB4R	1.12E-38	0.387963	0.337	0.103	2.04E-34	LPC-1
RPL9	1.42E-38	0.519695	0.919	0.785	2.58E-34	LPC-1
MYL9	1.53E-38	0.34683	0.31	0.085	2.78E-34	LPC-1
RPS18	2.12E-38	0.287923	1	1	3.87E-34	LPC-1
EEF1D	2.66E-38	0.375395	0.984	0.978	4.84E-34	LPC-1
FBXO2	2.67E-38	0.487918	0.488	0.21	4.86E-34	LPC-1
NUCB1	1.17E-36	0.558567	0.632	0.381	2.13E-32	LPC-1
COMMD6	2.64E-36	0.452751	0.957	0.925	4.81E-32	LPC-1
CBR1	3.53E-36	0.528727	0.853	0.688	6.43E-32	LPC-1
FOSB	8.39E-36	0.552987	0.926	0.83	1.53E-31	LPC-1
ATP1B3	9.69E-36	0.56502	0.93	0.823	1.76E-31	LPC-1
RPL36A	1.55E-35	0.351063	1	0.989	2.82E-31	LPC-1
DEGS1	2.34E-35	0.456488	0.682	0.375	4.26E-31	LPC-1
RPL29	3.96E-35	0.33956	0.996	0.993	7.21E-31	LPC-1
ENSG0000019888	4.88E-35	0.494538	0.992	0.96	8.88E-31	LPC-1
TPT1	1.30E-34	0.306501	1	0.997	2.37E-30	LPC-1
RPL23	1.41E-34	0.33195	0.992	0.985	2.57E-30	LPC-1
CPXM2	2.51E-34	0.689916	0.674	0.439	4.57E-30	LPC-1
TOMM7	9.06E-34	0.388447	0.981	0.969	1.65E-29	LPC-1
RPS24	9.63E-34	0.296336	0.996	0.998	1.75E-29	LPC-1
STXBP6	1.12E-33	0.361298	0.264	0.073	2.05E-29	LPC-1
SNORA33	4.99E-33	0.440511	0.888	0.766	9.09E-29	LPC-1
BAMBI	5.12E-33	0.494053	0.411	0.168	9.32E-29	LPC-1
TMEM98	1.02E-32	0.448306	0.411	0.164	1.86E-28	LPC-1
SNRPN	3.78E-32	0.512938	0.76	0.581	6.88E-28	LPC-1
PIGT	4.85E-32	0.545528	0.632	0.395	8.84E-28	LPC-1
AKR1C1	4.93E-32	0.396529	0.512	0.222	8.97E-28	LPC-1
CMTM7	8.87E-32	0.33724	0.318	0.106	1.62E-27	LPC-1
NRN1	1.53E-31	0.475041	0.291	0.093	2.79E-27	LPC-1
LAMB3	1.58E-31	0.493129	0.531	0.264	2.88E-27	LPC-1

RPL37A	3.73E-31	0.277839	1	0.998	6.78E-27	LPC-1
RPL38	5.80E-31	0.300641	0.992	0.989	1.06E-26	LPC-1
NUCB2	6.11E-31	0.507093	0.601	0.333	1.11E-26	LPC-1
MSS51	2.20E-30	0.354256	0.992	0.979	4.00E-26	LPC-1
ATRAID	6.30E-30	0.456569	0.876	0.802	1.15E-25	LPC-1
CDH13	7.73E-30	0.372093	0.368	0.14	1.41E-25	LPC-1
HEXA	3.12E-29	0.529544	0.519	0.289	5.68E-25	LPC-1
ENSG0000019872	7.91E-29	0.392936	1	0.993	1.44E-24	LPC-1
SLC22A17	1.68E-28	0.439563	0.477	0.239	3.07E-24	LPC-1
EIF4A2	2.25E-28	0.407124	0.926	0.902	4.10E-24	LPC-1
CSAD	3.69E-28	0.507496	0.422	0.199	6.72E-24	LPC-1
RGS2	4.41E-28	0.457695	0.519	0.275	8.03E-24	LPC-1
SYNE2	4.60E-28	0.520136	0.605	0.386	8.38E-24	LPC-1
P4HB	5.67E-28	0.531809	0.748	0.617	1.03E-23	LPC-1
PPP1R15A	7.23E-28	0.430067	0.969	0.916	1.32E-23	LPC-1
ATP1A1	1.37E-27	0.494201	0.756	0.587	2.50E-23	LPC-1
NFIB	2.04E-27	0.471567	0.38	0.163	3.72E-23	LPC-1
AKR1C3	2.21E-27	0.336471	0.318	0.114	4.02E-23	LPC-1
NME4	2.95E-27	0.373004	0.391	0.171	5.37E-23	LPC-1
HMGN3	1.07E-26	0.362921	0.988	0.93	1.94E-22	LPC-1
PABPC1	1.30E-26	0.423396	0.934	0.902	2.37E-22	LPC-1
PDLIM1	1.55E-25	0.384824	0.903	0.77	2.82E-21	LPC-1
CXCL2	2.80E-25	0.519314	0.492	0.231	5.10E-21	LPC-1
ZFP36	3.20E-25	0.454145	0.988	0.976	5.83E-21	LPC-1
DUSP1	5.07E-25	0.458767	0.996	0.988	9.23E-21	LPC-1
FBL	5.85E-25	0.495302	0.678	0.549	1.07E-20	LPC-1
ESD	8.15E-25	0.419556	0.702	0.532	1.48E-20	LPC-1
RPS14P3	1.60E-24	0.328518	0.969	0.962	2.91E-20	LPC-1
TXNIP	2.34E-24	0.490078	0.845	0.674	4.25E-20	LPC-1
MATN2	3.13E-24	0.44667	0.5	0.282	5.70E-20	LPC-1
ETFB	3.51E-24	0.459887	0.775	0.699	6.38E-20	LPC-1
OAT	4.62E-24	0.394618	0.601	0.379	8.41E-20	LPC-1
CYP27A1	9.07E-24	0.264159	0.252	0.085	1.65E-19	LPC-1
GOLGA8B	2.03E-23	0.335698	0.291	0.112	3.70E-19	LPC-1
TUBA1A	2.05E-23	0.610794	0.798	0.708	3.73E-19	LPC-1
DST	6.67E-23	0.419969	0.605	0.361	1.21E-18	LPC-1
CFI	8.08E-22	0.475462	0.442	0.252	1.47E-17	LPC-1
B2M	1.06E-21	0.265579	1	0.997	1.93E-17	LPC-1
CADM1	1.94E-21	0.444242	0.388	0.195	3.53E-17	LPC-1
TFAP2A	2.20E-21	0.465381	0.57	0.384	4.00E-17	LPC-1
LMNA	2.30E-21	0.417385	0.984	0.967	4.19E-17	LPC-1
EPAS1	3.70E-21	0.396927	0.512	0.304	6.74E-17	LPC-1
HOPX	4.51E-21	0.276596	0.81	0.558	8.22E-17	LPC-1
RNASE4	5.11E-21	0.267407	0.547	0.29	9.31E-17	LPC-1
L32131	5.47E-21	0.346639	0.849	0.744	9.96E-17	LPC-1
CD44	2.35E-20	0.350307	0.717	0.582	4.29E-16	LPC-1
COL17A1	2.86E-20	0.314267	0.76	0.484	5.21E-16	LPC-1
HSPA2	5.13E-20	0.417432	0.605	0.405	9.35E-16	LPC-1

FMO1	5.09E-19	0.297157	0.384	0.177	9.26E-15	LPC-1
CYP2S1	7.64E-19	0.277727	0.264	0.105	1.39E-14	LPC-1
TRA2B	1.86E-18	0.381252	0.837	0.794	3.39E-14	LPC-1
SLC2A1	2.22E-18	0.460079	0.93	0.859	4.04E-14	LPC-1
ENSG0000019888(	3.17E-18	0.252795	1	0.998	5.78E-14	LPC-1
LSR	3.35E-18	0.445614	0.593	0.444	6.10E-14	LPC-1
LRPAP1	5.21E-18	0.382994	0.748	0.685	9.49E-14	LPC-1
ENSG0000019889!	2.90E-17	0.254234	1	0.987	5.28E-13	LPC-1
BEX2	4.94E-17	0.345325	0.516	0.337	9.00E-13	LPC-1
CCNI	1.06E-16	0.371336	0.678	0.539	1.93E-12	LPC-1
PEBP1	1.61E-16	0.283645	0.977	0.972	2.93E-12	LPC-1
PAX6	1.62E-16	0.319183	0.973	0.799	2.95E-12	LPC-1
TPD52L1	3.12E-16	0.328787	0.709	0.552	5.67E-12	LPC-1
VKORC1	3.13E-16	0.415558	0.554	0.409	5.71E-12	LPC-1
GRN	4.64E-16	0.371986	0.806	0.765	8.45E-12	LPC-1
ANP32B	5.76E-16	0.36291	0.756	0.737	1.05E-11	LPC-1
MAGED1	1.09E-15	0.320132	0.372	0.21	1.98E-11	LPC-1
GPX2	1.44E-15	0.39879	0.558	0.385	2.63E-11	LPC-1
EFEMP1	1.84E-15	0.450715	0.484	0.312	3.36E-11	LPC-1
ZNF503	3.00E-15	0.294716	0.372	0.203	5.47E-11	LPC-1
ARL4A	3.34E-15	0.374419	0.74	0.615	6.08E-11	LPC-1
SPINT2	4.31E-15	0.27394	0.988	0.835	7.84E-11	LPC-1
FKBP8	4.84E-15	0.36355	0.725	0.668	8.81E-11	LPC-1
FBLN1	7.93E-15	0.317413	0.453	0.293	1.44E-10	LPC-1
HSPA1B	2.10E-14	0.366884	0.783	0.711	3.82E-10	LPC-1
RAB4A	2.72E-14	0.311788	0.802	0.737	4.95E-10	LPC-1
EIF3H	2.73E-14	0.300285	0.895	0.846	4.97E-10	LPC-1
BCAM	4.57E-14	0.256871	0.535	0.328	8.33E-10	LPC-1
DUSP6	6.30E-14	0.338083	0.364	0.207	1.15E-09	LPC-1
SEPT9	7.55E-14	0.250203	0.318	0.168	1.37E-09	LPC-1
BOC	8.10E-14	0.363577	0.337	0.186	1.47E-09	LPC-1
ERGIC3	9.11E-14	0.337457	0.717	0.659	1.66E-09	LPC-1
PHGDH	1.14E-13	0.363984	0.523	0.385	2.08E-09	LPC-1
TMEM173	1.33E-13	0.276486	0.267	0.129	2.43E-09	LPC-1
DANCR	1.50E-13	0.322923	0.729	0.654	2.74E-09	LPC-1
GNAL	1.54E-13	0.272181	0.283	0.143	2.80E-09	LPC-1
FOXP1	2.50E-13	0.256323	0.965	0.945	4.54E-09	LPC-1
NPM3	3.16E-13	0.292522	0.341	0.196	5.76E-09	LPC-1
PRKCSH	6.26E-13	0.313125	0.407	0.267	1.14E-08	LPC-1
IER2	6.53E-13	0.260581	0.996	0.996	1.19E-08	LPC-1
SAT2	2.66E-12	0.30359	0.566	0.45	4.84E-08	LPC-1
LAMP1	3.52E-12	0.279821	0.841	0.814	6.40E-08	LPC-1
NUCKS1	4.75E-12	0.288553	0.802	0.774	8.64E-08	LPC-1
SF1	7.70E-12	0.286666	0.492	0.359	1.40E-07	LPC-1
EMC10	8.09E-12	0.298556	0.496	0.378	1.47E-07	LPC-1
ID3	9.66E-12	0.356325	0.818	0.759	1.76E-07	LPC-1
IER3	1.30E-11	0.326344	0.884	0.831	2.37E-07	LPC-1
BEND5	1.39E-11	0.315846	0.341	0.208	2.53E-07	LPC-1

TMED10	2.49E-11	0.301184	0.826	0.816	4.53E-07	LPC-1
CYB5R3	3.69E-11	0.295303	0.531	0.422	6.72E-07	LPC-1
LOC100129195	5.19E-11	0.261864	0.264	0.139	9.45E-07	LPC-1
CHID1	5.37E-11	0.324104	0.419	0.301	9.77E-07	LPC-1
PHF1	5.83E-11	0.293861	0.399	0.272	1.06E-06	LPC-1
LAMP2	6.78E-11	0.29555	0.713	0.672	1.23E-06	LPC-1
IFRD1	7.27E-11	0.327451	0.519	0.41	1.32E-06	LPC-1
SLC3A2	8.02E-11	0.256064	0.814	0.788	1.46E-06	LPC-1
ENSG0000019884(	8.32E-11	0.28134	0.938	0.883	1.52E-06	LPC-1
RSL1D1	8.41E-11	0.286822	0.717	0.688	1.53E-06	LPC-1
PCBP2	9.17E-11	0.264054	0.841	0.816	1.67E-06	LPC-1
KRTCAP3	1.74E-10	0.27165	0.411	0.286	3.16E-06	LPC-1
SOCS3	2.11E-10	0.34109	0.469	0.331	3.84E-06	LPC-1
PPT1	2.27E-10	0.276237	0.667	0.581	4.13E-06	LPC-1
FSCN1	3.44E-10	0.289651	0.376	0.245	6.27E-06	LPC-1
ATF3	3.64E-10	0.261586	0.779	0.699	6.63E-06	LPC-1
DPP7	8.10E-10	0.258532	0.578	0.474	1.47E-05	LPC-1
CRNDE	1.15E-09	0.259859	0.492	0.353	2.10E-05	LPC-1
PNISR	1.25E-09	0.270952	0.698	0.66	2.27E-05	LPC-1
GUSB	1.45E-09	0.28772	0.403	0.288	2.64E-05	LPC-1
KMT2E	3.92E-09	0.252682	0.643	0.587	7.14E-05	LPC-1
DNAJC4	5.13E-09	0.283103	0.407	0.305	9.34E-05	LPC-1
CLSTN1	5.90E-09	0.321912	0.496	0.4	0.000107	LPC-1
GPNMB	1.04E-08	0.347138	0.674	0.55	0.000189	LPC-1
GAA	1.53E-08	0.263991	0.267	0.16	0.000278	LPC-1
SNHG7	1.79E-08	0.252707	0.655	0.599	0.000326	LPC-1
SH3BGRL	2.92E-08	0.267812	0.647	0.637	0.000532	LPC-1
FAM60A	5.23E-08	0.288282	0.45	0.359	0.000952	LPC-1
PDIA6	5.81E-08	0.25247	0.76	0.767	0.001059	LPC-1
EMX2	1.46E-07	0.291965	0.38	0.27	0.002664	LPC-1
MRPS6	1.53E-07	0.269378	0.62	0.588	0.002793	LPC-1
PLK2	2.42E-07	0.257836	0.616	0.53	0.0044	LPC-1
PHIP	4.02E-07	0.275445	0.372	0.279	0.007324	LPC-1
PRRC2C	4.04E-07	0.2795	0.562	0.519	0.007351	LPC-1
ARL2	1.03E-06	0.254831	0.512	0.463	0.01882	LPC-1
TGFBI	1.68E-06	0.280533	0.996	0.901	0.030616	LPC-1
NFKBIA	4.92E-06	0.260906	0.884	0.944	0.089672	LPC-1
CO9	2.13E-05	0.255476	0.43	0.363	0.388612	LPC-1
TOB1	0.003894	0.260746	0.663	0.687	1	LPC-1
HLA-DPA1	0	4.133391	1	0.09	0	LC
HLA-DPB1	0	4.092833	0.992	0.077	0	LC
RGS1	0	3.928151	0.977	0.005	0	LC
CCL3	0	3.569543	0.869	0.004	0	LC
LAPTM5	0	3.079728	0.977	0.015	0	LC
TYROBP	0	3.055224	1	0.004	0	LC
SRGN	0	2.98859	0.931	0.029	0	LC
HLA-DQA1	0	2.712115	0.931	0.007	0	LC
GPR183	0	2.554712	0.831	0.002	0	LC

FCER1G	0	2.325049	0.938	0.005	0	LC
AIF1	0	2.267234	0.823	0.002	0	LC
CCL4	0	2.218806	0.515	0.001	0	LC
CXCR4	0	2.208692	0.808	0.004	0	LC
IL1B	0	2.183816	0.523	0.01	0	LC
C1QA	0	2.17435	0.631	0.002	0	LC
HLA-DRB6	0	2.15595	0.723	0.031	0	LC
FCER1A	0	2.118797	0.654	0.003	0	LC
ALOX5AP	0	2.084345	0.815	0.001	0	LC
C1QC	0	1.920988	0.523	0.001	0	LC
LST1	0	1.903189	0.854	0.002	0	LC
CCL4L2	0	1.850983	0.392	0	0	LC
HLA-DMB	0	1.81013	0.785	0.019	0	LC
C1QB	0	1.744548	0.469	0.001	0	LC
MS4A6A	0	1.725233	0.738	0.001	0	LC
FCGR2A	0	1.670301	0.738	0.001	0	LC
PLEK	0	1.591219	0.638	0	0	LC
BCL2A1	0	1.46927	0.531	0.008	0	LC
FAM26F	0	1.46918	0.662	0.014	0	LC
C1orf162	0	1.442825	0.623	0.005	0	LC
HCST	0	1.429567	0.685	0.018	0	LC
ITGB2	0	1.402435	0.638	0.003	0	LC
E02193	0	1.382744	0.469	0.001	0	LC
RNASE6	0	1.280836	0.608	0.001	0	LC
MS4A7	0	1.267077	0.562	0.001	0	LC
SPI1	0	1.250395	0.538	0.003	0	LC
LY86	0	1.249993	0.615	0.001	0	LC
FCGR3A	0	1.238989	0.508	0	0	LC
FCGR2B	0	1.228467	0.508	0.001	0	LC
CD37	0	1.213313	0.592	0.005	0	LC
CYTIP	0	1.197614	0.454	0.001	0	LC
CLEC10A	0	1.181552	0.4	0	0	LC
CD86	0	1.168324	0.554	0.002	0	LC
MEF2C	0	1.139164	0.515	0.005	0	LC
OLR1	0	1.116283	0.385	0	0	LC
EVI2B	0	1.110733	0.538	0.001	0	LC
HCLS1	0	1.108072	0.477	0.001	0	LC
CD53	0	1.10221	0.508	0.001	0	LC
CLEC5A	0	1.064998	0.415	0	0	LC
MNDA	0	1.060348	0.454	0.001	0	LC
GLIPR1	0	1.059271	0.446	0.004	0	LC
TREM2	0	1.045185	0.385	0	0	LC
CD69	0	1.034915	0.362	0.003	0	LC
VSIG4	0	1.007226	0.423	0	0	LC
GMFG	0	0.98607	0.477	0.004	0	LC
P2RY13	0	0.984398	0.362	0	0	LC
NAPSB	0	0.957797	0.423	0.002	0	LC
TAGAP	0	0.943392	0.415	0.005	0	LC

PTPRC	0	0.939523	0.408	0	0	LC
CD163	0	0.931694	0.323	0.001	0	LC
EVI2A	0	0.910294	0.385	0.004	0	LC
HLA-DQB2	0	0.906998	0.315	0.001	0	LC
DOK2	0	0.886508	0.323	0	0	LC
CORO1A	0	0.885476	0.408	0.004	0	LC
GGTA1P	0	0.883661	0.408	0.006	0	LC
HLA-DQA2	0	0.873504	0.346	0	0	LC
LCP1	0	0.854059	0.362	0.004	0	LC
CXorf21	0	0.83429	0.338	0.001	0	LC
MSR1	0	0.830585	0.315	0	0	LC
BST2	0	0.80806	0.423	0.011	0	LC
AMICA1	0	0.807765	0.338	0.001	0	LC
HHEX	0	0.790284	0.331	0.004	0	LC
CD300A	0	0.771697	0.369	0.001	0	LC
LAT2	0	0.760834	0.354	0.003	0	LC
CSF1R	0	0.749202	0.346	0	0	LC
PLD4	0	0.72825	0.292	0	0	LC
HAVCR2	0	0.717219	0.338	0.001	0	LC
IGSF6	0	0.710682	0.3	0.001	0	LC
TBXAS1	0	0.69746	0.331	0.001	0	LC
HOTAIRM1	0	0.657432	0.269	0	0	LC
IRF8	0	0.656636	0.285	0	0	LC
FPR1	0	0.644836	0.331	0.004	0	LC
CSF2RA	0	0.617728	0.292	0.004	0	LC
TREM1	0	0.606118	0.269	0.002	0	LC
FGL2	0	0.603271	0.292	0.001	0	LC
RAC2	0	0.602074	0.277	0.002	0	LC
WAS	5.43E-308	0.526896	0.262	0.004	9.89E-304	LC
HSPA6	8.09E-288	2.906588	0.569	0.027	1.47E-283	LC
HLA-DMA	9.08E-263	2.249489	0.954	0.101	1.65E-258	LC
HSPA7	1.01E-246	1.52303	0.308	0.008	1.85E-242	LC
CD83	2.03E-239	2.398131	0.815	0.076	3.70E-235	LC
HLA-DQB1	7.55E-237	3.072491	0.992	0.127	1.38E-232	LC
COTL1	2.90E-193	1.906138	0.854	0.109	5.27E-189	LC
HLA-DRB1	1.18E-185	3.795778	1	0.178	2.15E-181	LC
IL8	1.66E-181	3.489674	0.831	0.102	3.02E-177	LC
CD68	4.06E-173	1.455681	0.723	0.081	7.39E-169	LC
PTGER4	2.38E-169	1.3294	0.485	0.034	4.34E-165	LC
HLA-DRA	2.56E-167	4.113041	1	0.205	4.66E-163	LC
NCF4	2.01E-162	0.682461	0.315	0.014	3.65E-158	LC
ARRB2	5.56E-146	1.175151	0.646	0.076	1.01E-141	LC
ADAM28	1.17E-133	0.615763	0.308	0.017	2.13E-129	LC
G0S2	2.36E-129	2.86415	0.6	0.072	4.30E-125	LC
PHACTR1	1.46E-126	0.948841	0.362	0.025	2.65E-122	LC
TNF	1.78E-125	1.448093	0.554	0.062	3.24E-121	LC
RGCC	1.33E-122	1.595746	0.731	0.112	2.42E-118	LC
SERPINA1	6.18E-121	0.930893	0.423	0.036	1.13E-116	LC

SERPINB9	3.51E-112	0.760681	0.262	0.014	6.39E-108	LC
RGS2	3.94E-108	2.870791	0.923	0.274	7.17E-104	LC
C3	1.34E-107	0.624003	0.262	0.015	2.44E-103	LC
GPSM3	1.39E-107	0.775095	0.392	0.035	2.52E-103	LC
CD74	3.65E-103	3.984999	0.992	0.422	6.65E-99	LC
IL1R2	3.81E-99	0.887083	0.277	0.018	6.93E-95	LC
RGS10	6.39E-95	1.428585	0.692	0.141	1.16E-90	LC
CXCL16	1.38E-90	1.560129	0.8	0.215	2.51E-86	LC
DUSP2	1.26E-85	1.777955	0.738	0.175	2.29E-81	LC
SH3KBP1	2.24E-85	0.626425	0.323	0.029	4.08E-81	LC
HLA-DRB5	2.73E-85	2.264308	0.538	0.087	4.98E-81	LC
LINC00936	2.65E-81	1.431162	0.677	0.156	4.83E-77	LC
RNASET2	1.16E-79	1.8308	0.923	0.434	2.10E-75	LC
CD14	6.17E-78	1.516364	0.608	0.124	1.12E-73	LC
FTL	1.33E-77	1.606744	1	0.996	2.41E-73	LC
OGFRL1	8.42E-76	0.896915	0.469	0.072	1.53E-71	LC
ID2	1.90E-72	2.024171	0.977	0.556	3.45E-68	LC
SPP1	1.44E-71	2.406655	0.346	0.039	2.63E-67	LC
SEPT6	3.66E-62	0.525728	0.277	0.029	6.67E-58	LC
KLF6	3.47E-61	1.740367	0.938	0.675	6.32E-57	LC
PTPRE	5.41E-60	0.90537	0.4	0.064	9.84E-56	LC
MALAT1	1.02E-59	0.85361	1	0.998	1.85E-55	LC
NR4A2	3.73E-57	1.501379	0.654	0.197	6.79E-53	LC
HSD17B11	4.26E-57	1.060925	0.515	0.113	7.76E-53	LC
VMO1	1.48E-55	1.216828	0.446	0.083	2.70E-51	LC
CTSS	1.68E-54	1.022818	0.692	0.233	3.06E-50	LC
VIM	2.61E-54	0.698095	0.985	0.326	4.75E-50	LC
RPS27	1.68E-53	0.675002	1	1	3.05E-49	LC
NFKBIA	5.74E-52	1.481944	0.977	0.942	1.05E-47	LC
LGALS1	9.00E-51	1.019847	0.908	0.324	1.64E-46	LC
EMP3	1.04E-50	1.091544	0.785	0.276	1.90E-46	LC
IFIT2	1.22E-50	0.674811	0.308	0.044	2.23E-46	LC
FYB	2.27E-49	0.728652	0.369	0.064	4.14E-45	LC
CYBA	5.36E-48	1.361904	0.854	0.552	9.75E-44	LC
RPS27A	1.01E-47	0.688229	1	0.997	1.83E-43	LC
GPX1	1.48E-47	1.024465	0.985	0.946	2.69E-43	LC
RPLP2	4.11E-47	0.615682	1	0.999	7.49E-43	LC
RNF130	8.86E-47	1.027677	0.546	0.157	1.61E-42	LC
RPS19	5.95E-45	0.716014	1	0.998	1.08E-40	LC
TMSB10	7.96E-43	0.765809	1	0.997	1.45E-38	LC
SGK1	6.50E-42	1.645178	0.892	0.709	1.18E-37	LC
NPC2	1.15E-41	1.074848	0.931	0.869	2.10E-37	LC
C15orf48	1.24E-40	1.08327	0.369	0.074	2.26E-36	LC
TPT1	2.03E-40	0.727199	0.985	0.997	3.69E-36	LC
RPL36A	2.79E-40	0.716689	1	0.989	5.09E-36	LC
ALOX5	3.15E-40	0.532897	0.269	0.041	5.73E-36	LC
LY96	5.82E-40	0.530321	0.3	0.051	1.06E-35	LC
TNFSF13B	6.13E-40	0.555127	0.292	0.049	1.12E-35	LC

RPL26	1.28E-39	0.540772	1	0.999	2.33E-35	LC
FXYD5	2.83E-39	0.93249	0.523	0.154	5.15E-35	LC
MAFB	1.11E-38	1.311692	0.877	0.611	2.02E-34	LC
RPS16	1.83E-38	0.623928	0.992	0.994	3.33E-34	LC
RPS29	5.25E-37	0.596799	1	0.996	9.55E-33	LC
RPL39	1.40E-36	0.463561	1	0.999	2.55E-32	LC
PLIN2	3.60E-36	1.106004	0.485	0.146	6.55E-32	LC
RPS23	3.35E-35	0.532319	1	0.998	6.11E-31	LC
HIF1A	9.36E-35	1.217288	0.485	0.158	1.70E-30	LC
CXCL3	6.25E-34	2.4675	0.362	0.085	1.14E-29	LC
KCTD12	6.28E-34	0.71979	0.354	0.08	1.14E-29	LC
RPL10	6.91E-34	0.464771	1	1	1.26E-29	LC
MSN	7.72E-34	0.735081	0.392	0.101	1.41E-29	LC
HIST2H2AA4	8.89E-34	1.207114	0.931	0.717	1.62E-29	LC
PABPC1	2.83E-33	0.858079	0.962	0.902	5.15E-29	LC
SOD2	6.51E-33	1.593751	0.654	0.319	1.19E-28	LC
RPL21	2.17E-32	0.477598	1	0.999	3.95E-28	LC
GADD45B	2.36E-32	0.967576	0.962	0.928	4.29E-28	LC
RPL18A	2.58E-32	0.471372	1	0.997	4.70E-28	LC
RPS2	3.58E-32	0.478491	1	0.997	6.52E-28	LC
CXCL2	3.74E-31	2.035002	0.592	0.232	6.82E-27	LC
EEF1A1	8.79E-31	0.504756	1	1	1.60E-26	LC
FCGRT	1.30E-30	1.069121	0.623	0.308	2.37E-26	LC
GADD45G	1.63E-30	1.369046	0.715	0.409	2.97E-26	LC
SLC2A3	3.29E-30	0.768834	0.392	0.105	5.98E-26	LC
IL13RA1	8.15E-30	0.868818	0.408	0.121	1.48E-25	LC
RPL38	1.23E-29	0.554167	1	0.989	2.23E-25	LC
RPS3A	3.20E-29	0.461285	1	0.998	5.83E-25	LC
EEF1B2	3.50E-29	0.754937	0.923	0.903	6.38E-25	LC
TRA2B	6.90E-29	1.019411	0.838	0.794	1.26E-24	LC
RPL37	8.18E-29	0.525183	1	0.995	1.49E-24	LC
PLAUR	8.64E-29	0.986176	0.654	0.287	1.57E-24	LC
CITED2	1.65E-28	1.034049	0.831	0.648	3.01E-24	LC
RPS15A	3.16E-28	0.462252	1	0.997	5.75E-24	LC
CLEC7A	3.43E-28	0.953219	0.638	0.344	6.25E-24	LC
ERV3-1	7.46E-28	0.831618	0.338	0.089	1.36E-23	LC
RPL13	7.49E-28	0.430206	1	0.999	1.36E-23	LC
RPL17	7.96E-28	0.529911	0.992	0.985	1.45E-23	LC
RPL32	9.96E-28	0.417309	1	0.999	1.81E-23	LC
UBA52	1.32E-27	0.511866	0.992	0.988	2.41E-23	LC
C1orf54	1.84E-27	0.653301	0.262	0.054	3.36E-23	LC
RAB31	2.58E-27	0.549917	0.262	0.055	4.70E-23	LC
FAU	2.93E-27	0.390613	1	0.998	5.34E-23	LC
LOC284454	3.67E-27	0.785095	0.308	0.075	6.68E-23	LC
EIF1	4.38E-27	0.418283	1	1	7.98E-23	LC
RPS20	1.02E-26	0.456572	0.992	0.997	1.85E-22	LC
RPS11	2.07E-26	0.517935	0.977	0.985	3.78E-22	LC
AP1S2	2.37E-26	0.62712	0.377	0.112	4.32E-22	LC

RPS10	1.01E-25	0.561121	0.977	0.978	1.84E-21	LC
DDIT4	2.17E-25	1.046864	0.715	0.462	3.96E-21	LC
AKAP13	2.39E-25	0.823316	0.462	0.178	4.36E-21	LC
MSS51	4.37E-25	0.521035	0.992	0.979	7.96E-21	LC
RPL23	9.66E-25	0.533841	1	0.985	1.76E-20	LC
RPL31	1.07E-24	0.410817	0.992	0.998	1.95E-20	LC
RASSF4	3.94E-23	0.487741	0.269	0.066	7.17E-19	LC
HSPA1A	4.24E-23	1.270885	0.438	0.173	7.72E-19	LC
CTSH	4.79E-23	0.8541	0.531	0.265	8.71E-19	LC
AMZ2P1	5.04E-23	0.698786	0.354	0.113	9.18E-19	LC
RGS19	6.67E-23	0.516455	0.277	0.07	1.21E-18	LC
PPP1R15A	7.89E-23	0.85156	0.954	0.916	1.44E-18	LC
CMTM6	8.74E-23	0.818017	0.523	0.256	1.59E-18	LC
HSP90AA1	1.04E-22	0.981525	0.946	0.975	1.90E-18	LC
RPL27	1.42E-22	0.480735	0.992	0.994	2.59E-18	LC
RPS13	1.72E-22	0.352663	1	0.998	3.14E-18	LC
CPVL	1.94E-22	0.789571	0.492	0.191	3.53E-18	LC
RPS3	2.28E-22	0.409761	1	0.996	4.15E-18	LC
PFDN5	4.27E-22	0.556623	0.992	0.974	7.77E-18	LC
RPL34	5.47E-22	0.362687	1	1	9.96E-18	LC
RPPH1	2.97E-21	0.894172	0.377	0.134	5.42E-17	LC
IER3	5.31E-21	0.852809	0.838	0.832	9.67E-17	LC
HSPA8	8.46E-21	0.914816	0.831	0.792	1.54E-16	LC
RPL28	8.51E-21	0.436602	0.992	0.992	1.55E-16	LC
ZFP36L2	9.89E-21	1.095912	0.7	0.521	1.80E-16	LC
PTMA	1.42E-20	0.443227	1	0.996	2.58E-16	LC
HSPA1B	1.90E-20	1.65956	0.746	0.712	3.46E-16	LC
RPS6	1.94E-20	0.380939	1	0.999	3.53E-16	LC
BTG1	2.09E-20	0.856831	0.938	0.953	3.81E-16	LC
RPL19	2.46E-20	0.359481	0.992	0.999	4.49E-16	LC
HERPUD1	2.62E-20	0.793326	0.938	0.942	4.77E-16	LC
SNHG5	2.94E-20	0.662705	0.923	0.918	5.35E-16	LC
MID1IP1	5.69E-20	1.008611	0.554	0.313	1.04E-15	LC
HIST1H2AE	6.97E-20	0.800794	0.369	0.13	1.27E-15	LC
OTUD1	8.89E-20	0.914163	0.408	0.166	1.62E-15	LC
CEBPD	1.18E-19	0.985172	0.731	0.601	2.16E-15	LC
RPL27A	4.04E-19	0.365568	1	0.998	7.35E-15	LC
NEAT1	4.41E-19	0.585891	0.985	0.932	8.03E-15	LC
EEF1G	6.33E-19	0.432066	0.985	0.988	1.15E-14	LC
CTSZ	6.60E-19	0.618854	0.369	0.137	1.20E-14	LC
RILPL2	9.56E-19	0.618743	0.346	0.124	1.74E-14	LC
PLSCR1	1.28E-18	0.744441	0.446	0.209	2.33E-14	LC
CHPT1	1.39E-18	0.509829	0.277	0.081	2.52E-14	LC
PRKAG2-AS1	2.01E-18	0.592017	0.269	0.079	3.66E-14	LC
YWHAH	3.15E-18	1.027846	0.692	0.604	5.74E-14	LC
NFKBIZ	3.18E-18	0.928329	0.569	0.342	5.78E-14	LC
RPL6	3.55E-18	0.356786	1	0.992	6.46E-14	LC
ARHGDIB	9.10E-18	0.832313	0.631	0.437	1.66E-13	LC

UPP1	9.48E-18	0.76839	0.415	0.186	1.73E-13	LC
RPL11	1.00E-17	0.326436	1	0.999	1.82E-13	LC
FAM46A	2.99E-17	1.004484	0.623	0.428	5.45E-13	LC
RPL13A	6.68E-17	0.316561	0.992	0.999	1.22E-12	LC
TXNIP	7.33E-17	1.015252	0.815	0.675	1.33E-12	LC
RPS25	8.76E-17	0.296525	1	0.999	1.59E-12	LC
HIST2H2AC	1.71E-16	0.672371	0.323	0.121	3.11E-12	LC
RPS9	1.81E-16	0.305096	1	0.998	3.30E-12	LC
RPL30	2.72E-16	0.340235	1	0.998	4.96E-12	LC
RPL12	2.81E-16	0.31314	1	0.998	5.12E-12	LC
ATN1	3.85E-16	0.82472	0.338	0.133	7.01E-12	LC
PHLDA1	4.45E-16	1.046924	0.438	0.205	8.10E-12	LC
CMTM3	1.14E-15	0.447477	0.269	0.087	2.08E-11	LC
PFN1	1.29E-15	0.594931	0.938	0.986	2.35E-11	LC
RPS21	1.79E-15	0.389734	0.992	0.99	3.26E-11	LC
RPS14	1.82E-15	0.321004	1	1	3.31E-11	LC
FAM49B	2.44E-15	0.707159	0.477	0.277	4.45E-11	LC
MYLIP	2.88E-15	0.898293	0.538	0.351	5.24E-11	LC
RUNX1	5.56E-15	0.572986	0.331	0.133	1.01E-10	LC
NFKBIE	7.13E-15	0.636046	0.338	0.14	1.30E-10	LC
RPL5	7.75E-15	0.317762	0.985	0.993	1.41E-10	LC
MCL1	8.83E-15	0.810607	0.769	0.699	1.61E-10	LC
CNPY3	1.60E-14	0.59801	0.346	0.15	2.91E-10	LC
RPL23A	2.42E-14	0.327139	0.992	0.999	4.40E-10	LC
CD44	2.62E-14	0.732944	0.669	0.584	4.77E-10	LC
SEC11A	2.74E-14	0.629678	0.715	0.713	4.99E-10	LC
SNHG6	3.66E-14	0.592752	0.777	0.76	6.67E-10	LC
RPS7	7.46E-14	0.321945	0.977	0.993	1.36E-09	LC
KCNQ1OT1	9.07E-14	0.722739	0.423	0.223	1.65E-09	LC
MTDH	9.87E-14	0.553367	0.669	0.573	1.80E-09	LC
C1orf63	1.14E-13	0.848132	0.623	0.513	2.08E-09	LC
IRF2BP2	1.32E-13	0.721223	0.531	0.352	2.41E-09	LC
RHOG	2.25E-13	0.671905	0.454	0.274	4.09E-09	LC
COMMD6	2.33E-13	0.512462	0.862	0.926	4.23E-09	LC
TNFAIP8	3.41E-13	0.624768	0.315	0.133	6.22E-09	LC
ETS2	4.85E-13	0.891785	0.523	0.357	8.83E-09	LC
CLK1	5.12E-13	0.717843	0.677	0.61	9.32E-09	LC
MEF2A	5.55E-13	0.510519	0.346	0.16	1.01E-08	LC
IRF1	5.63E-13	0.71826	0.538	0.377	1.02E-08	LC
EIF3F	6.31E-13	0.483773	0.746	0.773	1.15E-08	LC
UBC	6.62E-13	0.429983	0.992	0.999	1.21E-08	LC
HSP90AB1	7.57E-13	0.71667	0.923	0.964	1.38E-08	LC
STX11	8.20E-13	0.756315	0.315	0.136	1.49E-08	LC
HIST1H2BG	1.05E-12	0.583723	0.277	0.104	1.91E-08	LC
CELF2	1.05E-12	0.60641	0.369	0.183	1.92E-08	LC
CTSB	1.15E-12	0.658533	0.746	0.783	2.09E-08	LC
RBPJ	1.20E-12	0.647731	0.585	0.472	2.18E-08	LC
MAP3K8	1.22E-12	0.830284	0.454	0.265	2.21E-08	LC

PPT1	1.59E-12	0.596916	0.662	0.582	2.90E-08	LC
EIF5	2.96E-12	0.624473	0.738	0.72	5.38E-08	LC
SKIL	3.53E-12	0.49343	0.285	0.115	6.43E-08	LC
SAT1	1.16E-11	0.540443	1	0.991	2.11E-07	LC
HLA-E	1.74E-11	0.502966	0.854	0.885	3.16E-07	LC
IER5	1.79E-11	0.641856	0.5	0.351	3.25E-07	LC
ALDH2	2.87E-11	0.663315	0.523	0.383	5.23E-07	LC
RPL35A	3.53E-11	0.252562	1	0.998	6.43E-07	LC
LSP1	3.63E-11	0.576226	0.531	0.328	6.61E-07	LC
CKLF	4.09E-11	0.769244	0.462	0.314	7.44E-07	LC
JMJD1C	4.09E-11	0.730144	0.438	0.271	7.45E-07	LC
B2M	4.83E-11	0.349945	1	0.997	8.80E-07	LC
RIPK2	6.51E-11	0.672344	0.346	0.178	1.19E-06	LC
GRB2	6.93E-11	0.567387	0.415	0.251	1.26E-06	LC
PCBP2	1.08E-10	0.465791	0.754	0.817	1.97E-06	LC
HLA-C	1.28E-10	0.403933	0.923	0.957	2.33E-06	LC
NRP2	1.64E-10	0.566876	0.292	0.133	2.98E-06	LC
PPP1R10	2.97E-10	0.623435	0.431	0.278	5.41E-06	LC
MARCKSL1	2.99E-10	0.628467	0.431	0.248	5.45E-06	LC
ITSN2	3.33E-10	0.545256	0.369	0.21	6.07E-06	LC
ARPC3	4.83E-10	0.439268	0.838	0.882	8.79E-06	LC
TYMP	5.30E-10	0.678505	0.354	0.193	9.65E-06	LC
INSIG1	6.88E-10	0.86857	0.385	0.226	1.25E-05	LC
ZFAND5	9.22E-10	0.592408	0.685	0.664	1.68E-05	LC
DUSP6	9.30E-10	0.688713	0.369	0.208	1.69E-05	LC
ZNF503	1.11E-09	0.62175	0.362	0.204	2.03E-05	LC
SENP3-EIF4A1	1.38E-09	0.317784	0.962	0.979	2.50E-05	LC
CREM	1.60E-09	0.761563	0.354	0.204	2.91E-05	LC
ZNF331	1.82E-09	0.63683	0.269	0.125	3.31E-05	LC
DDX5	1.99E-09	0.412456	0.946	0.984	3.63E-05	LC
TMEM107	2.18E-09	0.861328	0.423	0.284	3.97E-05	LC
NXF1	2.50E-09	0.718621	0.392	0.242	4.55E-05	LC
HIST1H1C	2.82E-09	0.686533	0.385	0.224	5.13E-05	LC
HEXIM1	3.34E-09	0.728929	0.515	0.407	6.09E-05	LC
WSB1	3.55E-09	0.700062	0.623	0.607	6.46E-05	LC
CLEC2B	3.80E-09	0.56498	0.7	0.642	6.91E-05	LC
LITAF	4.61E-09	0.691964	0.6	0.568	8.40E-05	LC
ANXA5	4.70E-09	0.448469	0.585	0.416	8.56E-05	LC
C6orf48	5.16E-09	0.486539	0.662	0.651	9.40E-05	LC
NAMPT	5.66E-09	0.812013	0.485	0.394	0.000103	LC
CLIC1	8.26E-09	0.318568	0.877	0.914	0.00015	LC
ARMCX3	8.27E-09	0.384633	0.254	0.113	0.000151	LC
GABARAP	9.22E-09	0.340637	0.9	0.954	0.000168	LC
RPL29	9.32E-09	0.25189	0.992	0.993	0.00017	LC
YBX1	1.07E-08	0.265897	0.977	0.982	0.000196	LC
AKR1B1	1.16E-08	0.509906	0.577	0.467	0.00021	LC
RAB32	1.32E-08	0.51103	0.308	0.16	0.00024	LC
STK17B	1.99E-08	0.476981	0.262	0.125	0.000363	LC

BRD2	4.65E-08	0.589692	0.615	0.596	0.000846	LC
RBM3	4.82E-08	0.389302	0.869	0.916	0.000877	LC
ZC3H12A	5.11E-08	0.637039	0.408	0.268	0.00093	LC
AMD1	6.09E-08	0.536439	0.523	0.432	0.001109	LC
TPM3	8.93E-08	0.41119	0.785	0.85	0.001625	LC
ELF1	9.34E-08	0.503303	0.485	0.398	0.001701	LC
HIST1H4C	9.67E-08	0.598035	0.415	0.29	0.00176	LC
IFRD1	1.02E-07	0.693964	0.492	0.411	0.001864	LC
ARPC1B	1.03E-07	0.58804	0.577	0.601	0.001873	LC
PNRC1	1.80E-07	0.543872	0.8	0.868	0.00328	LC
SNHG8	1.82E-07	0.520909	0.692	0.701	0.003306	LC
EIF4E	2.07E-07	0.620577	0.615	0.665	0.003774	LC
CCNI	2.26E-07	0.417857	0.615	0.541	0.00412	LC
KPNA2	3.43E-07	0.613651	0.4	0.287	0.006246	LC
HLA-B	4.19E-07	0.454787	0.938	0.925	0.007633	LC
PEA15	8.12E-07	0.510459	0.485	0.434	0.014791	LC
NAP1L1	8.35E-07	0.463102	0.677	0.736	0.015196	LC
TACC1	1.00E-06	0.518161	0.338	0.221	0.018248	LC
TSPYL2	1.06E-06	0.525718	0.362	0.244	0.019365	LC
SNHG1	1.23E-06	0.623394	0.315	0.197	0.022345	LC
ZFAS1	1.60E-06	0.419906	0.785	0.867	0.029061	LC
CFLAR	1.67E-06	0.654338	0.4	0.299	0.03046	LC
B3GNT5	1.83E-06	0.485557	0.3	0.181	0.033265	LC
GLA	2.11E-06	0.432831	0.285	0.168	0.038411	LC
RPSA	2.28E-06	0.298111	0.8	0.871	0.041424	LC
SH3BGRL	2.34E-06	0.535372	0.585	0.637	0.042678	LC
UBB	2.40E-06	0.277942	0.977	0.979	0.043712	LC
SLC25A6	2.53E-06	0.38049	0.823	0.898	0.046077	LC
USP53	3.12E-06	0.452325	0.292	0.175	0.05687	LC
RBM39	3.53E-06	0.31422	0.777	0.881	0.064323	LC
HLA-A	3.76E-06	0.27661	0.892	0.962	0.068457	LC
DNAJB1	4.29E-06	1.055537	0.792	0.914	0.07814	LC
SOX4	6.13E-06	0.676412	0.462	0.382	0.111667	LC
ARRDC3	7.43E-06	0.625011	0.392	0.287	0.13534	LC
H2AFZ	1.16E-05	0.453032	0.785	0.897	0.212032	LC
HEXB	1.49E-05	0.475646	0.477	0.43	0.271567	LC
OAZ1	1.54E-05	0.273989	0.923	0.968	0.280366	LC
BID	1.65E-05	0.458745	0.385	0.288	0.299898	LC
PMAIP1	1.71E-05	0.296182	0.769	0.705	0.311541	LC
BTG2	1.77E-05	0.447526	0.638	0.652	0.322915	LC
PABPC4	1.81E-05	0.416312	0.308	0.207	0.329421	LC
ZFP36L1	1.98E-05	0.405809	0.8	0.848	0.360525	LC
SERTAD1	2.35E-05	0.47916	0.6	0.599	0.427391	LC
DUSP1	2.50E-05	0.373364	0.969	0.988	0.454709	LC
GNA15	3.12E-05	0.541085	0.354	0.262	0.567913	LC
MAP1LC3B	8.39E-05	0.432707	0.715	0.815	1	LC
ARL4A	8.90E-05	0.392721	0.631	0.617	1	LC
UBE2D3	9.32E-05	0.301729	0.838	0.919	1	LC

DRAM2	0.000139	0.388232	0.385	0.326	1	LC
SNHG15	0.0002	0.452071	0.377	0.32	1	LC
SLC7A5P2	0.000207	0.463049	0.377	0.31	1	LC
SRSF2	0.000242	0.313409	0.792	0.884	1	LC
LOC100996255	0.000261	0.353513	0.3	0.217	1	LC
IFNGR1	0.000265	0.496832	0.531	0.563	1	LC
PLK3	0.000317	0.390647	0.323	0.246	1	LC
HIST2H2BE	0.000376	0.483221	0.338	0.258	1	LC
CASP1	0.000411	0.386225	0.292	0.219	1	LC
PELI1	0.000433	0.41154	0.308	0.228	1	LC
PLBD1	0.000452	0.40102	0.285	0.201	1	LC
TUBA1A	0.000453	0.444166	0.631	0.711	1	LC
PRPF38B	0.000537	0.422364	0.485	0.519	1	LC
PER1	0.000541	0.455772	0.362	0.302	1	LC
HSPH1	0.000544	0.652611	0.377	0.34	1	LC
SAT2	0.000565	0.472912	0.454	0.452	1	LC
ZFAND2A	0.000813	0.639764	0.315	0.251	1	LC
PPIF	0.000869	0.551297	0.285	0.22	1	LC
CREG1	0.001062	0.401214	0.5	0.533	1	LC
CHORDC1	0.001097	0.486889	0.254	0.183	1	LC
MGAT1	0.001343	0.454293	0.354	0.316	1	LC
ACTB	0.00141	0.360047	0.992	0.999	1	LC
CCNL1	0.001427	0.30662	0.731	0.807	1	LC
RHOB	0.001475	0.435337	0.662	0.776	1	LC
DNAJA1	0.001932	0.433037	0.615	0.749	1	LC
HBEGF	0.001938	0.470038	0.369	0.317	1	LC
CKS2	0.002157	0.56613	0.369	0.338	1	LC
GNAI2	0.002157	0.37524	0.415	0.406	1	LC
H2AFY	0.002302	0.307195	0.592	0.705	1	LC
LPAR6	0.002342	0.560369	0.462	0.487	1	LC
CHD2	0.002665	0.363854	0.369	0.334	1	LC
PLXDC2	0.002697	0.434938	0.254	0.192	1	LC
CHCHD10	0.003143	0.286557	0.346	0.284	1	LC
HMGB2	0.003234	0.542102	0.446	0.458	1	LC
HSPA2	0.003838	0.505718	0.423	0.409	1	LC
USP15	0.005239	0.336445	0.254	0.198	1	LC
SOCS3	0.005484	0.405739	0.377	0.333	1	LC
DHRS7	0.006669	0.338873	0.415	0.427	1	LC
ZCCHC6	0.007467	0.355954	0.277	0.231	1	LC
BHLHE41	0.008211	0.38149	0.362	0.334	1	LC
PYCARD	0.008919	0.330756	0.5	0.544	1	LC
CEBPB	0.00938	0.77877	0.546	0.648	1	LC
DCT	0	4.497885	1	0.008	0	Mela
PMEL	0	3.925314	0.942	0.016	0	Mela
TYRP1	0	3.708586	0.962	0.006	0	Mela
MLANA	0	3.648404	1	0.009	0	Mela
GPX3	0	3.192207	0.981	0.021	0	Mela
CAPN3	0	2.902548	0.981	0.013	0	Mela

MITF	0	2.597103	0.942	0.019	0	Mela
BX647938	0	2.043014	0.75	0.004	0	Mela
DKFZp434C0631	0	1.702232	0.75	0.004	0	Mela
PLP1	0	1.621851	0.75	0.005	0	Mela
BCAN	0	1.610229	0.731	0.001	0	Mela
TYR	0	1.442724	0.615	0.001	0	Mela
PCSK2	0	1.378967	0.577	0.002	0	Mela
TRPM1	0	1.376101	0.635	0.001	0	Mela
LINC00462	0	1.357649	0.673	0.001	0	Mela
GAPDHS	0	1.270296	0.615	0.002	0	Mela
LOC100127888	0	1.25639	0.635	0.001	0	Mela
SOX10	0	0.986244	0.5	0	0	Mela
WDR63	0	0.843358	0.346	0.002	0	Mela
PAX3	0	0.804819	0.404	0	0	Mela
ROPN1	0	0.746952	0.365	0.004	0	Mela
AIF1L	0	0.722407	0.404	0.002	0	Mela
ALX1	0	0.668171	0.288	0	0	Mela
FABP7	0	0.655937	0.327	0.001	0	Mela
CLEC12B	0	0.628756	0.327	0	0	Mela
POSTN	0	0.611056	0.308	0.001	0	Mela
TRIM63	0	0.610156	0.308	0	0	Mela
SLC24A5	0	0.577065	0.308	0	0	Mela
SLC45A2	0	0.56384	0.288	0	0	Mela
C3orf79	0	0.556064	0.269	0.001	0	Mela
KU-MEL-3	0	0.511254	0.288	0	0	Mela
IDI2-AS1	4.76E-308	0.525249	0.269	0.002	8.66E-304	Mela
TUBB4A	2.28E-292	0.560604	0.288	0.002	4.16E-288	Mela
CA14	4.18E-289	0.760331	0.404	0.005	7.61E-285	Mela
S100B	6.30E-248	0.979779	0.365	0.005	1.15E-243	Mela
KCNAB2	4.65E-247	0.748671	0.385	0.005	8.47E-243	Mela
SNCA	3.76E-240	1.791489	0.846	0.032	6.85E-236	Mela
FGFBP2	4.92E-207	1.188093	0.385	0.007	8.96E-203	Mela
GPR143	3.41E-204	1.72534	0.769	0.031	6.21E-200	Mela
PTPLA	4.15E-198	0.651282	0.308	0.004	7.55E-194	Mela
APOE	3.90E-180	3.248988	0.923	0.054	7.09E-176	Mela
GCNT2	2.22E-166	1.083158	0.481	0.014	4.04E-162	Mela
GPM6B	3.14E-164	1.855595	0.885	0.054	5.71E-160	Mela
LINC00681	5.47E-161	1.05086	0.423	0.011	9.97E-157	Mela
MSC	6.10E-154	0.684891	0.288	0.005	1.11E-149	Mela
GYPC	8.27E-132	1.767811	0.846	0.062	1.51E-127	Mela
GMPR	3.94E-121	1.975518	0.865	0.071	7.18E-117	Mela
MCOLN3	4.66E-117	0.795085	0.385	0.013	8.49E-113	Mela
TBC1D16	1.67E-113	0.958844	0.404	0.015	3.03E-109	Mela
MIA	1.08E-108	0.504788	0.269	0.007	1.98E-104	Mela
ROPN1B	4.43E-107	0.803208	0.346	0.011	8.07E-103	Mela
EDNRB	3.21E-106	2.302705	0.981	0.112	5.85E-102	Mela
P2RX7	1.75E-101	0.671959	0.288	0.008	3.19E-97	Mela
TRIB2	6.07E-90	0.891073	0.481	0.027	1.11E-85	Mela

TMOD1	3.25E-85	0.507051	0.269	0.009	5.91E-81	Mela
QPCT	9.63E-84	2.822981	0.981	0.148	1.75E-79	Mela
CDC42EP3	5.96E-74	1.757835	0.904	0.137	1.08E-69	Mela
SLCO4A1	7.67E-72	0.788597	0.404	0.024	1.40E-67	Mela
AKAP12	1.25E-66	0.934762	0.538	0.046	2.28E-62	Mela
ENPP2	7.83E-63	0.869541	0.404	0.027	1.43E-58	Mela
STMN1	3.59E-62	1.983067	0.981	0.205	6.54E-58	Mela
AP1S2	5.83E-59	1.510021	0.75	0.112	1.06E-54	Mela
PHLDA1	2.14E-54	1.640152	0.942	0.205	3.90E-50	Mela
CHCHD6	1.33E-48	1.720097	0.808	0.17	2.42E-44	Mela
CYB561A3	7.87E-44	1.995967	0.846	0.22	1.43E-39	Mela
FMN1	1.09E-43	0.661443	0.308	0.023	1.98E-39	Mela
CAV1	1.65E-41	1.54441	0.923	0.263	3.00E-37	Mela
ZEB2	2.15E-38	0.721378	0.327	0.029	3.91E-34	Mela
MARCKSL1	1.19E-37	1.477914	0.865	0.248	2.17E-33	Mela
ITM2C	9.00E-37	0.674498	0.346	0.033	1.64E-32	Mela
SDCBP	1.70E-36	1.964614	1	0.564	3.09E-32	Mela
VIM	4.70E-36	1.796825	1	0.329	8.56E-32	Mela
C4orf48	1.19E-35	1.171414	0.692	0.154	2.18E-31	Mela
CYGB	2.75E-34	0.564225	0.269	0.022	5.00E-30	Mela
CD59	4.21E-32	1.864292	0.981	0.734	7.67E-28	Mela
DUSP4	4.38E-32	0.790376	0.423	0.057	7.98E-28	Mela
UBB	8.68E-32	1.471503	1	0.979	1.58E-27	Mela
TUBB2B	1.34E-31	0.951447	0.423	0.059	2.43E-27	Mela
CD44	1.85E-31	1.932622	0.962	0.583	3.38E-27	Mela
BACE2	2.38E-31	1.098987	0.596	0.125	4.33E-27	Mela
TFAP2A	4.36E-31	1.493326	0.885	0.385	7.94E-27	Mela
ST3GAL4	8.81E-31	1.293055	0.865	0.334	1.60E-26	Mela
PMP22	2.89E-30	1.081863	0.788	0.181	5.26E-26	Mela
RAB32	5.55E-30	1.222758	0.654	0.16	1.01E-25	Mela
SNHG7	1.65E-29	1.538661	0.942	0.598	3.00E-25	Mela
GPNMB	6.30E-29	1.806533	0.942	0.551	1.15E-24	Mela
FEZ1	7.21E-29	0.809345	0.365	0.047	1.31E-24	Mela
DLC1	1.51E-28	0.566262	0.308	0.034	2.76E-24	Mela
RPL13	4.42E-28	0.904969	1	0.999	8.04E-24	Mela
CDH3	7.53E-28	0.848754	0.385	0.055	1.37E-23	Mela
PLEKHA5	8.17E-28	1.416959	0.692	0.205	1.49E-23	Mela
ANXA5	2.27E-27	1.407987	0.923	0.415	4.14E-23	Mela
RPS2	4.31E-27	0.879992	1	0.997	7.84E-23	Mela
ARMCX1	5.75E-27	0.808603	0.442	0.075	1.05E-22	Mela
SGCE	1.57E-26	0.497514	0.269	0.028	2.85E-22	Mela
GNB2L1	4.20E-26	0.820604	1	0.99	7.64E-22	Mela
VAT1	9.02E-26	1.083396	0.673	0.205	1.64E-21	Mela
C17orf76-AS1	1.16E-25	1.038579	0.981	0.964	2.11E-21	Mela
CD63	1.29E-25	1.164526	1	0.98	2.35E-21	Mela
EEF1A1	7.90E-25	0.764884	1	1	1.44E-20	Mela
ST3GAL5	1.57E-24	0.692528	0.346	0.05	2.86E-20	Mela
RPS27A	1.74E-24	0.789334	1	0.997	3.18E-20	Mela

RPL13A	3.22E-24	0.686339	1	0.999	5.85E-20	Mela
PLOD3	6.97E-24	0.611181	0.365	0.057	1.27E-19	Mela
RPS6	7.23E-24	0.779525	1	0.999	1.32E-19	Mela
HSD17B11	1.06E-23	0.871777	0.519	0.115	1.93E-19	Mela
RPL3	3.67E-23	0.595082	1	0.999	6.69E-19	Mela
CYP27A1	4.32E-23	0.833123	0.442	0.086	7.87E-19	Mela
LOC729732	5.32E-23	0.9983	0.538	0.132	9.68E-19	Mela
KLF6	8.84E-23	1.868351	0.904	0.676	1.61E-18	Mela
HNMT	1.84E-22	0.466476	0.308	0.042	3.35E-18	Mela
RPS19	5.40E-22	0.804156	1	0.998	9.83E-18	Mela
RGS3	7.51E-22	0.779868	0.481	0.108	1.37E-17	Mela
RPL17	1.52E-21	0.846308	1	0.985	2.77E-17	Mela
EFHD1	2.49E-21	0.671471	0.519	0.113	4.54E-17	Mela
RPL6	2.62E-21	0.671177	1	0.992	4.77E-17	Mela
CYTH3	2.63E-21	0.612061	0.327	0.051	4.78E-17	Mela
RPL19	2.83E-21	0.591292	1	0.999	5.15E-17	Mela
RPL10	3.39E-21	0.619119	1	1	6.17E-17	Mela
FDFT1	2.18E-20	1.104135	0.827	0.481	3.97E-16	Mela
SEPT4	2.39E-20	0.506756	0.308	0.046	4.36E-16	Mela
RPS7	2.51E-20	0.669983	1	0.993	4.57E-16	Mela
ANXA6	3.00E-20	0.542452	0.308	0.046	5.47E-16	Mela
RPS18	4.12E-20	0.609875	1	1	7.49E-16	Mela
CLCN7	4.91E-20	0.625216	0.327	0.054	8.94E-16	Mela
RPS5	7.93E-20	0.699324	1	0.99	1.44E-15	Mela
RPL37A	1.15E-19	0.589973	1	0.998	2.09E-15	Mela
PYURF	1.26E-19	1.240681	0.885	0.687	2.29E-15	Mela
PIP4K2A	1.57E-19	0.562623	0.269	0.038	2.86E-15	Mela
CCDC61	1.92E-19	0.642055	0.327	0.055	3.49E-15	Mela
ST6GALNAC2	1.93E-19	1.107689	0.538	0.155	3.52E-15	Mela
RPL32	2.10E-19	0.608579	1	0.999	3.81E-15	Mela
ID2	2.20E-19	1.355503	0.904	0.559	4.01E-15	Mela
RPL7A	3.99E-19	0.654511	1	0.987	7.26E-15	Mela
LGALS1	6.70E-19	0.651405	0.904	0.326	1.22E-14	Mela
RPL29	8.30E-19	0.65385	0.981	0.993	1.51E-14	Mela
NPM1	8.46E-19	0.794059	0.981	0.928	1.54E-14	Mela
ILVBL	1.05E-18	1.22137	0.692	0.3	1.92E-14	Mela
RPS10	1.50E-18	0.790088	1	0.978	2.73E-14	Mela
DSTYK	2.01E-18	0.700793	0.346	0.064	3.67E-14	Mela
RPL18A	2.04E-18	0.527755	1	0.997	3.71E-14	Mela
TFAP2B	2.14E-18	0.972014	0.5	0.13	3.90E-14	Mela
RPS28	2.65E-18	0.523103	1	0.999	4.82E-14	Mela
A1BG	3.81E-18	0.683501	0.385	0.078	6.94E-14	Mela
RPL5	1.16E-17	0.655438	1	0.993	2.12E-13	Mela
LMNA	1.18E-17	0.99249	1	0.967	2.14E-13	Mela
RPL36A	1.24E-17	0.736082	1	0.989	2.26E-13	Mela
RPS3	1.39E-17	0.682495	1	0.996	2.53E-13	Mela
RPS3A	1.52E-17	0.603198	1	0.998	2.77E-13	Mela
JUN	1.54E-17	1.032101	1	0.979	2.80E-13	Mela

CHPT1	2.12E-17	0.619428	0.385	0.081	3.86E-13	Mela
RPL26	2.28E-17	0.570487	1	0.999	4.15E-13	Mela
RPL34	2.34E-17	0.549759	1	1	4.27E-13	Mela
RPL10A	2.77E-17	0.624758	1	0.995	5.05E-13	Mela
RPL18	5.53E-17	0.619934	1	0.996	1.01E-12	Mela
PNMA1	7.81E-17	0.554203	0.365	0.077	1.42E-12	Mela
RPS15	1.31E-16	0.455596	1	1	2.39E-12	Mela
RPS14	1.35E-16	0.540952	1	1	2.46E-12	Mela
GAS5	1.62E-16	0.832326	1	0.934	2.95E-12	Mela
RPL14	1.93E-16	0.534402	1	0.995	3.52E-12	Mela
PAG1	2.13E-16	0.522275	0.288	0.05	3.88E-12	Mela
METTL9	2.48E-16	1.302274	0.788	0.553	4.52E-12	Mela
BTG1	2.59E-16	0.779708	1	0.953	4.71E-12	Mela
RPL12	2.71E-16	0.614812	1	0.998	4.93E-12	Mela
RPL41	3.03E-16	0.448322	1	1	5.52E-12	Mela
RPS9	4.45E-16	0.562422	1	0.998	8.11E-12	Mela
TOMM7	4.92E-16	0.592908	1	0.969	8.96E-12	Mela
GNPTAB	5.16E-16	0.606276	0.308	0.058	9.40E-12	Mela
CTH	6.11E-16	0.643668	0.269	0.045	1.11E-11	Mela
SNAI2	1.34E-15	1.062885	0.712	0.361	2.44E-11	Mela
RPS4X	1.44E-15	0.530735	1	0.999	2.62E-11	Mela
HSPA1B	2.60E-15	1.454713	0.846	0.712	4.73E-11	Mela
RPL27A	2.99E-15	0.489618	1	0.998	5.45E-11	Mela
EMP3	4.17E-15	0.891255	0.692	0.279	7.60E-11	Mela
CCNI	6.23E-15	0.915841	0.846	0.54	1.13E-10	Mela
EEF1G	6.73E-15	0.610547	0.981	0.988	1.23E-10	Mela
RPLP0	7.19E-15	0.614044	1	0.992	1.31E-10	Mela
RPL8	8.45E-15	0.512987	1	0.998	1.54E-10	Mela
MLPH	9.77E-15	0.896129	0.577	0.23	1.78E-10	Mela
RPL35A	1.31E-14	0.412185	1	0.998	2.38E-10	Mela
RPS8	1.51E-14	0.55951	1	0.999	2.75E-10	Mela
C9orf3	2.18E-14	1.006781	0.654	0.324	3.97E-10	Mela
TUBA1A	4.24E-14	0.927278	0.923	0.709	7.71E-10	Mela
AP3M2	4.55E-14	0.586034	0.308	0.065	8.29E-10	Mela
NSG1	4.69E-14	0.975374	0.731	0.453	8.54E-10	Mela
TGFB1I1	4.91E-14	0.548895	0.269	0.05	8.94E-10	Mela
RPL31	5.72E-14	0.446113	1	0.998	1.04E-09	Mela
SOX4	9.08E-14	1.024481	0.692	0.381	1.65E-09	Mela
SLC25A6	1.13E-13	0.760323	0.923	0.897	2.06E-09	Mela
C1orf85	1.17E-13	0.621598	0.385	0.105	2.13E-09	Mela
PTTG1IP	1.55E-13	1.250934	0.788	0.586	2.83E-09	Mela
RPS16	1.69E-13	0.474835	1	0.994	3.08E-09	Mela
MYC	1.84E-13	1.005725	0.846	0.566	3.35E-09	Mela
DDIT4	2.44E-13	1.063466	0.75	0.463	4.43E-09	Mela
RPL11	2.98E-13	0.410549	1	0.999	5.43E-09	Mela
GLTSCR2	3.05E-13	0.776755	0.904	0.828	5.55E-09	Mela
RPS17L	3.57E-13	0.450554	1	0.996	6.50E-09	Mela
FAM213A	4.22E-13	0.837765	0.692	0.344	7.69E-09	Mela

RPS11	4.79E-13	0.503926	0.981	0.985	8.73E-09	Mela
RPL35	4.92E-13	0.450408	1	0.999	8.96E-09	Mela
MYO10	6.52E-13	0.914201	0.731	0.491	1.19E-08	Mela
MAGEH1	7.52E-13	0.614062	0.423	0.129	1.37E-08	Mela
RPL39	1.05E-12	0.421884	1	0.999	1.91E-08	Mela
RPS13	1.09E-12	0.433576	1	0.998	1.98E-08	Mela
TM7SF3	1.24E-12	0.856798	0.596	0.287	2.25E-08	Mela
EIF4A2	1.81E-12	0.794682	0.942	0.903	3.30E-08	Mela
CEP72	2.13E-12	0.739648	0.346	0.092	3.88E-08	Mela
RPL15	2.29E-12	0.384607	1	0.999	4.16E-08	Mela
EEF2	4.02E-12	1.120132	0.846	0.874	7.33E-08	Mela
C6orf1	5.14E-12	0.814662	0.462	0.166	9.36E-08	Mela
GPR155	5.39E-12	0.554337	0.308	0.075	9.81E-08	Mela
SLC25A5	5.98E-12	0.744452	0.942	0.874	1.09E-07	Mela
SIGIRR	7.01E-12	0.764	0.596	0.294	1.28E-07	Mela
PFKM	9.53E-12	0.629943	0.423	0.141	1.74E-07	Mela
C1orf63	9.70E-12	0.831788	0.75	0.513	1.77E-07	Mela
EEF1D	9.88E-12	0.479357	1	0.978	1.80E-07	Mela
ENSG0000021008:	1.01E-11	0.627623	1	1	1.85E-07	Mela
SERPINF1	1.01E-11	0.889746	0.827	0.525	1.85E-07	Mela
COX4I1	1.07E-11	0.423634	1	0.978	1.95E-07	Mela
RPS27	1.29E-11	0.373358	1	1	2.34E-07	Mela
MBP	1.47E-11	0.799786	0.481	0.192	2.69E-07	Mela
NACA	1.57E-11	0.323988	1	0.996	2.86E-07	Mela
CDK2	1.57E-11	0.800617	0.365	0.109	2.87E-07	Mela
PTEN	1.78E-11	0.631776	0.365	0.109	3.24E-07	Mela
ILK	2.06E-11	0.802511	0.615	0.341	3.74E-07	Mela
MLEC	2.34E-11	1.039122	0.538	0.257	4.26E-07	Mela
RPL7	2.42E-11	0.385623	1	0.999	4.41E-07	Mela
RBM3	2.85E-11	0.612475	0.962	0.916	5.20E-07	Mela
CERS4	3.36E-11	0.55491	0.308	0.079	6.11E-07	Mela
HSPA2	3.87E-11	0.907458	0.692	0.408	7.05E-07	Mela
CTSF	4.03E-11	0.745738	0.712	0.383	7.33E-07	Mela
UBL3	4.48E-11	0.95381	0.596	0.305	8.16E-07	Mela
RHOB	4.65E-11	1.171208	0.827	0.775	8.47E-07	Mela
PTMA	5.73E-11	0.52288	1	0.996	1.04E-06	Mela
PFDN5	5.89E-11	0.477412	0.962	0.974	1.07E-06	Mela
ADIPOR2	7.18E-11	0.512506	0.327	0.092	1.31E-06	Mela
FBXO32	7.64E-11	0.750189	0.519	0.232	1.39E-06	Mela
TNFRSF14	7.97E-11	0.680079	0.327	0.092	1.45E-06	Mela
PNRC1	8.11E-11	0.638864	0.942	0.868	1.48E-06	Mela
KIT	9.45E-11	0.511328	0.327	0.09	1.72E-06	Mela
RPSA	9.50E-11	0.639843	0.942	0.87	1.73E-06	Mela
ENSG0000021007	9.61E-11	0.67855	0.308	0.081	1.75E-06	Mela
TSPAN4	9.71E-11	0.691686	0.558	0.254	1.77E-06	Mela
AF063596	1.14E-10	0.823021	0.577	0.289	2.07E-06	Mela
FTL	1.57E-10	0.384012	1	0.996	2.86E-06	Mela
HLA-E	1.77E-10	0.542508	0.904	0.885	3.22E-06	Mela

ENSG0000021145!	1.82E-10	0.561013	1	0.997	3.31E-06	Mela
EEF1B2	1.86E-10	0.607237	0.923	0.903	3.38E-06	Mela
NPC2	1.98E-10	0.557698	0.923	0.869	3.61E-06	Mela
EIF3D	2.51E-10	0.724099	0.788	0.66	4.57E-06	Mela
RPL4	2.66E-10	0.430699	1	0.986	4.85E-06	Mela
TMEM258	3.31E-10	0.550735	0.942	0.924	6.02E-06	Mela
RPS12	3.88E-10	0.375382	1	0.999	7.07E-06	Mela
RPLP2	4.10E-10	0.428074	1	0.999	7.46E-06	Mela
STXBP6	4.22E-10	0.466914	0.288	0.075	7.69E-06	Mela
RPL23A	4.24E-10	0.358885	1	0.999	7.72E-06	Mela
HSP90AB1	4.44E-10	0.790303	0.981	0.963	8.09E-06	Mela
H2AFJ	5.85E-10	0.838617	0.75	0.554	1.07E-05	Mela
HPGD	6.89E-10	0.885624	0.442	0.169	1.26E-05	Mela
ASAH1	6.96E-10	0.876031	0.788	0.673	1.27E-05	Mela
RPL38	7.35E-10	0.421308	1	0.989	1.34E-05	Mela
RPS15A	7.55E-10	0.398822	1	0.997	1.37E-05	Mela
MT1F	7.59E-10	0.840633	0.365	0.123	1.38E-05	Mela
CYSTM1	7.65E-10	0.808206	0.673	0.464	1.39E-05	Mela
MSS51	8.21E-10	0.511043	1	0.979	1.50E-05	Mela
RPL36AL	9.86E-10	0.529476	0.942	0.955	1.79E-05	Mela
RPL24	1.22E-09	0.421782	1	0.994	2.23E-05	Mela
ATP1A1	1.47E-09	0.728638	0.75	0.589	2.68E-05	Mela
EID1	1.73E-09	0.514543	0.885	0.884	3.15E-05	Mela
RPS25	1.73E-09	0.304784	1	0.999	3.16E-05	Mela
PPP1R15A	1.82E-09	0.606834	0.962	0.917	3.31E-05	Mela
TPPP3	1.95E-09	0.934637	0.288	0.077	3.55E-05	Mela
CD99	2.12E-09	0.71449	0.904	0.899	3.86E-05	Mela
SLC3A2	2.61E-09	0.686331	0.865	0.788	4.76E-05	Mela
RPS20	2.99E-09	0.33752	1	0.997	5.45E-05	Mela
ENSG0000019876:	3.77E-09	0.549	0.981	0.984	6.87E-05	Mela
RPS23	4.22E-09	0.336095	1	0.998	7.68E-05	Mela
RPL37	5.53E-09	0.376331	1	0.995	0.000101	Mela
EIF3L	7.82E-09	0.662083	0.865	0.818	0.000142	Mela
PKIG	8.61E-09	0.660932	0.423	0.176	0.000157	Mela
HSP90AA1	8.63E-09	0.60541	0.981	0.975	0.000157	Mela
CCDC80	1.11E-08	0.609498	0.365	0.128	0.000203	Mela
SNW1	1.30E-08	0.604991	0.731	0.588	0.000236	Mela
EIF3E	1.89E-08	0.636295	0.865	0.822	0.000343	Mela
CTSA	2.00E-08	0.754076	0.596	0.391	0.000365	Mela
ACTG1	2.27E-08	0.470475	1	0.995	0.000413	Mela
CELF2	3.00E-08	0.586237	0.423	0.184	0.000546	Mela
VAMP5	3.55E-08	0.545019	0.365	0.133	0.000647	Mela
FAM195B	4.16E-08	0.577052	0.385	0.155	0.000758	Mela
NSMCE1	4.17E-08	0.704132	0.442	0.212	0.000758	Mela
CYB5R3	4.33E-08	0.667357	0.615	0.423	0.000788	Mela
GPR56	4.46E-08	0.874347	0.442	0.212	0.000812	Mela
TMEM55A	5.76E-08	0.516254	0.365	0.137	0.001049	Mela
TPP1	5.83E-08	0.586981	0.346	0.129	0.001062	Mela

SNHG16	5.87E-08	1.007911	0.654	0.512	0.001068	Mela
CSRP1	5.87E-08	0.679839	0.635	0.461	0.00107	Mela
RPL28	7.04E-08	0.317886	1	0.992	0.001281	Mela
RAB7A	7.19E-08	0.570479	0.904	0.88	0.001309	Mela
RSL24D1	8.68E-08	0.618402	0.769	0.668	0.00158	Mela
HNRNPA0	1.11E-07	0.489473	0.904	0.874	0.002018	Mela
PTRF	1.22E-07	0.742544	0.673	0.455	0.002226	Mela
CPQ	1.65E-07	0.611646	0.385	0.146	0.003003	Mela
ZNF106	1.91E-07	0.603351	0.327	0.123	0.003481	Mela
RPS14P3	2.07E-07	0.497771	0.904	0.962	0.003771	Mela
GNG11	2.22E-07	0.487964	0.481	0.202	0.004044	Mela
PRELP	2.78E-07	0.408667	0.385	0.137	0.005063	Mela
RPL22	3.41E-07	0.382083	1	0.994	0.006213	Mela
CMTM3	3.50E-07	0.486027	0.269	0.088	0.00638	Mela
UBXN1	3.79E-07	0.691274	0.692	0.649	0.006902	Mela
MAP3K11	3.87E-07	0.342708	0.269	0.087	0.007046	Mela
KMT2E	4.18E-07	0.663344	0.692	0.588	0.007617	Mela
SLC44A1	4.79E-07	0.509299	0.308	0.111	0.008727	Mela
HIST1H4C	5.46E-07	0.749367	0.5	0.291	0.009946	Mela
ATP5G2	5.78E-07	0.394658	0.923	0.911	0.01052	Mela
STX7	5.79E-07	0.714708	0.538	0.343	0.010543	Mela
RPL27	7.16E-07	0.289834	1	0.994	0.013043	Mela
SNHG6	7.19E-07	0.648929	0.769	0.76	0.013094	Mela
HIBCH	7.25E-07	0.705917	0.519	0.34	0.0132	Mela
ZFAND5	8.32E-07	0.539675	0.788	0.664	0.015153	Mela
MSN	9.34E-07	0.537382	0.288	0.102	0.017015	Mela
HSPA8	9.59E-07	0.664389	0.827	0.792	0.017456	Mela
MYADM	9.83E-07	0.812642	0.481	0.269	0.017899	Mela
DHRSX	1.36E-06	0.448334	0.269	0.095	0.024845	Mela
GABARAP	1.37E-06	0.419832	0.942	0.954	0.024911	Mela
C12orf57	1.46E-06	0.487973	0.75	0.742	0.026523	Mela
NAP1L1	1.74E-06	0.584367	0.769	0.736	0.031677	Mela
TRIM47	1.81E-06	0.445421	0.308	0.119	0.033022	Mela
TMEM147	1.93E-06	0.577105	0.712	0.637	0.035073	Mela
HSPA1A	2.86E-06	1.085733	0.365	0.174	0.052038	Mela
EIF3G	2.96E-06	0.54803	0.673	0.65	0.053852	Mela
ZFAS1	3.11E-06	0.373156	0.865	0.867	0.056615	Mela
HSPD1	3.43E-06	0.708702	0.731	0.69	0.06239	Mela
ENSG0000021019	3.44E-06	0.896224	0.462	0.273	0.062668	Mela
HYI	3.52E-06	0.429395	0.346	0.156	0.064124	Mela
ABHD14B	3.75E-06	0.52759	0.462	0.27	0.068301	Mela
IRS2	4.10E-06	0.502899	0.404	0.193	0.074573	Mela
RNASEK-C17orf49	5.40E-06	0.457354	0.865	0.929	0.098302	Mela
PABPC1	5.52E-06	0.533695	0.885	0.902	0.10046	Mela
ARMCX3	5.57E-06	0.488095	0.288	0.114	0.101352	Mela
SRSF8	5.58E-06	0.509855	0.558	0.414	0.101668	Mela
HPS1	6.30E-06	0.43207	0.308	0.128	0.114778	Mela
MXI1	6.31E-06	0.797584	0.404	0.218	0.114838	Mela

OPTN	8.64E-06	0.664907	0.5	0.343	0.157286	Mela
B2M	8.73E-06	0.285841	1	0.997	0.159025	Mela
VKORC1	8.85E-06	0.523991	0.558	0.411	0.161201	Mela
AGPAT2	9.36E-06	0.615796	0.673	0.591	0.170423	Mela
ENSG0000019884(	1.06E-05	0.416298	0.942	0.883	0.193801	Mela
GNG5	1.07E-05	0.388923	0.808	0.794	0.194513	Mela
DHRS7	1.11E-05	0.686414	0.558	0.426	0.202392	Mela
TCEAL8	1.12E-05	0.569407	0.615	0.538	0.203953	Mela
LOC100506421	1.15E-05	0.396677	0.346	0.137	0.209	Mela
CRYL1	1.18E-05	0.726901	0.423	0.252	0.21568	Mela
C2orf69	1.28E-05	0.491827	0.269	0.106	0.232858	Mela
FCGRT	1.38E-05	0.562981	0.481	0.31	0.250488	Mela
AK291701	1.53E-05	0.427778	0.788	0.758	0.279154	Mela
RHOA	1.54E-05	0.405278	0.808	0.875	0.280807	Mela
OSBPL9	1.60E-05	0.499512	0.385	0.205	0.291627	Mela
ENSG0000019888	1.63E-05	0.361853	0.923	0.96	0.295944	Mela
TIMP2	1.74E-05	0.254718	0.365	0.146	0.317063	Mela
CTSL1	1.88E-05	0.864509	0.577	0.495	0.341631	Mela
SLC25A3	2.05E-05	0.422432	0.865	0.911	0.374165	Mela
IRF2BP2	2.29E-05	0.649537	0.519	0.353	0.417105	Mela
VMP1	2.31E-05	0.639649	0.519	0.383	0.421398	Mela
SGK1	2.43E-05	1.029849	0.692	0.711	0.441695	Mela
CALD1	2.52E-05	0.36964	0.519	0.289	0.458508	Mela
RPS29	2.57E-05	0.265331	1	0.996	0.468197	Mela
UBA2	2.78E-05	0.707349	0.442	0.276	0.506681	Mela
EPB41L4A-AS1	2.80E-05	0.536413	0.615	0.525	0.509315	Mela
LRPAP1	2.82E-05	0.4407	0.731	0.686	0.512564	Mela
CISD1	2.88E-05	0.67812	0.519	0.386	0.525041	Mela
HPCAL1	2.94E-05	0.439777	0.308	0.14	0.535639	Mela
HLA-A	3.05E-05	0.699977	0.923	0.961	0.555761	Mela
PHGDH	3.46E-05	0.744048	0.519	0.387	0.629468	Mela
RHOBTB3	3.86E-05	0.571933	0.385	0.21	0.701921	Mela
NEU1	4.11E-05	0.62794	0.442	0.292	0.748196	Mela
PBRM1	4.80E-05	0.549686	0.346	0.181	0.873272	Mela
MZT2B	4.84E-05	0.431089	0.731	0.702	0.880692	Mela
IDI1	4.87E-05	0.655539	0.577	0.476	0.88587	Mela
MMP24-AS1	5.63E-05	0.513621	0.404	0.24	1	Mela
CPEB4	6.40E-05	0.480045	0.308	0.145	1	Mela
EIF1	6.54E-05	0.257134	1	1	1	Mela
SIAH1	7.90E-05	0.471872	0.308	0.152	1	Mela
HPS4	8.85E-05	0.429357	0.269	0.12	1	Mela
ARPC5	9.19E-05	0.482181	0.519	0.355	1	Mela
FOS	0.000102	0.397861	0.981	0.974	1	Mela
RPS27L	0.000104	0.376679	0.788	0.846	1	Mela
YBX1	0.000111	0.314362	0.981	0.982	1	Mela
CIRBP	0.000111	0.403184	0.923	0.981	1	Mela
SEPT9	0.000113	0.434036	0.327	0.17	1	Mela
EIF3K	0.000117	0.39334	0.865	0.897	1	Mela

ARL2	0.000128	0.454287	0.558	0.463	1	Mela
ARL6IP1	0.000142	0.403468	0.75	0.779	1	Mela
BAMBI	0.000161	0.552992	0.327	0.172	1	Mela
H2AFZ	0.00017	0.478788	0.865	0.896	1	Mela
PARVA	0.000177	0.556657	0.327	0.174	1	Mela
ZNF503	0.000192	0.420952	0.365	0.205	1	Mela
PEPD	0.000194	0.512111	0.462	0.334	1	Mela
S100A13	0.0002	0.374141	0.673	0.616	1	Mela
FBXO7	0.000228	0.647293	0.519	0.436	1	Mela
FASTK	0.000248	0.596305	0.404	0.265	1	Mela
TNIP1	0.000253	0.428616	0.385	0.244	1	Mela
TAF1D	0.00026	0.53743	0.462	0.339	1	Mela
CRNDE	0.00026	0.540548	0.5	0.355	1	Mela
GSTO1	0.000267	0.373311	0.769	0.77	1	Mela
COMT	0.000282	0.433162	0.846	0.863	1	Mela
FSCN1	0.000289	0.481476	0.404	0.246	1	Mela
SEC22C	0.000296	0.404752	0.308	0.164	1	Mela
TMSB10	0.00032	0.34175	0.981	0.997	1	Mela
NAMPT	0.000325	0.532232	0.5	0.394	1	Mela
TRA2B	0.000328	0.465371	0.75	0.795	1	Mela
CLTA	0.000328	0.382368	0.808	0.853	1	Mela
PLTP	0.000352	0.414817	0.269	0.128	1	Mela
ZFP36L2	0.000355	0.596506	0.596	0.522	1	Mela
MYLIP	0.000355	0.668438	0.462	0.352	1	Mela
ESD	0.000364	0.560263	0.577	0.534	1	Mela
NTAN1	0.000375	0.640932	0.385	0.249	1	Mela
FBXW5	0.000402	0.626651	0.404	0.276	1	Mela
CLEC2D	0.000427	0.423283	0.269	0.132	1	Mela
RPL9	0.000457	0.381761	0.788	0.787	1	Mela
CRTAP	0.000479	0.476614	0.404	0.27	1	Mela
CYB5R1	0.000494	0.462734	0.442	0.324	1	Mela
CRIP2	0.000508	0.32862	0.385	0.219	1	Mela
IFI16	0.000569	0.522379	0.558	0.488	1	Mela
MTDH	0.000599	0.48349	0.615	0.574	1	Mela
GSTM3	0.000617	0.525672	0.385	0.231	1	Mela
HIST2H2BE	0.000621	0.529461	0.404	0.259	1	Mela
SYNGR1	0.000643	0.609497	0.385	0.263	1	Mela
IMPDH2	0.000671	0.468216	0.519	0.466	1	Mela
TROVE2	0.000679	0.405491	0.423	0.303	1	Mela
CCT4	0.000679	0.476553	0.596	0.577	1	Mela
SLC35B2	0.000687	0.495146	0.365	0.239	1	Mela
FSTL1	0.000701	0.510686	0.269	0.132	1	Mela
CTSD	0.000743	0.353636	0.846	0.899	1	Mela
L32131	0.000748	0.548532	0.712	0.745	1	Mela
PRKCDBP	0.000758	0.396079	0.404	0.26	1	Mela
EIF1B	0.000801	0.40976	0.673	0.714	1	Mela
MAGED2	0.000833	0.601681	0.5	0.423	1	Mela
TTC3	0.000884	0.550688	0.404	0.281	1	Mela

HIST1H2BK	0.000899	0.602411	0.327	0.184	1	Mela
WBP5	0.001206	0.475714	0.577	0.482	1	Mela
SNHG5	0.001228	0.304648	0.865	0.919	1	Mela
MAD2L1BP	0.001273	0.489702	0.346	0.226	1	Mela
CCNDBP1	0.001275	0.466595	0.481	0.401	1	Mela
NR4A2	0.001297	0.584114	0.327	0.201	1	Mela
C6orf48	0.00132	0.397946	0.654	0.651	1	Mela
CCND3	0.001396	0.607833	0.442	0.365	1	Mela
CCNG1	0.001498	0.478427	0.442	0.339	1	Mela
HMG20B	0.001622	0.548377	0.442	0.361	1	Mela
RETSAT	0.001859	0.494839	0.288	0.167	1	Mela
PIR	0.001881	0.386179	0.615	0.531	1	Mela
PABPC4	0.001978	0.485249	0.327	0.208	1	Mela
RPS26	0.002005	0.318101	0.885	0.943	1	Mela
SLC7A5P2	0.002026	0.685067	0.404	0.311	1	Mela
RRAGC	0.002152	0.374445	0.365	0.243	1	Mela
SLC25A36	0.002168	0.517288	0.385	0.283	1	Mela
RNF114	0.002295	0.489606	0.558	0.556	1	Mela
ACYP1	0.002396	0.378089	0.442	0.351	1	Mela
BSG	0.002414	0.343956	0.75	0.85	1	Mela
ATP1B1	0.002708	0.535407	0.346	0.231	1	Mela
EIF3H	0.00278	0.36062	0.788	0.847	1	Mela
ST13	0.002938	0.340514	0.788	0.852	1	Mela
SSR2	0.003033	0.437676	0.654	0.693	1	Mela
ZFP36L1	0.003044	0.424928	0.788	0.848	1	Mela
EIF3F	0.003265	0.428242	0.712	0.773	1	Mela
RAB38	0.003286	0.577064	0.404	0.319	1	Mela
ABTB1	0.003298	0.340383	0.269	0.155	1	Mela
OXA1L	0.00333	0.430222	0.5	0.451	1	Mela
MAD2L2	0.003455	0.384067	0.269	0.157	1	Mela
TMED10	0.003502	0.425294	0.75	0.816	1	Mela
PCBP2	0.003615	0.340432	0.731	0.816	1	Mela
NARF	0.003707	0.390399	0.327	0.221	1	Mela
RWDD1	0.003862	0.366235	0.673	0.737	1	Mela
PRMT2	0.003927	0.473669	0.442	0.374	1	Mela
LETMD1	0.004302	0.360865	0.346	0.237	1	Mela
LAMP2	0.004633	0.462079	0.615	0.673	1	Mela
CCDC107	0.005413	0.459298	0.269	0.161	1	Mela
SAT2	0.00574	0.50728	0.481	0.452	1	Mela
DPP7	0.005751	0.523457	0.519	0.476	1	Mela
NR3C1	0.005885	0.449475	0.269	0.164	1	Mela
AMD1	0.005925	0.373534	0.5	0.432	1	Mela
TLE4	0.005937	0.470288	0.327	0.224	1	Mela
SYF2	0.006145	0.41999	0.673	0.775	1	Mela
FOSB	0.006291	0.296021	0.827	0.831	1	Mela
LITAF	0.007203	0.42283	0.558	0.568	1	Mela
RABAC1	0.007217	0.312426	0.692	0.757	1	Mela
SUCLG1	0.007439	0.390221	0.538	0.545	1	Mela

NONO	0.008574	0.448167	0.519	0.518	1	Mela
TBCC	0.008726	0.560479	0.385	0.317	1	Mela
P4HB	0.008829	0.415105	0.577	0.619	1	Mela
SWAP70	0.009056	0.449995	0.385	0.309	1	Mela
GRSF1	0.009331	0.499645	0.404	0.349	1	Mela
EMP1	0.009954	0.420024	0.923	0.925	1	Mela
CLDN5	0	2.342112	0.344	0.001	0	VEC
C7	0	1.519548	0.375	0	0	VEC
CXCL12	0	1.052164	0.281	0	0	VEC
ECSCR	0	0.958011	0.375	0	0	VEC
LOC100505495	0	0.914476	0.312	0.001	0	VEC
GUCY1A3	0	0.732555	0.281	0.001	0	VEC
COL4A2	0	0.463422	0.312	0	0	VEC
COL4A1	0	0.458767	0.281	0	0	VEC
PECAM1	5.80E-256	0.701854	0.312	0.002	1.06E-251	VEC
RAMP2	2.29E-220	1.419387	0.625	0.011	4.18E-216	VEC
A2M	2.02E-192	0.725153	0.312	0.003	3.68E-188	VEC
TSPAN7	2.97E-168	0.98167	0.375	0.005	5.40E-164	VEC
RARRES2	2.96E-103	1.119344	0.312	0.006	5.39E-99	VEC
IL6	2.39E-93	0.88354	0.344	0.008	4.35E-89	VEC
ITM2A	5.14E-91	1.373683	0.531	0.02	9.36E-87	VEC
MFRP	2.33E-81	0.551276	0.344	0.009	4.24E-77	VEC
CPE	3.68E-68	1.681015	0.594	0.035	6.70E-64	VEC
DPT	3.30E-60	0.876632	0.281	0.009	6.01E-56	VEC
MEF2C	1.22E-58	0.596738	0.281	0.009	2.21E-54	VEC
CRISPLD2	7.92E-49	0.789043	0.375	0.019	1.44E-44	VEC
TFPI	2.81E-45	1.717443	0.688	0.072	5.12E-41	VEC
CTHRC1	8.12E-44	0.685151	0.344	0.018	1.48E-39	VEC
GYPC	3.37E-41	0.994915	0.625	0.063	6.14E-37	VEC
SRPX	1.18E-38	0.747991	0.312	0.017	2.14E-34	VEC
ABCA9	5.10E-38	0.704881	0.312	0.017	9.29E-34	VEC
C10orf10	1.52E-36	1.350345	0.562	0.059	2.76E-32	VEC
COL3A1	5.65E-36	1.020073	0.312	0.018	1.03E-31	VEC
MGP	2.20E-34	2.038732	0.844	0.142	4.01E-30	VEC
MEST	1.14E-29	0.963643	0.375	0.031	2.08E-25	VEC
LEPR	1.85E-29	1.074083	0.406	0.037	3.37E-25	VEC
NNMT	9.73E-29	1.557339	0.875	0.17	1.77E-24	VEC
ABCA8	1.95E-28	0.865905	0.281	0.018	3.56E-24	VEC
NKD2	2.59E-28	0.603177	0.344	0.027	4.71E-24	VEC
SOCS2	7.38E-28	0.75074	0.344	0.028	1.34E-23	VEC
MEG3	1.08E-27	0.813238	0.281	0.019	1.96E-23	VEC
PMP22	5.27E-26	1.326823	0.875	0.182	9.60E-22	VEC
PDK4	6.22E-26	0.879618	0.5	0.062	1.13E-21	VEC
LAMA4	2.65E-25	0.432784	0.281	0.02	4.83E-21	VEC
FAM105A	3.56E-25	0.997359	0.531	0.074	6.48E-21	VEC
GREM2	5.63E-25	0.677514	0.312	0.026	1.02E-20	VEC
C1R	2.32E-24	1.911892	0.781	0.179	4.22E-20	VEC
IFITM2	3.18E-24	1.190793	0.844	0.186	5.78E-20	VEC

TCF4	7.95E-24	1.474401	0.719	0.144	1.45E-19	VEC
IGFBP5	1.20E-23	1.77725	0.406	0.046	2.19E-19	VEC
EMCN	2.01E-23	0.875447	0.469	0.061	3.67E-19	VEC
PDGFRA	3.00E-23	0.686451	0.406	0.047	5.46E-19	VEC
SPRY1	5.57E-23	1.63029	0.719	0.17	1.01E-18	VEC
ARHGAP29	1.99E-22	1.264899	0.625	0.12	3.62E-18	VEC
AQP1	3.07E-22	1.23604	0.844	0.183	5.59E-18	VEC
VIM	4.67E-22	1.647404	1	0.33	8.50E-18	VEC
IFITM1	5.68E-22	1.995206	0.906	0.329	1.03E-17	VEC
SERPING1	4.50E-20	1.515313	0.875	0.238	8.19E-16	VEC
TPM2	4.60E-20	0.661083	0.438	0.06	8.38E-16	VEC
GEM	5.12E-20	1.312309	0.406	0.055	9.33E-16	VEC
GGT5	1.24E-19	1.251697	0.5	0.083	2.26E-15	VEC
GPM6B	1.56E-19	0.832668	0.406	0.056	2.84E-15	VEC
SLC2A3	9.81E-19	0.973552	0.562	0.107	1.79E-14	VEC
IFITM3	1.31E-18	1.537046	1	0.587	2.38E-14	VEC
COL18A1	1.39E-18	0.594417	0.469	0.074	2.53E-14	VEC
GNG11	2.51E-18	1.749652	0.75	0.202	4.58E-14	VEC
ENG	3.38E-18	0.538505	0.344	0.041	6.15E-14	VEC
PRELP	4.49E-18	1.538584	0.625	0.137	8.17E-14	VEC
MFAP4	8.26E-18	1.519958	0.531	0.103	1.50E-13	VEC
F2R	8.43E-18	1.190254	0.469	0.08	1.53E-13	VEC
DAB2	1.84E-17	0.841988	0.594	0.118	3.35E-13	VEC
IGFBP4	2.06E-17	1.218161	0.719	0.203	3.76E-13	VEC
PPAP2B	2.10E-17	1.166543	0.75	0.2	3.83E-13	VEC
C1S	2.15E-17	1.825482	0.656	0.162	3.92E-13	VEC
ADAMTS1	2.49E-17	0.64458	0.375	0.052	4.54E-13	VEC
COL6A1	4.97E-17	1.142152	0.688	0.166	9.05E-13	VEC
CFH	5.53E-17	1.448007	0.688	0.166	1.01E-12	VEC
CEBPD	6.09E-17	1.374764	0.969	0.602	1.11E-12	VEC
SELM	8.29E-17	1.119943	0.906	0.342	1.51E-12	VEC
FHL1	1.78E-16	0.914666	0.656	0.159	3.23E-12	VEC
ANXA6	3.77E-16	0.61795	0.344	0.047	6.86E-12	VEC
LHFP	3.98E-16	1.063036	0.781	0.248	7.24E-12	VEC
SPON1	6.08E-16	0.466743	0.281	0.033	1.11E-11	VEC
NTRK3	1.86E-15	0.547224	0.344	0.048	3.39E-11	VEC
IGFBP7	2.18E-15	1.537144	0.969	0.658	3.98E-11	VEC
ICAM1	7.04E-15	0.962375	0.406	0.071	1.28E-10	VEC
CTGF	9.21E-15	1.35467	0.688	0.2	1.68E-10	VEC
TWIST2	1.07E-14	0.735368	0.5	0.103	1.96E-10	VEC
EGFL7	1.12E-14	0.992987	0.344	0.052	2.04E-10	VEC
RPS27A	1.26E-14	0.712606	1	0.997	2.29E-10	VEC
ARID5B	1.47E-14	1.230343	0.844	0.429	2.68E-10	VEC
SPARCL1	2.14E-14	1.735045	0.75	0.268	3.90E-10	VEC
SRGN	5.03E-14	0.39933	0.281	0.035	9.16E-10	VEC
LGALS1	5.10E-14	1.097899	0.906	0.327	9.29E-10	VEC
COL1A1	8.56E-14	0.838273	0.375	0.064	1.56E-09	VEC
RGS16	1.37E-13	1.222785	0.625	0.181	2.49E-09	VEC

EEF1D	2.08E-13	0.645622	1	0.978	3.80E-09	VEC
CCDC80	3.45E-13	0.657238	0.531	0.128	6.27E-09	VEC
RPL10	5.79E-13	0.610982	1	1	1.05E-08	VEC
BC005927	9.82E-13	0.847789	0.469	0.105	1.79E-08	VEC
CH25H	2.11E-12	0.435472	0.281	0.041	3.84E-08	VEC
FAM107A	2.16E-12	0.734479	0.406	0.081	3.93E-08	VEC
HLA-DPB1	2.52E-12	0.6468	0.406	0.084	4.59E-08	VEC
TGFB1I1	2.53E-12	0.487711	0.312	0.051	4.61E-08	VEC
CCL2	2.55E-12	0.805969	0.469	0.101	4.65E-08	VEC
CALD1	2.95E-12	1.101807	0.75	0.288	5.37E-08	VEC
TUBB2B	3.02E-12	0.58596	0.344	0.059	5.50E-08	VEC
B2M	3.64E-12	1.017189	1	0.997	6.62E-08	VEC
TSC22D1	4.33E-12	1.407638	0.938	0.587	7.88E-08	VEC
ZFP36L2	5.25E-12	1.252298	0.844	0.522	9.56E-08	VEC
CNN3	5.79E-12	0.859175	0.844	0.36	1.05E-07	VEC
CTSK	8.94E-12	0.933968	0.469	0.113	1.63E-07	VEC
FGFR1	1.21E-11	0.703907	0.5	0.126	2.21E-07	VEC
TIMP3	1.22E-11	1.380555	0.781	0.346	2.22E-07	VEC
STOM	1.30E-11	0.91137	0.75	0.384	2.37E-07	VEC
GADD45B	1.37E-11	0.976971	1	0.928	2.50E-07	VEC
RPL13	1.46E-11	0.604186	1	0.999	2.66E-07	VEC
ARMCX1	2.53E-11	0.612928	0.375	0.075	4.60E-07	VEC
RPL12	2.55E-11	0.613614	1	0.998	4.64E-07	VEC
PTMA	2.56E-11	0.870871	1	0.996	4.66E-07	VEC
HLA-C	3.51E-11	0.791017	1	0.956	6.40E-07	VEC
FBLN5	3.73E-11	0.715608	0.531	0.136	6.78E-07	VEC
SERPINE1	4.74E-11	0.649049	0.312	0.055	8.63E-07	VEC
ANXA5	6.59E-11	0.856834	0.844	0.416	1.20E-06	VEC
AEBP1	7.79E-11	0.96489	0.469	0.122	1.42E-06	VEC
EEF1A1	1.07E-10	0.531751	1	1	1.94E-06	VEC
IL6ST	1.17E-10	0.718915	0.531	0.165	2.13E-06	VEC
VWA1	1.35E-10	0.528792	0.344	0.07	2.46E-06	VEC
NPM1	1.60E-10	0.651641	1	0.928	2.92E-06	VEC
TGFBR3	2.14E-10	0.499806	0.312	0.059	3.89E-06	VEC
SOCS3	2.47E-10	1.272682	0.719	0.333	4.50E-06	VEC
PPAP2A	3.30E-10	0.936657	0.656	0.298	6.01E-06	VEC
KLF2	3.43E-10	0.628322	0.312	0.061	6.24E-06	VEC
MMP2	3.52E-10	0.941465	0.438	0.112	6.41E-06	VEC
FIBIN	3.95E-10	0.686937	0.5	0.129	7.19E-06	VEC
HSD17B11	4.06E-10	0.700986	0.438	0.116	7.40E-06	VEC
RPL3	4.17E-10	0.489473	1	0.999	7.60E-06	VEC
SNHG8	4.48E-10	0.957339	0.875	0.7	8.16E-06	VEC
VAMP5	5.69E-10	0.71349	0.469	0.133	1.04E-05	VEC
HSP90AB1	6.06E-10	0.621338	0.969	0.963	1.10E-05	VEC
SEPP1	6.49E-10	1.266441	0.844	0.469	1.18E-05	VEC
ZEB1	1.21E-09	0.385386	0.344	0.071	2.21E-05	VEC
PRRX1	1.48E-09	0.906625	0.5	0.139	2.70E-05	VEC
HLA-A	1.74E-09	0.893076	1	0.961	3.16E-05	VEC

CPQ	1.94E-09	0.66432	0.5	0.146	3.54E-05	VEC
CD63	2.16E-09	0.647962	1	0.98	3.94E-05	VEC
SOD2	2.19E-09	1.926128	0.656	0.321	3.99E-05	VEC
TSHZ2	2.29E-09	0.601778	0.312	0.064	4.17E-05	VEC
RPL32	2.47E-09	0.54583	1	0.999	4.50E-05	VEC
EFEMP2	2.69E-09	0.439648	0.344	0.076	4.90E-05	VEC
MSN	3.24E-09	0.506506	0.406	0.102	5.90E-05	VEC
RPS23	3.24E-09	0.566998	1	0.998	5.91E-05	VEC
CDC42EP3	3.93E-09	0.700659	0.469	0.139	7.15E-05	VEC
RPL7	4.90E-09	0.628603	1	0.999	8.92E-05	VEC
NFKBIZ	4.93E-09	0.901906	0.688	0.343	8.98E-05	VEC
TPST1	5.06E-09	0.432875	0.281	0.055	9.21E-05	VEC
TXNIP	5.51E-09	1.216787	0.906	0.676	0.0001	VEC
DPYSL2	6.16E-09	0.498687	0.469	0.134	0.000112	VEC
COL1A2	7.02E-09	0.639295	0.531	0.158	0.000128	VEC
PAM	7.31E-09	0.786808	0.5	0.153	0.000133	VEC
CRIP2	7.45E-09	0.982854	0.562	0.219	0.000136	VEC
SAV1	7.81E-09	0.417842	0.344	0.081	0.000142	VEC
RPS27	7.98E-09	0.421231	1	1	0.000145	VEC
PLSCR4	8.37E-09	0.477554	0.406	0.105	0.000152	VEC
ACTN1	8.40E-09	0.802974	0.5	0.169	0.000153	VEC
C1QTNF7	1.10E-08	0.492292	0.312	0.066	0.000199	VEC
RPL35A	1.19E-08	0.444662	1	0.998	0.000218	VEC
TIMP2	1.53E-08	0.5172	0.5	0.146	0.000279	VEC
RPL17	1.73E-08	0.575667	1	0.985	0.000316	VEC
RPS3	1.76E-08	0.524046	1	0.996	0.000321	VEC
EPB41L2	1.83E-08	0.318436	0.312	0.067	0.000332	VEC
SNAI1	1.87E-08	0.950702	0.438	0.135	0.000341	VEC
HNRNPA1	2.01E-08	0.477132	1	0.97	0.000365	VEC
RNASE4	2.09E-08	0.966638	0.656	0.293	0.00038	VEC
SERPINH1	2.26E-08	0.773171	0.5	0.18	0.000412	VEC
RPL6	2.37E-08	0.482121	0.969	0.992	0.000432	VEC
KCTD12	2.60E-08	0.408606	0.344	0.081	0.000474	VEC
VIT	2.66E-08	0.993948	0.469	0.17	0.000484	VEC
RPS16	2.99E-08	0.402907	1	0.994	0.000545	VEC
EEF2	3.41E-08	0.597421	0.938	0.874	0.00062	VEC
MID1IP1	3.59E-08	1.040584	0.625	0.314	0.000654	VEC
LEPROT	3.60E-08	0.779219	0.75	0.505	0.000656	VEC
SH3BP5	4.27E-08	0.600491	0.344	0.09	0.000778	VEC
RPS2	5.05E-08	0.603763	1	0.997	0.000919	VEC
RPL13A	5.55E-08	0.449744	1	0.999	0.001011	VEC
PITX2	6.74E-08	0.596508	0.406	0.113	0.001226	VEC
LAMC1	6.92E-08	0.391309	0.281	0.062	0.00126	VEC
MAGI2-AS3	7.62E-08	0.45475	0.312	0.074	0.001387	VEC
RPS15	8.37E-08	0.326142	1	1	0.001524	VEC
RPS6	9.30E-08	0.454092	1	0.999	0.001693	VEC
ZBTB16	9.36E-08	0.800983	0.312	0.077	0.001703	VEC
COL6A2	9.39E-08	0.632152	0.375	0.103	0.00171	VEC

RPL26	9.41E-08	0.443845	1	0.999	0.001713	VEC
SOD3	9.69E-08	0.855742	0.688	0.288	0.001765	VEC
MYL9	9.89E-08	1.088633	0.344	0.088	0.001801	VEC
ZNF503	1.14E-07	0.908258	0.5	0.205	0.002082	VEC
RASD1	1.22E-07	1.20481	0.719	0.443	0.002224	VEC
C6orf48	1.44E-07	0.610889	0.875	0.651	0.00263	VEC
RPS9	1.49E-07	0.43349	1	0.998	0.002705	VEC
APOD	1.49E-07	1.166726	0.844	0.651	0.002718	VEC
LY6E	1.62E-07	0.824745	0.844	0.543	0.002946	VEC
SNHG5	1.63E-07	0.648012	0.969	0.918	0.002961	VEC
RPL39	1.69E-07	0.333282	1	0.999	0.003078	VEC
TIMP1	1.70E-07	0.724046	0.875	0.641	0.003087	VEC
SMCR7L	1.73E-07	0.355001	0.281	0.063	0.003152	VEC
SLC9A3R2	2.00E-07	0.473452	0.312	0.08	0.003643	VEC
FN1	2.07E-07	0.287007	0.281	0.06	0.003774	VEC
FXYD5	2.17E-07	0.517071	0.469	0.156	0.003948	VEC
HLA-DPA1	2.29E-07	0.814971	0.344	0.097	0.004176	VEC
SGCB	2.31E-07	0.535287	0.344	0.097	0.004215	VEC
CCNI	2.41E-07	0.797289	0.781	0.541	0.004396	VEC
IL11RA	2.65E-07	0.481283	0.281	0.067	0.004831	VEC
SRRM2	3.83E-07	0.520352	0.781	0.505	0.00698	VEC
RPL34	4.17E-07	0.453079	1	1	0.007592	VEC
RCN1	5.28E-07	0.632679	0.688	0.408	0.009608	VEC
PTN	5.34E-07	0.815092	0.344	0.095	0.009718	VEC
AKR1C3	5.39E-07	0.554526	0.375	0.117	0.009818	VEC
VASN	5.47E-07	0.656457	0.344	0.101	0.009958	VEC
RPS13	5.54E-07	0.34517	1	0.998	0.010088	VEC
LUZP1	6.38E-07	0.672564	0.562	0.271	0.011623	VEC
PFKFB3	6.80E-07	0.516268	0.375	0.112	0.012381	VEC
CXCL1	6.91E-07	1.305441	0.406	0.133	0.012588	VEC
BC016015	7.25E-07	1.060143	0.375	0.122	0.013206	VEC
SORBS3	8.71E-07	0.592232	0.5	0.208	0.015853	VEC
TSPAN4	9.11E-07	0.668983	0.562	0.255	0.016594	VEC
RPL31	9.21E-07	0.466725	1	0.998	0.016769	VEC
GNB2L1	1.01E-06	0.466995	1	0.99	0.01836	VEC
OLFML3	1.05E-06	0.576306	0.5	0.166	0.019169	VEC
AKAP13	1.14E-06	0.559001	0.469	0.179	0.020757	VEC
RRAS	1.27E-06	0.374201	0.312	0.087	0.023153	VEC
CAV1	1.32E-06	0.817045	0.562	0.264	0.024096	VEC
PLIN2	1.41E-06	0.908344	0.406	0.148	0.025651	VEC
RPL11	1.45E-06	0.391108	1	0.999	0.026426	VEC
CMTM3	1.70E-06	0.4185	0.312	0.088	0.03101	VEC
PPP1R18	2.00E-06	0.537925	0.406	0.146	0.036437	VEC
PHLDA1	2.02E-06	0.824885	0.5	0.206	0.036717	VEC
CXCL2	2.05E-06	1.287979	0.531	0.234	0.037323	VEC
MYLIP	2.34E-06	0.699014	0.625	0.352	0.042596	VEC
TMEM173	2.61E-06	0.573013	0.375	0.131	0.047438	VEC
JUN	2.73E-06	0.776844	1	0.979	0.049755	VEC

FKBP10	2.75E-06	0.508345	0.312	0.093	0.050126	VEC
RPS5	2.79E-06	0.428766	1	0.99	0.050766	VEC
GAS5	3.07E-06	0.579702	0.969	0.934	0.055979	VEC
RPL10A	3.34E-06	0.393491	1	0.995	0.060836	VEC
ID2	3.50E-06	1.230132	0.781	0.559	0.063652	VEC
FOXO3	4.05E-06	0.467463	0.406	0.147	0.073707	VEC
RPS8	4.44E-06	0.459654	1	0.999	0.080819	VEC
PRKCDBP	5.36E-06	0.601718	0.562	0.26	0.09762	VEC
RPS4X	5.48E-06	0.423624	1	0.999	0.099774	VEC
MXRA8	5.60E-06	0.416219	0.344	0.105	0.101904	VEC
C11orf96	6.25E-06	0.564811	0.281	0.077	0.113809	VEC
NME4	6.90E-06	0.484234	0.438	0.174	0.125673	VEC
CYB5R3	7.03E-06	0.748436	0.625	0.423	0.128087	VEC
RPS18	7.10E-06	0.450356	1	1	0.12923	VEC
PTRF	7.48E-06	0.655925	0.719	0.455	0.136243	VEC
HLA-E	7.66E-06	0.914415	0.906	0.885	0.139413	VEC
C16orf45	8.02E-06	0.439484	0.344	0.111	0.146112	VEC
FSTL1	8.10E-06	0.536015	0.375	0.132	0.14741	VEC
SDCBP	9.48E-06	0.615684	0.75	0.565	0.17261	VEC
RPL29	9.81E-06	0.445861	1	0.993	0.178612	VEC
FMOD	9.91E-06	0.60772	0.406	0.141	0.180403	VEC
RPS19	1.00E-05	0.4873	1	0.998	0.182763	VEC
MAP1LC3A	1.05E-05	0.531172	0.469	0.201	0.190653	VEC
RPS25	1.12E-05	0.354671	1	0.999	0.203285	VEC
PTMS	1.12E-05	0.614203	0.719	0.542	0.203549	VEC
SVIL	1.15E-05	0.646482	0.562	0.284	0.208889	VEC
EPHX1	1.23E-05	0.666092	0.719	0.495	0.22472	VEC
SDC2	1.32E-05	0.347491	0.5	0.172	0.239668	VEC
RPL21	1.37E-05	0.32575	1	0.999	0.248622	VEC
WTAP	1.81E-05	0.664576	0.625	0.391	0.328855	VEC
FCGRT	2.00E-05	1.004049	0.531	0.311	0.363482	VEC
PPP1R15A	2.41E-05	0.642217	0.969	0.917	0.438903	VEC
RPL18A	2.46E-05	0.393056	0.969	0.997	0.447431	VEC
PCBP2	2.58E-05	0.39512	0.875	0.816	0.469526	VEC
RSL1D1	2.93E-05	0.577825	0.812	0.688	0.533379	VEC
RPL5	2.97E-05	0.476102	1	0.993	0.540511	VEC
BGN	3.07E-05	0.901138	0.469	0.237	0.558147	VEC
SON	3.12E-05	0.502922	0.781	0.69	0.567742	VEC
TMEM98	3.22E-05	0.506215	0.406	0.168	0.586025	VEC
QKI	3.25E-05	0.324836	0.344	0.12	0.590915	VEC
FRMD6	3.38E-05	0.416412	0.375	0.148	0.615047	VEC
LOC541471	3.60E-05	0.542192	0.406	0.167	0.655351	VEC
MT1M	3.68E-05	0.792443	0.469	0.218	0.670942	VEC
MSS51	4.17E-05	0.384494	1	0.979	0.758884	VEC
RPS3A	4.19E-05	0.333525	1	0.998	0.762356	VEC
RPL41	4.22E-05	0.277246	1	1	0.768971	VEC
RPL36A	4.30E-05	0.503123	1	0.989	0.782482	VEC
NFKBIA	4.75E-05	0.950246	0.938	0.943	0.864178	VEC

PKIG	4.82E-05	0.386976	0.406	0.176	0.876892	VEC
GBP2	4.82E-05	0.376338	0.281	0.089	0.878438	VEC
LAPTM4A	4.84E-05	0.591425	1	0.971	0.880597	VEC
TRAM1	4.92E-05	0.519379	0.656	0.442	0.896031	VEC
RPS11	5.36E-05	0.354765	0.969	0.985	0.976841	VEC
CXCL3	5.92E-05	0.848192	0.281	0.087	1	VEC
RPL15	6.92E-05	0.287693	1	0.999	1	VEC
CD320	6.99E-05	0.398903	0.281	0.089	1	VEC
KIAA0930	7.26E-05	0.410977	0.281	0.092	1	VEC
COTL1	7.38E-05	0.605997	0.312	0.114	1	VEC
APCDD1	7.42E-05	0.500362	0.281	0.09	1	VEC
GLTSCR2	9.80E-05	0.677037	0.844	0.828	1	VEC
CCDC85B	0.000102	0.551197	0.688	0.551	1	VEC
DAD1	0.000104	0.36883	0.875	0.839	1	VEC
RPL36	0.000106	0.419468	1	0.997	1	VEC
RTN4	0.000107	0.451948	0.844	0.896	1	VEC
ARRDC3	0.000112	0.593578	0.531	0.288	1	VEC
RPL18	0.000127	0.272131	1	0.996	1	VEC
PPIC	0.000128	0.520573	0.531	0.312	1	VEC
SASH1	0.000129	0.278034	0.375	0.145	1	VEC
SERPINB6	0.000139	0.51311	0.5	0.268	1	VEC
BRD2	0.000151	0.385824	0.75	0.596	1	VEC
SSB	0.000177	0.332223	0.875	0.727	1	VEC
ZNF22	0.000179	0.379952	0.281	0.1	1	VEC
SERPINF1	0.000182	0.879366	0.688	0.525	1	VEC
RIF1	0.00019	0.34434	0.281	0.098	1	VEC
CYBRD1	0.000223	0.455615	0.562	0.311	1	VEC
RPS15A	0.000227	0.355796	1	0.997	1	VEC
FKBP7	0.00023	0.320071	0.312	0.116	1	VEC
ID3	0.000237	0.666499	0.844	0.76	1	VEC
GSTM3	0.000249	0.58223	0.469	0.231	1	VEC
IFRD1	0.000266	0.538686	0.594	0.411	1	VEC
FTL	0.000267	0.436293	1	0.996	1	VEC
HLA-B	0.000283	0.642649	0.938	0.925	1	VEC
CD276	0.000294	0.289364	0.312	0.119	1	VEC
RPL35	0.00032	0.285442	1	0.999	1	VEC
AXL	0.000339	0.364259	0.344	0.131	1	VEC
UBC	0.00035	0.355181	0.969	0.999	1	VEC
CTSZ	0.000365	0.308019	0.344	0.138	1	VEC
SPARC	0.000379	0.584568	0.719	0.453	1	VEC
COL6A3	0.000391	0.556093	0.344	0.139	1	VEC
NMB	0.000398	0.320588	0.281	0.104	1	VEC
LOC100996255	0.000417	0.313622	0.438	0.218	1	VEC
KLF6	0.000472	0.77616	0.75	0.677	1	VEC
TPM4	0.000486	0.648394	0.625	0.492	1	VEC
LOC654342	0.00049	0.835155	0.406	0.226	1	VEC
EFHD1	0.000515	0.31759	0.312	0.114	1	VEC
RPS17L	0.000557	0.306788	1	0.996	1	VEC

RPL7A	0.000558	0.333234	1	0.987	1	VEC
PFDN5	0.000584	0.386052	0.969	0.974	1	VEC
EEF1G	0.000603	0.272563	1	0.988	1	VEC
ZBTB20	0.000622	0.364761	0.344	0.152	1	VEC
NAMPT	0.000641	0.690142	0.531	0.395	1	VEC
NAP1L1	0.000651	0.491124	0.781	0.736	1	VEC
LMNA	0.000665	0.378313	0.938	0.967	1	VEC
EIF3E	0.000675	0.397769	0.844	0.822	1	VEC
MAFF	0.000679	0.594106	0.625	0.439	1	VEC
MYC	0.000719	0.643836	0.719	0.566	1	VEC
LOX	0.000724	0.305831	0.375	0.157	1	VEC
AMOTL2	0.000735	0.32164	0.312	0.129	1	VEC
RPL28	0.000744	0.322822	1	0.992	1	VEC
CD59	0.00075	0.568355	0.812	0.734	1	VEC
TMEM204	0.000776	0.301362	0.375	0.156	1	VEC
EHD2	0.000781	0.366505	0.344	0.155	1	VEC
SSPN	0.000797	0.499049	0.406	0.214	1	VEC
RGS3	0.000809	0.315745	0.281	0.108	1	VEC
RPS14	0.000836	0.296041	1	1	1	VEC
CANX	0.000847	0.370521	0.719	0.608	1	VEC
OAZ2	0.000855	0.430738	0.594	0.444	1	VEC
LGMN	0.000877	0.445365	0.469	0.277	1	VEC
NFIC	0.000929	0.584326	0.562	0.402	1	VEC
JAM3	0.000985	0.474301	0.312	0.132	1	VEC
RPLP2	0.001014	0.259422	1	0.999	1	VEC
RPL38	0.001021	0.343315	1	0.989	1	VEC
JMJD1C	0.001048	0.532377	0.469	0.272	1	VEC
OSBPL8	0.001049	0.566168	0.344	0.169	1	VEC
HIAT1	0.001057	0.40693	0.406	0.224	1	VEC
SLC39A1	0.001061	0.433079	0.656	0.544	1	VEC
NR4A2	0.001087	0.765846	0.375	0.201	1	VEC
FSCN1	0.001143	0.557287	0.438	0.246	1	VEC
SEC62	0.001158	0.298171	0.875	0.845	1	VEC
FBLN2	0.001205	0.484678	0.312	0.138	1	VEC
ETS2	0.001233	0.688329	0.531	0.358	1	VEC
ZFAND5	0.0013	0.591532	0.75	0.664	1	VEC
EVA1C	0.001304	0.514974	0.344	0.168	1	VEC
PCOLCE	0.001348	0.547098	0.312	0.137	1	VEC
CAT	0.001363	0.488925	0.438	0.27	1	VEC
ZFAS1	0.001363	0.520864	0.906	0.867	1	VEC
TXLNG	0.001376	0.43797	0.312	0.146	1	VEC
RPS14P3	0.001387	0.32601	0.969	0.962	1	VEC
SMIM3	0.001435	0.397695	0.375	0.185	1	VEC
FBLN1	0.001448	1.356966	0.438	0.295	1	VEC
EHD4	0.001491	0.379164	0.312	0.144	1	VEC
LSM14A	0.001494	0.315061	0.625	0.497	1	VEC
GADD45A	0.001525	0.440087	0.719	0.601	1	VEC
TMEM45A	0.001532	0.497917	0.344	0.164	1	VEC

BIN3	0.001545	0.304057	0.281	0.118	1	VEC
RPSA	0.001548	0.345005	0.875	0.871	1	VEC
SNX9	0.001643	0.394421	0.375	0.2	1	VEC
KMT2E	0.00165	0.599546	0.656	0.588	1	VEC
EIF4A2	0.001671	0.483774	0.906	0.903	1	VEC
RAB32	0.001698	0.441758	0.344	0.161	1	VEC
TFAP2B	0.00173	0.445362	0.312	0.13	1	VEC
LINC00152	0.001763	0.299082	0.281	0.113	1	VEC
MTDH	0.001799	0.385843	0.688	0.574	1	VEC
SNHG6	0.001866	0.332172	0.875	0.76	1	VEC
KLF9	0.001944	0.557978	0.406	0.242	1	VEC
HLA-F	0.001989	0.3071	0.281	0.12	1	VEC
ZFP36L1	0.002022	0.399196	0.938	0.847	1	VEC
RBM8A	0.002025	0.33133	0.812	0.806	1	VEC
HEXA	0.002048	0.478644	0.469	0.293	1	VEC
QSOX1	0.002064	0.255936	0.312	0.134	1	VEC
NFIB	0.002304	0.33367	0.344	0.167	1	VEC
ERGIC3	0.00233	0.35718	0.688	0.66	1	VEC
TNPO1	0.002335	0.435508	0.344	0.171	1	VEC
XRN1	0.002492	0.280667	0.281	0.127	1	VEC
TTC28	0.002518	0.370367	0.406	0.228	1	VEC
MBNL1	0.002533	0.286012	0.312	0.142	1	VEC
NR2F2	0.002583	0.6436	0.312	0.152	1	VEC
PLTP	0.002605	0.508456	0.281	0.128	1	VEC
SEC11A	0.002621	0.382618	0.719	0.713	1	VEC
MGLL	0.002673	0.343393	0.281	0.12	1	VEC
NASP	0.002701	0.374069	0.531	0.373	1	VEC
BACE2	0.002729	0.350568	0.281	0.126	1	VEC
PDLIM2	0.002788	0.381292	0.375	0.202	1	VEC
SPAG9	0.003005	0.291415	0.344	0.174	1	VEC
VMP1	0.00303	0.332479	0.531	0.384	1	VEC
RPL24	0.003063	0.250742	1	0.994	1	VEC
APP	0.003116	0.655829	0.656	0.567	1	VEC
UBE2J1	0.003267	0.43008	0.438	0.278	1	VEC
TLE4	0.003334	0.479673	0.375	0.224	1	VEC
EMP3	0.003395	0.541149	0.469	0.28	1	VEC
TMSB10	0.003639	0.549808	1	0.997	1	VEC
PRSS23	0.003741	0.587835	0.406	0.227	1	VEC
RGS2	0.003769	0.472164	0.438	0.279	1	VEC
CHD1	0.003836	0.359092	0.344	0.189	1	VEC
HSP90B1	0.00384	0.39092	0.844	0.824	1	VEC
FAM195B	0.003911	0.302802	0.312	0.155	1	VEC
RPLP0	0.00404	0.290404	1	0.992	1	VEC
DUSP6	0.004295	0.508888	0.375	0.209	1	VEC
SRSF11	0.004307	0.377177	0.812	0.757	1	VEC
ITGB1BP1	0.004335	0.459389	0.531	0.417	1	VEC
IFT57	0.004413	0.402269	0.469	0.326	1	VEC
DDIT4	0.004591	0.632211	0.625	0.463	1	VEC

MFGE8	0.00461	0.680452	0.469	0.325	1	VEC
RPL37	0.004621	0.272302	1	0.995	1	VEC
L32131	0.004833	0.429417	0.719	0.745	1	VEC
MAD2L2	0.005592	0.305891	0.312	0.158	1	VEC
SSBP4	0.005618	0.251888	0.312	0.15	1	VEC
RBM27	0.005672	0.372813	0.438	0.271	1	VEC
PGRMC1	0.005734	0.399081	0.719	0.646	1	VEC
RPS27L	0.005906	0.423519	0.812	0.846	1	VEC
PLSCR1	0.006064	0.342559	0.375	0.21	1	VEC
EID1	0.006402	0.380988	0.812	0.884	1	VEC
HSF1	0.006622	0.359481	0.406	0.262	1	VEC
UBXN4	0.006628	0.350909	0.719	0.689	1	VEC
CERS2	0.006731	0.372689	0.406	0.253	1	VEC
MAPK3	0.006878	0.389695	0.344	0.19	1	VEC
SEC22C	0.006936	0.398282	0.312	0.164	1	VEC
RPL9	0.006986	0.411114	0.75	0.787	1	VEC
WWTR1	0.007092	0.267379	0.281	0.138	1	VEC
C5orf24	0.007116	0.402466	0.312	0.176	1	VEC
IRF1	0.007288	0.614539	0.5	0.378	1	VEC
MPHOSPH8	0.007457	0.386169	0.562	0.483	1	VEC
H1FX	0.007572	0.555851	0.531	0.422	1	VEC
SOX4	0.007828	0.453007	0.531	0.382	1	VEC
SPTBN1	0.008129	0.430078	0.344	0.198	1	VEC
NR3C1	0.008206	0.322365	0.312	0.164	1	VEC
IPO7	0.008231	0.299284	0.312	0.156	1	VEC
XAB2	0.008326	0.286093	0.281	0.139	1	VEC
SEPT2	0.008328	0.28062	0.625	0.504	1	VEC
BCAR1	0.00836	0.275433	0.312	0.167	1	VEC
SMC4	0.008481	0.293813	0.281	0.143	1	VEC
CTSH	0.008577	0.463383	0.406	0.267	1	VEC
LSP1	0.008629	0.459672	0.5	0.329	1	VEC
C19orf10	0.008722	0.361949	0.656	0.597	1	VEC
TJP1	0.008902	0.303865	0.312	0.173	1	VEC
WASF2	0.009575	0.341862	0.531	0.376	1	VEC
RPS10	0.009656	0.302078	0.875	0.979	1	VEC
PSIP1	0.009766	0.363279	0.406	0.261	1	VEC
NUCKS1	0.009826	0.31243	0.75	0.774	1	VEC
C19orf66	0.009955	0.352323	0.375	0.247	1	VEC
CA3	0	4.088734	1	0.01	0	CenC
FGF7	0	2.208004	1	0.009	0	CenC
MSMP	0	2.17086	0.964	0.002	0	CenC
TMEM178A	0	1.546879	0.857	0.002	0	CenC
EDN3	0	1.530471	0.857	0.006	0	CenC
AGXT2L1	0	1.168631	0.714	0	0	CenC
PVALB	0	1.104873	0.607	0	0	CenC
FGF9	0	0.926191	0.679	0.007	0	CenC
STC1	0	0.839382	0.5	0.004	0	CenC
KISS1	0	0.696484	0.5	0.001	0	CenC

NELL2	0	0.56955	0.536	0.002	0	CenC
SHC4	0	0.562504	0.464	0.001	0	CenC
CDH2	0	0.54296	0.536	0.001	0	CenC
NOG	0	0.500715	0.5	0.004	0	CenC
CRABP1	0	0.480106	0.286	0.001	0	CenC
PLK5	0	0.476208	0.393	0.001	0	CenC
CHGB	0	0.459093	0.357	0.001	0	CenC
CEND1	0	0.392736	0.357	0.001	0	CenC
ADCYAP1R1	0	0.386844	0.321	0	0	CenC
RGS7BP	0	0.386464	0.357	0	0	CenC
GAL3ST3	0	0.355975	0.393	0	0	CenC
LRRN1	0	0.332713	0.357	0.001	0	CenC
RALYL	0	0.325445	0.357	0.001	0	CenC
POU6F2	0	0.314586	0.393	0	0	CenC
PIP5K1B	5.59E-297	1.085468	0.929	0.016	1.02E-292	CenC
MT3	5.43E-283	0.295369	0.321	0.002	9.89E-279	CenC
FZD2	1.31E-269	0.554403	0.429	0.003	2.38E-265	CenC
MIA	2.22E-234	0.714153	0.536	0.006	4.04E-230	CenC
A2M	1.64E-219	0.595615	0.357	0.003	2.98E-215	CenC
SLC4A4	4.09E-201	1.311377	0.964	0.027	7.44E-197	CenC
RGS5	1.28E-188	2.898271	1	0.032	2.33E-184	CenC
PAPPA	1.45E-188	0.959591	0.714	0.015	2.64E-184	CenC
CLRN1	7.57E-187	0.286226	0.286	0.002	1.38E-182	CenC
XAGE2	1.35E-186	0.522936	0.5	0.007	2.47E-182	CenC
ITM2C	2.17E-183	1.942354	1	0.033	3.96E-179	CenC
PCDH7	1.05E-179	1.264456	0.964	0.03	1.91E-175	CenC
GNG4	3.44E-179	0.368089	0.286	0.002	6.26E-175	CenC
CDO1	3.13E-178	0.408799	0.321	0.003	5.69E-174	CenC
CLDN10	3.43E-174	0.511808	0.429	0.006	6.25E-170	CenC
ASGR1	2.29E-168	0.454471	0.5	0.008	4.16E-164	CenC
OGDHL	4.58E-166	0.304486	0.393	0.005	8.33E-162	CenC
TNNC1	2.13E-159	2.05545	0.964	0.035	3.88E-155	CenC
PLCE1	3.36E-156	0.307445	0.321	0.003	6.11E-152	CenC
MICAL2	7.18E-152	0.612416	0.714	0.019	1.31E-147	CenC
FAM181B	1.34E-151	0.463269	0.429	0.007	2.44E-147	CenC
ANK1	2.66E-149	0.391492	0.429	0.007	4.84E-145	CenC
PLCE1-AS1	1.94E-147	0.461626	0.321	0.004	3.52E-143	CenC
AJAP1	3.16E-147	0.259474	0.321	0.004	5.76E-143	CenC
LIF	2.94E-145	0.376801	0.321	0.004	5.36E-141	CenC
HAGHL	3.41E-144	0.494847	0.5	0.01	6.21E-140	CenC
DAAM2	3.02E-138	0.276282	0.286	0.003	5.50E-134	CenC
TMOD1	7.30E-137	0.417356	0.464	0.009	1.33E-132	CenC
CYP1A2	1.12E-132	0.370284	0.393	0.006	2.04E-128	CenC
LINC00839	7.16E-127	0.278985	0.357	0.005	1.30E-122	CenC
NECAB1	2.46E-120	0.604678	0.607	0.017	4.48E-116	CenC
SFRP1	2.17E-109	2.092107	1	0.058	3.95E-105	CenC
COL4A3	1.48E-108	1.193394	0.929	0.048	2.69E-104	CenC
SH3BP4	2.36E-103	0.673261	0.643	0.023	4.29E-99	CenC

MAOB	2.60E-102	0.381671	0.321	0.006	4.74E-98	CenC
SALL2	4.94E-100	0.293023	0.286	0.004	9.00E-96	CenC
FADS2	3.10E-94	0.747714	0.643	0.025	5.64E-90	CenC
NTN1	5.32E-94	0.660501	0.786	0.038	9.68E-90	CenC
APOE	6.67E-94	1.273927	0.929	0.055	1.22E-89	CenC
PIFO	7.06E-94	0.4231	0.429	0.011	1.29E-89	CenC
SOBP	2.20E-93	0.398636	0.464	0.013	4.01E-89	CenC
CCDC144CP	1.01E-91	0.992844	0.643	0.026	1.84E-87	CenC
COL8A2	1.35E-89	1.552421	0.964	0.065	2.46E-85	CenC
NRXN3	1.16E-87	0.261978	0.286	0.005	2.11E-83	CenC
ATP1B2	1.10E-86	0.480283	0.393	0.01	2.00E-82	CenC
SYNPO	8.45E-84	0.279019	0.393	0.01	1.54E-79	CenC
ENO2	7.78E-82	1.068262	0.75	0.041	1.42E-77	CenC
SCD	5.55E-80	1.960239	0.929	0.07	1.01E-75	CenC
MARCH3	3.12E-75	0.613432	0.571	0.025	5.68E-71	CenC
TPM2	2.15E-74	1.075244	0.857	0.06	3.92E-70	CenC
DNAJC6	4.91E-72	0.33579	0.393	0.012	8.95E-68	CenC
FGF11	9.22E-71	0.263127	0.321	0.008	1.68E-66	CenC
TGFBR3	1.22E-69	0.944784	0.821	0.058	2.23E-65	CenC
LOC339535	5.41E-68	0.605936	0.536	0.024	9.86E-64	CenC
SLC4A11	7.24E-67	2.233627	1	0.102	1.32E-62	CenC
RAP1GAP	5.82E-66	0.515204	0.571	0.029	1.06E-61	CenC
RBP7	4.18E-65	0.371605	0.321	0.009	7.62E-61	CenC
MRAP2	4.40E-64	0.252334	0.286	0.007	8.02E-60	CenC
ISYNA1	6.65E-64	0.874186	0.786	0.058	1.21E-59	CenC
GLIS3	1.89E-61	0.348963	0.321	0.01	3.44E-57	CenC
MYOC	7.08E-60	2.836388	0.964	0.097	1.29E-55	CenC
CA12	8.74E-58	2.022894	1	0.121	1.59E-53	CenC
ALCAM	2.21E-57	1.3188	0.929	0.096	4.02E-53	CenC
C1QTNF4	4.50E-57	0.346431	0.393	0.015	8.19E-53	CenC
SULF1	3.87E-54	0.529506	0.536	0.03	7.04E-50	CenC
RBM38	9.07E-54	0.422992	0.429	0.019	1.65E-49	CenC
SYMPK	1.16E-53	1.016179	0.821	0.077	2.12E-49	CenC
TBX2	3.30E-53	0.506499	0.571	0.034	6.02E-49	CenC
MPP1	2.51E-52	0.671464	0.714	0.056	4.57E-48	CenC
SYNJ1	3.92E-52	0.473609	0.429	0.02	7.14E-48	CenC
PROCR	7.29E-52	0.328429	0.357	0.014	1.33E-47	CenC
COL4A4	1.17E-51	0.661156	0.571	0.036	2.14E-47	CenC
MGP	4.52E-51	3.819393	1	0.142	8.22E-47	CenC
PVRL3	5.32E-51	0.367605	0.321	0.012	9.68E-47	CenC
ZDHHC14	1.20E-50	0.408057	0.393	0.017	2.18E-46	CenC
RAB17	2.55E-50	0.835123	0.679	0.054	4.64E-46	CenC
NCAM1	5.94E-50	0.626639	0.643	0.046	1.08E-45	CenC
PPARGC1B	6.50E-48	0.387858	0.357	0.015	1.18E-43	CenC
SPAG4	3.96E-47	0.260633	0.286	0.01	7.21E-43	CenC
PITX2	2.00E-45	1.249837	0.929	0.112	3.65E-41	CenC
ANXA6	2.75E-45	0.747299	0.607	0.046	5.01E-41	CenC
TRIB2	6.15E-44	0.456789	0.464	0.028	1.12E-39	CenC

EFCAB4A	7.41E-44	0.335623	0.393	0.02	1.35E-39	CenC
VEGFA	3.97E-43	0.86139	0.786	0.085	7.23E-39	CenC
PCDH18	6.47E-43	0.326561	0.321	0.014	1.18E-38	CenC
LPHN1	1.15E-42	0.33115	0.357	0.017	2.09E-38	CenC
COL8A1	2.12E-42	1.281131	0.821	0.095	3.86E-38	CenC
METRN	7.26E-42	0.300862	0.286	0.011	1.32E-37	CenC
MYH10	1.65E-40	0.469229	0.5	0.035	3.00E-36	CenC
SEMA3E	3.20E-40	0.38055	0.393	0.021	5.82E-36	CenC
BMP3	5.15E-40	0.526958	0.571	0.046	9.37E-36	CenC
OBSL1	5.72E-40	0.633393	0.643	0.058	1.04E-35	CenC
TFAP2B	7.85E-40	1.082514	0.964	0.129	1.43E-35	CenC
SLC9A3R2	3.26E-39	0.597729	0.75	0.079	5.93E-35	CenC
HEG1	1.34E-38	0.506355	0.643	0.059	2.44E-34	CenC
SORL1	1.42E-38	0.676482	0.643	0.061	2.58E-34	CenC
TCEAL2	2.39E-38	0.312274	0.357	0.019	4.34E-34	CenC
SPON1	3.34E-38	0.549715	0.464	0.032	6.08E-34	CenC
RPRML	1.19E-36	0.940305	0.536	0.046	2.18E-32	CenC
KDELR3	4.34E-36	0.602005	0.607	0.057	7.91E-32	CenC
AGPHD1	4.41E-36	0.350439	0.464	0.034	8.03E-32	CenC
ERG	7.50E-36	0.420988	0.536	0.043	1.36E-31	CenC
HIST4H4	2.16E-35	0.321587	0.286	0.013	3.94E-31	CenC
AK4	3.76E-35	0.936905	0.857	0.128	6.84E-31	CenC
SEMA3C	4.23E-35	0.405597	0.536	0.044	7.70E-31	CenC
SERPINI1	4.79E-34	1.698395	0.857	0.137	8.72E-30	CenC
PTPRM	8.16E-34	0.426897	0.429	0.03	1.49E-29	CenC
CYYR1	1.87E-33	0.40646	0.393	0.026	3.40E-29	CenC
HAAO	7.61E-33	0.331265	0.393	0.026	1.39E-28	CenC
CD83	1.59E-32	0.685286	0.679	0.081	2.89E-28	CenC
GULP1	2.33E-32	0.405395	0.5	0.042	4.24E-28	CenC
SCARB1	2.62E-32	0.30212	0.286	0.015	4.77E-28	CenC
CYTL1	9.04E-32	0.730397	0.821	0.109	1.65E-27	CenC
ID4	2.52E-31	0.51125	0.643	0.074	4.58E-27	CenC
SNCA	8.20E-31	0.753718	0.429	0.034	1.49E-26	CenC
PCOLCE	9.30E-31	0.928079	0.857	0.136	1.69E-26	CenC
SLC20A2	9.31E-31	0.509516	0.607	0.067	1.69E-26	CenC
AMT	2.59E-30	0.282785	0.357	0.024	4.71E-26	CenC
SMIM10	2.97E-30	0.323136	0.429	0.033	5.41E-26	CenC
ITPKB	3.73E-30	0.285717	0.321	0.019	6.79E-26	CenC
GMPR	4.40E-30	0.439103	0.643	0.073	8.02E-26	CenC
ATP1B1	4.67E-30	1.352733	0.964	0.23	8.51E-26	CenC
ALDOC	4.78E-30	0.884192	0.786	0.119	8.71E-26	CenC
BNIP3	6.23E-30	0.308372	0.464	0.039	1.13E-25	CenC
TSPAN6	6.90E-30	1.897499	1	0.293	1.26E-25	CenC
SMOX	1.13E-29	0.289641	0.321	0.02	2.06E-25	CenC
AK125699	2.05E-29	0.581336	0.571	0.063	3.74E-25	CenC
NDNF	9.71E-29	0.684413	0.821	0.118	1.77E-24	CenC
EGLN3	2.81E-28	0.860642	0.75	0.115	5.12E-24	CenC
SLC16A3	5.76E-28	0.690919	0.857	0.142	1.05E-23	CenC

LAMB1	8.43E-28	0.655774	0.714	0.102	1.53E-23	CenC
SMARCD3	1.00E-27	0.258718	0.286	0.017	1.83E-23	CenC
AMIGO2	7.75E-27	0.486527	0.464	0.045	1.41E-22	CenC
NUDT4	1.11E-26	0.985392	0.929	0.208	2.03E-22	CenC
CHCHD10	1.18E-26	1.470709	0.964	0.283	2.15E-22	CenC
NRN1	2.36E-26	0.549774	0.679	0.095	4.30E-22	CenC
MAN1A1	2.64E-26	0.497319	0.429	0.04	4.81E-22	CenC
TCEAL7	3.76E-26	0.412686	0.357	0.027	6.84E-22	CenC
GPC4	4.03E-26	0.500033	0.571	0.067	7.33E-22	CenC
FGF2	2.03E-25	0.37439	0.357	0.028	3.70E-21	CenC
PFKP	2.45E-25	1.264354	0.929	0.267	4.46E-21	CenC
SULF2	3.54E-25	0.652606	0.643	0.094	6.44E-21	CenC
PTPRU	1.18E-24	0.332434	0.429	0.041	2.15E-20	CenC
PBXIP1	1.84E-24	1.109462	0.857	0.196	3.36E-20	CenC
MBD3	2.82E-24	0.36054	0.393	0.035	5.13E-20	CenC
SULT1A1	8.67E-24	0.514503	0.536	0.067	1.58E-19	CenC
LMX1B	2.00E-23	0.297272	0.357	0.03	3.64E-19	CenC
AK055712	2.70E-23	0.312241	0.357	0.03	4.92E-19	CenC
CAV1	2.92E-23	1.129196	1	0.263	5.32E-19	CenC
FAM65B	5.45E-23	0.328454	0.429	0.043	9.93E-19	CenC
TCEAL3	6.51E-23	0.881026	0.857	0.191	1.19E-18	CenC
MAP4K3	6.93E-23	0.411275	0.464	0.051	1.26E-18	CenC
TCF4	6.96E-23	0.713241	0.821	0.144	1.27E-18	CenC
BC073897	1.02E-22	0.669788	0.607	0.089	1.86E-18	CenC
B3GNT7	1.05E-22	0.774861	0.643	0.099	1.90E-18	CenC
MYL9	1.35E-22	0.633154	0.607	0.088	2.46E-18	CenC
MADD	1.59E-22	0.324852	0.357	0.032	2.90E-18	CenC
FOXC1	2.95E-21	0.593804	0.786	0.157	5.37E-17	CenC
IGFBP5	3.07E-21	0.482065	0.429	0.046	5.59E-17	CenC
FSTL1	3.41E-21	0.853204	0.714	0.132	6.21E-17	CenC
LOC728392	3.49E-21	0.476556	0.5	0.064	6.35E-17	CenC
APP	3.58E-21	1.781258	1	0.567	6.52E-17	CenC
TMEM64	4.20E-21	0.562355	0.536	0.075	7.66E-17	CenC
NIN	5.41E-21	0.434816	0.5	0.066	9.85E-17	CenC
SLC5A3	5.85E-21	0.278547	0.393	0.04	1.07E-16	CenC
PRKAR1A	1.23E-20	1.42195	1	0.473	2.23E-16	CenC
CDON	1.45E-20	0.387981	0.429	0.048	2.64E-16	CenC
COX7A1	2.96E-20	1.409498	1	0.483	5.38E-16	CenC
PPAP2B	3.20E-20	0.619414	0.929	0.2	5.82E-16	CenC
PRUNE2	3.91E-20	0.297957	0.393	0.041	7.11E-16	CenC
EPB41L2	4.21E-20	0.478173	0.5	0.067	7.66E-16	CenC
DPYSL3	8.55E-20	0.649649	0.607	0.099	1.56E-15	CenC
AKR1C1	9.48E-20	1.356775	0.857	0.225	1.73E-15	CenC
RHOQ	1.31E-19	0.310199	0.429	0.05	2.39E-15	CenC
LOC100129195	1.35E-19	0.584981	0.714	0.14	2.47E-15	CenC
MIF	2.09E-19	1.16718	1	0.993	3.80E-15	CenC
UQCRB	2.69E-19	1.156594	1	0.968	4.90E-15	CenC
COX4I1	3.04E-19	1.143537	1	0.978	5.54E-15	CenC

GLI3	3.71E-19	0.304701	0.357	0.037	6.76E-15	CenC
UQCRH	4.27E-19	1.287132	1	0.913	7.77E-15	CenC
EMX2	4.62E-19	0.900853	0.929	0.271	8.42E-15	CenC
CCDC144B	4.68E-19	0.285724	0.286	0.024	8.53E-15	CenC
RBP1	4.92E-19	0.811454	0.643	0.121	8.95E-15	CenC
KIAA0355	8.25E-19	0.39266	0.393	0.045	1.50E-14	CenC
FLJ46906	1.05E-18	0.502984	0.464	0.063	1.91E-14	CenC
ENSG0000021008	1.37E-18	1.395657	1	1	2.50E-14	CenC
WNT5B	1.50E-18	0.300672	0.393	0.045	2.73E-14	CenC
PTGDS	1.70E-18	1.906264	1	0.441	3.09E-14	CenC
SLC25A4	2.05E-18	0.982446	1	0.451	3.74E-14	CenC
C1orf54	2.06E-18	0.449235	0.429	0.055	3.75E-14	CenC
COX6C	2.10E-18	0.897358	1	0.967	3.82E-14	CenC
TPI1	2.20E-18	0.90003	1	0.967	4.00E-14	CenC
NDUFA1	3.21E-18	0.971979	1	0.928	5.85E-14	CenC
GAPDH	3.68E-18	1.160291	1	0.998	6.70E-14	CenC
SMAD7	4.07E-18	0.278004	0.286	0.025	7.42E-14	CenC
COX5B	4.24E-18	1.085354	1	0.963	7.72E-14	CenC
LURAP1L	4.38E-18	0.318321	0.393	0.045	7.98E-14	CenC
ZFHX3	5.30E-18	0.608329	0.679	0.138	9.65E-14	CenC
ITGB5	6.32E-18	0.430738	0.607	0.104	1.15E-13	CenC
DDB1	6.71E-18	0.898807	0.857	0.253	1.22E-13	CenC
MAGI2-AS3	7.68E-18	0.407023	0.5	0.073	1.40E-13	CenC
PDLIM2	8.18E-18	0.810656	0.786	0.202	1.49E-13	CenC
SMIM3	8.44E-18	0.687128	0.786	0.184	1.54E-13	CenC
FAM195A	1.01E-17	0.627953	0.786	0.203	1.84E-13	CenC
PRDX2	1.63E-17	0.979271	1	0.91	2.96E-13	CenC
NDUFB9	2.74E-17	0.966166	1	0.855	4.99E-13	CenC
APBB2	3.03E-17	0.334226	0.286	0.027	5.51E-13	CenC
NDUFA4	3.17E-17	0.850927	1	0.961	5.77E-13	CenC
BEX2	4.89E-17	0.833413	0.929	0.339	8.90E-13	CenC
ATP5L	5.45E-17	0.827608	1	0.981	9.92E-13	CenC
AHCYL1	6.73E-17	0.961691	0.893	0.339	1.22E-12	CenC
COX7C	7.39E-17	0.852109	1	0.985	1.34E-12	CenC
FBXO2	8.95E-17	0.607473	0.786	0.213	1.63E-12	CenC
CALD1	1.05E-16	0.892949	0.929	0.288	1.92E-12	CenC
MAGI3	1.20E-16	0.40293	0.464	0.07	2.18E-12	CenC
GLS	1.21E-16	0.648293	0.857	0.237	2.20E-12	CenC
CLDN11	1.24E-16	0.397053	0.571	0.093	2.26E-12	CenC
TMEM160	1.28E-16	1.085153	0.964	0.529	2.33E-12	CenC
HSD17B14	1.29E-16	0.303169	0.321	0.034	2.34E-12	CenC
HMGXB3	1.40E-16	0.32461	0.393	0.051	2.55E-12	CenC
GSTM3	1.40E-16	0.703784	0.857	0.23	2.55E-12	CenC
PITX1	1.44E-16	0.562819	0.607	0.112	2.62E-12	CenC
GPI	1.75E-16	0.974459	0.929	0.392	3.18E-12	CenC
NGFRAP1	1.86E-16	1.104607	1	0.763	3.39E-12	CenC
DMKN	2.21E-16	0.465964	0.607	0.116	4.02E-12	CenC
ARL5A	2.53E-16	1.023368	0.964	0.52	4.60E-12	CenC

IER3	2.74E-16	1.311322	1	0.832	4.99E-12	CenC
ENSG00000198886	4.55E-16	0.865171	1	0.998	8.28E-12	CenC
CDC42EP3	4.81E-16	0.486287	0.679	0.138	8.75E-12	CenC
NRIP3	5.44E-16	0.256603	0.286	0.029	9.90E-12	CenC
LOC143666	5.70E-16	0.455241	0.357	0.045	1.04E-11	CenC
PDK1	6.85E-16	0.316839	0.393	0.052	1.25E-11	CenC
ATP5G3	8.54E-16	0.951211	1	0.902	1.56E-11	CenC
A1BG	8.64E-16	0.344914	0.5	0.078	1.57E-11	CenC
ATP5J	8.91E-16	0.797923	1	0.94	1.62E-11	CenC
SMC2	1.03E-15	0.598173	0.607	0.124	1.88E-11	CenC
ARHGEF37	1.24E-15	0.278281	0.286	0.029	2.26E-11	CenC
APOL2	1.39E-15	0.268188	0.393	0.052	2.53E-11	CenC
NDUFS3	1.43E-15	0.743172	1	0.591	2.61E-11	CenC
COL12A1	1.69E-15	0.443218	0.857	0.192	3.08E-11	CenC
CRIM1	1.72E-15	0.754384	0.821	0.254	3.13E-11	CenC
DDIT4	1.78E-15	1.485711	0.964	0.463	3.25E-11	CenC
RPL21	1.91E-15	0.714745	1	0.999	3.47E-11	CenC
EVA1B	1.91E-15	0.263505	0.357	0.044	3.48E-11	CenC
ENSG0000021145	1.99E-15	1.197272	1	0.997	3.62E-11	CenC
COX6B1	2.07E-15	0.877383	1	0.949	3.76E-11	CenC
ZMAT3	2.27E-15	0.490956	0.536	0.098	4.13E-11	CenC
RCAN1	2.77E-15	0.557195	0.393	0.058	5.04E-11	CenC
PDK4	3.06E-15	0.389499	0.429	0.062	5.58E-11	CenC
POLR2L	3.12E-15	0.733541	1	0.966	5.68E-11	CenC
RBMS3	3.36E-15	0.282207	0.357	0.044	6.12E-11	CenC
IFI6	3.71E-15	0.943109	0.786	0.203	6.76E-11	CenC
ATP5O	5.06E-15	0.945273	1	0.912	9.22E-11	CenC
ABLIM1	5.09E-15	0.493628	0.571	0.115	9.26E-11	CenC
ENSG0000019871	5.30E-15	0.847531	1	0.997	9.65E-11	CenC
COX7B	5.73E-15	0.803891	1	0.937	1.04E-10	CenC
ATP1A1	8.30E-15	1.000741	0.964	0.589	1.51E-10	CenC
GLIS1	8.44E-15	0.292969	0.321	0.038	1.54E-10	CenC
ATP5B	8.51E-15	0.79055	1	0.917	1.55E-10	CenC
COL1A2	9.01E-15	0.387242	0.75	0.158	1.64E-10	CenC
STON2	9.99E-15	0.496943	0.5	0.091	1.82E-10	CenC
SETD3	1.37E-14	0.550473	0.679	0.174	2.49E-10	CenC
IGFBP2	1.43E-14	1.02041	0.964	0.555	2.61E-10	CenC
WDR54	1.58E-14	0.544954	0.75	0.203	2.87E-10	CenC
RERG	1.60E-14	0.493572	0.786	0.201	2.91E-10	CenC
CDKN1C	1.65E-14	0.516958	0.679	0.16	3.01E-10	CenC
PKIG	1.82E-14	0.546344	0.714	0.175	3.32E-10	CenC
ENSG0000021290	2.96E-14	0.995781	1	0.839	5.39E-10	CenC
LRIG1	3.19E-14	0.527767	0.5	0.097	5.81E-10	CenC
NDUFA13	3.49E-14	0.756579	1	0.936	6.36E-10	CenC
ATP5I	3.67E-14	0.841022	1	0.919	6.68E-10	CenC
AQP1	4.53E-14	0.301025	0.821	0.184	8.25E-10	CenC
FADS1	5.04E-14	0.258293	0.393	0.057	9.17E-10	CenC
TMEM139	5.07E-14	0.299907	0.286	0.033	9.23E-10	CenC

PIK3R1	5.62E-14	0.452787	0.571	0.117	1.02E-09	CenC
HPS5	5.83E-14	0.45083	0.5	0.096	1.06E-09	CenC
NDUFB2	5.86E-14	0.927159	1	0.864	1.07E-09	CenC
DYNLRB2	5.87E-14	0.333844	0.393	0.058	1.07E-09	CenC
NDUFB8	6.18E-14	0.961459	1	0.84	1.12E-09	CenC
TSC22D1	7.41E-14	1.163186	1	0.587	1.35E-09	CenC
SNRPN	7.47E-14	0.778507	0.964	0.583	1.36E-09	CenC
CCK	8.16E-14	1.468991	0.536	0.118	1.49E-09	CenC
MORN2	9.60E-14	0.581258	0.75	0.226	1.75E-09	CenC
ENSG0000019878[illegible]	9.93E-14	0.93171	1	0.975	1.81E-09	CenC
TES	1.09E-13	0.743555	0.75	0.235	1.98E-09	CenC
ARMCX3	1.75E-13	0.378606	0.571	0.113	3.18E-09	CenC
NDUFB7	1.79E-13	0.794677	1	0.851	3.26E-09	CenC
ATP5A1	2.20E-13	0.818527	1	0.829	4.01E-09	CenC
FAM162A	2.44E-13	0.772597	1	0.749	4.44E-09	CenC
STK35	2.70E-13	0.581862	0.821	0.273	4.92E-09	CenC
VIM	2.75E-13	0.61961	1	0.33	5.01E-09	CenC
GNAS	3.44E-13	0.899708	1	0.659	6.26E-09	CenC
EIF3K	3.55E-13	0.750847	1	0.897	6.46E-09	CenC
AKR1B1	3.86E-13	0.825121	0.964	0.467	7.03E-09	CenC
COX7A2	3.88E-13	0.694628	1	0.976	7.07E-09	CenC
GCSH	4.51E-13	0.721001	1	0.479	8.21E-09	CenC
SEC14L1	4.72E-13	0.404668	0.607	0.138	8.59E-09	CenC
ADD3	4.78E-13	0.597546	0.679	0.185	8.71E-09	CenC
CTSZ	5.24E-13	0.424973	0.607	0.138	9.54E-09	CenC
CXADR	5.88E-13	0.828752	0.929	0.555	1.07E-08	CenC
RPS27A	6.67E-13	0.517603	1	0.997	1.21E-08	CenC
TIMP2	8.64E-13	0.320236	0.679	0.145	1.57E-08	CenC
SLC25A6	9.41E-13	0.783924	1	0.897	1.71E-08	CenC
SMYD3	1.14E-12	0.402099	0.536	0.117	2.08E-08	CenC
PFKM	1.26E-12	0.398484	0.607	0.141	2.29E-08	CenC
HEXIM1	1.47E-12	0.676382	0.929	0.407	2.67E-08	CenC
RHOBTB3	1.69E-12	0.655671	0.714	0.21	3.09E-08	CenC
AHDC1	1.71E-12	0.402537	0.464	0.09	3.11E-08	CenC
PHPT1	1.73E-12	0.868736	1	0.771	3.15E-08	CenC
PGK1	1.77E-12	0.721873	1	0.885	3.22E-08	CenC
KDM6B	2.07E-12	0.414432	0.571	0.127	3.77E-08	CenC
AFAP1L2	2.12E-12	0.339554	0.286	0.037	3.86E-08	CenC
INSIG1	2.16E-12	0.70507	0.714	0.227	3.93E-08	CenC
PDPN	2.41E-12	0.342385	0.5	0.099	4.39E-08	CenC
DPCD	2.78E-12	0.462692	0.536	0.123	5.06E-08	CenC
SLC16A11	3.15E-12	0.320648	0.429	0.076	5.73E-08	CenC
CYC1	3.96E-12	0.652161	1	0.717	7.21E-08	CenC
IFITM2	4.27E-12	0.401215	0.75	0.186	7.77E-08	CenC
GMDS	4.35E-12	0.732075	0.857	0.375	7.93E-08	CenC
SELM	4.39E-12	0.684759	0.929	0.342	7.99E-08	CenC
WDR74	4.56E-12	1.092045	0.821	0.37	8.30E-08	CenC
ENSG0000019880[illegible]	4.89E-12	0.600997	1	0.998	8.91E-08	CenC

RPS27L	6.20E-12	0.844056	1	0.845	1.13E-07	CenC
SNHG6	8.92E-12	0.763312	1	0.76	1.62E-07	CenC
H2AFJ	1.04E-11	0.863834	0.929	0.554	1.89E-07	CenC
TIFA	1.09E-11	0.321421	0.536	0.118	1.98E-07	CenC
RPL34	1.15E-11	0.489393	1	1	2.10E-07	CenC
LINC00152	1.40E-11	0.371032	0.536	0.112	2.55E-07	CenC
FN3K	1.49E-11	0.298974	0.357	0.059	2.72E-07	CenC
CTGF	1.63E-11	0.82214	0.714	0.2	2.97E-07	CenC
COL4A5	1.82E-11	0.285824	0.357	0.058	3.32E-07	CenC
HUWE1	1.93E-11	0.289283	0.393	0.069	3.52E-07	CenC
NTN4	2.35E-11	0.365977	0.5	0.101	4.28E-07	CenC
LOC100506421	2.54E-11	0.375489	0.607	0.137	4.63E-07	CenC
VPS41	2.87E-11	0.383776	0.5	0.111	5.23E-07	CenC
ID3	2.92E-11	0.83641	1	0.76	5.32E-07	CenC
KANSL3	2.93E-11	0.250346	0.321	0.049	5.34E-07	CenC
RBMS1	2.94E-11	0.397606	0.643	0.175	5.36E-07	CenC
ENO1	3.02E-11	0.600027	1	0.994	5.50E-07	CenC
SLC22A4	3.18E-11	0.267994	0.357	0.058	5.78E-07	CenC
RASIP1	3.55E-11	0.287374	0.321	0.05	6.46E-07	CenC
IL13RA1	3.60E-11	0.337008	0.536	0.123	6.55E-07	CenC
PDLIM4	4.93E-11	0.588736	0.75	0.254	8.98E-07	CenC
MPC2	5.38E-11	0.590828	1	0.682	9.79E-07	CenC
ENSG0000021019	5.48E-11	0.834694	0.75	0.273	9.98E-07	CenC
TJP1	5.64E-11	0.384739	0.643	0.172	1.03E-06	CenC
USMG5	5.75E-11	0.664888	1	0.95	1.05E-06	CenC
DNAJC1	5.78E-11	0.578706	0.714	0.246	1.05E-06	CenC
EFNA5	5.79E-11	0.413305	0.464	0.101	1.05E-06	CenC
NDUFB1	5.81E-11	0.592138	1	0.877	1.06E-06	CenC
SMIM1	6.32E-11	0.263478	0.393	0.072	1.15E-06	CenC
PGAM1	7.43E-11	0.664469	1	0.883	1.35E-06	CenC
OSTC	7.47E-11	0.919805	0.929	0.636	1.36E-06	CenC
NAV1	7.72E-11	0.289344	0.321	0.051	1.41E-06	CenC
UQCRQ	7.78E-11	0.672107	1	0.95	1.42E-06	CenC
ENSG0000019876	7.86E-11	0.760718	1	0.983	1.43E-06	CenC
SRP14-AS1	7.97E-11	0.253326	0.357	0.061	1.45E-06	CenC
RPL31	8.24E-11	0.433995	1	0.998	1.50E-06	CenC
RAB11FIP2	9.54E-11	0.344029	0.5	0.112	1.74E-06	CenC
SNHG10	9.89E-11	0.264898	0.393	0.072	1.80E-06	CenC
CCL2	1.00E-10	0.80956	0.464	0.101	1.83E-06	CenC
EEF1A1	1.02E-10	0.476404	1	1	1.85E-06	CenC
PTPRN2	1.14E-10	0.315749	0.393	0.076	2.08E-06	CenC
DNPH1	1.22E-10	0.576498	0.786	0.327	2.22E-06	CenC
NDUFS7	1.22E-10	0.709637	0.964	0.604	2.23E-06	CenC
ERRFI1	1.31E-10	0.728415	0.75	0.3	2.39E-06	CenC
DDT	1.53E-10	0.748353	0.964	0.716	2.79E-06	CenC
SLC35E1	1.62E-10	0.304116	0.393	0.075	2.95E-06	CenC
HYAL1	1.70E-10	0.307666	0.321	0.053	3.09E-06	CenC
NUPL1	1.76E-10	0.280504	0.429	0.088	3.20E-06	CenC

UQCR11	1.76E-10	0.543367	1	0.965	3.20E-06	CenC
TIMP3	1.89E-10	0.71359	0.857	0.346	3.44E-06	CenC
ID2	1.92E-10	0.721482	0.964	0.559	3.50E-06	CenC
SLC20A1	1.94E-10	0.589325	0.964	0.429	3.53E-06	CenC
ZFHX4	2.02E-10	0.412612	0.429	0.088	3.68E-06	CenC
PHACTR2	2.27E-10	0.532568	0.75	0.267	4.13E-06	CenC
WWTR1	2.59E-10	0.387301	0.536	0.138	4.72E-06	CenC
ESYT1	2.62E-10	0.370808	0.357	0.064	4.77E-06	CenC
SDHA	2.69E-10	0.651744	0.821	0.359	4.90E-06	CenC
EFEMP1	2.91E-10	0.736954	0.786	0.314	5.30E-06	CenC
NDUFB10	3.05E-10	0.550918	1	0.811	5.55E-06	CenC
MYH9	3.29E-10	0.620967	0.679	0.244	5.99E-06	CenC
FOXO1	3.32E-10	0.290272	0.393	0.077	6.05E-06	CenC
MRPL21	3.59E-10	0.597483	0.929	0.472	6.55E-06	CenC
P4HA1	3.63E-10	0.411744	0.429	0.093	6.61E-06	CenC
TOMM7	3.83E-10	0.532191	1	0.969	6.97E-06	CenC
TWIST1	4.50E-10	0.260075	0.357	0.063	8.20E-06	CenC
B3GNT1	4.57E-10	0.428432	0.464	0.107	8.33E-06	CenC
ENSG0000019888	4.89E-10	0.668978	1	0.96	8.90E-06	CenC
NDUFB3	5.27E-10	0.59433	1	0.825	9.60E-06	CenC
TLE4	5.41E-10	0.40808	0.714	0.223	9.85E-06	CenC
NDUFAB1	5.53E-10	0.651651	0.964	0.803	1.01E-05	CenC
ATN1	6.15E-10	0.352117	0.536	0.134	1.12E-05	CenC
ATP6V0E2	6.57E-10	0.316055	0.429	0.093	1.20E-05	CenC
ATP5D	7.05E-10	0.581887	0.964	0.803	1.28E-05	CenC
COX17	8.43E-10	0.56755	0.964	0.592	1.53E-05	CenC
COX6A1	8.91E-10	0.463083	1	0.978	1.62E-05	CenC
CLEC11A	9.13E-10	0.595723	0.714	0.266	1.66E-05	CenC
SPRY2	9.48E-10	0.429398	0.429	0.096	1.73E-05	CenC
FBXL17	1.02E-09	0.265743	0.286	0.045	1.85E-05	CenC
MRPL41	1.02E-09	0.692685	0.964	0.674	1.87E-05	CenC
HINT1	1.04E-09	0.529371	1	0.973	1.89E-05	CenC
MINOS1	1.10E-09	0.561119	1	0.891	2.00E-05	CenC
QDPR	1.25E-09	0.45121	0.714	0.245	2.28E-05	CenC
C1orf122	1.30E-09	0.499174	0.821	0.339	2.37E-05	CenC
NDUFA6	1.36E-09	0.596648	1	0.813	2.48E-05	CenC
ALDH7A1	1.41E-09	0.707133	0.964	0.607	2.57E-05	CenC
C21orf33	1.46E-09	0.56601	0.857	0.391	2.66E-05	CenC
SERPINB6	1.61E-09	0.441489	0.75	0.268	2.94E-05	CenC
C14orf2	1.66E-09	0.524284	1	0.953	3.03E-05	CenC
FAM229B	1.68E-09	0.469619	0.571	0.163	3.07E-05	CenC
LDHB	1.71E-09	0.615289	1	0.838	3.12E-05	CenC
DPYSL2	1.83E-09	0.334584	0.536	0.134	3.34E-05	CenC
CPEB4	1.90E-09	0.466731	0.536	0.145	3.46E-05	CenC
CNN3	1.93E-09	0.541639	0.857	0.361	3.52E-05	CenC
ENSG0000019872	2.13E-09	0.542449	1	0.993	3.88E-05	CenC
CTNNAL1	2.56E-09	0.529945	0.679	0.227	4.66E-05	CenC
ENSG0000019889	2.64E-09	0.606558	1	0.988	4.81E-05	CenC

RPS3	3.05E-09	0.432133	1	0.996	5.56E-05	CenC
LOC338799	3.18E-09	0.324929	0.393	0.083	5.80E-05	CenC
VDAC3	3.23E-09	0.645455	0.893	0.585	5.88E-05	CenC
CSTF3	3.33E-09	0.404826	0.679	0.217	6.06E-05	CenC
RPL41	4.04E-09	0.332842	1	1	7.35E-05	CenC
TMEM129	4.06E-09	0.371678	0.464	0.117	7.39E-05	CenC
MAF	4.33E-09	0.288573	0.393	0.086	7.89E-05	CenC
ATP5G1	5.15E-09	0.798905	0.964	0.784	9.38E-05	CenC
MZT2B	5.21E-09	0.583016	0.964	0.701	9.49E-05	CenC
CA11	5.28E-09	0.421894	0.5	0.132	9.61E-05	CenC
COL5A2	6.10E-09	0.303334	0.464	0.108	0.000111	CenC
HYOU1	6.34E-09	0.268692	0.357	0.073	0.000115	CenC
ESRRA	6.47E-09	0.335854	0.464	0.118	0.000118	CenC
RPS8	7.37E-09	0.437774	1	0.999	0.000134	CenC
CIRBP	7.53E-09	0.491089	1	0.981	0.000137	CenC
BCAR3	7.57E-09	0.252985	0.357	0.073	0.000138	CenC
ANKH	7.64E-09	0.428043	0.536	0.143	0.000139	CenC
CADM1	8.27E-09	0.527607	0.607	0.197	0.000151	CenC
CYCS	8.39E-09	0.630645	1	0.87	0.000153	CenC
SLC2A1-AS1	8.81E-09	0.27068	0.357	0.075	0.00016	CenC
DL491896	8.89E-09	0.380336	0.679	0.229	0.000162	CenC
HILPDA	9.06E-09	0.792928	0.464	0.128	0.000165	CenC
IRF2BPL	9.08E-09	0.539601	0.75	0.308	0.000165	CenC
TMEM141	9.56E-09	0.578131	0.893	0.487	0.000174	CenC
NDUFS4	1.28E-08	0.544951	1	0.757	0.000233	CenC
VAMP2	1.29E-08	0.600494	1	0.751	0.000236	CenC
UQCRC2	1.30E-08	0.612412	0.929	0.647	0.000237	CenC
MGARP	1.33E-08	0.641846	1	0.827	0.000243	CenC
HSPD1	1.38E-08	0.53393	0.929	0.69	0.000252	CenC
C4orf48	1.48E-08	0.389636	0.536	0.155	0.000269	CenC
RPL7	1.48E-08	0.363968	1	0.999	0.000269	CenC
AKAP13	1.52E-08	0.410151	0.571	0.179	0.000277	CenC
ANKRD9	1.54E-08	0.274004	0.429	0.102	0.00028	CenC
ABHD14A	1.57E-08	0.340448	0.464	0.121	0.000285	CenC
PGP	1.60E-08	0.389207	0.5	0.15	0.000292	CenC
SNHG8	1.68E-08	0.568636	0.964	0.7	0.000305	CenC
LAGE3	1.82E-08	0.485279	0.857	0.456	0.000332	CenC
MYO1B	1.93E-08	0.357593	0.464	0.119	0.000352	CenC
WDR61	1.99E-08	0.582623	0.929	0.624	0.000362	CenC
CCDC85B	2.14E-08	0.633087	0.857	0.551	0.000389	CenC
EMX2OS	2.28E-08	0.3104	0.429	0.106	0.000416	CenC
TMEM106C	2.39E-08	0.403186	0.714	0.267	0.000434	CenC
SLC25A5	2.78E-08	0.596653	1	0.874	0.000506	CenC
ST13	2.82E-08	0.531966	0.964	0.852	0.000513	CenC
CCDC151	2.96E-08	0.341539	0.357	0.078	0.000539	CenC
TCEA2	2.99E-08	0.435631	0.714	0.276	0.000544	CenC
CBX5	3.01E-08	0.369294	0.536	0.161	0.000547	CenC
QKI	3.08E-08	0.298032	0.464	0.12	0.000561	CenC

UROD	3.17E-08	0.433441	0.893	0.446	0.000578	CenC
PBX1	3.21E-08	0.478357	0.536	0.171	0.000584	CenC
PIAS2	3.33E-08	0.293035	0.357	0.076	0.000607	CenC
LINC00685	3.72E-08	0.298067	0.5	0.142	0.000677	CenC
RPL38	3.96E-08	0.398679	1	0.989	0.000721	CenC
SMIM4	4.03E-08	0.544124	0.714	0.334	0.000734	CenC
ARL2	4.27E-08	0.483867	0.857	0.463	0.000777	CenC
NUMA1	4.68E-08	0.310797	0.607	0.186	0.000852	CenC
RABL5	4.94E-08	0.37447	0.429	0.114	0.0009	CenC
NDUFB5	5.48E-08	0.509644	0.929	0.69	0.000998	CenC
SNRNP200	5.54E-08	0.304554	0.536	0.161	0.001008	CenC
NDUFAF4	5.57E-08	0.426282	0.714	0.28	0.001013	CenC
HIST1H4C	5.68E-08	0.591582	0.679	0.291	0.001033	CenC
LOC541471	5.68E-08	0.343544	0.571	0.167	0.001035	CenC
LANCL1	5.80E-08	0.292645	0.357	0.082	0.001056	CenC
VOPP1	6.15E-08	0.393882	0.429	0.112	0.001119	CenC
MPC1	6.77E-08	0.48432	0.893	0.559	0.001232	CenC
PRCP	7.22E-08	0.511387	0.714	0.311	0.001315	CenC
DPP7	7.33E-08	0.546075	0.821	0.475	0.001335	CenC
TCEAL4	7.68E-08	0.589632	0.964	0.625	0.001398	CenC
REPIN1	8.13E-08	0.288294	0.464	0.126	0.00148	CenC
ARID5B	8.27E-08	0.473561	0.893	0.429	0.001506	CenC
AKR1C2	8.75E-08	0.482843	0.75	0.31	0.001593	CenC
GLRX5	8.76E-08	0.546327	0.75	0.368	0.001595	CenC
USP11	9.26E-08	0.382333	0.5	0.153	0.001687	CenC
DQ570096	1.07E-07	0.272015	0.321	0.068	0.001941	CenC
RPL26	1.15E-07	0.324005	1	0.999	0.002096	CenC
LRPPRC	1.16E-07	0.28239	0.393	0.097	0.002112	CenC
TAB2	1.18E-07	0.250739	0.357	0.082	0.00214	CenC
COX20	1.20E-07	0.532483	0.929	0.543	0.002193	CenC
HIPK3	1.23E-07	0.286712	0.357	0.084	0.002236	CenC
LDLR	1.32E-07	0.262966	0.429	0.109	0.002399	CenC
NXPH4	1.60E-07	0.267419	0.357	0.083	0.002914	CenC
RPL8	1.64E-07	0.311974	1	0.998	0.002985	CenC
SNAPC1	1.66E-07	0.256707	0.536	0.16	0.003014	CenC
TMEM55A	1.70E-07	0.262245	0.5	0.138	0.003099	CenC
DANCR	2.12E-07	0.633589	0.929	0.654	0.003859	CenC
DDIT3	2.15E-07	0.652384	0.893	0.549	0.003909	CenC
LINC00657	2.19E-07	0.482047	0.857	0.52	0.003981	CenC
LOC654342	2.21E-07	0.328535	0.643	0.226	0.00403	CenC
FXN	2.30E-07	0.314601	0.393	0.105	0.004185	CenC
SLC2A1	2.50E-07	0.501895	1	0.86	0.004548	CenC
SERPINF1	2.53E-07	0.316308	0.929	0.525	0.004609	CenC
ZNF664	2.60E-07	0.259517	0.393	0.102	0.004743	CenC
CLTC	2.72E-07	0.274996	0.536	0.168	0.004951	CenC
ST20	3.00E-07	0.290534	0.321	0.075	0.005462	CenC
BNIP3L	3.04E-07	0.560501	0.893	0.621	0.005542	CenC
LARP6	3.11E-07	0.279813	0.5	0.139	0.005671	CenC

SLC12A4	3.16E-07	0.286614	0.393	0.099	0.00575	CenC
HIBADH	3.37E-07	0.403913	0.75	0.292	0.006134	CenC
NDUFS8	3.71E-07	0.595197	0.929	0.688	0.006759	CenC
LGALS3BP	3.73E-07	0.460458	0.857	0.505	0.00679	CenC
SMPD1	3.75E-07	0.276404	0.536	0.168	0.006837	CenC
AGPAT1	3.95E-07	0.266993	0.5	0.149	0.007199	CenC
FAM210A	4.01E-07	0.273387	0.429	0.122	0.007297	CenC
MARCKSL1	4.05E-07	0.49116	0.643	0.249	0.007379	CenC
PTK7	4.09E-07	0.258529	0.393	0.104	0.007454	CenC
FGFR1	4.22E-07	0.309298	0.464	0.126	0.007685	CenC
VDAC2	4.69E-07	0.39456	1	0.886	0.008541	CenC
HPCAL1	4.81E-07	0.326062	0.464	0.14	0.008765	CenC
C17orf89	4.82E-07	0.442882	0.893	0.537	0.00878	CenC
CDKN1B	5.02E-07	0.324498	0.607	0.215	0.009132	CenC
HINT2	5.43E-07	0.546247	0.893	0.557	0.00988	CenC
ACAP1	5.52E-07	0.302363	0.321	0.077	0.010044	CenC
COX5A	6.57E-07	0.561706	0.929	0.862	0.011957	CenC
MRPS6	6.62E-07	0.652065	0.821	0.588	0.012046	CenC
NDUFS6	6.97E-07	0.516932	0.929	0.752	0.012694	CenC
HIST2H2AC	7.05E-07	0.287257	0.429	0.122	0.01283	CenC
SEPT8	7.14E-07	0.31904	0.321	0.078	0.013001	CenC
HIF1A	7.15E-07	0.275045	0.5	0.16	0.013023	CenC
WBP5	7.23E-07	0.457015	0.857	0.482	0.013167	CenC
BCL6	7.54E-07	0.259534	0.286	0.062	0.013734	CenC
CCDC107	7.55E-07	0.379587	0.5	0.161	0.013752	CenC
NFE2L1	7.76E-07	0.4052	0.679	0.303	0.014138	CenC
RPL17	8.10E-07	0.37587	1	0.985	0.014754	CenC
MYL6	8.40E-07	0.320057	1	0.988	0.015293	CenC
RPL3	8.83E-07	0.335406	1	0.999	0.016074	CenC
KGFLP2	9.39E-07	0.419204	0.429	0.131	0.017103	CenC
DUSP2	1.01E-06	0.415914	0.536	0.179	0.018322	CenC
S100A13	1.05E-06	0.428858	0.964	0.615	0.019163	CenC
NDUFA3	1.07E-06	0.518981	0.821	0.607	0.019443	CenC
THBS1	1.18E-06	0.281112	0.536	0.176	0.021462	CenC
RPSA	1.23E-06	0.493667	0.964	0.871	0.022325	CenC
TRPT1	1.27E-06	0.391939	0.536	0.197	0.023138	CenC
PRADC1	1.28E-06	0.372924	0.571	0.215	0.023392	CenC
CCDC104	1.36E-06	0.46165	0.607	0.272	0.02485	CenC
NUPR1	1.37E-06	1.332221	0.929	0.689	0.024992	CenC
SYNGR1	1.41E-06	0.35145	0.679	0.262	0.025699	CenC
CCDC23	1.43E-06	0.499279	0.643	0.291	0.025947	CenC
ENSG0000019884(	1.44E-06	0.515635	1	0.883	0.026201	CenC
TIMM10	1.49E-06	0.4264	0.786	0.425	0.027106	CenC
CD81	1.50E-06	0.451039	0.571	0.229	0.027312	CenC
TCEB2	1.50E-06	0.392906	1	0.961	0.027395	CenC
AMZ2P1	1.71E-06	0.308692	0.393	0.114	0.031184	CenC
UQCRHL	1.87E-06	0.370887	0.464	0.161	0.033988	CenC
CUTA	1.90E-06	0.44072	1	0.864	0.034601	CenC

NDUFA5	1.96E-06	0.45114	0.929	0.754	0.035767	CenC
CHCHD2	1.97E-06	0.331941	1	0.982	0.035937	CenC
ATPAF1	2.02E-06	0.459177	0.714	0.346	0.036768	CenC
C9orf3	2.05E-06	0.416416	0.714	0.324	0.037315	CenC
GSTM4	2.20E-06	0.426022	0.536	0.214	0.039998	CenC
MZT2A	2.22E-06	0.479337	0.714	0.399	0.040403	CenC
CTSL1	2.39E-06	0.725823	0.786	0.494	0.043442	CenC
SRP14	2.39E-06	0.320695	1	0.991	0.043603	CenC
EPB41L4A-AS1	2.40E-06	0.5063	0.821	0.525	0.043667	CenC
SAV1	2.44E-06	0.263844	0.321	0.081	0.04443	CenC
C19orf70	2.66E-06	0.477244	0.964	0.626	0.048408	CenC
NBR1	2.71E-06	0.311028	0.571	0.212	0.04943	CenC
PGM1	2.76E-06	0.42501	0.607	0.261	0.050286	CenC
SCOC	2.77E-06	0.384559	0.929	0.521	0.050351	CenC
SIGIRR	2.77E-06	0.474836	0.679	0.295	0.050404	CenC
RABGGTB	2.83E-06	0.497028	0.714	0.365	0.051527	CenC
HSP90AB1	3.05E-06	0.38657	1	0.963	0.055572	CenC
C6orf48	3.11E-06	0.534898	0.893	0.651	0.056637	CenC
SEC22C	3.16E-06	0.277624	0.5	0.164	0.057621	CenC
IRF1	3.21E-06	0.361162	0.786	0.377	0.058445	CenC
DNM2	3.48E-06	0.354954	0.464	0.161	0.063383	CenC
COX14	3.73E-06	0.438068	0.929	0.759	0.067967	CenC
GNB5	3.76E-06	0.375759	0.5	0.184	0.068547	CenC
C17orf76-AS1	3.79E-06	0.405053	1	0.964	0.068954	CenC
IL6ST	3.80E-06	0.253881	0.5	0.165	0.069116	CenC
HNRNPA1	3.86E-06	0.391795	1	0.97	0.070262	CenC
ZNF770	3.87E-06	0.306244	0.5	0.174	0.070395	CenC
TXN2	3.93E-06	0.361578	0.893	0.563	0.071484	CenC
EXT2	3.96E-06	0.271155	0.393	0.114	0.072052	CenC
ATP5H	4.44E-06	0.433738	0.964	0.854	0.080897	CenC
NDUFB11	5.05E-06	0.415415	0.964	0.784	0.09197	CenC
PTGS2	5.09E-06	0.671188	0.393	0.122	0.092686	CenC
MTHFD2L	5.84E-06	0.276696	0.571	0.223	0.106287	CenC
CEBPD	5.86E-06	0.52854	0.893	0.602	0.106618	CenC
CKS2	6.16E-06	0.496373	0.679	0.337	0.112173	CenC
RPL22	6.94E-06	0.283709	1	0.994	0.126422	CenC
RPPH1	7.05E-06	0.303445	0.429	0.136	0.12843	CenC
CIAO1	7.21E-06	0.335832	0.607	0.254	0.131365	CenC
FAM134A	7.46E-06	0.254647	0.607	0.233	0.13584	CenC
WDTC1	7.74E-06	0.257356	0.286	0.069	0.140883	CenC
NDRG2	7.98E-06	0.378307	0.714	0.357	0.145312	CenC
ENSG0000019893[illegible]	8.00E-06	0.363261	1	0.998	0.145673	CenC
NDUFA8	8.02E-06	0.464127	0.857	0.578	0.145974	CenC
GAS6	8.06E-06	0.270701	0.643	0.259	0.146686	CenC
MRPL23	8.26E-06	0.36998	0.929	0.637	0.150396	CenC
HAGH	8.97E-06	0.434386	0.75	0.452	0.163403	CenC
NOL3	9.17E-06	0.422134	0.679	0.35	0.166963	CenC
RPS26	9.37E-06	0.483402	1	0.943	0.170616	CenC

RAB13	9.76E-06	0.487042	0.857	0.592	0.177656	CenC
NDUFC1	9.95E-06	0.478383	1	0.915	0.181113	CenC
SLIRP	1.06E-05	0.41237	1	0.881	0.19311	CenC
FAM84B	1.06E-05	0.380014	0.5	0.188	0.193787	CenC
GHITM	1.10E-05	0.41546	0.964	0.854	0.199926	CenC
TCEAL1	1.11E-05	0.302925	0.5	0.189	0.20204	CenC
CD320	1.11E-05	0.277706	0.321	0.089	0.202449	CenC
FKBP2	1.14E-05	0.416027	0.964	0.788	0.207006	CenC
ADAR	1.29E-05	0.250393	0.321	0.091	0.235703	CenC
UQCR10	1.30E-05	0.41691	1	0.903	0.235981	CenC
CRABP2	1.33E-05	0.34717	0.679	0.285	0.242333	CenC
SPRYD3	1.40E-05	0.302326	0.464	0.17	0.25447	CenC
SMS	1.47E-05	0.608375	0.786	0.485	0.26685	CenC
MRPL12	1.48E-05	0.524854	0.714	0.402	0.269709	CenC
PLA2G16	1.49E-05	0.363656	0.5	0.191	0.271622	CenC
ATP6V1A	1.52E-05	0.316175	0.429	0.153	0.276572	CenC
BAG6	1.53E-05	0.296386	0.571	0.243	0.277835	CenC
PURA	1.64E-05	0.285609	0.536	0.226	0.298527	CenC
NDUFAF3	1.73E-05	0.414715	0.786	0.523	0.314307	CenC
COA6	1.80E-05	0.343015	0.607	0.274	0.328415	CenC
PFDN5	1.94E-05	0.313454	1	0.974	0.353176	CenC
IFIT1	1.98E-05	0.454575	0.464	0.176	0.360994	CenC
MTFP1	1.99E-05	0.260552	0.321	0.095	0.363059	CenC
TOMM20	2.01E-05	0.459902	0.893	0.723	0.365828	CenC
ZNF428	2.02E-05	0.425556	0.75	0.398	0.367157	CenC
TMEM223	2.07E-05	0.281599	0.464	0.174	0.376044	CenC
NDUFA11	2.11E-05	0.445748	0.929	0.875	0.384352	CenC
EIF1AY	2.16E-05	0.653688	0.429	0.172	0.393375	CenC
GGT7	2.29E-05	0.254791	0.357	0.104	0.417864	CenC
ROMO1	2.30E-05	0.451475	0.964	0.782	0.41845	CenC
NDUFS5	2.30E-05	0.387438	1	0.899	0.418734	CenC
KCNQ1OT1	2.32E-05	0.347548	0.536	0.224	0.423165	CenC
CISD1	2.35E-05	0.447385	0.714	0.386	0.427337	CenC
OAZ2	2.35E-05	0.404377	0.786	0.444	0.42844	CenC
MMP24-AS1	2.36E-05	0.338458	0.571	0.24	0.430173	CenC
POLB	2.38E-05	0.262692	0.321	0.095	0.433384	CenC
FAM115A	2.57E-05	0.28058	0.429	0.149	0.468647	CenC
LSMD1	2.58E-05	0.421031	0.929	0.813	0.469209	CenC
CCNL1	2.60E-05	0.458299	0.964	0.806	0.473744	CenC
RPS19	2.64E-05	0.279873	1	0.998	0.481024	CenC
MDK	2.72E-05	0.253288	0.679	0.284	0.495198	CenC
MAP2K1	2.73E-05	0.351217	0.536	0.236	0.496413	CenC
RPL37A	2.78E-05	0.385732	1	0.998	0.506164	CenC
MYCL1	2.89E-05	0.292135	0.393	0.132	0.52668	CenC
SDHD	2.94E-05	0.376469	0.893	0.69	0.534854	CenC
PUM1	2.96E-05	0.25324	0.536	0.201	0.538271	CenC
HSPE1	3.04E-05	0.359639	0.964	0.852	0.553521	CenC
FAM200B	3.13E-05	0.40295	0.857	0.56	0.569406	CenC

HIGD1A	3.26E-05	0.37833	0.857	0.655	0.593015	CenC
LRP1	3.30E-05	0.277385	0.607	0.253	0.601262	CenC
ATP5C1	3.54E-05	0.431759	0.964	0.827	0.644049	CenC
ZFAND5	3.80E-05	0.384085	0.929	0.664	0.692187	CenC
APOO	3.98E-05	0.27704	0.5	0.197	0.723984	CenC
NQO2	4.01E-05	0.2773	0.821	0.4	0.730352	CenC
AK1	4.18E-05	0.359511	0.643	0.299	0.760983	CenC
SMDT1	4.43E-05	0.359017	0.964	0.721	0.806149	CenC
MAGED2	4.60E-05	0.373033	0.786	0.423	0.838084	CenC
AKR1C3	4.77E-05	0.282646	0.357	0.117	0.868035	CenC
PDE4D	4.80E-05	0.305118	0.321	0.101	0.873536	CenC
BC009467	4.85E-05	0.274105	0.429	0.161	0.883977	CenC
DHCR24	4.97E-05	0.413674	0.464	0.194	0.905735	CenC
GOLGA8B	5.34E-05	0.259085	0.357	0.114	0.971412	CenC
FLJ10038	5.55E-05	0.306555	0.607	0.274	1	CenC
BOLA3	5.60E-05	0.397496	0.679	0.338	1	CenC
PRDX3	5.73E-05	0.382639	0.821	0.47	1	CenC
SLITRK4	5.73E-05	0.300071	0.607	0.28	1	CenC
PTMS	5.76E-05	0.343689	0.893	0.541	1	CenC
MTFR1L	5.83E-05	0.287427	0.607	0.278	1	CenC
NCOA7	5.95E-05	0.304927	0.5	0.203	1	CenC
ATP5J2	6.21E-05	0.371228	1	0.921	1	CenC
RPL24	6.38E-05	0.284228	1	0.994	1	CenC
MYL6B	6.84E-05	0.474229	0.75	0.453	1	CenC
PSIP1	6.97E-05	0.314099	0.571	0.261	1	CenC
MRPS9	7.20E-05	0.271241	0.607	0.277	1	CenC
ETS2	7.21E-05	0.524911	0.643	0.358	1	CenC
IDI1	7.33E-05	0.39116	0.75	0.476	1	CenC
C12orf57	7.54E-05	0.390668	0.964	0.742	1	CenC
MRPL34	7.63E-05	0.417594	0.821	0.517	1	CenC
GCAT	7.78E-05	0.267642	0.393	0.142	1	CenC
GAS5	7.83E-05	0.367788	1	0.934	1	CenC
SOD1	8.36E-05	0.329742	1	0.9	1	CenC
NDUFV1	8.49E-05	0.375106	0.821	0.578	1	CenC
C7orf50	8.71E-05	0.413354	0.643	0.321	1	CenC
ARRDC3	9.20E-05	0.307511	0.607	0.288	1	CenC
SPTY2D1	9.21E-05	0.305584	0.357	0.123	1	CenC
VKORC1	9.24E-05	0.340652	0.75	0.411	1	CenC
FASN	9.24E-05	0.262451	0.286	0.087	1	CenC
EID1	9.25E-05	0.418351	0.857	0.884	1	CenC
IRF3	9.43E-05	0.27544	0.5	0.215	1	CenC
LYRM4	9.43E-05	0.353359	0.607	0.323	1	CenC
HNRNPL	9.49E-05	0.307168	0.5	0.222	1	CenC
RPL5	9.91E-05	0.278194	1	0.993	1	CenC
MTRNR2L8	0.000101	0.285493	0.357	0.128	1	CenC
PTGR1	0.000102	0.285668	0.786	0.402	1	CenC
LOC100505806	0.000105	0.273394	0.714	0.35	1	CenC
TTC3	0.000105	0.2915	0.607	0.281	1	CenC

LAMTOR5	0.000106	0.377475	1	0.897	1	CenC
EEF2	0.000107	0.353525	1	0.874	1	CenC
ARHGEF3	0.000119	0.372405	0.714	0.355	1	CenC
SAT2	0.000125	0.458164	0.679	0.451	1	CenC
PIGH	0.000126	0.341345	0.607	0.322	1	CenC
HSPA9	0.000129	0.410495	0.857	0.668	1	CenC
BRD2	0.000133	0.380503	0.857	0.596	1	CenC
C11orf71	0.000139	0.297642	0.464	0.181	1	CenC
ARHGDIB	0.000146	0.427596	0.679	0.439	1	CenC
FLNA	0.000161	0.259959	0.393	0.147	1	CenC
CMC1	0.000161	0.253399	0.643	0.316	1	CenC
FDPS	0.000175	0.321569	0.857	0.514	1	CenC
SMIM11	0.000184	0.286674	0.571	0.268	1	CenC
NCBP2-AS2	0.000196	0.349157	0.75	0.488	1	CenC
ALDOA	0.000198	0.304503	1	0.978	1	CenC
CHID1	0.0002	0.25127	0.643	0.302	1	CenC
C9orf123	0.000204	0.386129	0.929	0.619	1	CenC
RPL36AL	0.000206	0.385776	0.964	0.955	1	CenC
CUTC	0.00021	0.277098	0.464	0.203	1	CenC
TRIM2	0.000215	0.293697	0.321	0.112	1	CenC
RBBP6	0.00022	0.323556	0.714	0.383	1	CenC
MRPL40	0.00022	0.316803	0.857	0.543	1	CenC
AURKAIP1	0.000222	0.318198	0.929	0.75	1	CenC
FCGRT	0.000247	0.330496	0.643	0.31	1	CenC
RNF5	0.000256	0.328787	0.714	0.439	1	CenC
PCYOX1	0.000257	0.277866	0.536	0.269	1	CenC
TFRC	0.000257	0.266028	0.429	0.177	1	CenC
SNHG16	0.000268	0.326722	0.821	0.512	1	CenC
ZSWIM7	0.000268	0.369027	0.643	0.364	1	CenC
ANKRD37	0.000274	0.510456	0.5	0.245	1	CenC
CANX	0.000287	0.409413	0.857	0.608	1	CenC
SPAG7	0.000295	0.352723	0.821	0.581	1	CenC
NEDD8	0.000306	0.275501	1	0.885	1	CenC
REST	0.000309	0.381786	0.571	0.29	1	CenC
BEX4	0.000315	0.28725	0.857	0.587	1	CenC
ETFB	0.000354	0.383225	0.821	0.7	1	CenC
SLC25A3	0.000354	0.296487	1	0.91	1	CenC
JUND	0.000358	0.355901	0.821	0.64	1	CenC
PDHA1	0.000364	0.285232	0.643	0.352	1	CenC
NDUFA12	0.000366	0.336037	0.857	0.782	1	CenC
LDHA	0.000375	0.282877	1	0.99	1	CenC
MRPL14	0.000403	0.37944	0.75	0.502	1	CenC
ATAD3C	0.000407	0.258595	0.393	0.161	1	CenC
TMEM100	0.000428	0.314	0.607	0.296	1	CenC
TSPYL2	0.000433	0.340709	0.5	0.244	1	CenC
TMEM14B	0.000438	0.281161	0.893	0.676	1	CenC
NUTF2	0.00044	0.255888	0.643	0.348	1	CenC
ESD	0.00045	0.353108	0.821	0.534	1	CenC

JMJD6	0.00046	0.28282	0.571	0.296	1	CenC
PPIA	0.000467	0.307234	1	0.96	1	CenC
THAP7	0.000469	0.271877	0.536	0.261	1	CenC
HNRNPU	0.000477	0.32827	0.929	0.712	1	CenC
ATP6V1G1	0.000515	0.26338	1	0.965	1	CenC
IFT27	0.000542	0.321493	0.5	0.262	1	CenC
RPS21	0.0006	0.279396	1	0.99	1	CenC
MRPS36	0.000646	0.389268	0.714	0.539	1	CenC
HSPA8	0.000695	0.331867	0.964	0.792	1	CenC
COPRS	0.000732	0.292908	0.464	0.226	1	CenC
GADD45G	0.000736	0.269585	0.679	0.411	1	CenC
SLC25A1	0.000743	0.251038	0.5	0.25	1	CenC
SRSF9	0.000758	0.272947	0.964	0.833	1	CenC
MRPS21	0.000779	0.307125	0.929	0.759	1	CenC
COMTD1	0.000784	0.294289	0.571	0.292	1	CenC
MRPL2	0.000821	0.318242	0.571	0.319	1	CenC
EIF4B	0.000829	0.358478	0.714	0.491	1	CenC
NDN	0.000848	0.282823	0.607	0.335	1	CenC
COX8A	0.000857	0.340507	0.964	0.85	1	CenC
NINJ1	0.000858	0.318459	0.607	0.325	1	CenC
PLOD1	0.00087	0.269272	0.393	0.16	1	CenC
UQCRFS1	0.0009	0.336295	0.964	0.801	1	CenC
KLHDC8B	0.000922	0.272153	0.321	0.123	1	CenC
SNRPE	0.000938	0.273181	1	0.822	1	CenC
TRAPPC5	0.00098	0.373415	0.857	0.723	1	CenC
NFS1	0.00103	0.291161	0.357	0.159	1	CenC
GLO1	0.001039	0.270828	0.893	0.622	1	CenC
SSBP4	0.001057	0.258587	0.357	0.15	1	CenC
PHLDA1	0.001132	0.61648	0.429	0.207	1	CenC
MSS51	0.001143	0.268966	1	0.979	1	CenC
CSRNP1	0.001263	0.344482	0.643	0.416	1	CenC
MYLIP	0.001279	0.266305	0.607	0.352	1	CenC
FUNDC2	0.00132	0.275277	0.679	0.426	1	CenC
COA4	0.00133	0.264366	0.786	0.47	1	CenC
GABARAPL2	0.001332	0.27775	0.964	0.877	1	CenC
FKBPL	0.001359	0.273761	0.321	0.131	1	CenC
PIN1	0.001372	0.260012	0.786	0.509	1	CenC
TIPARP	0.001435	0.478564	0.607	0.378	1	CenC
MRPL20	0.001464	0.317067	0.929	0.753	1	CenC
FIS1	0.001491	0.345509	0.893	0.681	1	CenC
LOC100288911	0.001557	0.355797	0.429	0.201	1	CenC
SDHB	0.001591	0.354275	0.786	0.618	1	CenC
JTB	0.001744	0.302555	0.964	0.743	1	CenC
NHP2L1	0.001859	0.288509	0.929	0.833	1	CenC
NDUFA2	0.001891	0.327969	0.857	0.777	1	CenC
HERPUD1	0.001909	0.352132	0.929	0.942	1	CenC
TMEM107	0.001917	0.390894	0.5	0.285	1	CenC
AAMDC	0.001925	0.376748	0.679	0.415	1	CenC

C19orf60	0.001969	0.314045	0.714	0.52	1	CenC
MOAP1	0.001978	0.256509	0.571	0.304	1	CenC
PLEKHA3	0.001998	0.310716	0.464	0.234	1	CenC
PELO	0.002276	0.389017	0.5	0.283	1	CenC
IL8	0.002316	0.59613	0.286	0.108	1	CenC
MRPS7	0.002534	0.2698	0.75	0.503	1	CenC
SETD5-AS1	0.002638	0.32034	0.5	0.27	1	CenC
RNF181	0.002648	0.334803	0.857	0.718	1	CenC
RNASEH2C	0.002761	0.269099	0.607	0.335	1	CenC
ALKBH7	0.002804	0.30123	0.929	0.656	1	CenC
NFKBIE	0.002992	0.255193	0.321	0.142	1	CenC
PPP1R10	0.003447	0.263097	0.5	0.279	1	CenC
NEDD4L	0.003709	0.253887	0.5	0.272	1	CenC
GADD45B	0.003908	0.268045	1	0.928	1	CenC
GRHPR	0.003992	0.321848	0.75	0.54	1	CenC
PNKD	0.004224	0.339356	0.821	0.746	1	CenC
WDR1	0.004412	0.333921	0.786	0.555	1	CenC
TUFM	0.004433	0.251027	0.893	0.751	1	CenC
ZFAS1	0.004501	0.297529	1	0.866	1	CenC
TUBB	0.004827	0.253765	0.929	0.828	1	CenC
CKB	0.004929	0.410882	0.607	0.355	1	CenC
7SK-87	0.005131	0.548256	0.821	0.609	1	CenC
DARS	0.005392	0.373126	0.75	0.534	1	CenC
PRDX6	0.005535	0.254395	1	0.855	1	CenC
ECI2	0.00582	0.331668	0.679	0.439	1	CenC
TOMM5	0.006443	0.281451	0.857	0.724	1	CenC
CXCL2	0.006988	0.299158	0.464	0.235	1	CenC
GOLGB1	0.00857	0.262052	0.607	0.373	1	CenC
FUS	0.008951	0.2615	0.786	0.525	1	CenC

Molecular characteristics and spatial distribution of adult human corneal cell subtypes

Ann J Ligocki, Wen Fury, Christian Gutierrez, Christina Adler, Tao Yang, Min Ni, Yu Bai, Yi Wei, Guillermo L Lehmann, Carmelo Romano

Supplementary Table 2. CA3, RGS5, and PDGFRB gene expression levels

Average UMI

gene	CenC	LPC-1	LPC-2	Epi-B1	Epi-B2	Epi-T	Epi-S1	Epi-S2	Epi-S3	Conj-1	Conj-2	Conj-3	Stro	LC	Mela	VEC
CA3	59.3842	0.007257	0.008556	0.01023	0.004089	0.007236	0.004859	0.010335	0.008295	0.028764	0.02266	0.054414	0.007751	0.02422	0	0
RGS5	17.77359	0.017267	0.01673	0.03779	0.03627	0.020661	0.033466	0.055378	0.040581	0.0282	0.026579	0.021474	0.021251	0	0	0.44411
PDGFRB	0.064965	0	0	0.006185	0	0	0.002378	0.000374	0.001348	0.000635	0	0	0.263109	0	0	1.019454
